# *HvPAA1* Encodes a P-Type ATPase, a Novel Gene for Cadmium Accumulation and Tolerance in Barley (*Hordeum vulgare* L.)

**DOI:** 10.3390/ijms20071732

**Published:** 2019-04-08

**Authors:** Xin-Ke Wang, Xue Gong, Fangbin Cao, Yizhou Wang, Guoping Zhang, Feibo Wu

**Affiliations:** 1 Department of Agronomy, College of Agriculture and Biotechnology, Zijingang Campus, Zhejiang University, Hangzhou 310058, China; ke87795593@163.com (X.-K.W.); caofangbin@zju.edu.cn (F.C.); wangyizhou@zju.edu.cn (Y.W.); zhanggp@zju.edu.cn (G.Z.); 2 School of Agriculture, Food and Wine, the University of Adelaide, Waite Campus, Adelaide 5064, Australia; redchian123@gmail.com; 3 Jiangsu Co-Innovation Center for Modern Production Technology of Grain Crops, Yangzhou University, Yangzhou 225009, China

**Keywords:** barley (*Hordeum vulgare* L.), BSMV-VIGS, cadmium tolerance and accumulation, *HvPAA1*, quantitative trait loci (QTLs)

## Abstract

The identification of gene(s) that are involved in Cd accumulation/tolerance is vital in developing crop cultivars with low Cd accumulation. We developed a doubled haploid (DH) population that was derived from a cross of Suyinmai 2 (Cd-sensitive) × Weisuobuzhi (Cd-tolerant) to conduct quantitative trait loci (QTL) mapping studies. We assessed chlorophyll content, traits that are associated with development, metal concentration, and antioxidative enzyme activity in DH population lines and parents under control and Cd stress conditions. A single QTL, designated as *qShCd7H*, was identified on chromosome 7H that was linked to shoot Cd concentration; *qShCd7H* explained 17% of the phenotypic variation. Comparative genomics, map-based cloning, and gene silencing were used in isolation, cloning, and functional characterization of the candidate gene. A novel gene *HvPAA1*, being related to shoot Cd concentration, was identified from *qShCd7H*. Sequence comparison indicated that *HvPAA1* carried seven domains with an N-glycosylation motif. *HvPAA1* is predominantly expressed in shoots. Subcellular localization verified that HvPAA1 is located in plasma membrane. The silencing of *HvPAA1* resulted in growth inhibition, greater Cd accumulation, and a significant decrease in Cd tolerance. We conclude *HvPAA1* is a novel plasma membrane-localized ATPase that contributes to Cd tolerance and accumulation in barley. The results provide us with new insights that may aid in the screening and development of Cd-tolerant and low-Cd-accumulation crops.

## 1. Introduction

Cadmium (Cd) is highly toxic to organisms, even at low concentrations. Cd has relatively high bioavailability in soil when compared with other heavy metals, and it is readily taken up by plants, which is a threat to human health via the food chain [1,2]. Therefore, it is imperative to identify the genotypes with less Cd uptake from soil and the relevant genes for developing crop cultivars with reduced Cd uptake and accumulation.

Quantitative trait loci (QTLs) that contribute to Cd accumulation have been reported in wheat [3], rice [4,5], maize [6], soybean [7], and barley [8]. A major Cd-specific QTL has been identified on the short arm of chromosome 7 in rice [4], and major QTLs that contribute to Cd tolerance during seedling stage found in *Arabidopsis* [9] and maize [6]. A major QTL that affects Cd accumulation in seeds was detected in soybean [7], and a major QTL that contributes to low Cd uptake was mapped to the 5BL chromosome in wheat [3,10]. Studies have been conducted with the goal of identifying Cd-specific genes. For example, Zhao et al. [11] performed a comparative transcriptome analysis and found that Cd stress induced the expression of general and specific genes in *Arabidopsis thaliana* roots. Villiers et al. [12] found that Cd-detoxifying proteins, such as phytochelatin synthase (PCS), antioxidative enzymes, and glutathione S-transferases (GST), were upregulated in plants that were subject to Cd stress. The loss of function of *CAX1* in *A. thaliana* resulted in higher Cd sensitivity in the presence of low concentrations of Cd [13]. Our previous study identified several transporter genes, including *HvZIP3* and *HvZIP8*, which are associated with low grain Cd accumulation [14]. Elevated expression of the heavy metal transporting ATPase 4 (HMA4), which is a P_1B_-type ATPase, increased the Cd tolerance in plants that were subject to Cd stress resulting from the accumulation of low levels of cellular Cd in the cytoplasm [9]. Despite the identification of these genes, the molecular mechanisms underlying Cd tolerance and accumulation are still unclear at the present time.

P-type ATPases are transmembrane proteins that play a crucial role in the transport of a wide variety of cations across membranes and they are vital for ion homeostasis and heavy metal detoxification [15]. Plant P_1B_ ATPases have been classified that are expected to be involved in the transport of heavy metals. Multiple alignments between all of the P_1B_ ATPase sequences indicate that HMA1, HMA2, HMA3, and HMA4 in *Arabidopsis* likely to serve as Zn^2+^/Co^2^+/Cd^2+^/Pb^2+^ ATPases, while RAN1, PAA1, and HMA5 are candidate Cu^2+^/Ag^2+^ ATPases [16]. However, *PAAs* are poorly characterized in barley, and their function, structure, and expression remain unclear. 

In this study, we developed a doubled haploid (DH) barley population that was derived from a cross of Cd-tolerant and Cd-sensitive parents to identify the genes that are associated with Cd tolerance and accumulation, which were then characterized in terms of function. As a result, a novel p-type ATPase, *HvPAA1*, was identified and then cloned for the first time. Furthermore, the function of *HvPAA1* was investigated using BSMV-VIGS (barley stripe mosaic virus- virus induced gene silencing) system. The results showed that *HvPAA1* plays a vital role in the detoxification of Cd in barley. 

## 2. Results

### 2.1. Transgressive Segregation was Observed for Most Investigated Traits

Table 1 summarizes the means, ranges, skewness, and kurtosis of the 17 assessed traits. In general, the values of the growth traits were reduced in the presence Cd stress relative to the control for the parental and DH lines. The coefficient variance (CV) of these parameters for the DH lines ranged between 4.2%-48.86% in the control and between 6.2%–119.43% in 10 µM Cd (Table 1). The Cd tolerance index for 11 traits revealed that Suyinmai 2 exhibited a greater reduction in growth than Weisuobuzhi (Table 1). In general, Cd decreased APX activity in shoots and roots and guaiacol peroxidase (POD) activity in shoots in both parental and DH lines. However, POD and catalase (CAT) activities in roots reflected differences in CTI between Suyinmai 2 and Weisuobuzhi. The kurtosis and the skewness for the majority of the traits examined was less than 1, with the exception of the shoot Zn concentration in the presence of 10 μM Cd, which indicated that the variations in these traits were consistent with a normal distribution. 

### 2.2. Cd, Zn and Mn Concentrations 

**Figure 1:**
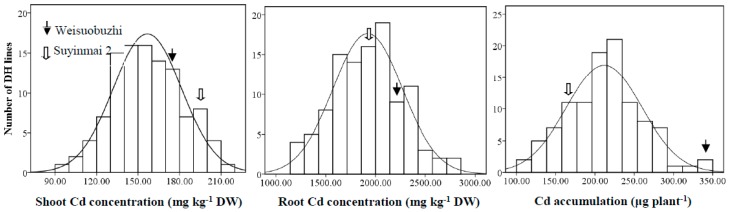
Frequency distribution of cadmium (Cd) concentration in shoots and roots, and Cd accumulation per plant. Parents and 108 DH lines were grown in a nutrient solution containing 10 μM CdCl_2_ for 15 days. The data are represented as the mean of three replicates obtained from the spatial analysis. The solid arrow represents Weisuobuzhi (Cd-tolerant genotype) and the hollow arrow represents Suyinmai 2 (Cd-sensitive).

Concentrations of Cd, Zn, and Mn in shoots and roots were measured under both control and Cd stress conditions; however, no Cd was detected in the control. Shoot Cd concentration in the tolerant parent, Weisuobuzhi, was significantly lower than that in the sensitive parent, Suyinmai 2, in the presence of 10 µM Cd. After treatment with 10 µM Cd, a four-fold phenotypic variation in Sh_Cd_ was observed that ranged between 74.2–277.6 mg kg^−1^ with an average of 164.26 mg kg^−1^. In contrast, root R_Cd_ was significantly higher in Weisuobuzhi than in Suyinmai 2. An approximately three-fold phenotypic variation in Cd concentration in the DH lines was observed in roots that ranged between 1161.0–2749.7 mg kg^−1^, with an average of 1923.77 mg kg^−1^. Cd accumulation was markedly higher in Suyinmai 2 than in Weisuobuzhi. Of note, most of the DH lines accumulated less Cd than the parental line (Weisuobuzhi; Figure 1). 

The Zn and Mn concentrations significantly differed between the parental and DH lines in both shoots and roots, and significantly higher concentrations were observed in roots than in shoots. The 10 µM Cd stress decreased the Zn concentration in shoots in Weisuobuzhi but not in Suyinmai 2. However, Cd stress resulted in an increase in Zn concentration in roots from both the parental and DH lines. In DH lines, an approximately 2.7-fold variation was observed for Sh_Mn_ and Sh_Zn_, in comparison with 3.3-fold and 4.0-fold variation for R_Mn_ and R_Zn_, respectively, in 10 µM Cd (Table 1). 

### 2.3. Genetic Linkage Maps

Within the linkage map, a total of 1572 markers (1532 GBS and 40 SSR markers) were successfully assigned to the seven barley chromosomes. The total length of the map was 1134.5 cM, the length of each chromosome varied from 126.7 cM to 238.59 cM, and the number of markers per chromosome varied from 50 to 549. The average distance between two adjacent makers was 0.72 cM and it ranged between 0.45 to 2.77 cM (Figure S1). 

### 2.4. Collocations of QTLs were Found for the Investigated Traits

**Figure 2:**
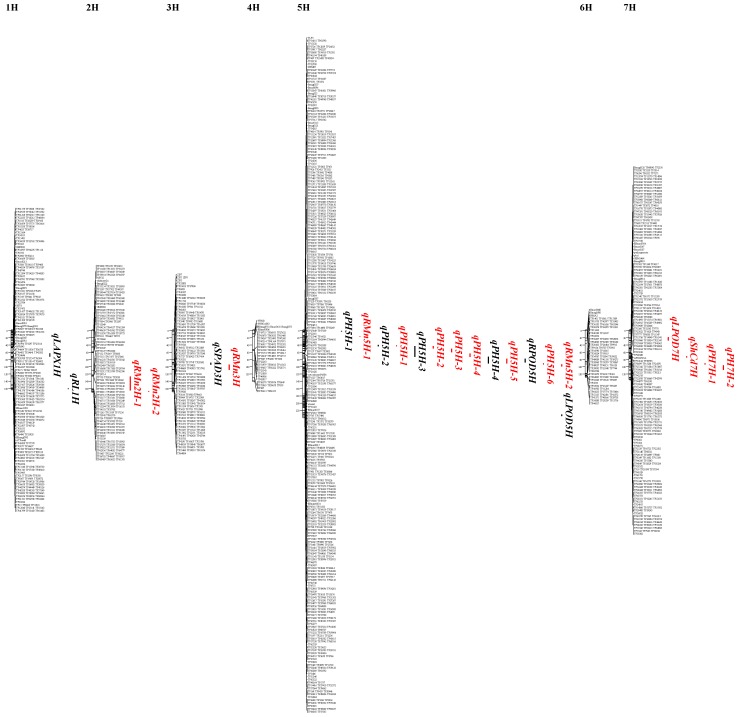
Quantitative trait loci (QTL) significantly associated with different parameters linked to cadmium (Cd) tolerance in the Suyinmai 2 × Weisuobuzhi doubled haploid (DH) population obtained using the multiple QTL model (MQM) mapping method. QTLs in black and red were detected in control and 10 µM Cd conditions, respectively. The one- and two-logarithm of odds (LOD) support intervals for each of the QTLs, as calculated in Mapchart, are displayed on the right side of each linkage group.

Among the 17 traits that were investigated in the three conditions (control, Cd treatment and CTI), QTLs were identified for eight traits: the chloroform content presented as SPAD (Soil and Plant Analyzer Development) value, plant height, root length, leaf and root POD, leaf APX, Mn concentration in root, and Cd concentration in shoot. A total of 24 QTLs were detected from this study, 15 of which were from Cd stress; the majority of the QTLs were resolved within 10 cM (Table 2, Figure 2). The majorities of the QTLs identified during this study were for plant height and Mn concentration and it explained 10% to 17% of the phenotypic variation (Table 2). In terms of the additive effect, Weisuobuzhi’s allele increased the plant height, root length, and leaf APX, regardless of QTL positions. Suyinmai 2’s allele increased SPAD and POD values, regardless of plant tissues and Cd treatments. Some QTLs for plant height were mapped to the same positions under control and Cd stress on chromosome 5H and Weisuobuzhi’s allele increased plant height. QTLs for leaf POD were also detected under both the control and Cd stress, but they were mapped to different chromosomal regions. The QTLs were detected on chromosomes 2H, 3H, and 5H for Mn concentration in root under Cd stress. These QTLs had a similar effect that explained around 13% of phenotypic variation. The Weisuobuzhi allele increase Mn concentration on chromosomes 2H and 3H, and the Suyinmai 2 allele increased the Mn concentration on chromosome 5H.

### 2.5. A single QTL was Identified on Chromosome 7H for Shoot Cd Concentration

**Figure 3:**
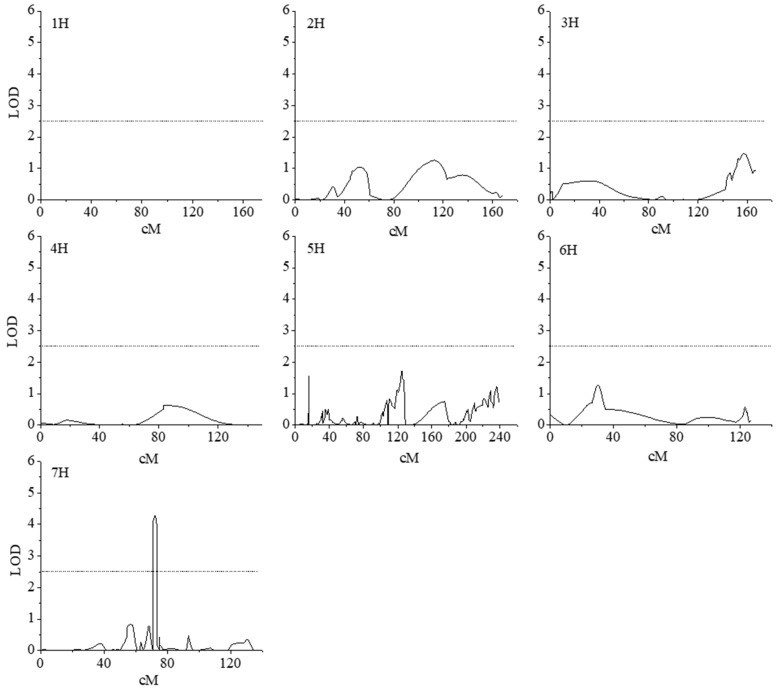
Logarithm of odds (LOD) score profiles for Cd tolerance in terms of shoot Cd concentration. The map positions are indicated along the abscissa. The LOD scores are indicated along the *y*-axis. The dashed lines represent the LOD score threshold (2.5) at a 0.05 error level for QTL detection.

A single QTL for Cd concentration in shoots was identified on chromosome 7H in Cd stress conditions that had the greatest effects among all QTLs. This QTL explained 17% of phenotypic variation (Figure 3); we designated this QTL *qShCd7H*. The Suyinmai 2 allele increased the Cd concentration in shoots by 11% in comparison to the Weisuobuzhi allele (Table 2). The *qShCd7H* QTL was mapped to a 2.6 cM region between the GBS markers TP18054 and TP11089 (Table S2). The 2.6 cM region included approximately 60 genes that were annotated in the Barley Map and IBCS genome database (Table S4). As a result, AK355848.1 was cloned, sequenced, and functionally analyzed in the parents Suyinmai 2 and Weisuobuzhi, as well as the check variety Zhenong 8.

### 2.6. Cloning, Sequencing and Functional Analysis of A Candidate Gene Designated HvPAA1

**Figure 4:**
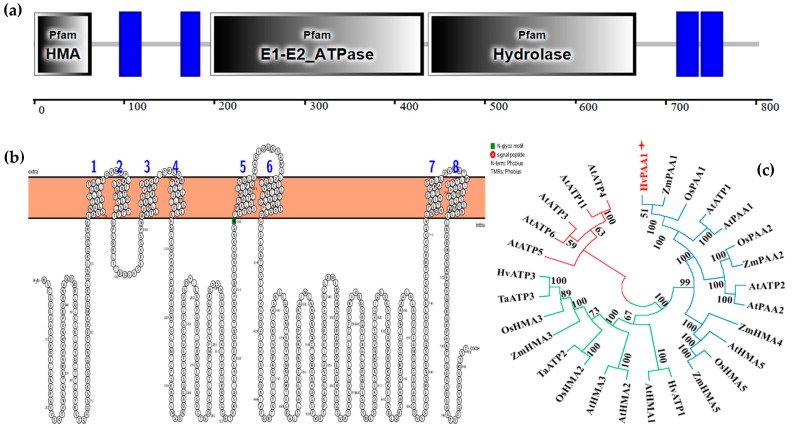
Protein structure and phylogenetic analysis of HvPAA1. (**a**) Amino acid sequence analysis based on the SMATR database. Some features and domains are not shown due to overlap with other annotations. (**b**) Amino acid sequence analysis based on the Protter database. (**c**) Phylogenetic tree constructed in MEGA7 using the neighbor-joining algorithm analysis method.

The full length of the cloned cDNA was 2412 bp and its encoded protein contains 803 amino acids and it has a predicted molecular weight of 85.1 KDa and a theoretical pI of 6.35 (Figure S2). The parents shared the same amino acid and CDS sequence (Figure S4). There are seven important domains, including four transmembrane domains, one HMA domain, one E1-E2_ATPase domain, and one HAD domain, and, most importantly, an N-glycosylation motif (Figure 4). The predicted sequence of *HvPAA1* between the transcription start site and the termination site showed lengthy and diverse introns (Figure S5). Furthermore, the alignment result (Figure S6) showed that Weisuobuzhi and Suyinmai 2 contain four single nucleotide polymorphism sites (SNPs), all of which are located in intron. Polygenetic analysis showed that the 28 genes were clustered into three major groups; the cloned gene was a member of the group that was composed of ATP1, ATP2, HMA4, HMA5, PAA1, and PAA2. The cloned sequences of Suyinmai 2, Weisuobuzhi, and Zhenong 8 were mostly similar to those from *OsPAA1* (*Oryza sativa* L.) and *ZmPAA1* (*Zea mays* L.) and they showed 89.7%, 88.1%, and 68.3% similarity to those from *OsPAA1*, *ZmPAA1*, and *AtPAA1*, respectively (Figure S3). As a result, we designated this gene as barley *HvPAA1*.

### 2.7. Expression Pattern and Subcellular Localization of HvPAA1

**Figure 5:**
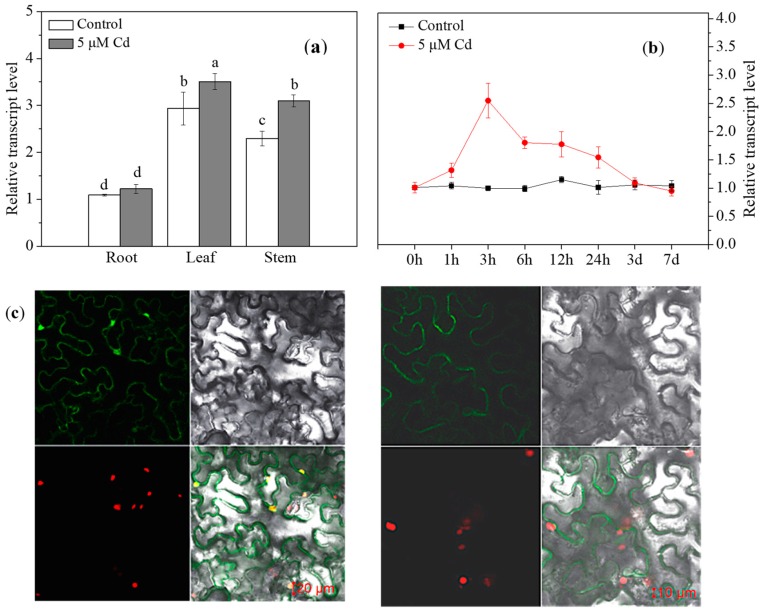
Tissue expression and time-dependent expression patterns of HvPAA1 and the subcellular localization of HvPAA1. (**a**,**b**) qRT-PCR analysis of the relative transcript levels of HvPAA1 in different tissues and at different times in Zhenong 8. (**c**) Transient expression of GFP and HvPAA1-sGFP fusion protein in tobacco. Microscopic image on left shows GFP; the image on right shows the HvPAA1-sGFP fusion protein. The results represent an average (±SE) from three independent experiments. Different letters indicate significant differences among the treatments within three sampling data according to Duncan’s multiple range test with *p* < 0.05. The error bars represent the SE values.

Exposure to 5 µM Cd significantly promoted the relative levels of *HvPAA1* transcript in leaves and stems, and the leaves were found to have higher levels than stems (Figure 5a). The relative level of *HvPAA1* transcript peaked at 3 h after Cd exposure and then sharply decreased to the same level that was observed at 0 h after 3 d and 7 d (Figure 5b). To determine the subcellular localization of HvPAA1, we transiently expressed HvPAA1–GFP (green fluoresence protein) in a nuclear marker-containing *Nicotiana benthamiana* line that expressed red fluorescent protein (RFP) that was fused to histone 2B (H2B) [17]. Based on the GFP signal, the HvPAA1 protein was localized to plasma membrane (Figure 5c). 

### 2.8. Phenotypic Analysis and Cd Concentration in BSMV-Inoculated Lines

**Figure 6:**
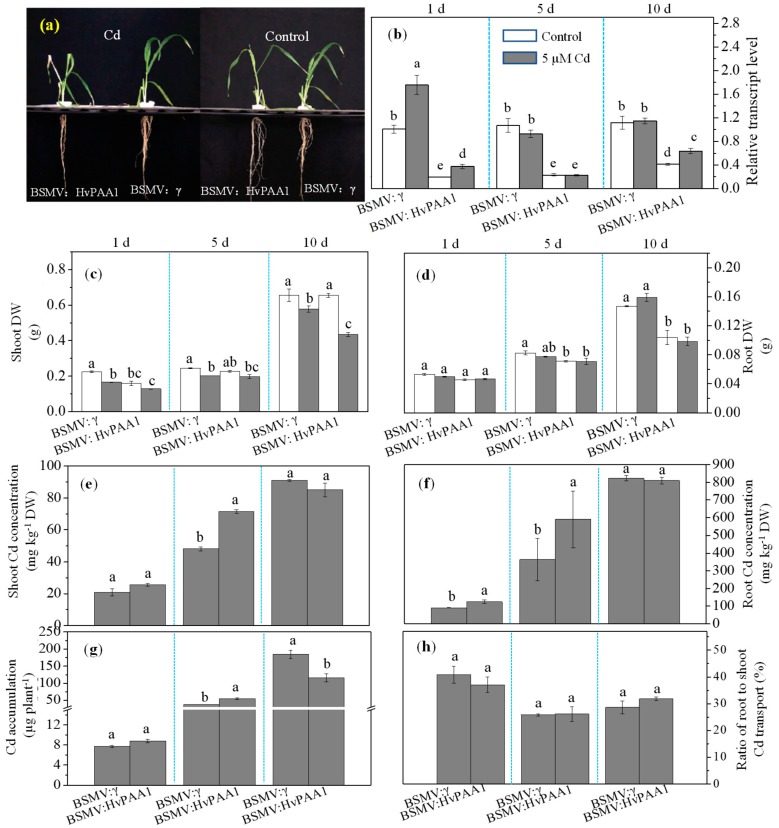
Functional assessment of *HvPAA1* in Zhenong 8 using BSMV-VIGS (barley stripe mosaic virus- virus induced gene silencing). (**a**) Morphology of mock- or BSMV: HvPAA1-inoculated Zhenong 8 seedlings after 10 days of exposure to 5 µM Cd. Scale bars = 6 cm. (**b**) RT-PCR analysis of the relative transcript levels of *HvPAA1* in Zhenong 8 leaves. Different letters indicate significant differences among the treatments within 3 sampling data (1, 5, 10 d after the initiation of Cd stress) according to Duncan’s multiple range test with *p* < 0.05. (**c**,**d**) Dry weights of mock- and BSMV:HvPAA1-inoculated Zhenong 8 plants. (**e**,**f**) Cd concentrations of mock- and BSMV:HvPAA1-inoculated Zhenong 8 plants grown in 5 µM Cd. (**g**) Cd accumulation in mock- and BSMV:HvPAA1-inoculated Zhenong 8 plants grown in 5 µM Cd. (**h**) root-to-shoot Cd transport ratio. Error bars represent the SE values. Different letters indicate significant differences between control and Cd treatment, between each sampling date, and between the mock- and BSMV:HvPAA1 inoculated seedlings according to Duncan’s multiple range tests with *p* < 0.05.

To clarify the function of *HvPAA1*, a BSMV-VIGS-based gene silencing technology was used to determine whether the silencing of this gene at the mRNA level affected Cd concentration in barley. Cd exposure increased the transcript level of *HvPAA1* in both mock- and BSMV:HvPAA1-inoculated Zhenong 8 plants at 1 dpi when compared with the controls (Figure 6b). However, mock-inoculated seedlings that were treated with Cd exhibited a similar expression pattern to that of the mock-inoculated seedlings at 5 and 10 dpi, which was similar to the results of the time-dependent experiment. The *HvPAA1* transcript level in leaves of BSMV:HvPAA1-inoculated seedlings was decreased by 80.4%, 76.9%, and 58.7% when compared to mock-inoculated seedlings at 1, 5, and 10 dpi, respectively. The *HvPAA1* transcript level was significantly increased in leaves from BSMV:HvPAA1-inoculated seedlings under Cd stress as compared with BSMV:HvPAA1-inoculated seedlings at 1 and 10 dpi; at 5 dpi, no significant differences were found between them. 

As shown in Figure 6a,c, the mock-inoculated seedlings in control showed no significant differences when compared to that in Cd stress at each of the three sampling times. In the absence of Cd, the mock- and BSMV:HvPAA1-inoculated seedlings exhibited similar root dry weights at 1 dpi and similar shoot dry weights at 5 and 10 dpi. However, the presence of 5 µM Cd resulted in decreases in the shoot dry weight, especially at 10 dpi, in BSMV:HvPAA1-inoculated plants when compared with the BSMV:HvPAA1-inoculated plants. Nevertheless, no differences were found in RDW of BSMV:HvPAA1-inoculated seedlings under Cd and control conditions. 

As for Cd concentration in mock- and BSMV:HvPAA1-inoculated seedlings that were exposed to 5 µM Cd (Figure 6e,f), shoot Cd concentration showed no significant differences between the mock- and BSMV:HvPAA1-inoculated seedlings at 1 dpi, while root Cd concentration in BSMV:HvPAA1-inoculated seedlings was notably higher than in mock-inoculated seedlings. At 5 dpi, the silencing of *HvPAA1* significantly increased both the shoot and root Cd concentrations by 48.3% and 62.2%, respectively, as compared with the mock-inoculated seedlings. Meanwhile, BSMV:HvPAA1-inoculated seedlings accumulated more Cd than the mock-inoculated seedlings, which was in contrast to the result that was observed at 10 dpi (Figure 6g). This may have been due to the higher dry weight of mock-inoculated seedlings at 10 dpi. As shown in Figure 6h, the root-to-shoot transport ratio showed no significant difference between mock- and BSMV:HvPAA1-inoculated seedlings at 1, 5, and 10 dpi. These results proved that *HvPAA1* is closely associated with barley Cd tolerance and accumulation, but it has little effect on the root-to-shoot transport ratio.

## 3. Discussion

Cd is harmful to human health if it is incorporated into the food chain. The development of low Cd crops has become an urgent need and it relies heavily on knowledge regarding Cd uptake, accumulation, translocation, and exudation and excavating related genes. Such an understanding is vital in sustainable crop production, so as to alleviate health risks that are associated with exposure to Cd-contented food.

### 3.1. QTLs for Traits Investigated

In the present study, all of the detected 24 QTLs for the traits investigated were located on chromosome 1H, 2H, 3H, 5H, and 7H, respectively, which have a high density of markers. QTLs for SPAD value, root length, and leaf APX activity were only detected under control condition. As to QTLs for plant height, a total of 12 QTLs were detected, four of them were detected under the control condition located on chromosome 5H, the rest of them were detected under Cd stress, and they distributed on chromosome 5H and 7H. Furthermore, *qPH5H-2* under the control condition was overlapped with *qPH5H-2* under Cd stress, suggesting that this QTL region contains important genetic messages that are associated with plant height. This overlapped QTL was on chromosome 5H and it resolved in 2.81 cM, with the negative additive effect indicating Weisuobuzhi’s allele increased plant height. For leaf POD activity, a single QTL was detected under the control and Cd condition, respectively, but the location was differed from each other. However, *qRPOD5H*, which was associated with root POD activity under the control condition, have the same chromosome location but different position as *qLPOD5H* that was detected under control condition. Five QTLs that were associated with root Mn concentration under Cd stress were found on chromosome 2H, 3H, and 5H, which can explain 62% of the phenotypic variation in total. 

### 3.2. A major QTL qShCd7H was Detected for Cd Concentration in Shoot

Cadmium concentration could not be detected during our control experiment. A significantly larger phenotypic difference in terms of the shoot Cd concentration was observed in the parental and DH lines in the presence of Cd stress, which allowed for a single QTL, *qShCd7H*, to be identified. The *qShCd7H* QTL was associated with Cd concentration in the presence of 10 µM Cd stress, and the Suyinmai 2 allele was shown to increase the Cd concentration in shoots. During the phenotypic analysis, Suyinmai 2 exhibited a significantly higher shoot concentration than Weisuobuzhi, which suggested that the Cd concentration during seedling stage might be controlled by a major gene with dominant effects. The influence of a single QTL with dominant effects on Cd concentration in the presence of Cd stress has been well documented in other crops [3,4,7,10]. It is worth noting that, in the presence of low amounts (0.5 µM) of Cd stress, a marker-trait association with shoot Cd concentration was reported for all barley chromosomes [8]. The *qShCd7H* QTL was mostly likely the same QTL as the QTL that was anchored by marker 3140-491, and it was detected from a soil culture, which demonstrates that *qShCd7H* could be detected in different genetic backgrounds and test environments. This is very important for the marker-assisted selection of Cd accumulation during a short-term experiment. The GBS markers, TP18054 and TP11089, which were used to identify *qShCd7H*, could be converted into local laboratory-based PCR markers. 

### 3.3. A Novel Gene HvPAA1 is the Candidate Gene of qShCd7H for Cd Tolerance

A novel gene, *HvPAA1*, related to shoot Cd concentration, was identified from *qShCd7H*. The polygenetic analysis showed that *HvPAA1* was clustered into the *PAA1* group. The amino acid sequence of *HvPAA1* was mostly related to the *OsPAA1* (*Oryza sativa* L.). Sequence comparison between two parents demonstrated that they possess the same CDS sequence but have four SNPs in introns. Sequence analysis indicated that *HvPAA1* carried seven domains with an N-glycosylation motif, which is required for the regulation of enzymatic activity according to Migocka [18]. PAA1, also known as HMA6, is a Cu-ATPase that belongs to the heavy-metal ATPase (P_1B_-ATPases, HMAs) subfamily of cation-transporting P-ATPases. Both Cu- and Cd stress had a significant impact on the gene expression levels of *OsHMA6*, *ZmHMA6*, and *SbHMA6*, but the influence of Cd stress on gene expression appeared to be greater than that of Cu-induced stress [19]. The *Arabidopsis AtHMA6* is primarily involved in Cu transport [20,21], suggesting that the function of *HMA6* may be relevant to its sequence and the effects of external stress during different growth stages and/or in different plant organs. Other studies have attempted to reveal the relationship between Cd and Cu, and they have found that their relationship is dependent on the relevant genotypes, concentrations, and tissues [22,23,24]. The role of *PAA1* (*HMA6*) in Cd tolerance has not been reported in barley previously. Thus, we consider *HvPAA1* to be the candidate gene for *qShCd7H* with regard to Cd tolerance in barley. Further studies are needed to verify the relationship between *HvPAA1* and Cu transportation in the future. 

Regarding the physical position of *HvPAA1*, the EnsemblPlants and IPK database were used to investigate the position of *HvPAA1* in the barley physical map. The results indicated that this gene is located at 167 Mbp (million base pairs) on the chromosome 7H short arm approximately, while the whole chromosome size is about 680 Mbp. Mascher et al. [25] revealed a chromosome structure of the barley genome, in which has information regarding the position of centromere. According to their results, we find centromere on 7H is near to 350 Mbp. As a result, *HvPAA1* is far away from the centromere or telomere.

### 3.4. HvPAA1, Localized on Plasma Membrane, Contributes to Cd Tolerance and Accumulation in Barley

The tissue-specific expression pattern indicated that *HvPAA1* was mainly expressed in shoots in the absence of Cd, but that expression was induced by Cd in leaves and stems, which indicated that the expression of *HvPAA1* was upregulated by 5 µM Cd in shoots. This result was in agreement with those that were observed for the *OsHMA6* gene in rice, which showed that Cd stress significantly increased *OsHMA6* expression in shoots and stems, but not in roots [19]. The upregulation of *HvPAA1* peaked at 3 h and then dropped to the same level that was observed at 0 h at 3 and 7 dpi, which demonstrated that the Cd expression of *HvPAA1* was time-dependent and Cd only induced increases in the relative transcript level at 1 dpi. Our subcellular localization experiment showed that *HvPAA1* is located at the plasma membrane, similarly to *OsHMA6* in rice, *ZmHMA6* in maize, and *SbHMA6* in sorghum [19], but differently from *AtHMA6* in *Arabidopsis thaliana*, which is localized to the chloroplast. The differences in locations and functions of *PAA1* may be due to functional differences in different plant species. 

The characterization of gene function via overexpression or silencing in transgenic plants plays an important role in producing plants with novel traits, but this approach is time consuming [26]. Virus-induced gene silencing (VIGS) is a powerful functional genomics tool that can be used for rapid gene function analysis [27]. The expression of *HvPAA1* was significantly decreased after BSMV:HvPAA1-inoculation at 1, 5, and 10 dpi; a rise in the transcript level was detected at 10 dpi, although it was significantly lower than the increase that was observed in mock-inoculated plants. Cadmium stress increased *HvPAA1* expression level in BSMV:HvPAA1-inoculatd plants at 1 and 10 dpi. The silencing of *HvPAA1* resulted in an obvious decrease in the root and shoot dry weights at 10 dpi, which indicates that *HvPAA1* was responsible for Cd tolerance in Zhenong 8 (Figure 6). Meanwhile, the silencing of *HvPAA1* also resulted in increased Cd concentration in shoots/roots at 5 dpi. The results from the three sampling times differed from each other, which may be the result of the different expression level of *HvPAA1* at 1, 5, and 10 dpi. These results all indicated that *HvPAA1* is involved in Cd tolerance in barley.

## 4. Materials and Methods

### 4.1. Plant Materials and Experimental Design

A population consisting of 108 DH lines for QTL analysis was derived from a cross between the Suyinmai 2 (Cd-sensitive) and Weisuobuzhi (Cd-tolerant) [28] via a microspore culture. 

The hydroponic experiments were carried out at Zijingang Campus of Zhejiang University, Hangzhou, China, according to previously described methods [14,29]. At the second leaf stage (10 days old), uniform plants were selected and then transplanted into 35 L containers filled with 30 L of basal nutrient solution (BNS) [29]. The container was covered with a polystyrene plate with 64 evenly spaced holes (two plants per hole) and then placed in a net house. On the seventh day after transplantation, 10 µM Cd (CdCl_2_) was added to the appropriate containers (10 µM Cd), and BNS (control) was added to the remaining containers. The experiment was laid out with a split-plot design with Cd treatment as main plot and DH line as subplot; there were three replicates for each treatment. The nutrient solution was continuously aerated with pumps and renewed every four days. The plants were collected after 15 days of treatment.

### 4.2. Phenotyping of the DH population Lines and Parents

After 15 days of treatment, the SPAD values (chlorophyll meter readings) were determined in the top second fully expanded leaves using a chlorophyll meter (Minolta SPAD-502). Plants were collected and separated into roots and shoots after the plant height (PH) and root length (RL) were measured. Shoot dry weight (SDW) and root dry weight (RDW) was measured, after which the shoots and roots were powdered and ashed at 550 °C for 12 h. The ash was digested with 5 mL 30% HNO_3_ and then diluted with deionized water. Cd, Mn, and Zn concentrations in shoots (Sh_Cd_, Sh_Mn_, and Sh_Zn_) and roots (R_Cd_, R_Mn_, and R_Zn_) were determined using flame atomic absorption spectrometry (AA6300; Shimadzu, Tokyo, Japan).

### 4.3. Antioxidative Enzyme Activities

The fully expanded leaves and root samples were immediately frozen in liquid nitrogen and stored at −80 °C for the determination of guaiacol peroxidase (POD), catalase (CAT), and ascorbic acid oxidase (APX) activity in the shoots (Sh_POD_, Sh_CAT_, and Sh_APX_) and roots (R_POD_, R_CAT_, and R_APX_), according to Chen et al. [30] and Wu et al. [29]. 

Cd tolerance indexes (CTI) were calculated as a reduced (−)/increased (+) percentage of the control. i.e., CTI = [(parameter under stress − parameter under control)/parameter under control] × 100%]. 

### 4.4. Genotyping, Linkage Map Construction and QTL Mapping

Genomic DNA from each of the parental and DH lines was extracted from fresh leaves that were obtained from the seedling stage using the DNeasy Plant Mini Kit (QIAGEN, Hilden, Germany) according to the manufacturer’s protocol. The DH and parental lines were genotyped using sequencing technology (GBS). The GBS library was prepared using protocols that were described by Elshire et al. [31] and Poland et al. [32]. The DNA samples were digested using PstI and MspI for complexity reduction and they were then barcoded and multiplexed. Each GBS library, which contained 96 DNA samples (96-plex), was run on a single lane of an Illumina HiSeq2000 for sequencing. The GBS raw data were analyzed using the Universal Network Enabled Analysis Kit (UNEAK) pipeline in TASSEL [33]. Heterozygous markers and those that were missing more than 20% of their data were removed. Subsequent to this procedure, 1532 GBS markers were used for linkage map construction. The population was also genotyped using simple sequence repeat (SSR) markers, which were amplified using a polymerase chain reaction (PCR) method (Tables S2 and S3). The software package JoinMap 4.0 constructed the Weisuobuzhi × Suyinmai 2 linkage map [34]. The QTL intervals for all measured data were defined within the linkage map using MapQTL 5.0 software [35]. All data were expressed as the mean of three replicates that were conducted for the purposes of the QTL analysis. Interval mapping (IM) analysis was first performed to determine the potential regions of the QTLs, after which the markers that were close to the detected QTLs (identified by IM mapping) were selected as cofactors to be used to test the multiple QTL model (MQM) [36]. A logarithm of odds (LOD) threshold > 2.5 (α= 0.05) was the criterion that was used to confirm the presence of a QTL during the IM and MQM analysis. The detected QTLs were named according to the barley QTL nomenclature described by Szucs et al. [37]. 

### 4.5. Identification, Amplification, and Sequencing of Candidate Genes

Based on the QTL mapping study, a major QTL that was responsible for the Cd concentration in shoots was located, and the candidate genes that contribute to this QTL were identified. The positions of the flanking markers were aligned with the physical barley map using BLASTN tag sequences that were mapped against the sequence of the IBSC barley Morex cultivar in RefSeq v1.0 (http://webblast.ipk-gatersleben.de/registration/) and they are shown in Table S3. All of the genes within the QTL interval were extracted from the Barleymap database (http://floresta.eead.csic.es/barleymap). Based on gene annotation and literature studies, the AK355848.1 gene, which was annotated as “copper-transporting ATPase 1”, was compared against the National Center for Biotechnology Information (NCBI) nucleotide database while using BLAST. Very little information about AK355848.1 in barley was available based on a literature search. EnsemblPlants (http://plants.ensembl.org/index.html) and IPK (https://webblast.ipk-gatersleben.de/barley_ibsc/) database investigated the chromosome position of AK355848. The sequence of AK355848.1 was then cloned in the parents Suyinmai 2 and Weisuobuzhi and it was designated as the barley *HvPAA1*. Another Cd-tolerance cultivar Zhenong 8 was introduced to this study as a check variety with high-grain-Cd-accumulation [14]. Total RNA was extracted from Suyinmai 2, Weisuobuzhi, and Zhenong 8, according to the instructions that were included with the TaKaRa MiniBST Plant RNA Extraction Kit (Takara, Japan). Supplementary Table S1 shows the primer pairs. The PCR products were connected into the pMD18-T vector (Takara, Japan) and sequenced. The SMART (http://smart.embl-heidelberg.de/) and Protter (http://wlab.ethz.ch/protter/start/) databases were used to predict the amino acid sequence of the cloned gene. The amino acid composition, CDS, protein sequence, and structure of the cloned gene were compared with those of 14 other members of the heavy-metal ATPase (P_1B_-ATPases, HMAs) family. A phylogenetic tree was constructed in MEGA7 using the neighbor-joining algorithm analysis method that is based on the protein sequences of 28 ATPs. The full-length sequences of *HvPAA1* from two parents, including the exons and introns, were also analyzed. 

### 4.6. Expression Patterns, Tissue and Subcellular Localization of HvPAA1

Hydroponic experiments were repeated for quantitative real-time PCR (qRT-PCR) expression analysis. The Zhenong 8 seedlings were exposed to 5 μM Cd (CdCl_2_) for up to seven days to determine the time-dependent expression of *HvPAA1* in barley. Tissue-specific expression analysis of *HvPAA1* was conducted using roots, stems, and leaves from two-leaf-stage seedlings. Supplementary Table S1 shows the primer sequences that were used. 

To investigate subcellular localization, the coding regions of *HvPAA1* were directly amplified from the full length cDNA using primers 35SGFP-HvPAA1-F and 35SGFP-HvPAA1-R (Supplementary Table S1) and then connected into the pCAMBIA 1300 vector, which contains a CaMV 35S promoter:green fluorescent protein (35S:GFP) cassette, to create a HvPAA1-GFP fusion protein. The 35S:HvPAA1-GFP fusion construct was then introduced into the *Agrobacterium tumefaciens* GV3101 strain. *Nicotiana benthamiana* containing a red nuclear histone 2B marker (H2B) [17] was infected by the *Agrobacterium*-mediated system, as previously described [38]. 

### 4.7. BSMV Inoculation and Measurement of the Relative Transcript Level, Cd Tolerance and Metal Concentration

Total RNA was extracted from Zhenong 8. A PrimeScriptTM RT reagent kit (Takara, Japan) was used to synthesis the first-strand cDNA. A 286 bp cDNA fragment from the barley phytoene desaturase gene (*HvPDS*) and a 239 bp cDNA fragment from the *HvPAA1* gene containing *Nhe*I sites were amplified while using oligonucleotide primers (Table S1). These two fragments were inserted in reverse into the RNAγ cDNA strand to create two cDNA clones, BSMV:HvPDS, and BSMV:HvPAA1, to ensure further gene silencing in the Zhenong 8.

Inoculation was performed according to the protocol that was described by He et al. [27]. Seven days after inoculation, the transcript levels of *HvPDS* and *HvPAA1* were verified using qRT-PCR, after which the plants were treated with 5 μM CdCl_2_ for 10 d. Six replicates were used for each treatment.

The third leaves, which were collected at 1, 5, and 10 dpi, were used for the analysis of the transcript levels. Only plants that exhibited viral infection symptoms were chosen to serve as samples. The RNA extraction, cDNA synthesis, and qRT-PCR were performed, as described above.

The Cd tolerance of BSMV:HvPAA1 was evaluated. The roots and shoots of infected plants were separately harvested at 1, 5, and 10 dpi. SDW, RDW, Cd concentration/accumulation, and Cd root-to-shoot transport ratio were measured, as described above. All of the treatments used three replicates.

## 5. Conclusions

In conclusion, we developed a new mapping population that was based on the cross of Suyinmai 2 and Weisuobuzhi that utilizes 1532 GBS and 40 SSR markers. Seventeen traits were assessed in control and Cd stress conditions, and 24 QTLs were identified. A major QTL, *qShCd7H*, which is associated with Cd concentration, was mapped to chromosome 7H and it was found to explain 17% of phenotypic variation. The *qShCd7H* QTL was resolved to a 2.6 cM region between the GBS markers TP18054 and TP11089, which resulted in the cloning of a copper-transporting ATPase 1 (AK355848.1). We designated the cloned gene *HvPAA1*, which was found to be expressed mainly in shoots and localized to plasma membrane, and we observed that the silencing of *HvPAA1* leads to a decrease in biomass and increase in Cd concentration. All of this evidence confirmed that *HvPAA1* is a candidate gene for Cd concentration in *qShCd7H*. The results provide a molecular basis for understanding Cd accumulation in barley that will contribute to the development of molecular markers that can be used to quickly select low Cd accumulation varieties and to facilitate the cultivation of low-Cd barley varieties.

## Figures and Tables

**Table 1:** Phenotypes of the traits of growth and physiology and Cd tolerance indexes of the doubled haploid population from the cross between Suyinmai 2 and Weisuobuzhi.

	Parents			The Doubled Haploid Population (108 Lines)					
Traits	Suyinmai 2	Weisuobuzhi		Skewness	Kurtosis	Min	Max	Mean	CV (%)
***Under control condition***									
SDW	0.40	0.70 *		0.50	0.20	0.30	0.50	0.40	14.10
RDW	0.10	0.20 *		0.10	0.00	0.10	0.20	0.10	17.40
RL	22.90	24.80		0.60	2.50	18.50	31.10	22.60	8.50
PH	41.70	45.50		0.00	0.30	32.20	46.50	39.60	6.60
SPAD	32.00	30.30		0.80	4.20	27.60	33.60	29.80	4.20
L_POD_	9.85	8.15		0.98	0.86	2.64	19.91	9.17	35.74
L_APX_	3.62	24.43 *		3.64	21.79	5.04	63.30	15.07	48.86
L_CAT_	0.46	0.37		0.06	−1.01	0.10	0.94	0.47	47.82
R_POD_	32.59	25.56		2.16	10.95	17.90	89.45	36.37	24.44
R_APX_	15.90	8.27 *		0.88	1.58	3.91	29.28	14.60	15.85
R_CAT_	0.12	0.39 *		1.44	1.48	0.13	2.11	0.65	23.53
Sh_Mn_	30.49	44.27 *		−0.10	−0.97	20.11	56.19	37.07	24.66
Sh_Zn_	98.12	86.00 *		1.16	3.14	71.17	167.81	94.48	16.47
R_Mn_	134.14	167.45 *		0.79	1.48	90.82	245.09	135.32	21.50
R_Zn_	137.90	149.70		0.23	−0.12	84.99	180.93	143.31	12.81
***Under 10 µM Cd condition***									
SDW	0.20	0.40 *		0.60	1.10	0.20	0.40	0.30	15.30
RDW	0.10	0.10		0.20	0.20	0.00	0.10	0.10	16.30
RL	16.80	22.80 *		0.50	−0.20	16.10	23.40	19.20	8.80
PH	21.20	23.60		0.70	1.10	20.30	29.50	23.70	7.30
SPAD	27.10	29.00		−0.10	−0.50	22.80	30.10	26.70	6.20
L_POD_	35.81	15.03 *		1.09	1.45	8.49	48.99	21.07	39.08
L_APX_	12.79	47.77 *		0.25	1.62	6.01	65.02	20.68	60.48
L_CAT_	0.30	0.12		2.65	6.79	0.10	2.78	0.47	119.43
R_POD_	43.14	28.05 *		−0.35	0.047	14.35	67.61	42.89	16.35
R_APX_	21.40	6.36 *		0.72	0.99	4.23	46.49	18.68	17.20
R_CAT_	0.13	0.91 *		2.04	5.61	0.11	4.06	0.80	17.04
Sh_Cd_	199.60	172.20 *		0.10	−0.40	98.32	213.70	156.70	15.80
Sh_Mn_	30.80	45.90 *		0.50	1.70	24.20	65.10	42.10	16.20
Sh_Zn_	95.20	77.30 *		2.60	16.40	61.50	173.00	86.70	15.20
R_Cd_	1861.10	2235.00 *		0.10	−0.30	1161.00	2749.70	1921.50	18.20
R_Mn_	127.40	188.30 *		0.80	1.10	73.20	294.40	156.10	24.90
R_Zn_	158.80	169.00		0.60	2.30	76.30	248.20	157.90	15.70
***Cd tolerance index (CTI)***									
SDW	−36.3	−34.4		0.2	−0.7	−41.3	−0.2	−21.3	48.1
RDW	−41.5	−27.7		0.1	0.8	−43.7	−0.8	−21.9	47.8
RL	−26.5	−7.8		−0.1	0.0	−39.3	−29.0	−14.8	56.1
PH	−49.2	−48.1		0.5	−0.6	−48.9	−29.0	−40.1	10.4
SPAD	−15.5	−4.2		−0.2	0.7	−25.8	−0.4	−10.7	51.5
L_POD_	263.4	84.3		0.7	−0.2	3.2	391.3	135.3	66.7
L_APX_	253.9	95.6		4.7	27.9	39.1	341.8	167.6	79.2
L_CAT_	−35.6	−66.5		1.2	1.6	−99.0	38.5	−59.5	51.1
R_POD_	−42.5	9.8		1.8	3.5	2.6	187.9	42.5	87.4
R_APX_	34.6	268.3		1.6	2.6	3.0	436.3	97.4	97.7
R_CAT_	−50.4	16.5		0.4	−1.1	−99.8	−2.1	−57.0	52.3

Cd tolerance index (CTI) was calculated as CTI = (parameter under Cd stress − parameter under control)/parameter under the control) × 100%. * significant difference between the two parents Suyinmai 2 and Weisuobuzhi at the 0.05 level. SDW, shoot dry weight (g plant^−1^); RDW, root dry weight (g plant^−1^); RL, root length (cm); PH, plant height (cm); SPAD, SPAD value; L_POD_, leaf POD activity (OD470 g^−1^ FW min^−1^); L_APX_, leaf APX activity (mmol g^−1^ FW min^−1^); L_CAT_, leaf CAT activity (mmol g^−1^ FW min^−1^); R_POD_, root POD activity; R_APX_, root APX activity; R_CAT_, root CAT activity. Sh_Cd_, Sh_Mn_, Sh_Zn_, and R_Cd_, R_Mn_, R_Zn_ represent Cd, Mn, Zn concentrations (mg kg^−1^ DW) in shoots and roots, respectively. No Cd in plants was detected under control condition. CV (%), the coefficient of variation was presented as the absolute value of the ratio of the standard deviation to the mean value.

**Table 2:** Quantitative trait loci (QTLs) identified under control and 10 µM Cd condition.

Trait	QTL ^a^	Chr. ^b^	Position (cM) ^c^	Interval (cM) ^d^	Marker ^e^	LOD ^f^	R^2 g^ (%)	Add ^h^
	***Under control condition***							
SPAD value	*qSPAD3H*	3H	91.88	5.88	TP41927	2.82	11.30	0.42
Plant height (cm)	*qPH5H-1*	5H	27.20	3.90	Bmag323	2.83	11.40	−0.90
	*qPH5H-2*	5H	42.46	2.81	TP33838	3.26	13.00	−0.95
	*qPH5H-3*	5H	64.57	29.50	TP63873	4.31	16.80	−1.06
	*qPH5H-4*	5H	84.09	17.50	TP21896	3.77	14.90	−1.03
Root length (cm)	*qRL1H*	1H	161.32	1.29	TP13	2.87	11.50	−0.66
Leaf POD (OD470 g^−1^ FW min^−1^)	*qLPOD5H*	5H	233.32	3.91	TP52094	2.86	11.50	1.12
Root POD (OD470 g^−1^ FW min^−1^)	*qRPOD5H*	5H	84.09	4.00	TP21896	2.73	10.90	3.05
Leaf APX (mmol g^−1^ FW min^−1^)	*qLAPX1H*	1H	70.90	9.20	EBmac0501	2.59	10.40	−0.54
	***Under 10 µM Cd condition***							
Plant height (cm)	*qPH5H-1*	5H	37.24	4.24	TP20247	2.76	11.10	−0.58
	*qPH5H-2*	5H	42.46	2.81	TP33838	3.87	15.20	−0.68
	*qPH5H-3*	5H	43.76	5.97	TP220	3.55	14.00	−0.66
	*qPH5H-4*	5H	55.47	11.94	TP53164	3.55	14.10	−0.62
	*qPH5H-5*	5H	64.57	8.23	TP13622	3.76	14.80	−0.66
	*qPH5H-6*	5H	88.09	13.27	TP13479	3.80	15.00	−0.67
	*qPH7H-1*	7H	92.02	11.78	TP26119	3.00	12.00	−0.60
	*qPH7H-2*	7H	100.78	5.13	TP35770	2.81	11.50	−0.59
Leaf POD (OD470 g^−1^ FW min^−1^)	*qLPOD7H*	7H	22.50	8.80	Bmac0187	3.00	12.00	3.06
Root Mn (μg g^−1^ DW)	*qRMn2H-1*	2H	159.46	39.00	TP48811	3.19	12.70	−13.88
	*qRMn2H-2*	2H	168.58	2.60	TP10446	3.09	12.30	−13.65
	*qRMn3H*	3H	107.69	54.17	TP60843	3.46	13.70	−14.36
	*qRMn5H-1*	5H	15.90	1.29	HVM07	2.73	11.00	12.86
	*qRMn5H-2*	5H	94.60	5.11	TP27265	3.07	12.30	13.60
Shoot Cd (μg g^−1^ DW)	*qShCd7H*	7H	72.39	2.60	TP30771	4.28	17.00	10.67

^a^ QTLs are named by trait and chromosome; ^b^ The chromosome on which the QTL is mapped; ^c^ The position (in cM) of the QTL on the chromosome; ^d^ The genetic distance between two markers; ^e^ The marker at the maximum logarithm of odds (LOD) score for QTL; ^f^ The LOD score at the QTL; ^g^ The percentage of explained variance of the marker at QTL; ^h^ Additive effect, the estimated additive effect.

## Supplementary Materials

Supplementary materials can be found at https://www.mdpi.com/1422-0067/20/7/1732/s1.
