# Supplementary material for: HvPAA1 Encodes a P-Type ATPase, a Novel Gene for Cadmium Accumulation and Tolerance in Barley (Hordeum vulgare L.)
Source: Int J Mol Sci. 2019 Apr 8;20(7):1732. doi: 10.3390/ijms20071732 (PMC6480696; doi:10.3390/ijms20071732)
Supplement: Supplementary file 1 [file ijms-20-01732-s001.pdf]

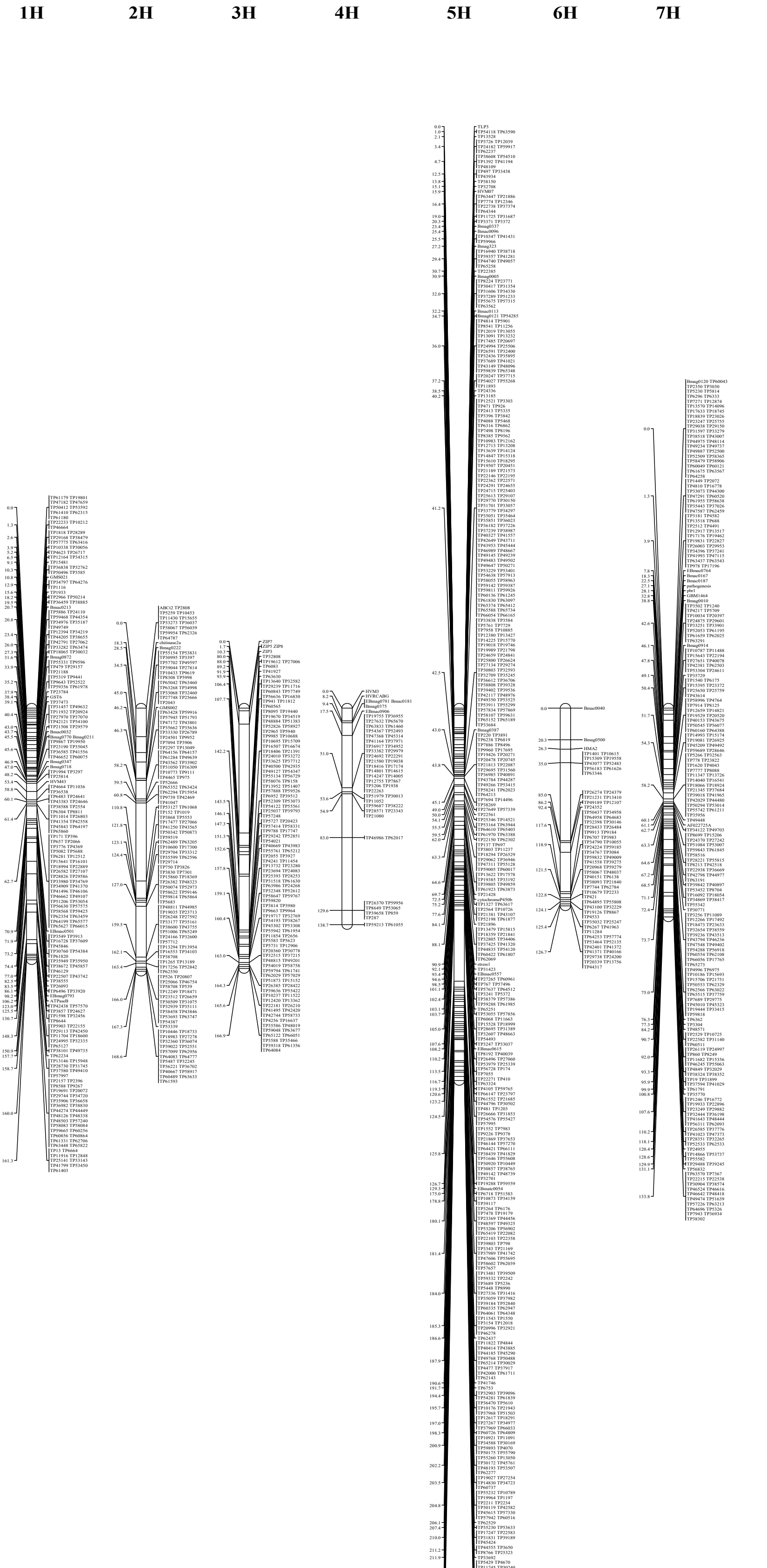

Figure S1. Genetic linkage groups of the Suyinmai 2 x Weisuobuzhi DH population. Marker distance in cM is on the left of the chromosome and marker name is on the right of the chromosome.

```
               10        20        30        40        50        60        70
1      ATGTCTTGTGGGGGATGTGCAGCAAGCGTTAAACGCATTTTGGAGAATGAGCCCCAGGTGGTGTCCGCAACTGTC
1       M  S  C  G  G  C  A  A  S  V  K  R  I  L  E  N  E  P  Q  V  V  S  A  T  V

               85        95       105       115       125       135       145
76     AATCTTGCCACCGAGATGGCAGTTGTGTGGGCTGTGCCAGAAGATAGAGCTGTACAAGATTGGAAACTGCAGTTG
26      N  L  A  T  E  M  A  V  V  W  A  V  P  E  D  R  A  V  Q  D  W  K  L  Q  L

              160       170       180       190       200       210       220
151    GGTGAGAAGCTCGCTAGTCAGTTGACAACATGTGGGTACAAATCCAGCCAGCGAGATTCTTCAAAAGTCAGTTCA
51      G  E  K  L  A  S  Q  L  T  T  C  G  Y  K  S  S  Q  R  D  S  S  K  V  S  S

              235       245       255       265       275       285       295
226    CAGAATGTTTTCGAAAGAAAGATGGGCGAAAAACTGCAAAATCTGAAGCAAAGTGGTCGAGAACTTGCTGTATCT
76      Q  N  V  F  E  R  K  M  G  E  K  L  Q  N  L  K  Q  S  G  R  E  L  A  V  S

              310       320       330       340       350       360       370
301    TGGGCACTATGTGCTGTTTGCCTACTGGGACATATTTCTCATCTCTTTGGAGTTAATGCACCATTGATGCACCTG
101     W  A  L  C  A  V  C  L  L  G  H  I  S  H  L  F  G  V  N  A  P  L  M  H  L

              385       395       405       415       425       435       445
376    TTTCATTCCACTGGATTCCATTTGTCTCTCTCAATATTTACATTTATTGGGCCTGGCCGAAGACTAATTATCGAT
126     F  H  S  T  G  F  H  L  S  L  S  I  F  T  F  I  G  P  G  R  R  L  I  I  D

              460       470       480       490       500       510       520
451    GGATTAAAGAGTCTATTCAAGGGTTCTCCAAACATGAATACATTGGTTGGTTTAGGTGCTCTGTCATCTTTTGCT
151     G  L  K  S  L  F  K  G  S  P  N  M  N  T  L  V  G  L  G  A  L  S  S  F  A

              535       545       555       565       575       585       595
526    GTCAGCTCAGTTGCAGCCTTCATTCCAAAACTGGGATGGAAGACATTTTTTGAGGAACCAATTATGTTGATAGCT
176     V  S  S  V  A  A  F  I  P  K  L  G  W  K  T  F  F  E  E  P  I  M  L  I  A

              610       620       630       640       650       660       670
601    TTTGTTCTTCTAGGGAAGAATCTTGAGCAGAGGGCGAAGCTAAAAGCTGCTAGTGATATGACCGGATTACTCAAT
201     F  V  L  L  G  K  N  L  E  Q  R  A  K  L  K  A  A  S  D  M  T  G  L  L  N

              685       695       705       715       725       735       745
676    ATACTTCCATCAAAAGCACGCCTAATGGTGGATAACGATGCTGAGCAATCATCATTCACAGAGGTCCCATGTGGT
226     I  L  P  S  K  A  R  L  M  V  D  N  D  A  E  Q  S  S  F  T  E  V  P  C  G

              760       770       780       790       800       810       820
751    ACTCTTGCTGTTGGGGACTATATATTAGTGCTGCCTGGGGACCGCATTCCAGCCGATGGACTTGTGAAAGCTGGA
251     T  L  A  V  G  D  Y  I  L  V  L  P  G  D  R  I  P  A  D  G  L  V  K  A  G

              835       845       855       865       875       885       895
826    AGAAGTACAGTTGACGAGTCAAGTTTGACAGGTGAACCTATGCCGGTAACTAAGATTGCAGGGGCAGAAGTATCA
276     R  S  T  V  D  E  S  S  L  T  G  E  P  M  P  V  T  K  I  A  G  A  E  V  S

              910       920       930       940       950       960       970
901    GCGGGGAGCATTAATTTAAACGGTAAACTGACAGTTGAAGTTCGACGACCTGGCGGTGAGACTGTCATGTCTGAC
301     A  G  S  I  N  L  N  G  K  L  T  V  E  V  R  R  P  G  G  E  T  V  M  S  D

              985       995      1005      1015      1025      1035      1045
976    ATACTTCACTTAGTGGAAGAAGCACAGACAAGGGAAGCCCCTGTTCAACGATTAGCTGACAAGGTTGCTGGGAAC
326     I  L  H  L  V  E  E  A  Q  T  R  E  A  P  V  Q  R  L  A  D  K  V  A  G  N

             1060      1070      1080      1090      1100      1110      1120
1051   TTTACATATGGTGTTATGGCGCTTTCTTCTGCTACCTTTATGTTCTGGAGTATTTTTGGTTCACAACTTGTACCT
351     F  T  Y  G  V  M  A  L  S  S  A  T  F  M  F  W  S  I  F  G  S  Q  L  V  P

             1135      1145      1155      1165      1175      1185      1195
1126   GCTGCTATCCAGCAGGGAAGTGCAATGTCTCTGGCTTTGCAGCTTTCTTGCAGTGTTCTGGTAATTGCTTGCCCA
376     A  A  I  Q  Q  G  S  A  M  S  L  A  L  Q  L  S  C  S  V  L  V  I  A  C  P

             1210      1220      1230      1240      1250      1260      1270
1201   TGTGCTCTTGGTCTTGCCACACCCACTGCAGTGCTGGTTGGTACTTCGTTAGGCGCAACGAGAGGACTTCTTTTA
401     C  A  L  G  L  A  T  P  T  A  V  L  V  G  T  S  L  G  A  T  R  G  L  L  L
```

```
              1285      1295      1305      1315      1325      1335      1345
1276     CGTGGTGGGGATGTTTTGGAGAAATTCGCGGAAGTTGATGCCATTGTGTTTGACAAGACCGGAACTTTAACAATT
426       R  G  G  D  V  L  E  K  F  A  E  V  D  A  I  V  F  D  K  T  G  T  L  T  I

              1360      1370      1380      1390      1400      1410      1420
1351     GGGAAGCCTGTAGTGACAAAAGTAATAGCTTCTCACAGCGAGGGAGGTGTAAATACAAAAGATTACAGGAACAAT
451       G  K  P  V  V  T  K  V  I  A  S  H  S  E  G  G  V  N  T  K  D  Y  R  N  N

              1435      1445      1455      1465      1475      1485      1495
1426     GAATGGACAGAAGGTGACGTTCTTAGTTTGGCTGCCGGAGTAGAATCAAATACAAACCACCCACTTGGAAAAGCC
476       E  W  T  E  G  D  V  L  S  L  A  A  G  V  E  S  N  T  N  H  P  L  G  K  A

              1510      1520      1530      1540      1550      1560      1570
1501     ATCATGGAAGCTGCCCAGGCTGCCAACTGCATCAATATGAAGGCAAAGGATGGGTCCTTTATGGAAGAACCAGGG
501       I  M  E  A  A  Q  A  A  N  C  I  N  M  K  A  K  D  G  S  F  M  E  E  P  G

              1585      1595      1605      1615      1625      1635      1645
1576     TCTGGTGCTGTGGCTACGATTGGTGAAAAACAGGTTTCGGTTGGGACATTAGACTGGATTAGGAGGCATGGTGTT
526       S  G  A  V  A  T  I  G  E  K  Q  V  S  V  G  T  L  D  W  I  R  R  H  G  V

              1660      1670      1680      1690      1700      1710      1720
1651     GTTCGTGAACCATTTCCTGAAGCAGAAAATTTTGGTCAGTCTGTTGCATATGTAGCAGTTGACGGTACTCTAGCT
551       V  R  E  P  F  P  E  A  E  N  F  G  Q  S  V  A  Y  V  A  V  D  G  T  L  A

              1735      1745      1755      1765      1775      1785      1795
1726     GGTCTTATTTGTTTCGAGGATAAGATCAGAGAAGATTCTCATCAAGTTATCAATGCCCTGTCTAAGCAAGGAATT
576       G  L  I  C  F  E  D  K  I  R  E  D  S  H  Q  V  I  N  A  L  S  K  Q  G  I

              1810      1820      1830      1840      1850      1860      1870
1801     AGTGTGTATATGTTATCTGGGGACAAGGAGAGTGCTGCTATGAATGTTGCCTCAATTGTTGGCATTCAGTTAGAC
601       S  V  Y  M  L  S  G  D  K  E  S  A  A  M  N  V  A  S  I  V  G  I  Q  L  D

              1885      1895      1905      1915      1925      1935      1945
1876     AAGGTGATTTCTGAAGTTAAACCACACGAGAAAAAGAAGTTCATATCTGAACTTCAAAAGGAGCACAAATTAGTT
626       K  V  I  S  E  V  K  P  H  E  K  K  K  F  I  S  E  L  Q  K  E  H  K  L  V

              1960      1970      1980      1990      2000      2010      2020
1951     GCCATGGTTGGTGATGGCATTAATGATGCTGCAGCCCTAGCTTTAGCTGACGTTGGAATTGCAATGGGTGGAGGT
651       A  M  V  G  D  G  I  N  D  A  A  A  L  A  L  A  D  V  G  I  A  M  G  G  G

              2035      2045      2055      2065      2075      2085      2095
2026     GTTGGTGCAGCTAGTGACGTATCTTCAGTTGTTCTCATGGGTAATAGGTTATCTCAGCTTGTTGATGCTTTAGAG
676       V  G  A  A  S  D  V  S  S  V  V  L  M  G  N  R  L  S  Q  L  V  D  A  L  E

              2110      2120      2130      2140      2150      2160      2170
2101     TTAAGTAAAGAAACCATGAGAACAGTGAAGCAAAATCTTTGGTGGGCTTTTCTGTATAACATTGTTGGCTACCC
701       L  S  K  E  T  M  R  T  V  K  Q  N  L  W  W  A  F  L  Y  N  I  V  G  L  P

              2185      2195      2205      2215      2225      2235      2245
2176     GTTGCTGCTGGAGCATTGCTGCCAGTGACGGGTACGATGCTGACCCCGTCGATAGCTGGAGCACTCATGGGTTTT
726       V  A  A  G  A  L  L  P  V  T  G  T  M  L  T  P  S  I  A  G  A  L  M  G  F

              2260      2270      2280      2290      2300      2310      2320
2251     AGTTCAGTCAGCGTGATGGCCAATTCCTTGCTTTTGAGGGCGAGGATGAGTTCAAAGCATCATGTTCAGAGCAGA
751       S  S  V  S  V  M  A  N  S  L  L  L  R  A  R  M  S  S  K  H  H  V  Q  S  R

              2335      2345      2355      2365      2375      2385      2395
2326     CAAAAGCCTCACAACACTATTTCTGATGTGTCAGACGGGGCTGGTGAGGTAGAGCAAAGTTATCCATCAAAATGG
776       Q  K  P  H  N  T  I  S  D  V  S  D  G  A  G  E  V  E  Q  S  Y  P  S  K  W

              2410
2401     AGGAGTACCTGA
801       R  S  T  *
```

Figure S2. The nucleotide and amino acid sequences of *HvPAA1*

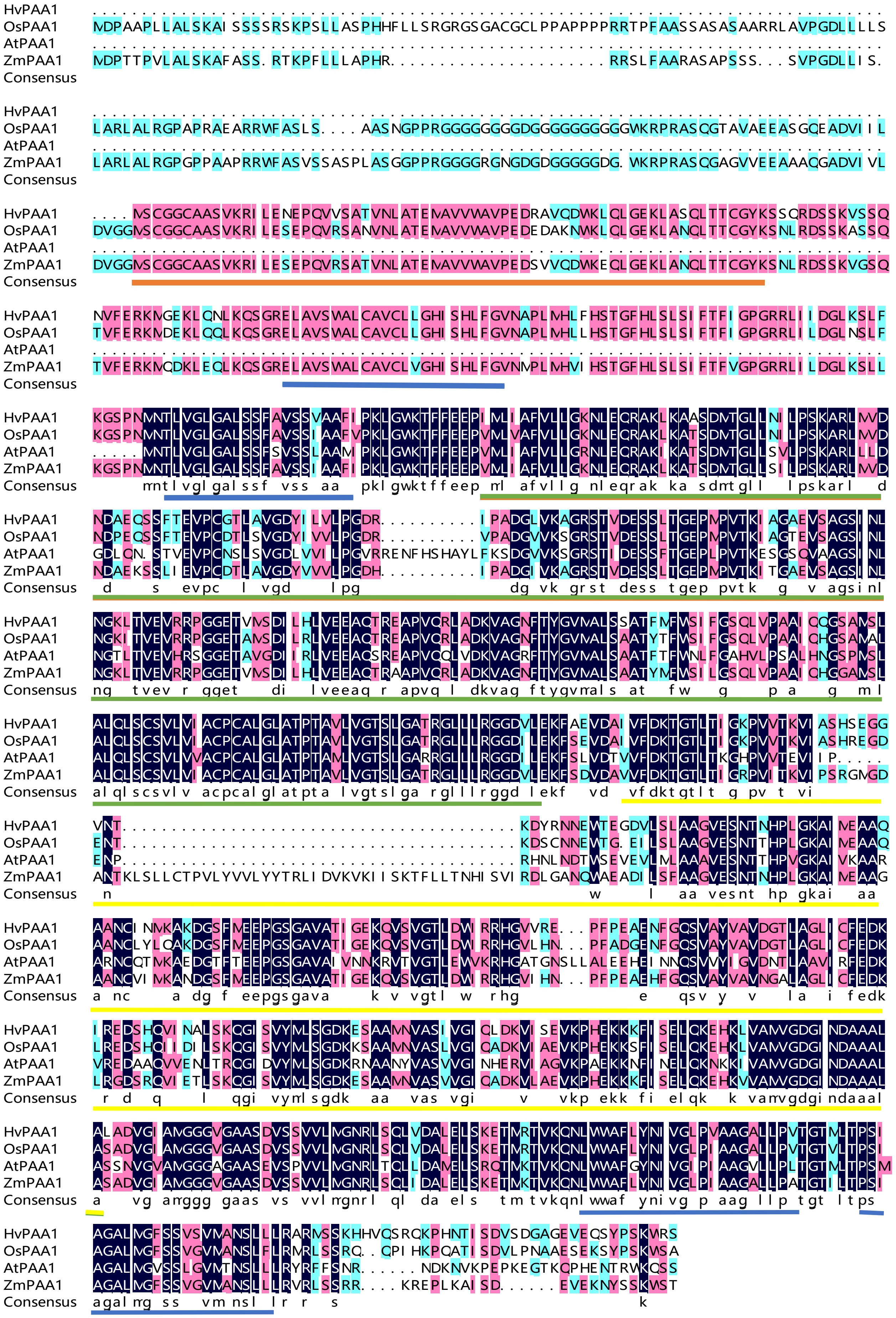

Figure S3. Alignment of the *HvPAA1* amino acid sequence with that of three other plant species: *OsPAA1* rice (*Oryza sativa*), *ZmPAA1* maize (*Zea mays* L.) and *AtPAA1* Arabidopsis (*Arabidopsis thaliana*). Lines in dark blue, pink and light green represent 100%, 75% and 50% identity, as defined by ClustalX. The red line indicates HMA domain, the blue line point out four transmembrane regions, the green line point out the E1-E2_ATPase domain, the yellow line point out HAD domain.

```
suyinmai2     ATGTCTTGTGGGGGATGTGCAGCAAGCGTTAAACGCATTTTGGAGAATGAGCCCCAGGTGGTGTCCGCAACTGTCAATCTTGCCACCGAGATGGCAGTTGTGTGGGCTGTGCCAGAAGAT   120
weisuobuzhi   ATGTCTTGTGGGGGATGTGCAGCAAGCGTTAAACGCATTTTGGAGAATGAGCCCCAGGTGGTGTCCGCAACTGTCAATCTTGCCACCGAGATGGCAGTTGTGTGGGCTGTGCCAGAAGAT   120
zhenong8      ATGTCTTGTGGGGGATGTGCAGCAAGCGTTAAACGCATTTTGGAGAATGAGCCCCAGGTGGTGTCCGCAACTGTCAATCTTGCCACCGAGATGGCAGTTGTGTGGGCTGTGCCAGAAGAT   120
Consensus     atgtcttgtgggggatgtgcagcaagcgttaaacgcattttggagaatgagccccaggtggtgtccgcaactgtcaatcttgccaccgagatggcagttgtgtgggctgtgccagaagat

suyinmai2     AGAGCTGTACAAGATTGGAAACTGCAGTTGGGTGAGAAGCTCGCTAGTCAGTTGACAACATGTGGGTACAAATCCAGCCAGCGAGATTCTTCAAAAGTCAGTTCACAGAATGTTTTCGAA   240
weisuobuzhi   AGAGCTGTACAAGATTGGAAACTGCAGTTGGGTGAGAAGCTCGCTAGTCAGTTGACAACATGTGGGTACAAATCCAGCCAGCGAGATTCTTCAAAAGTCAGTTCACAGAATGTTTTCGAA   240
zhenong8      AGAGCTGTACAAGATTGGAAACTGCAGTTGGGTGAGAAGCTCGCTAGTCAGTTGACAACATGTGGGTACAAATCCAGCCAGCGAGATTCTTCAAAAGTCAGTTCACAGAATGTTTTCGAA   240
Consensus     agagctgtacaagattggaaactgcagttgggtgagaagctcgctagtcagttgacaacatgtgggtacaaatccagccagcgagattcttcaaaagtcagttcacagaatgttttcgaa

suyinmai2     AGAAAGATGGGCGAAAAACTGCAAAATCTGAAGCAAAGTGGTCGAGAACTTGCTGTATCTTGGGCACTATGTGCTGTTTGCCTACTGGGACATATTTCTCATCTCTTTGGAGTTAATGCA   360
weisuobuzhi   AGAAAGATGGGCGAAAAACTGCAAAATCTGAAGCAAAGTGGTCGAGAACTTGCTGTATCTTGGGCACTATGTGCTGTTTGCCTACTGGGACATATTTCTCATCTCTTTGGAGTTAATGCA   360
zhenong8      AGAAAGATGGGCGAAAAACTGCAAAATCTGAAGCAAAGTGGTCGAGAACTTGCTGTATCTTGGGCACTATGTGCTGTTTGCCTACTGGGACATATTTCTCATCTCTTTGGAGTTAATGCA   360
Consensus     agaaagatgggcgaaaaactgcaaaatctgaagcaaagtggtcgagaacttgctgtatcttgggcactatgtgctgtttgcctactgggacatatttctcatctctttggagttaatgca

suyinmai2     CCATTGATGCACCTGTTTCATTCCACTGGATTCCATTTGTCTCTCTCAATATTTACATTTATTGGGCCTGGCCGAAGACTAATTATCGATGGATTAAAGAGTCTATTCAAGGGTTCTCCA   480
weisuobuzhi   CCATTGATGCACCTGTTTCATTCCACTGGATTCCATTTGTCTCTCTCAATATTTACATTTATTGGGCCTGGCCGAAGACTAATTATCGATGGATTAAAGAGTCTATTCAAGGGTTCTCCA   480
zhenong8      CCATTGATGCACCTGTTTCATTCCACTGGATTCCATTTGTCTCTCTCAATATTTACATTTATTGGGCCTGGCCGAAGACTAATTATCGATGGATTAAAGAGTCTATTCAAGGGTTCTCCA   480
Consensus     ccattgatgcacctgtttcattccactggattccatttgtctctctcaatatttacatttattgggcctggccgaagactaattatcgatggattaaagagtctattcaagggttctcca

suyinmai2     AACATGAATACATTGGTTGGTTTAGGTGCTCTGTCATCTTTTGCTGTCAGCTCAGTTGCAGCCTTCATTCCAAAACTGGGATGGAAGACATTTTTTGAGGAACCAATTATGTTGATAGCT   600
weisuobuzhi   AACATGAATACATTGGTTGGTTTAGGTGCTCTGTCATCTTTTGCTGTCAGCTCAGTTGCAGCCTTCATTCCAAAACTGGGATGGAAGACATTTTTTGAGGAACCAATTATGTTGATAGCT   600
zhenong8      AACATGAATACATTGGTTGGTTTAGGTGCTCTGTCATCTTTTGCTGTCAGCTCAGTTGCAGCCTTCATTCCAAAACTGGGATGGAAGACATTTTTTGAGGAACCAATTATGTTGATAGCT   600
Consensus     aacatgaatacattggttggtttaggtgctctgtcatcttttgctgtcagctcagttgcagccttcattccaaaactgggatggaagacattttttgaggaaccaattatgttgatagct

suyinmai2     TTTGTTCTTCTAGGGAAGAATCTTGAGCAGAGGGCGAAGCTAAAAGCTGCTAGTGATATGACCGGATTACTCAATATACTTCCATCAAAAGCACGCCTAATGGTGGATAATGATGCTGAG   720
weisuobuzhi   TTTGTTCTTCTAGGGAAGAATCTTGAGCAGAGGGCGAAGCTAAAAGCTGCTAGTGATATGACCGGATTACTCAATATACTTCCATCAAAAGCACGCCTAATGGTGGATAATGATGCTGAG   720
zhenong8      TTTGTTCTTCTAGGGAAGAATCTTGAGCAGAGGGCGAAGCTAAAAGCTGCTAGTGATATGACCGGATTACTCAATATACTTCCATCAAAAGCACGCCTAATGGTGGATAATGATGCTGAG   720
Consensus     tttgttcttctagggaagaatcttgagcagagggcgaagctaaaagctgctagtgatatgaccggattactcaatatacttccatcaaaagcacgcctaatggtggataatgatgctgag

suyinmai2     CAATCATCATTCACAGAGGTCCCATGTGGTACTCTTGCTGTTGGGGACTATATATTAGTGCTGCCTGGGGACCGCATTCCAGCCGATGGACTTGTGAAAGCTGGAAGAAGTACAGTTGAC   840
weisuobuzhi   CAATCATCATTCACAGAGGTCCCATGTGGTACTCTTGCTGTTGGGGACTATATATTAGTGCTGCCTGGGGACCGCATTCCAGCCGATGGACTTGTGAAAGCTGGAAGAAGTACAGTTGAC   840
zhenong8      CAATCATCATTCACAGAGGTCCCATGTGGTACTCTTGCTGTTGGGGACTATATATTAGTGCTGCCTGGGGACCGCATTCCAGCCGATGGACTTGTGAAAGCTGGAAGAAGTACAGTTGAC   840
Consensus     caatcatcattcacagaggtcccatgtggtactcttgctgttggggactatatattagtgctgcctggggaccgcattccagccgatggacttgtgaaagctggaagaagtacagttgac

suyinmai2     GAGTCAAGTTTGACAGGTGAACCTATGCCGGTAACTAAGATTGCAGGGGCAGAAGTATCAGCGGGGAGCATTAATTTAAACGGTAAACTGACAGTTGAAGTTCGACGACCTGGCGGTGAG   960
weisuobuzhi   GAGTCAAGTTTGACAGGTGAACCTATGCCGGTAACTAAGATTGCAGGGGCAGAAGTATCAGCGGGGAGCATTAATTTAAACGGTAAACTGACAGTTGAAGTTCGACGACCTGGCGGTGAG   960
zhenong8      GAGTCAAGTTTGACAGGTGAACCTATGCCGGTAACTAAGATTGCAGGGGCAGAAGTATCAGCGGGGAGCATTAATTTAAACGGTAAACTGACAGTTGAAGTTCGACGACCTGGCGGTGAG   960
Consensus     gagtcaagtttgacaggtgaacctatgccggtaactaagattgcaggggcagaagtatcagcggggagcattaatttaaacggtaaactgacagttgaagttcgacgacctggcggtgag

suyinmai2     ACTGTCATGTCTGACATACTTCACTTAGTGGAAGAAGCACAGACAAGGGAAGCCCCTGTTCAACGATTAGCTGACAAGGTTGCTGGGAACTTTACATATGGTGTTATGGCGCTTTCTTCT   1080
weisuobuzhi   ACTGTCATGTCTGACATACTTCACTTAGTGGAAGAAGCACAGACAAGGGAAGCCCCTGTTCAACGATTAGCTGACAAGGTTGCTGGGAACTTTACATATGGTGTTATGGCGCTTTCTTCT   1080
zhenong8      ACTGTCATGTCTGACATACTTCACTTAGTGGAAGAAGCACAGACAAGGGAAGCCCCTGTTCAACGATTAGCTGACAAGGTTGCTGGGAACTTTACATATGGTGTTATGGCGCTTTCTTCT   1080
Consensus     actgtcatgtctgacatacttcacttagtggaagaagcacagacaagggaagcccctgttcaacgattagctgacaaggttgctgggaactttacatatggtgttatggcgctttcttct

suyinmai2     GCTACCTTTATGTTCTGGAGTATTTTTGGTTCACAACTTGTACCTGCTGCTATCCAGCAGGGAAGTGCAATGTCTCTGGCTTTGCAGCTTTCTTGCAGTGTTCTGGTAATTGCTTGCCCA   1200
weisuobuzhi   GCTACCTTTATGTTCTGGAGTATTTTTGGTTCACAACTTGTACCTGCTGCTATCCAGCAGGGAAGTGCAATGTCTCTGGCTTTGCAGCTTTCTTGCAGTGTTCTGGTAATTGCTTGCCCA   1200
zhenong8      GCTACCTTTATGTTCTGGAGTATTTTTGGTTCACAACTTGTACCTGCTGCTATCCAGCAGGGAAGTGCAATGTCTCTGGCTTTGCAGCTTTCTTGCAGTGTTCTGGTAATTGCTTGCCCA   1200
Consensus     gctacctttatgttctggagtatttttggttcacaacttgtacctgctgctatccagcagggaagtgcaatgtctctggctttgcagctttcttgcagtgttctggtaattgcttgccca

suyinmai2     TGTGCTCTTGGTCTTGCCACACCCACTGCAGTGCTGGTTGGTACTTCGTTAGGTGCAACGAGAGGACTTCTTTTACGTGGTGGGGATGTTTTGGAGAAATTCGCGGAAGTTGATGCCATT   1320
weisuobuzhi   TGTGCTCTTGGTCTTGCCACACCCACTGCAGTGCTGGTTGGTACTTCGTTAGGTGCAACGAGAGGACTTCTTTTACGTGGTGGGGATGTTTTGGAGAAATTCGCGGAAGTTGATGCCATT   1320
zhenong8      TGTGCTCTTGGTCTTGCCACACCCACTGCAGTGCTGGTTGGTACTTCGTTAGGTGCAACGAGAGGACTTCTTTTACGTGGTGGGGATGTTTTGGAGAAATTCGCGGAAGTTGATGCCATT   1320
Consensus     tgtgctcttggtcttgccacacccactgcagtgctggttggtacttcgttaggtgcaacgagaggacttcttttacgtggtggggatgttttggagaaattcgcggaagttgatgccatt

suyinmai2     GTGTTTGACAAGACCGGAACTTTAACAATTGGGAAGCCTGTAGTGACAAAAGTAATAGCTTCTCACAGCGAGGGAGGTGTAAATACAAAAGATTACAGGAACAATGAATGGACAGAAGGT   1440
weisuobuzhi   GTGTTTGACAAGACCGGAACTTTAACAATTGGGAAGCCTGTAGTGACAAAAGTAATAGCTTCTCACAGCGAGGGAGGTGTAAATACAAAAGATTACAGGAACAATGAATGGACAGAAGGT   1440
zhenong8      GTGTTTGACAAGACCGGAACTTTAACAATTGGGAAGCCTGTAGTGACAAAAGTAATAGCTTCTCACAGCGAGGGAGGTGTAAATACAAAAGATTACAGGAACAATGAATGGACAGAAGGT   1440
Consensus     gtgtttgacaagaccggaactttaacaattgggaagcctgtagtgacaaaagtaatagcttctcacagcgagggaggtgtaaatacaaaagattacaggaacaatgaatggacagaaggt

suyinmai2     GACGTTCTTAGTTTGGCTGCCGGAGTAGAATCAAATACAAACCACCCACTTGGAAAAGCCATCATGGAAGCTGCCCAGGCTGCCAACTGCATCAATATGAAGGCAAAGGATGGGTCCTTT   1560
weisuobuzhi   GACGTTCTTAGTTTGGCTGCCGGAGTAGAATCAAATACAAACCACCCACTTGGAAAAGCCATCATGGAAGCTGCCCAGGCTGCCAACTGCATCAATATGAAGGCAAAGGATGGGTCCTTT   1560
zhenong8      GACGTTCTTAGTTTGGCTGCCGGAGTAGAATCAAATACAAACCACCCACTTGGAAAAGCCATCATGGAAGCTGCCCAGGCTGCCAACTGCATCAATATGAAGGCAAAGGATGGGTCCTTT   1560
Consensus     gacgttcttagtttggctgccggagtagaatcaaatacaaaccacccacttggaaaagccatcatggaagctgcccaggctgccaactgcatcaatatgaaggcaaaggatgggtccttt

suyinmai2     ATGGAAGAACCAGGGTCTGGTGCTGTGGCTACGATTGGTGAAAAACAGGTTTCGGTTGGGACATTAGACTGGATTAGGAGGCATGGTGTTGTTCGTGAACCATTTCCTGAAGCAGAAAAT   1680
weisuobuzhi   ATGGAAGAACCAGGGTCTGGTGCTGTGGCTACGATTGGTGAAAAACAGGTTTCGGTTGGGACATTAGACTGGATTAGGAGGCATGGTGTTGTTCGTGAACCATTTCCTGAAGCAGAAAAT   1680
zhenong8      ATGGAAGAACCAGGGTCTGGTGCTGTGGCTACGATTGGTGAAAAACAGGTTTCGGTTGGGACATTAGACTGGATTAGGAGGCATGGTGTTGTTCGTGAACCATTTCCTGAAGCAGAAAAT   1680
Consensus     atggaagaaccagggtctggtgctgtggctacgattggtgaaaaacaggtttcggttgggacattagactggattaggaggcatggtgttgttcgtgaaccatttcctgaagcagaaaat

suyinmai2     TTTGGTCAGTCTGTTGCATATGTAGCAGTTGACGGTACTCTAGCTGGTCTTATTTGTTTCGAGGATAAGATCAGAGAAGATTCTCATCAAGTTATCAATGCCCTGTCTAAGCAAGGAATT   1800
weisuobuzhi   TTTGGTCAGTCTGTTGCATATGTAGCAGTTGACGGTACTCTAGCTGGTCTTATTTGTTTCGAGGATAAGATCAGAGAAGATTCTCATCAAGTTATCAATGCCCTGTCTAAGCAAGGAATT   1800
zhenong8      TTTGGTCAGTCTGTTGCATATGTAGCAGTTGACGGTACTCTAGCTGGTCTTATTTGTTTCGAGGATAAGATCAGAGAAGATTCTCATCAAGTTATCAATGCCCTGTCTAAGCAAGGAATT   1800
Consensus     tttggtcagtctgttgcatatgtagcagttgacggtactctagctggtcttatttgtttcgaggataagatcagagaagattctcatcaagttatcaatgccctgtctaagcaaggaatt

suyinmai2     AGTGTGTATATGTTATCTGGGGACAAGGAGAGTGCTGCTATGAATGTTGCCTCAATTGTTGGCATTCAGTTAGACAAGGTGATTTCTGAAGTTAAACCACACGAGAAAAAGAAGTTCATA   1920
weisuobuzhi   AGTGTGTATATGTTATCTGGGGACAAGGAGAGTGCTGCTATGAATGTTGCCTCAATTGTTGGCATTCAGTTAGACAAGGTGATTTCTGAAGTTAAACCACACGAGAAAAAGAAGTTCATA   1920
zhenong8      AGTGTGTATATGTTATCTGGGGACAAGGAGAGTGCTGCTATGAATGTTGCCTCAATTGTTGGCATTCAGTTAGACAAGGTGATTTCTGAAGTTAAACCACACGAGAAAAAGAAGTTCATA   1920
Consensus     agtgtgtatatgttatctggggacaaggagagtgctgctatgaatgttgcctcaattgttggcattcagttagacaaggtgatttctgaagttaaaccacacgagaaaaagaagttcata

suyinmai2     TCTGAACTTCAAAAGGAGCACAAATTAGTTGCCATGGTTGGTGATGGCATTAATGATGCTGCAGCCCTAGCTTTAGCTGACGTTGGAATTGCAATGGGTGGAGGTGTTGGTGCAGCTAGT   2040
weisuobuzhi   TCTGAACTTCAAAAGGAGCACAAATTAGTTGCCATGGTTGGTGATGGCATTAATGATGCTGCAGCCCTAGCTTTAGCTGACGTTGGAATTGCAATGGGTGGAGGTGTTGGTGCAGCTAGT   2040
zhenong8      TCTGAACTTCAAAAGGAGCACAAATTAGTTGCCATGGTTGGTGATGGCATTAATGATGCTGCAGCCCTAGCTTTAGCTGACGTTGGAATTGCAATGGGTGGAGGTGTTGGTGCAGCTAGT   2040
Consensus     tctgaacttcaaaaggagcacaaattagttgccatggttggtgatggcattaatgatgctgcagccctagctttagctgacgttggaattgcaatgggtggaggtgttggtgcagctagt

suyinmai2     GACGTATCTTCAGTTGTTCTCATGGGTAATAGGTTATCTCAGCTTGTTGATGCTTTAGAGTTAAGTAAAGAAACCATGAGAACAGTGAAGCAAAATCTTTGGTGGGCTTTTCTGTATAAC   2160
weisuobuzhi   GACGTATCTTCAGTTGTTCTCATGGGTAATAGGTTATCTCAGCTTGTTGATGCTTTAGAGTTAAGTAAAGAAACCATGAGAACAGTGAAGCAAAATCTTTGGTGGGCTTTTCTGTATAAC   2160
zhenong8      GACGTATCTTCAGTTGTTCTCATGGGTAATAGGTTATCTCAGCTTGTTGATGCTTTAGAGTTAAGTAAAGAAACCATGAGAACAGTGAAGCAAAATCTTTGGTGGGCTTTTCTGTATAAC   2160
Consensus     gacgtatcttcagttgttctcatgggtaataggttatctcagcttgttgatgctttagagttaagtaaagaaaccatgagaacagtgaagcaaaatctttggtgggcttttctgtataac

suyinmai2     ATTGTTGGGCTACCCGTTGCTGCTGGAGCATTGCTGCCAGTGACGGGTACGATGCTGACCCCGTCGATAGCTGGAGCACTCATGGGTTTTAGTTCAGTCAGCGTGATGGCCAATTCCTTG   2280
weisuobuzhi   ATTGTTGGGCTACCCGTTGCTGCTGGAGCATTGCTGCCAGTGACGGGTACGATGCTGACCCCGTCGATAGCTGGAGCACTCATGGGTTTTAGTTCAGTCAGCGTGATGGCCAATTCCTTG   2280
zhenong8      ATTGTTGGGCTACCCGTTGCTGCTGGAGCATTGCTGCCAGTGACGGGTACGATGCTGACCCCGTCGATAGCTGGAGCACTCATGGGTTTTAGTTCAGTCAGCGTGATGGCCAATTCCTTG   2280
Consensus     attgttgggctacccgttgctgctggagcattgctgccagtgacgggtacgatgctgaccccgtcgatagctggagcactcatgggttttagttcagtcagcgtgatggccaattccttg

suyinmai2     CTTTTGAGGGCGAGGATGAGTTCAAAGCATCATGTTCAGAGCAGACAAAAGCCTCACAACACTATTTCTGATGTGTCAGACGGGGCTGGTGAGGTAGAGCAAAGTTATCCATCAAAATGG   2400
weisuobuzhi   CTTTTGAGGGCGAGGATGAGTTCAAAGCATCATGTTCAGAGCAGACAAAAGCCTCACAACACTATTTCTGATGTGTCAGACGGGGCTGGTGAGGTAGAGCAAAGTTATCCATCAAAATGG   2400
zhenong8      CTTTTGAGGGCGAGGATGAGTTCAAAGCATCATGTTCAGAGCAGACAAAAGCCTCACAACACTATTTCTGATGTGTCAGACGGGGCTGGTGAGGTAGAGCAAAGTTATCCATCAAAATGG   2400
Consensus     cttttgagggcgaggatgagttcaaagcatcatgttcagagcagacaaaagcctcacaacactatttctgatgtgtcagacggggctggtgaggtagagcaaagttatccatcaaaatgg

suyinmai2     AGGAGTACCTG                                                                                                                2411
weisuobuzhi   AGGAGTACCTG                                                                                                                2411
zhenong8      AGGAGTACCTG                                                                                                                2411
Consensus     aggagtacctg
```

```
suyinmai2     MSCGGCAASVKRILENEPQVVSATVNLATEMAVVWAVPEDRAVQDWKLQLGEKLASQLTTCGYKSSQRDSSKVSSQNVFE    80
weisuobuzhi   MSCGGCAASVKRILENEPQVVSATVNLATEMAVVWAVPEDRAVQDWKLQLGEKLASQLTTCGYKSSQRDSSKVSSQNVFE    80
zhenong8      MSCGGCAASVKRILENEPQVVSATVNLATEMAVVWAVPEDRAVQDWKLQLGEKLASQLTTCGYKSSQRDSSKVSSQNVFE    80
Consensus     mscggcaasvkrilenepqvvsatvnlatemavvwavpedravqdwklqlgeklasqlttcgykssqrdsskvssqnvfe

suyinmai2     RKMGEKLQNLKQSGRELAVSWALCAVCLLGHISHLFGVNAPLMHLFHSTGFHLSLSIFTFIGPGRRLIIDGLKSLFKGSP   160
weisuobuzhi   RKMGEKLQNLKQSGRELAVSWALCAVCLLGHISHLFGVNAPLMHLFHSTGFHLSLSIFTFIGPGRRLIIDGLKSLFKGSP   160
zhenong8      RKMGEKLQNLKQSGRELAVSWALCAVCLLGHISHLFGVNAPLMHLFHSTGFHLSLSIFTFIGPGRRLIIDGLKSLFKGSP   160
Consensus     rkmgeklqnlkqsgrelavswalcavcllghishlfgvnaplmhlfhstgfhlslsiftfigpgrrliidglkslfkgsp

suyinmai2     NMNTLVGLGALSSFAVSSVAAFIPKLGWKTFFEEPIMLIAFVLLGKNLEQRAKLKAASDMTGLLNILPSKARLMVDNDAE   240
weisuobuzhi   NMNTLVGLGALSSFAVSSVAAFIPKLGWKTFFEEPIMLIAFVLLGKNLEQRAKLKAASDMTGLLNILPSKARLMVDNDAE   240
zhenong8      NMNTLVGLGALSSFAVSSVAAFIPKLGWKTFFEEPIMLIAFVLLGKNLEQRAKLKAASDMTGLLNILPSKARLMVDNDAE   240
Consensus     nmntlvglgalssfavssvaafipklgwktffeepimliafvllgknleqraklkaasdmtgllnilpskarlmvdndae

suyinmai2     QSSFTEVPCGTLAVGDYILVLPGDRIPADGLVKAGRSTVDESSLTGEPMPVTKIAGAEVSAGSINLNGKLTVEVRRPGGE   320
weisuobuzhi   QSSFTEVPCGTLAVGDYILVLPGDRIPADGLVKAGRSTVDESSLTGEPMPVTKIAGAEVSAGSINLNGKLTVEVRRPGGE   320
zhenong8      QSSFTEVPCGTLAVGDYILVLPGDRIPADGLVKAGRSTVDESSLTGEPMPVTKIAGAEVSAGSINLNGKLTVEVRRPGGE   320
Consensus     qssftevpcgtlavgdyilvlpgdripadglvkagrstvdessltgepmpvtkiagaevsagsinlngkltvevrrpgge

suyinmai2     TVMSDILHLVEEAQTREAPVQRLADKVAGNFTYGVMALSSATFMFWSIFGSQLVPAAIQQGSAMSLALQLSCSVLVIACP   400
weisuobuzhi   TVMSDILHLVEEAQTREAPVQRLADKVAGNFTYGVMALSSATFMFWSIFGSQLVPAAIQQGSAMSLALQLSCSVLVIACP   400
zhenong8      TVMSDILHLVEEAQTREAPVQRLADKVAGNFTYGVMALSSATFMFWSIFGSQLVPAAIQQGSAMSLALQLSCSVLVIACP   400
Consensus     tvmsdilhlveeaqtreapvqrladkvagnftygvmalssatfmfwsifgsqlvpaaiqqgsamslalqlscsvlviacp

suyinmai2     CALGLATPTAVLVGTSLGATRGLLLRGGDVLEKFAEVDAIVFDKTGTLTIGKPVVTKVIASHSEGGVNTKDYRNNEWTEG   480
weisuobuzhi   CALGLATPTAVLVGTSLGATRGLLLRGGDVLEKFAEVDAIVFDKTGTLTIGKPVVTKVIASHSEGGVNTKDYRNNEWTEG   480
zhenong8      CALGLATPTAVLVGTSLGATRGLLLRGGDVLEKFAEVDAIVFDKTGTLTIGKPVVTKVIASHSEGGVNTKDYRNNEWTEG   480
Consensus     calglatptavlvgtslgatrglllrggdvlekfaevdaivfdktgtltigkpvvtkviashseggvntkdyrnnewteg

suyinmai2     DVLSLAAGVESNTNHPLGKAIMEAAQAANCINMKAKDGSFMEEPGSGAVATIGEKQVSVGTLDWIRRHGVVREPFPEAEN   560
weisuobuzhi   DVLSLAAGVESNTNHPLGKAIMEAAQAANCINMKAKDGSFMEEPGSGAVATIGEKQVSVGTLDWIRRHGVVREPFPEAEN   560
zhenong8      DVLSLAAGVESNTNHPLGKAIMEAAQAANCINMKAKDGSFMEEPGSGAVATIGEKQVSVGTLDWIRRHGVVREPFPEAEN   560
Consensus     dvlslaagvesntnhplgkaimeaaqaancinmkakdgsfmeepgsgavatigekqvsvgtldwirrhgvvrepfpeaen

suyinmai2     FGQSVAYVAVDGTLAGLICFEDKIREDSHQVINALSKQGISVYMLSGDKESAAMNVASIVGIQLDKVISEVKPHEKKKFI   640
weisuobuzhi   FGQSVAYVAVDGTLAGLICFEDKIREDSHQVINALSKQGISVYMLSGDKESAAMNVASIVGIQLDKVISEVKPHEKKKFI   640
zhenong8      FGQSVAYVAVDGTLAGLICFEDKIREDSHQVINALSKQGISVYMLSGDKESAAMNVASIVGIQLDKVISEVKPHEKKKFI   640
Consensus     fgqsvayvavdgtlaglicfedkiredshqvinalskqgisvymlsgdkesaamnvasivgiqldkvisevkphekkkfi

suyinmai2     SELQKEHKLVAMVGDGINDAAALALADVGIAMGGGVGAASDVSSVVLMGNRLSQLVDALELSKETMRTVKQNLWWAFLYN   720
weisuobuzhi   SELQKEHKLVAMVGDGINDAAALALADVGIAMGGGVGAASDVSSVVLMGNRLSQLVDALELSKETMRTVKQNLWWAFLYN   720
zhenong8      SELQKEHKLVAMVGDGINDAAALALADVGIAMGGGVGAASDVSSVVLMGNRLSQLVDALELSKETMRTVKQNLWWAFLYN   720
Consensus     selqkehklvamvgdgindaaalaladvgiamgggvgaasdvssvvlmgnrlsqlvdalelsketmrtvkqnlwwaflyn

suyinmai2     IVGLPVAAGALLPVTGTMLTPSIAGALMGFSSVSVMANSLLLRARMSSKHHVQSRQKPHNTISDVSDGAGEVEQSYPSKW   800
weisuobuzhi   IVGLPVAAGALLPVTGTMLTPSIAGALMGFSSVSVMANSLLLRARMSSKHHVQSRQKPHNTISDVSDGAGEVEQSYPSKW   800
zhenong8      IVGLPVAAGALLPVTGTMLTPSIAGALMGFSSVSVMANSLLLRARMSSKHHVQSRQKPHNTISDVSDGAGEVEQSYPSKW   800
Consensus     ivglpvaagallpvtgtmltpsiagalmgfssvsvmansllllrarmsskhhvqsrqkphntisdvsdgageveqsypskw

suyinmai2     RS                                                                                 802
weisuobuzhi   RS                                                                                 802
zhenong8      RS                                                                                 802
Consensus     rs
```

Figure S4. Alignment-based analysis of the CDS and amino acid sequences of *HvPAA1* from Suyinmai 2, Weisubuzhi and Zhenong 8. The dark blue represents 100% identity as defined by Mega7.

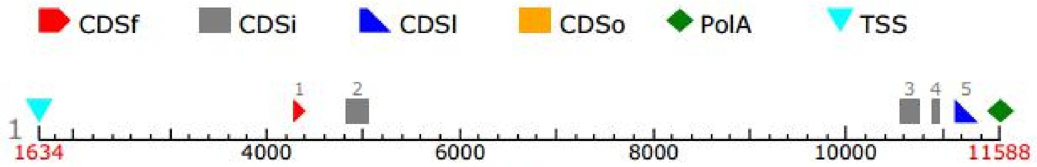

1 +		TSS	1634		-1.61					
1 +	1	CDSf	4267 -	4397	141.47	4267 -	4395	129	669	705
1 +	2	CDSi	4812 -	5049	381.50	4813 -	5049	237	707	785
1 +	3	CDSi	10546 -	10749	373.89	10546 -	10749	204	786	853
1 +	4	CDSi	10881 -	10961	138.24	10881 -	10961	81	854	880
1 +	5	CDSl	11113 -	11361	314.59	11113 -	11361	249	881	959
1 +		PolA	11588		-3.33					

```
Predicted protein(s):
>FGENESH:[mRNA] 1 5 exon (s) 4267 - 11361 903 bp, chain +
ATGCTCGCCATTTTCCCTTGTATATGGTTACAGGCAAAGGATGGGTCCTTTATGGAAGAA
CCAGGGTCTGGTGCTGTGGCTACGATTGGTGAAAAACAGGTTTCGGTTGGGACATTAGAC
TGGATTAGGAGGCATGGTGTTGTTCGTGAACCATTTCCTGAAGCAGAAAATTTTGGTCAG
TCTGTTGCATATGTAGCAGTTGACGGTACTCTAGCTGGTCTTATTTGTTTCGAGGATAAG
ATCAGAGAAGATTCTCATCAAGTTATCAATGCCCTGTCTAAGCAAGGAATTAGTGTGTAT
ATGTTATCTGGGGACAAGGAGAGTGCTGCTATGAATGTTGCCTCAATTGTTGGCATTCAG
TTAGACAAGGTGATTTCTGAAGTTAAACCACACGAGAAAAAGAAGTTCATATCTGAACTT
CAAAAGGAGCACAAATTAGTTGCCATGGTTGGTGATGGCATTAATGATGCTGCAGCCCTA
GCTTTAGCTGACGTTGGAATTGCAATGGGTGGAGGTGTTGGTGCAGCTAGTGACGTATCT
TCAGTTGTTCTCATGGGTAATAGGTTATCTCAGCTTGTTGATGCTTTAGAGTTAAGTAAA
GAAACCATGAGAACAGTGAAGCAAAATCTTTGGTGGGCTTTTCTGTATAACATTGTTGGG
CTACCCGTTGCTGCTGGAGCATTGCTGCCAGTGACGGGTACGATGCTGACCCCGTCGATA
GCTGGAGCACTCATGGGTTTTAGTTCAGTCAGCGTGATGGCCAATTCCTTGCTTTTGAGG
GCGAGGATGAGTTCAAAGCATCATGTTCAGAGCAGACAAAAGCCTCACAACACTATTTCT
GATGTGTCAGACGGGGCTGGTGAGGTAGAGCAAAGTTATCCATCAAAATGGAGGAGTACC
TGA
```

Figure S5 The predicted sequence of *HvPAA1* between the transcription start site and termination site. TSS, transcription start site. CDSf, the first CDS. CDSi, the intermediate CDS. CDSl, the last CDS. PolA, polA tag, the termination of transcription.

```
Suyinmai_2    ..............................GGAAGGTAGGATGGGTC.TTTATGGAG..ACCAGGGTCTGGTGCTGTGGC   47
Weisuobuzhi   .............................GGATGGTAGGGATGGGTCCTTTATGGAAG.ACCAGGGTCTGGTGCTGTGGC   50
Template      ATGCTCGCCATTTTCCCTTGTATATGGTTACAGGCAAAGGATGGGTCCTTTATGGAAGAACCAGGGTCTGGTGCTGTGGC   80
Consensus                                           ggatgggtc tttatgga   accagggtctggtgctgtggc

Suyinmai_2    TACGATTGGTGAAAAACAGGTTTCGGTTGGGACATTAGACTGGATTAGGAGGTACACTTGAATTATTTATTCAATTGTTG   127
Weisuobuzhi   TACGATTGGTGAAAAACAGGTTTCGGTTGGGACATTAGACTGGATTAGGAGGTACACTTGAATTATTTATTCAATTGTTG   130
Template      TACGATTGGTGAAAAACAGGTTTCGGTTGGGACATTAGACTGGATTAGGAGGTACACTTGAATTATTTATTCAATTGTTG   160
Consensus     tacgattggtgaaaaacaggtttcggttgggacattagactggattaggaggtacacttgaattatttattcaattgttg

Suyinmai_2    TCCTAAATAAGACTTGTTCTTTCACTAAGTTGCCAAAGTTTGATAGTCGTATTTCCGGTATCAGTTCTGTACTAGTGACA   207
Weisuobuzhi   TCCTAAATAAGACTTGTTCTTTCACTAAGTTGCCAAAGTTTGATAGTCGTATTTCCGGTATCAGTTCTGTACTAGTGACA   210
Template      TCCTAAATAAGACTTGTTCTTTCACTAAGTTGCCAAAGTTTGATAGTCGTATTTCCGGTATCAGTTCTGTACTAGTGACA   240
Consensus     tcctaaataagacttgttctttcactaagttgccaaagtttgatagtcgtatttccggtatcagttctgtactagtgaca

Suyinmai_2    TACTCCCTCCGTTCCTAAATATAAGTCTTTTTAGAGATATCAATAGGAGACTATATACGGAGCAAAATGAGTGAATCTAC   287
Weisuobuzhi   TACTCCCTCCGTTCCTAAATATAAGTCTTTTTAGAGATATCAATAGGAGACTATATACGGAGCAAAATGAGTGAATCTAC   290
Template      TACTCCCTCCGTTCCTAAATATAAGTCTTTTTAGAGATATCAATAGGAGACTATATACGGAGCAAAATGAGTGAATCTAC   320
Consensus     tactccctccgttcctaaatataagtctttttagagatatcaataggagactatatacggagcaaaatgagtgaatctac

Suyinmai_2    ACTCCAAAATAAGTCTATACATCCATATGTAGTCCCCTAATGAAACCTCTAAAAAGGCTTATATTTAGGAATGGAGGAAG   367
Weisuobuzhi   ACTCCAAAATAAGTCTATACATCCATATGTAGTCCCCTAATGAAACCTCTAAAAAGGCTTATATTTAGGAATGGAGGAAG   370
Template      ACTCTAAAATAAGTCTATACATCCATATGTAGTCCCCTAATGAAACCTCTAAAAAGGCTTATATTTAGGAATGGAGGAAG   400
Consensus     actc aaaataagtctatacatccatatgtagtcccctaatgaaacctctaaaaaggcttatatttaggaatggaggaag

Suyinmai_2    TATATGTTAACTCATTCTAGCAATACAACTTTGTTTGGATTATTGCATCAATTTTCTGCTCCGTTACACTAGCTCCATGT   447
Weisuobuzhi   TATATGTTAACTCATTCTAGCAATACAACTTTGTTTGGATTATTGCATCAATTTTCTGCTCCGTTACACTAGCTCCATGT   450
Template      TATATGTTAACTCATTCTAGCAATACAACTTTGTTTGGATTATTGCATCAATTTTCTGCTCCGTTACACTAGCTCCATGT   480
Consensus     tatatgttaactcattctagcaatacaactttgtttggattattgcatcaattttctgctccgttacactagctccatgt

Suyinmai_2    GTAATTGTTGTGTTGCACCGAGGAACTGTGCTCACTAGTAAACAAATGTTTTGACAATATCTCAGGCATGGTGTTGTTCG   527
Weisuobuzhi   GTAATTGTTGTGTTGCACCGAGGAACTGTGCTCACTAGTAAACAAATGTTTTGACAATATCTCAGGCATGGTGTTGTTCG   530
Template      GTAATTGTTGTGTTGCACCGAGGAACTGTGCTCACTAGTAAACAAATGTTTTGACAATATCTCAGGCATGGTGTTGTTCG   560
Consensus     gtaattgttgtgttgcaccgaggaactgtgctcactagtaaacaaatgttttgacaatatctcaggcatggtgttgttcg

Suyinmai_2    TGAACCATTTCCTGAAGCAGAAAATTTTGGTCAGTCTGTTGCATATGTAGCAGTTGACGGTACTCTAGCTGGTCTTATTT   607
Weisuobuzhi   TGAACCATTTCCTGAAGCAGAAAATTTTGGTCAGTCTGTTGCATATGTAGCAGTTGACGGTACTCTAGCTGGTCTTATTT   610
Template      TGAACCATTTCCTGAAGCAGAAAATTTTGGTCAGTCTGTTGCATATGTAGCAGTTGACGGTACTCTAGCTGGTCTTATTT   640
Consensus     tgaaccatttcctgaagcagaaaattttggtcagtctgttgcatatgtagcagttgacggtactctagctggtcttattt

Suyinmai_2    GTTTCGAGGATAAGATCAGAGAAGATTCTCATCAAGTTATCAATGCCCTGTCTAAGCAAGGAATTAGTGTGTATATGTTA   687
Weisuobuzhi   GTTTCGAGGATAAGATCAGAGAAGATTCTCATCAAGTTATCAATGCCCTGTCTAAGCAAGGAATTAGTGTGTATATGTTA   690
Template      GTTTCGAGGATAAGATCAGAGAAGATTCTCATCAAGTTATCAATGCCCTGTCTAAGCAAGGAATTAGTGTGTATATGTTA   720
Consensus     gtttcgaggataagatcagagaagattctcatcaagttatcaatgccctgtctaagcaaggaattagtgtgtatatgtta

Suyinmai_2    TCTGGGGACAAGGAGAGTGCTGCTATGAATGTTGCCTCAATTGTTGGCATTCAGTTAGACAAGGTAATATCAACTAAAGT   767
Weisuobuzhi   TCTGGGGACAAGGAGAGTGCTGCTATGAATGTTGCCTCAATTGTTGGCATTCAGTTAGACAAGGTAATATCAACTAAAGT   770
Template      TCTGGGGACAAGGAGAGTGCTGCTATGAATGTTGCCTCAATTGTTGGCATTCAGTTAGACAAGGTAATATCAACTAAAGT   800
Consensus     tctggggacaaggagagtgctgctatgaatgttgcctcaattgttggcattcagttagacaaggtaatatcaactaaagt

Suyinmai_2    AAATTTTGCTTTGCCGATGTGGACATTTTGTTCTGGCGAATAGTCCCCGTAATAAAACAACAAACTTCTGTAATATATGA   847
Weisuobuzhi   AAATTTTGCTTTGCCGATGTGGACATTTTGTTCTGGCGAATAGTCCCCGTAATAAAACAACAAACTTCTGTAATATATGA   850
Template      AAATTTTGCTTTGCCGATGTGGACATTTTGTTCTGGCGAATAGTCCCCGTAATAAAACAACAAACTTCTGTAATATATGA   880
Consensus     aaattttgctttgccgatgtggacattttgttctggcgaatagtccccgtaataaaacaacaaacttctgtaatatatga

Suyinmai_2    CATCAAAGTACGAGAATGAACATGTTGCAGGTGCTTGCTGCCTCTTCTGCTGTTGTCTGACTTCAAGTATGCAGTTTAAA   927
Weisuobuzhi   CATCAAAGTACGAGAATGAACATGTTGCAGGTGCTTGCTGCCTCTTCTGCTGTTGTCTGACTTCAAGTATGCAGTTTAAA   930
Template      CATCAAAGTACGAGAATGAACATGTTGCAGGTGCTTGCTGCCTCTTCTGCTGTTGTCTGACTTCAAGTATGCAGTTTAAA   960
Consensus     catcaaagtacgagaatgaacatgttgcaggtgcttgctgcctcttctgctgttgtctgacttcaagtatgcagtttaaa

Suyinmai_2    TTTTTTATATACATCTTAGAAGGTGCACATGAAGAAAAATAAATATGAATTCATCTTTTCATAAAGATTTTTTTTTTAAT   1007
Weisuobuzhi   TTTTTTATATACATCTTAGAAGGTGCACATGAAGAAAAATAAATATGAATTCATCTTTTCATAAAGATTTTTTTTTTAAT   1010
Template      TTTTTTATATACATCTTAGAAGGTGCACATGAAGAAAAATAAATATGAATTCATCTTTTCATAAAGATTTTTTTTTTAAT   1040
Consensus     ttttttatatacatcttagaaggtgcacatgaagaaaaataaatatgaattcatcttttcataaagattttttttttaat

Suyinmai_2    TTGTCATAAAGTTATGATATTGTTATGTACAGTATCTTTGTAGGAAATACTGAAAAAGGCTTTCGCCCCGCTTTATAGAT   1087
Weisuobuzhi   TTGTCATAAAGTTATGATATTGTTATGTACAGTATCTTTGTAGGAAATACTGAAAAAGGCTTTCGCCCCGCTTTATAGAT   1090
Template      TTGTCATAAAGTTATGATATTGTTATGTACAGTATCTTTGTAGGAAATACTGAAAAAGGCTTTCGCCCCGCTTTATAGAT   1120
Consensus     ttgtcataaagttatgatattgttatgtacagtatctttgtaggaaatactgaaaaaggctttcgccccgctttatagat

Suyinmai_2    AAAGCAAACCACCGAAGCATAACATCCGACAACAGTCATAACACCACACACACGCCGCACTGAGGTAGCACAACAGAGGG   1167
Weisuobuzhi   AAAGCAAACCACCGAAGCATAACATCCGACAACAGTCATAACACCACACACACGCCGCACTGAGGTAGCACAACAGAGGG   1170
Template      AAAGCAAACCACCGAAGCATAACATCCGACAACAGTCATAACACCACACACACGCCGCACTGAGGTAGCACAACAGAGGG   1200
Consensus     aaagcaaaccaccgaagcataacatccgacaacagtcataacaccacacacacgccgcactgaggtagcacaacagaggg
```

```
Suyinmai_2     TACAAAGGTTACGCTGTGGGCACAACACAACAAGCCCAAAGAAATAGAAACAACACGGCGGCGTGGCCTGGAGGAGGGAA  1247
Weisuobuzhi    TACAAAGGTTACGCTGTGGGCACAACACAACAAGCCCAAAGAAATAGAAACAACACGGCGGCGTGGCCTGGAGGAGGGAA  1250
Template       TACAAAGGTTACGCTGTGGGCACAACACAACAAGCCCAAAGAAATAGAAACAACACGGCGGCGTGGCCTGGAGGAGGGAA  1280
Consensus      tacaaaggttacgctgtgggcacaacacaacaagcccaaagaaatagaaacaacacggcggcgtggcctggaggagggaa

Suyinmai_2     CACTAATCCGGTTCCGGAGGTGGCGGAGGTAGCGGCGGCGCCAAGCGGAGAGCCATCGCACGAAGACGAGAGATGATGGA  1327
Weisuobuzhi    CACTAATCCGGTTCCGGAGGTGGCGGAGGTAGCGGCGGCGCCAAGCGGAGAGCCATCGCACGAAGACGAGAGATGATGGA  1330
Template       CACTAATCCGGTTCCGGAGGTGGCGGAGGTAGCGGCGGCGCCAAGCGGAGAGCCATCGCACGAAGACGAGAGATGATGGA  1360
Consensus      cactaatccggttccggaggtggcggaggtagcggcggcgccaagcggagagccatcgcacgaagacgagagatgatgga

Suyinmai_2     GTTGATGGCGTCTCGGTCCCGCGAGCGGCTAAGCGGCCGCCAGAGCTGCAGATATCCACACAATTTGAACACTGCGTCAG  1407
Weisuobuzhi    GTTGATGGCGTCTCGGTCCCGCGAGCGGCTAAGCGGCCGCCAGAGCTGCAGATATCCACACAATTTGAACACTGCGTCAG  1410
Template       GTTGATGGCGTCTCGGTCCCGCGAGCGGCTAAGCGGCCGCCAGAGCTGCAGATATCCACACAATTTGAACACTGCGTCAG  1440
Consensus      gttgatggcgtctcggtcccgcgagcggctaagcggccgccagagctgcagatatccacacaatttgaacactgcgtcag

Suyinmai_2     TAGCACGACGAAGAGGAACACGTTGAATAACAAGTTTTTTCCGAACAGTCCACATCGTCCAGGCCAGAACCCCAACCGTC  1487
Weisuobuzhi    TAGCACGACGAAGAGGAACACGTTGAATAACAAGTTTTTTCCGAACAGTCCACATCGTCCAGGCCAGAACCCCAACCGTC  1490
Template       TAGCACGACGAAGAGGAACACGTTGAATAACAAGTTTTTTCCGAACAGTCCACATCGTCCAGGCCAGAACCCCAACCGTC  1520
Consensus      tagcacgacgaagaggaacacgttgaataacaagttttttccgaacagtccacatcgtccaggccagaaccccaaccgtc

Suyinmai_2     AGCCACCTAGTGTGGCGGACAGATGGTGGCGTAGCCTGGAGTTCGGCGAAAAGGTCAGGGAAGTTTGTGTGGCACCAGGC  1567
Weisuobuzhi    AGCCACCTAGTGTGGCGGACAGATGGTGGCGTAGCCTGGAGTTCGGCGAAAAGGTCAGGGAAGTTTGTGTGGCACCAGGC  1570
Template       AGCCACCTAGTGTGGCGGACAGATGGTGGCGTAGCCTGGAGTTCGGCGAAAAGGTCAGGGAAGTTTGTGTGGCACCAGGC  1600
Consensus      agccacctagtgtggcggacagatggtggcgtagcctggagttcggcgaaaaggtcagggaagtttgtgtggcaccaggc

Suyinmai_2     GCCATCGACCACTTCGCGGAAACAACTCTAGAGGAATTGCGCGGACACGCAAGAGAAGAATATGTGATTCGAGTCTTCTC  1647
Weisuobuzhi    GCCATCGACCACTTCGCGGAAACAACTCTAGAGGAATTGCGCGGACACGCAAGAGAAGAATATGTGATTCGAGTCTTCTC  1650
Template       GCCATCGACCACTTCGCGGAAACAACTCTAGAGGAATTGCGCGGACACGCAAGAGAAGAATATGTGATTCGAGTCTTCTC  1680
Consensus      gccatcgaccacttcgcggaaacaactctagaggaattgcgcggacacgcaagagaagaatatgtgattcgagtcttctc

Suyinmai_2     CCGTCGCACAGAGCGGACATATGCCATCCCCTGGCCCGTTCCGCTTAAGGACCTTGACACCAGAGGGAAGGCGGCCACGA  1727
Weisuobuzhi    CCGTCGCACAGAGCGGACATATGCCATCCCCTGGCCCGTTCCGCTTAAGGACCTTGACACCAGAGGGAAGGCGGCCACGA  1730
Template       CCGTCGCACAGAGCGGACATATGCCATCCCCTGGCCCGTTCCGCTTAAGGACCTTGACACCAGAGGGAAGGCGGCCACGA  1760
Consensus      ccgtcgcacagagcggacatatgccatcccctggcccgttccgcttaaggaccttgacaccagagggaaggcggccacga

Suyinmai_2     AGCCATTGCCAGAGGAATATTCGAATCTTCAAGGGGAGTCAAATCTCCCAGAGCGCCGCGAGAGGCTCGATACCCTGGGA  1807
Weisuobuzhi    AGCCATTGCCAGAGGAATATTCGAATCTTCAAGGGGAGTCAAATCTCCCAGAGCGCCGCGAGAGGCTCGATACCCTGGGA  1810
Template       AGCCATTGCCAGAGGAATATTCGAATCTTCAAGGGGAGTCAAATCTCCCAGAGCGCCGCGAGAGGCTCGATACCCTGGGA  1840
Consensus      agccattgccagaggaatattcgaatcttcaaggggagtcaaatctcccagagcgccgcgagaggctcgataccctggga

Suyinmai_2     TGGCGTTATGGCCCGGTAGAGGGACTTCGTAGAGAAACGCCCTGATGGCTCTAGCCGCCAAGAGAGACAGTCGATGGATC  1887
Weisuobuzhi    TGGCGTTATGGCCCGGTAGAGGGACTTCGTAGAGAAACGCCCTGATGGCTCTAGCCGCCAAGAGAGACAGTCGATGGATC  1890
Template       TGGCGTTATGGCCCGGTAGAGGGACTTCGTAGAGAAACGCCCTGATGGCTCTAGCCGCCAAGAGAGACAGTCGATGGATC  1920
Consensus      tggcgttatggcccggtagagggacttcgtagagaaacgccctgatggctctagccgccaagagagacagtcgatggatc

Suyinmai_2     GCTCCATGTCCGTTCATGAAGGGCGATGGACTCCAGGAGGTCCTGCCAGGCGATCGTGTCCATAGGAGTCCCGAAAGGCC  1967
Weisuobuzhi    GCTCCATGTCCGTTCATGAAGGGCGATGGACTCCAGGAGGTCCTGCCAGGCGATCGTGTCCATAGGAGTCCCGAAAGGCC  1970
Template       GCTCCATGTCCGTTCATGAAGGGCGATGGACTCCAGGAGGTCCTGCCAGGCGATCGTGTCCATAGGAGTCCCGAAAGGCC  2000
Consensus      gctccatgtccgttcatgaagggcgatggactccaggaggtcctgccaggcgatcgtgtccataggagtcccgaaaggcc

Suyinmai_2     GTCTAAAAGCGAGGCGCCCGAGGTCGATAAGGGCCTCCTCCATTGAGATCCGAGGCTCGACCGCGATGGAGAATAGGGCT  2047
Weisuobuzhi    GTCTAAAAGCGAGGCGCCCGAGGTCGATAAGGGCCTCCTCCATTGAGATCCGAGGCTCGACCGCGATGGAGAATAGGGCT  2050
Template       GTCTAAAAGCGAGGCGCCCGAGGTCGATAAGGGCCTCCTCCATTGAGATCCGAGGCTCGACCGCGATGGAGAATAGGGCT  2080
Consensus      gtctaaaagcgaggcgcccgaggtcgataagggcctcctccattgagatccgaggctcgaccgcgatggagaatagggct

Suyinmai_2     GGGAAACGAGCAACAAAGGGGGTCACGCCGGCCCAGCGGTCAAACCAGAACAAGGTGGATGCGCTAGAGCCAACCTCGAT  2127
Weisuobuzhi    GGGAAACGAGCAACAAAGGGGGTCACGCCGGCCCAGCGGTCAAACCAGAACAAGGTGGATGCGCTAGAGCCAACCTCGAT  2130
Template       GGGAAACGAGCAACAAAGGGGGTCTCGCCGGCCCAGCGGTCAAACCAGAACAAGGTGGATGCGCTAGAGCCAACCTCGAT  2160
Consensus      gggaaacgagcaacaaagggggtc cgccggcccagcggtcaaaccagaacaaggtggatgcgctagagccaacctcgat

Suyinmai_2     AGAGGTCCCAATGCGAAGGACCGGGAGCAGCTGGATGAGGGACTGCCAGAACTAAGACCCCC.AGACCGCTGGCAGAAGG  2206
Weisuobuzhi    AGAGGTCCCAATGCGAAGGACCGGGAGCAGCTGGATGAGGGACTGCCAGAACTAAGACCCCC.AGACCGCTGGCAGAAGG  2209
Template       AGAGGTCCCAATGCGAAGGACCGGGAGCAGCTGGATGAGGGACTGCCAGAACTAAGACCCCCTAGACCGCTGGCAGAAGG  2240
Consensus      agaggtcccaatgcgaaggaccgggagcagctggatgagggactgccagaactaagaccccc agaccgctggcagaagg

Suyinmai_2     CTTATTCTGAATTATGCGAAGCCAGAGGCCCCCCTCGCCCTTAGCAATCCCTCGGAGCCAGTGGGTGGGCAGAGCGATAT  2286
Weisuobuzhi    CTTATTCTGAATTATGCGAAGCCAGAGGCCCCCCTCGCCCTTAGCAATCCCTCGGAGCCAGTGGGTGGGCAGAGCGATAT  2289
Template       CTTATTCTGAATTATGCGAAGCCAGAGGCCCCCCTCGCCCTTAGCAATCCCCCGGAGCCAGTGGGTGGGCAGAGCGATAT  2320
Consensus      cttattctgaattatgcgaagccagaggcccccctcgcccttagcaatccc cggagccagtgggtgggcagagcgatat

Suyinmai_2     TCATACGCTTGGAGGACATGATCCCAAGTCCACCCTGCTCGCGCGGCTTGCAGATCTCGGACCATCGCACCATGTGGTAC  2366
Weisuobuzhi    TCATACGCTTGGAGGACATGATCCCAAGTCCACCCTGCTCGCGCGGCTTGCAGATCTCGGACCATCGCACCATGTGGTAC  2369
Template       TCATACGCTTGGAGGACATGATCCCAAGTCCACCCTGCTCGCGCGGCTTGCAGATCTCGGACCATCGCACCATGTGGTAC  2400
Consensus      tcatacgcttggaggacatgatcccaagtccaccctgctcgcgcggcttgcagatctcggaccatcgcaccatgtggtac
```

```
Suyinmai_2    TTCTGCTTATCTCCCTCGCCGGCCCAGAAGAATCGCGCCTGAACCGTGGCGATCTCATGGTGGAGGGTCTCTGGCAAGCT  2446
Weisuobuzhi   TTCTGCTTATCTCCCTCGCCGGCCCAGAAGAATCGCGCCTGAACCGTGGCGATCTCATGGTGGAGGGTCTCTGGCAAGCT  2449
Template      TTCTGCTTATCTCCCTCGCCGGCCCAGAAGAATCGCGCCTGAACCGTGGCGATCTCATGGTGGAGGGTCTCTGGCAAGCT  2480
Consensus     ttctgcttatctccctcgccggcccagaagaatcgcgcctgaaccgtggcgatctcatggtggagggtctctggcaagct
```

```
Suyinmai_2    GTAGAAGCTCATCAAGAAGAGCAGCAAGCTGGACAAGGAGGAGTTGATGAGAATCGTCCTGACAGCCTT.GATAGCCACC  2525
Weisuobuzhi   GTAGAAGCTCATCAAGAAGAGCAGCAAGCCGGACAAGGAGGAGTTGATGAGAATCGTCCTGACAGCCTT.GATAGCCACC  2528
Template      GTAGAAGCTCATCAAGAAGAGCAGCAAGCTGGACAAGGAGGAGTTGATGAGAATCGTCCTGACAGCCTTCGATAGCCACC  2560
Consensus     gtagaagctcatcaagaagagcagcaagc ggacaaggaggagttgatgagaatcgtcctgacagcctt gatagccacc
```

```
Suyinmai_2    GCACCTGCCATGGTTCAATACGGTGCTGAAGCTTGCCGACCGTAGGTCGCAGCTCCGCAACCGAGGTGGGTGTCACTAAT  2605
Weisuobuzhi   GCACCTGCCATGGTTCAATACGGTGCTGAAGCTTGCCGACCGTAGGTCGCAGCTCCGCAACCGAGGTGGGTGTCACTAAT  2608
Template      GCACCTGCCATGGTTCAATACGGTGCTGAAGCTTGCCGACCGTAGGTCGCAGCTCCGCAACCGAGGTGGGTGTCACTAAT  2640
Consensus     gcacctgccatggttcaatacggtgctgaagcttgccgaccgtaggtcgcagctccgcaaccgaggtgggtgtcactaat
```

```
Suyinmai_2    GGGGATCCCCAGGTATGAAGTGGGGAAGGAGCCCAACTGACAATTGAGGCGGTCGGCAATGCTCTGGGCCGCGGCAGGAG  2685
Weisuobuzhi   GGGGATCCCCAGGTATGAAGTGGGGAAGGAGCCCAACTGACAATTGAGGCGGTCGGCAATGCTCTGGGCCGCGGCAGGAG  2688
Template      GGGGATCCCCAGGTATGAAGTGGGGAAGGAGCCCAACTGACAATTGAGGCGGTCGGCAATGCTCTGGGCCGCGGCAGGAG  2720
Consensus     ggggatccccaggtatgaagtggggaaggagcccaactgacaattgaggcggtcggcaatgctctgggccgcggcaggag
```

```
Suyinmai_2    GGTAGCCCAGGACCATGACCGCACTCTTATTAAAGTTTATTTTGAGACCGGACATATGTTGAAAGCAGAGGAGGAGGAAT  2765
Weisuobuzhi   GGTAGCCCAGGACCATGACCGCACTCTTATTAAAGTTTATTTTGAGACCGGACATATGTTGAAAGCAGAGGAGGAGGAAT  2768
Template      GGTAGCCCAGGACCATGACCGCACTCTTATTAAAGTTTATTTTGAGACCGGACATATGTTGAAAGCAGAGGAGGAGGAAT  2800
Consensus     ggtagcccaggaccatgaccgcactcttattaaagtttattttgagaccggacatatgttgaaagcagaggaggaggaat
```

```
Suyinmai_2    TTCAGGTACGAGATGTCATCGTCCGAGCCTTCAACCATGATGATCGTGTTGTCCGCATATTGCAGGAGAGAGACGCCAAG  2845
Weisuobuzhi   TTCAGGTACGAGATGTCATCGTCCGAGCCTTCAACCATGATGATCGTGTTGTCCGCATATTGCAGGAGAGAGACGCCAAG  2848
Template      TTCAGGTACGAGATGTCATCGTCCGAGCCTTCAACCATGATGATCGTGTTGTCCGCATATTGCAGGAGAGAGACGCCAAG  2880
Consensus     ttcaggtacgagatgtcatcgtccgagccttcaaccatgatgatcgtgttgtccgcatattgcaggagagagacgccaag
```

```
Suyinmai_2    ATCTCCCACTAGGTGCGGGACGATGCCGCGGATGTGGCCGGCCTCCTTGGCCTTGTCAAGGATGGAGGCTAGCGCATCGA  2925
Weisuobuzhi   ATCTCCCACTAGGTGCGGGACGATGCCGCGGATGTGGCCGGCCTCCTTGGCCTTGTCAAGGATGGAGGCTAGCGCATCGA  2928
Template      ATCTCCCACTAGGTGCGGGACGATGCCGCGGATGTGGCCGGCCTCCTTGGCCTTGTCAAGGATGGAGGCTAGCGCATCGA  2960
Consensus     atctcccactaggtgcgggacgatgccgcggatgtggccggcctccttggccttgtcaaggatggaggctagcgcatcga
```

```
Suyinmai_2    CCACCATGTTAAACAAGAACGGGGAGAAAGGATCACCCTAACGAACCCCACACATGGTGGGAAGTAGGGTCCGATCTCCC  3005
Weisuobuzhi   CCACCATGTTAAACAAGAACGGGGAGAAAGGATCACCCTAACGAACCCCACACATGGTGGGAAGTAGGGTCCGATCTCCC  3008
Template      CCACCATGTTAAACAAGAACGGGGAGAAAGGATCACCCTAACGAACCCCACACATGGTGGGAAGTAGGGTCCGATCTCCC  3040
Consensus     ccaccatgttaaacaagaacggggagaaaggatcaccctaacgaaccccacacatggtgggaagtagggtccgatctccc
```

```
Suyinmai_2    CATTGATGTTTACTGCGGTACGACCAGAGGAGACTATCTGCATCACCCTAGTCACCCACCGGTCGTCGAAGCCCTTACGC  3085
Weisuobuzhi   CATTGATGTTTACTGCGGTACGACCAGAGGAGACTATCTGCATCACCCTAGTCACCCACCGGTCGTCGAAGCCCTTACGC  3088
Template      CATTGATGTTTACTGCGGTACGACCAGAGGAGACTATCTGCATCACCCTAGTCACCCACCGGTCGTCGAAGCCCTTACGC  3120
Consensus     cattgatgtttactgcggtacgaccagaggagactatctgcatcaccctagtcacccaccggtcgtcgaagcccttacgc
```

```
Suyinmai_2    TGTAGAACTTCCCGAAGGAAATCCCAGCTAACAGTGTCATACGCCTTGTGAAAGTCCAGCTTCAAGAAGGGTCCCTAACA  3165
Weisuobuzhi   TGTAGAACTTCCCGAAGGAAATCCCAGCTAACAGTGTCATACGCCTTGTGAAAGTCCAGCTTCAAGAAGGGTCCCTAACA  3168
Template      TGTAGAACTTCCCGAAGGAAATCCCAGCTAACAGTGTCATACGCCTTGTGAAAGTCCAGCTTCAAGAAGGGTCCCTAACA  3200
Consensus     tgtagaacttcccgaaggaaatcccagctaacagtgtcatacgccttgtgaaagtccagcttcaagaagggtccctaaca
```

```
Suyinmai_2    GTGTCATTTGCCTTGAGGTTTTTGGACCGGACCTCATGGATAGTCTCATGAAGGACGAGCACACCATCCAGGATGTACCG  3245
Weisuobuzhi   GTGTCATTTGCCTTGAGGTTTTTGGACCGGACCTCATGGATAGTCTCATGAAGGACGAGCACACCATCCAGGATGTACCG  3248
Template      GTGTCATTTGCCTTGAGGTTTTTGGACCGGACCTCATGGATAGTCTCATGAAGGACGAGCACACCATCCAGGATGTACCG  3280
Consensus     gtgtcatttgccttgaggtttttggaccggacctcatggatagtctcatgaaggacgagcacaccatccaggatgtaccg
```

```
Suyinmai_2    GCCTCGGATGAAGGCCGATTGATCCGAATGTGTGATCTGATCAGCAAGAAGGGTCACCCTATTGGCGTACCCCTTGGCCA  3325
Weisuobuzhi   GCCTCGGATGAAGGCCGATTGATCCGAATGTGTGATCTGATCAGCAAGAAGGGTCACCCTATTGGCGTACCCCTTGGCCA  3328
Template      GCCTCGGATGAAGGCCGATTGATCCGAATGTGTGATCTGATCAGCAAGAAGGGTCACCCTATTGGCGTACCCCTTGGCCA  3360
Consensus     gcctcggatgaaggccgattgatccgaatgtgtgatctgatcagcaagaagggtcaccctattggcgtaccccttggcca
```

```
Suyinmai_2    GGATGCGGAAGATGACGTTGATCACCGTGATCGGCCGGAATTGGCTGATCTCAGCTGCACCCCAAACTTTTGGAATAAGC  3405
Weisuobuzhi   GGATGCGGAAGATGACGTTGATCACCGTGATCGGCCGGAATTGGCTGATCTCAGCTGCACCCCAAACTTTTGGAATAAGC  3408
Template      GGATGCGGAAGATGACGTTGATCACCGTGATCGGCCGGAATTGGCTGATCTCAGCTGCACCCCAAACTTTTGGAATAAGC  3440
Consensus     ggatgcggaagatgacgttgatcaccgtgatcggccggaattggctgatctcagctgcaccccaaacttttggaataagc
```

```
Suyinmai_2    GTGATAATCCCGAAGTTAAGGCGTGCGAGGTCAATTGAGCCAATGAAGAACTCGTCAAATATGGCCATGACCTCCGGTTT  3485
Weisuobuzhi   GTGATAATCCCGAAGTTAAGGCGTGCGAGGTCAATTGAGCCAATGAAGAACTCGTCAAATATGGCCATGACCTCCGGTTT  3488
Template      GTGATAATCCCGAAGTTAAGGCGTGCGAGGTCAATTGAGCCAATGAAGAACTCGTCAAATATGGCCATGACCTCTGGTTT  3520
Consensus     gtgataatcccgaagttaaggcgtgcgaggtcaattgagccaatgaagaactcgtcaaatatggccatgacctc ggttt
```

```
Suyinmai_2    AATGGTGTTCCAGAATGTTTGGAAGAACTTCACCGTCAAGCCATCCGGGCCCGGGGCGGAGGTCGGGTTCATGCCCTTGA  3565
Weisuobuzhi   AATGGTGTTCCAGAATGTTTGGAAGAACTTCACCGTCAAGCCATCCGGGCCCGGGGCGGAGGTCGGGTTCATGCCCTTGA  3568
Template      AATGGTGTTCCAGAATGTTTGGAAGAACTTCACCGTCAAGCCATCCGGGCCCGGGGCGGAGGTCGGGTTCATGCCCTTGA  3600
Consensus     aatggtgttccagaatgtttggaagaacttcaccgtcaagccatccgggcccggggcggaggtcgggttcatgcccttga
```

```
Suyinmai 2     TGGCTGACCAAACCTCCTCCTTCGAGAACGGAGCCATAAGCGCCAAGTTCTCCTCCGCAGATACGCGTCGATCGGTCGGC   3645
Weisuobuzhi    TGGCTGACCAAACCTCCTCCTTCGAGAACGGAGCCATAAGCGCCAAGTTCTCCTCCGCAGATACGCGTCGATCGGTCGGC   3648
Template       TGGCTGACCAAACCTCCTCCTTCGAGAACGGAGCCATAAGCGCCAAGTTCTCCTCCGCAGATACGCGTCGATCGGTCGGC   3680
Consensus      tggctgaccaaacctcctccttcgagaacggagccataagcgccaagttctcctccgcagatacgcgtcgatcggtcggc

Suyinmai 2     CAAATGTCGAGGGCCAGGGACACCCCTCCCCAAGGGGAAGAGGAAAACAAGGCTTTACAAAAGCCATCTACGTGGGCGCG   3725
Weisuobuzhi    CAAATGTCGAGGGCCAGGGACACCCCTCCCCAAGGGGAAGAGGAAAACAAGGCTTTACAAAAGCCATCTACGTGGGCGCG   3728
Template       CAAATGTCGAGGGCCAGGGACACCCCTCCCCAAGGGGAAGAGGAAAACAAGGCTTTACAAAAGCCATCTACGTGGGCGCG   3760
Consensus      caaatgtcgagggccagggacacccctccccaaggggaagaggaaaacaaggctttacaaaagccatctacgtgggcgcg

Suyinmai 2     GACGTCTGCCGACCACTGCAACAGGGTATCCCCGTCCCAGAGCCAGGGGATGGTGTTCCGGCCGCAGCGGCCATTAGCGA   3805
Weisuobuzhi    GACGTCTGCCGACCACTGCAACAGGGTATCCCCGTCCCAGAGCCAGGGGATGGTGTTCCGGCCGCAGCGGCCATTAGCGA   3808
Template       GACGTCTGCCGACCACTGCAACAGGGTATCCCCGTCCCAGAGCCAGGGGATGGTGTTCCGGCCGCAGCGGCCATTAGCGA   3840
Consensus      gacgtctgccgaccactgcaacagggtatccccgtcccagagccaggggatggtgttccggccgcagcggccattagcga

Suyinmai 2     TGGCTTGGAAGTAGGCCGTATTGGCGTCACCCTTGAGGACCCACTTTTGGGTGCCTCGCATCTGCCAATAATTCTCCTCG   3885
Weisuobuzhi    TGGCTCGGAAGTAGGCCGTATTGGCGTCACCCTTGAGGACCCACTTTTGGGTGCCTCGCATCTGCCAATAATTCTCCTCG   3888
Template       TGGCTTGGAAGTAGGCCGTATTGGCGTCACCCTTGAGGACCCACTTTTGGGTGCCTCGCATCTGCCAATAAGCCTCCTCG   3920
Consensus      tggct ggaagtaggccgtattggcgtcacccttgaggacccacttttgggtgcctcgcatctgccaataa  ctcctcg

Suyinmai 2     TTCGAGTAGATGACGGACAGTTGGTCCTCAAGATCGTATCGCAAAAAGCCATTCGTCGGCAGACAGACTCGAGGCGTCGG   3965
Weisuobuzhi    TTCGAGTAGATGACGGACAGTTGGTCCTCAAGATCGTATCGCAAAAAGCCATTCGTCGGCAGACAGACTCGAGGCGTCGG   3968
Template       TTCGAGTAGATGACGGACAGTTGGTCCTCAAGATCGTATCGCAAAAAGCCATTCGTCGGCAGACAGACTCGAGGCGTCGG   4000
Consensus      ttcgagtagatgacggacagttggtcctcaagatcgtatcgcaaaaagccattcgtcggcagacagactcgaggcgtcgg

Suyinmai 2     CCCTGGAGGTCGAGAAGCTGAATGGATGCTAGCAGAGCTTGCTTTTGCATCCGTAGATCCCATCCGAGGTTCGCGCCCCA   4045
Weisuobuzhi    CCCTGGAGGTCGAGAAGCTGAATGGATGCTAGCAGAGCTTGCTTTTGCATCCGTAGATCCCATCCGAGGTTCGCGCCCCA   4048
Template       CCCTGGAGGTCGAGAAGCTGAATGGATGCTAGCAGAGCTTGCTTTTGCATCCGTAGATCCCATCCGAGGTTCGCGCCCCA   4080
Consensus      ccctggaggtcgagaagctgaatggatgctagcagagcttgcttttgcatccgtagatcccatccgaggttcgcgcccca

Suyinmai 2     CCCCCTCATGAATTGGCGCGAACGCTTCGCACAGAGCTGCCATGCATCTACAGCGGAGGGTGCACGAAGGGGACGAGGTG   4125
Weisuobuzhi    CCCCCTCATGAATTGGCGCGAACGCTTCGCACAGAGCTGCCATGCATCTACAGCGGAGGGTGCACGAAGGGGACGAGGTG   4128
Template       CCCCCTCATGAATTGGCGCGAACGCTTCGCACAGAGCTGCCATGCATCTACAGCGGAGGGTGCACGAAGGGGACGAGGTG   4160
Consensus      ccccctcatgaattggcgcgaacgcttcgcacagagctgccatgcatctacagcggagggtgcacgaaggggacgaggtg

Suyinmai 2     AGCCTCCCGCCACTTATCGCACACAACCGCGTACAAACCCAGCCTGGTTAAGCCAGAATATCTTGAACCAGAACCGAGGA   4205
Weisuobuzhi    AGCCTCCCGCCACTTATCGCACACAACCGCG.ACAAACCCAGCCTGGTTAAGCCAGAATATCTTGAACCAGAACCGAGGA   4207
Template       AGCCTCCCGCCACTTATCGCACACAACCGCG.ACAAACCCAGCCTGGTTAAGCCAGAATATCTTGAACCAGAACCGAGGA   4239
Consensus      agcctcccgccacttatcgcacacaaccgcg acaaacccagcctggttaagccagaatatcttgaaccagaaccgagga

Suyinmai 2     GGTTGAGGCGGACGGTCGTCAACGGAAGAGAGGGAGGGGACATGGTCCGACCCAATCCTAGTAATCGCCTTAAGCGAAG   4285
Weisuobuzhi    GGTTGAGGCGGACGGTCGTCAACGGAAGAGAGGGAGGGGACATGGTCCGACCCAATCCTAGTAATCGCCTTAAGCGAAG   4287
Template       GGTTGAGGCGGACGGTCGTCAACGGAAGAGAGGGAGGGGACATGGTCCGACCCAATCCTAGTAATCGCCTTAAGCGAAG   4319
Consensus      ggttgaggcggacggtcgtcaacggaagagagggaggggacatggtccgacccaatcctagtaatcgccttaagcgaag

Suyinmai 2     CAAGAGGGCAGCAAAGCTCCCACTCTGGGGAGACGAGGACCTGGTCGAGGACGGAGCGGGTCGGGTCGGCCTGGCGGTTG   4365
Weisuobuzhi    CAAGAGGGCAGCAAAGCTCCCACTCTGGGGAGACGAGGACCTGGTCGAGGACGGAGCGGGTCGGGTCGGCCTGGCGGTTG   4367
Template       CAAGAGGGCAGCAAAGCTCCCACTCTGGGGAGACGAGGACCTGGTCGAGGACGGAGCGGGTCGGGTCGGCCTGGCGGTTG   4399
Consensus      caagagggcagcaaagctcccactctggggagacgaggacctggtcgaggacggagcgggtcgggtcggcctggcggttg

Suyinmai 2     GTCCAGGTAAACCTGGCCCCCACTCTATCAAGTTCCCGAAGGCCAAGGTCCACGATGCAATCGTTGAACATCTGCATCCT   4445
Weisuobuzhi    GTCCAGGTAAACCTGGCCCCCACTCTATCAAGTTCCCGAAGGCCAAGGTCCACGATGCAATCGTTGAACATCTGCATCCT   4447
Template       GTCCAGGTAAACCTGGCCCCCACTCTATCAAGTTCCCGAAGGCCAAGGTCCACGATGCAATCGTTGAACATCTGCATCCT   4479
Consensus      gtccaggtaaacctggcccccactctatcaagttcccgaaggccaaggtccacgatgcaatcgttgaacatctgcatcct

Suyinmai 2     TGGAAAGTTGACCAAGTTATTGCTTTTATCTGCCTCCAAGCGAATGAGATTTAAAGTCGCCATCAACGACCAGAGGGAGCT   4525
Weisuobuzhi    TGGAAAGTTGACCAAGTTATTGCTTTTATCTGCCTCCAAGCGAATGAGATTTAAAGTCGCCATCAACGACCAGAGGGAGCT   4527
Template       TGGAAAGTTGACCAAGTTATTGCTTTTATCTGCCTCCAAGCGAATGAGATTTAAAGTCGCCATCAACGACCAGAGGGAGCT   4559
Consensus      tggaaagttgaccaagttattgcttttatctgcctccaagcgaatgagatttaaagtcgccatcaacgaccagagggagct

Suyinmai 2     GGGCTGCTGCTACTTTCCTCTTTAATTCTTCGAGGAAAGCCGCCGAACGGCGGTGGTCGGTCGGGCCATAGATGACGATC   4605
Weisuobuzhi    GGGCTGCTGCTACTTTCCTCTTTAATTCTTCGAGGAAAGCCGCCGAACGGCGGTGGTCGGTCGGGCCATAGATGACGATC   4607
Template       GGGCTGCTGCTACTTTCCTCTTTAATTCTTCGAGGAAAGCCGCCGAACGGCGGTGGTCGGTCGGGCCATAGATGACGATC   4639
Consensus      gggctgctgctactttcctctttaattcttcgaggaaagccgccgaacggcggtggtcggtcgggccatagatgacgatc

Suyinmai 2     ACCTCCCACTTGACGTTCAAAGCCCGTTCAAAGAGTTCCATGCTAACATAGAACTCTCCGCCATCCATACGACCTATCTC   4685
Weisuobuzhi    ACCTCCCACTTGACGTTCAAAGCCCGTTCAAAGAGTTCCATGCTAACATAGAACTCTCCGCCATCCATACGACCTATCTC   4687
Template       ACCTCCCACTTGACGTTCAAAGCCCGTTCAAAGAGTTCCATGCTAACATAGAACTCTCCGCCATCCATACGACCTATCTC   4719
Consensus      acctcccacttgacgttcaaagcccgttcaaagagttccatgctaacatagaactctccgccatccatacgacctatctc

Suyinmai 2     AAAGGTGGCATCCTTCACTCCTAGGAGGATGCCGCCCGAGTGGCTAGTGGTCCCACTAGATGGGAGCCAGTGCCAGGCAA   4765
Weisuobuzhi    AAAGGTGGCATCCTTCACTCCTAGGAGGATGCCGCCCGAGTGGCTAGTGGTCCCACTAGATGGGAGCCAGTGCCAGGCAA   4767
Template       AAAGGTGGCATCCTTCACTCCTAGGAGGATGCCGCCCGAGTGGCTAGTGGTCCCACTAGATGGGAGCCAGTGCCAGGCAA   4799
Consensus      aaaggtggcatccttcactcctaggaggatgccgcccgagtggctagtggtcccactagatgggagccagtgccaggcaa
```

```
Suyinmai 2     ACAGATGGGAGCTCAGGGGCTTGAGCTCCGAGAGCGATAACTCAGTTCGCAATGTCTCCTGAATGGCATCTATGTCAATC  4845
Weisuobuzhi    ACAGATGGGAGCTCAGGGGCTTGAGCTCCGAGAGCGATAACTCAGTTCGCAATGTCTCCTGAATGGCATCTATGTCAATC  4847
Template       ACAGATGGGAGCTCAGGGGCTTGAGCTCCGAGAGCGATAACTCAGTTCGCAATGTCTCCTGAATGGCATCTATGTCAATC  4879
Consensus      acagatgggagctcaggggcttgagctccgagagcgataactcagttcgcaatgtctcctgaatggcatctatgtcaatc

Suyinmai 2     CGTTCTTCACGCATGTATTCGATCAACTGGCGGCGGCGGCCATCCTGACTGAAACCTCGGATGTTCCAGAAGAGCGTTCG  4925
Weisuobuzhi    CGTTCTTCACGCATGTATTCGATCAACTGGCGGCGGCGGCCATCCTGACTGAAACCTCGGATGTTCCAGAAGAGCGTTCG  4927
Template       CGTTCTTCACGCATGTATTCGATCAACTGGCGGCGGCGGCCATCCTGACTGAAACCTCGGATGTTCCAGAAGAGCGTTCG  4959
Consensus      cgttcttcacgcatgtattcgatcaactggcggcggcggccatcctgactgaaacctcggatgttccagaagagcgttcg

Suyinmai 2     CATCACTGACCCATCGGGGGGGCTGCTTGACCCCAGGACCCGAGAAGCGCTCTGGGCGCGTAGGATAGCGGTGTGGGTGC  5005
Weisuobuzhi    CATCACTGACCCATCGGGGGGGCTGCTTGACCCCAGGACCCGAGAAGCGCTCTGGGCGCGTAGGATAGCGGTGTGGGTGC  5007
Template       CATCACTGACCCATCGGGGGGGCTGCTTGACCCCAGGACCCGAGAAGCGCTCTGGGCGCGTAGGATAGCGGTGTGGGTGC  5039
Consensus      catcactgacccatcgggggggctgcttgaccccaggacccgagaagcgctctgggcgcgtaggatagcggtgtgggtgc

Suyinmai 2     GTGTGCGGTCCCAGGCCTCGACCGGCACGACCGAGTTGCGAGGGGCCCGTGCTGGCATGGGGGAAGCGGAGACGTCCGCT  5085
Weisuobuzhi    GTGTGCGGTCCCAGGCCTCGACCGGCACGACCGAGTTGCGAGGGGCCCGTGCTGGCATGGGGGAAGCGGAGACGTCCGCT  5087
Template       GTGTGCGGTCCCAGGCCTCGACCGGCACGACCGAGTTGCGAGGGGCCCGTGCTGGCATGGGGGAAGCGGAGACGTCCGCT  5119
Consensus      gtgtgcggtcccaggcctcgaccggcacgaccgagttgcgaggggcccgtgctggcatgggggaagcggagacgtccgct

Suyinmai 2     CCGCCCTGGGGCTGTAAGCGAGAGCGAGCCTCGGCCAGCCGACCATCGAGAATTTCTCGAGCACAGATCGCCTTGATTTG  5165
Weisuobuzhi    CCGCCCTGGGGCTGTAAGCGAGAGCGAGCCTCGGCCAGCCGACCATCGAGAATTTCTCGAGCACAGATCGCCTTGATTTG  5167
Template       CCGCCCTGGGGCTGTAAGCGAGAGCGAGCCTCGGCCAGCCGACCATCGAGAATTTCTCGAGCACAGATCGCCTTGATTTG  5199
Consensus      ccgccctggggctgtaagcgagagcgagcctcggccagccgaccatcgagaatttctcgagcacagatcgccttgatttg

Suyinmai 2     GACCAACGGCGGGGCGACTTCGCCCCGGAACACTATCGCAGAATCAGCCGCGATTTTCGCAAGACGGCCGAGCGGAATCG  5245
Weisuobuzhi    GACCAACGGCGGGGCGACTTCGCCCCGGAACACTATCGCAGAATCAGCCGCGATTTTCGCAAGACGGCCGAGCGGAATCG  5247
Template       GACCAACGGCGGGGCGACTTCGCCCCGGAACACTATCGCAGAATCAGCCGTGATTTTCGCAAGACGGCCGAGCGGAATCG  5279
Consensus      gaccaacggcggggcgacttcgccccggaacactatcgcagaatcagccg gattttcgcaagacggccgagcggaatcg

Suyinmai 2     ATTCAAGAGCCGAGATTCAAGAGCCGAGAACGAGCAGGTAGCAGTAGAAGCAGGGATGGCAGGGGTACCTGGCTCCAGGT  5325
Weisuobuzhi    ATTCAAGAGCCGAGATTCAAGAGCCGAGAACGAGCAGGTAGCAGTAGAAGCAGGGATGGCAGGGGTACCTGGCTCCAGGT  5327
Template       ATTCAAGAGCCGAGATTCAAGAGCCGAGAACGAGCAGGTAGCAGTAGAAGCAGGGATGGCAGGGGTACCTGGCTCCAGGT  5359
Consensus      attcaagagccgagattcaagagccgagaacgagcaggtagcagtagaagcagggatggcaggggtacctggctccaggt

Suyinmai 2     TCCGCGTAGCCGCCCGTAGCTCGGCCCGCTTCGGGATGGGCGGAGCAGGGCTCCCGTCGGGGTGTGCTGCCTCGAGCCGG  5405
Weisuobuzhi    TCCGCGTAGCCGCCCGTAGCTTGGCCCGCTTCGGGATGGGCGGAGCAGGGCTCCCGTCGGGGTGTGCTGCCTCGAGCCGG  5407
Template       TCCGCGTAGCCGCCCGTAGCTCGGCCCGCTTCGGGATGGGCGGAGCAGGGCTCCCGTCGGGGTGTGCTGCCTCGAGCCGG  5439
Consensus      tccgcgtagccgcccgtagct ggcccgcttcgggatgggcggagcagggctcccgtcggggtgtgctgcctcgagccgg

Suyinmai 2     GCACTGTGGCGAGAAGACGTGACCGGGTCGAGGATGCGCAGCCCCGCCTGGAGTAGGCCGCGGTGCACGGATAGAGCGGG  5485
Weisuobuzhi    GCACTGTGGCGAGAAGACGTGACCGGGTCGAGGATGCGCAGCCCCGCCTGGAGTAGGCCGCGGTGCACGGATAGAGCGGG  5487
Template       GCACTGTGGCGAGAAGACGTGACCGGGTCGAGGACGCGCAGCCCCGCCTGGAGTAGGCCGCGGTGCACGGATAGAGCGGG  5519
Consensus      gcactgtggcgagaagacgtgaccgggtcgagga gcgcagccccgcctggagtaggccgcggtgcacggatagagcggg

Suyinmai 2     TGGGAAGCCACCGGAGTGGTAATCACCGCAGACGCGAGGCCCGTGGCGTCGAAGCAGAGGGCGAGGTCGGGAGGGCCGAG  5565
Weisuobuzhi    TGGGAAGCCACCGGAGTGGTAATCACCGCAGACGCGAGGCCCGTGGCGTCGAAGCAGAGGGCGAGGTCGGGAGGGCCGAG  5567
Template       TGGGAAGCCACCGGAGTGGTAATCACCGCAGACGCGAGGCCCGTGGCGTCGAAGCAGAGGGCGAGGTCGGGAGGGCCGAG  5599
Consensus      tgggaagccaccggagtggtaatcaccgcagacgcgaggcccgtggcgtcgaagcagagggcgaggtcgggagggccgag

Suyinmai 2     CAGCACAGGGAGGCCGGGGAGACCGAACGTCGAAGTCACGGCCGCCCGCGAAGGCGAAGCCGAGAGCGCGACCCGGGAGG  5645
Weisuobuzhi    CAGCACAGGGAGGCCGGGGAGACCGAACGTCGAAGTCACGGCCGCCCGCGAAGGCGAAGCCGAGAGCGCGACCCGGGAGG  5647
Template       CAGCACAGGGAGGCCGGGGAGACCGAACGTCGAAGTCACGGCCGCCCGCGAAGGCGAAGCCGAGAGCGCGACCCGGGAGG  5679
Consensus      cagcacagggaggccggggagaccgaacgtcgaagtcacggccgcccgcgaaggcgaagccgagagcgcgacccgggagg

Suyinmai 2     GGGGGGGGGACCGCCAGCGCCACAGGTACCACTGGGGGAGGCTTGGAGACATCCGCCAGAGGTGGCTGTGGTGACGGTGT  5725
Weisuobuzhi    GGGGGGGGGACCGCCAGCGCCACAGGTACCACTGGGGGAGGCTTGGAGACATCCGCCAGAGGTGGCTGTGGTGACGGTGT  5727
Template       GGGGGG..GACCGCCAGCGCCACAGGTACCACTGGGGGAGGCTTGGAGACATCCGCCAGAGGTGGCTGTGGTGACGGTGT  5757
Consensus      gggggg  gaccgccagcgccacaggtaccactgggggaggcttggagacatccgccagaggtggctgtggtgacggtgt

Suyinmai 2     CCAGAGCGCCGCCCGCCGGAGGGGGCTAGGGCGACAGCGCGTCGGTGTCCAGAGCCCCAAGAGTTGGGGTGGCCGCGGCC  5805
Weisuobuzhi    CCAGAGCGCCGCCCGCCGGAGGGGGCTAGGGCGACAGCGCGTCGGTGTCCAGAGCCCCAAGAGTTGGGGTGGCCGCGGCC  5807
Template       CCAGAGCGCCGCCCGCCGGAGGGGGCTAGGGCGACAGCGCGTCGGTGTCCAGAGCCCCAAGAGTTGGGGTGGCCGCGGCC  5837
Consensus      ccagagcgccgcccgccggagggggctagggcgacagcgcgtcggtgtccagagccccaagagttggggtggccgcggcc

Suyinmai 2     AGCATCACGGCAGGAGGTCGGGGCGACGACGGCGGGAGCACCAGCAGGCCGACGCTGGACGTGTCACTCGTCTCCGCCGA  5885
Weisuobuzhi    AGCATCACGGCAGGAGGTCGGGGCGACGACGGCGGGAGCACCAGCAGGCCGACGCTGGACGTGTCACTCGTCTCCGCCGA  5887
Template       AGCATCACGGCAGGAGGTCGGGGCGACGACGGCGGGAGCACCAGCAGGCCGACGCTGGACGTGTCACTCGTCTCCGCCGA  5917
Consensus      agcatcacggcaggaggtcggggcgacgacggcgggagcaccagcaggccgacgctggacgtgtcactcgtctccgccga

Suyinmai 2     GCGGGTCAGGGAGGCTTGGCTGGAGAGCTCACGGGGTCCCCGCTCGTCGCGGACGGCAGCGAGGGGAGTGAGCATCCCGG  5965
Weisuobuzhi    GCGGGTCAGGGAGGCTTGGCTGGAGAGCTCACGGGGTCCCCGCTCGTCGCGGACGGCAGCGAGGGGAGTGAGCATCCCGG  5967
Template       GCGGGTCAGGGAGGCTTGGCTGGAGAGCTCACGGGGTCCCCGCTCGTCGCGGACGGCAGCGAGGGGAGTGAGCATCCCGG  5997
Consensus      gcgggtcagggaggcttggctggagagctcacggggtccccgctcgtcgcggacggcagcgaggggagtgagcatcccgg
```

```
Suyinmai 2     ACCCGAGCATCCTATTCATGCTTTTGATTATGCATGAAAATCTTACAGCAAGCTGAAAGCCGTTAATATGTAAATTGACT   6045
Weisuobuzhi    ACCCGAGCATCCTATTCATGCTTTTGATTATGCATGAAAATCTTACAGCAAGCTGAAAGCCGTTAATATGTAAATTGACT   6047
Template       ACCCGAGCATCCTATTCATGCTTTTGATTATGCATGAAAATCTTACAGCAAGCTGAAAGCCGTTAATATGTAAATTGACT   6077
Consensus      acccgagcatcctattcatgcttttgattatgcatgaaaatcttacagcaagctgaaagccgttaatatgtaaattgact

Suyinmai 2     TCATCTGGTATTATCAATTTCTCATCCCCATGCACTATTGCTCGAACATAAAGATCTATATGATTGAAACCAATTCAGCC   6125
Weisuobuzhi    TCATCTGGTATTATCAATTTCTCATCCCCATGCACTATTGCTCGAACATAAAGATCTATATGATTGAAACCAATTCAGCC   6127
Template       TCATCTGGTATTATCAATTTCTCATCCCCATGCACTATTGCTCGAACATAAAGATCTATATGATTGAAACCAATTCAGCC   6157
Consensus      tcatctggtattatcaatttctcatccccatgcactattgctcgaacataaagatctatatgattgaaaccaattcagcc

Suyinmai 2     ACAAAAGGAAGCATGAAGAAACGGTTGTTTCATTCATTAGGATTCTTTTTCTTACCATGATTTGTGTATCCTTTGTTACC   6205
Weisuobuzhi    ACAAAAGGAAGCATGAAGAAACGGTTGTTTCATTCATTAGGATTCTTTTTCTTACCATGATTTGTGTATCCTTTGTTACC   6207
Template       ACAAAAGGAAGCATGAAGAAACGGTTGTTTCATTCATTAGGATTCTTTTTCTTACCATGATTTGTGTATCCTTTGTTACC   6237
Consensus      acaaaaggaagcatgaagaaacggttgtttcattcattaggattctttttcttaccatgatttgtgtatcctttgttacc

Suyinmai 2     TTTTCAGTATGATTACTTGTTTACTTAATTAAATGGTATCAGGTGATTTCTGAAGTTAAACCACACGAGAAAAAGAAGTT   6285
Weisuobuzhi    TTTTCAGTATGATTACTTGTTTACTTAATTAAATGGTATCAGGTGATTTCTGAAGTTAAACCACACGAGAAAAAGAAGTT   6287
Template       TTTTCAGTATGATTACTTGTTTACTTAATTAAATGGTATCAGGTGATTTCTGAAGTTAAACCACACGAGAAAAAGAAGTT   6317
Consensus      ttttcagtatgattacttgtttacttaattaaatggtatcaggtgatttctgaagttaaaccacacgagaaaaagaagtt

Suyinmai 2     CATATCTGAACTTCAAAAGGAGCACAAATTAGTTGCCATGGTTGGTGATGGCATTAATGATGCTGCAGCCCTAGCTTTAG   6365
Weisuobuzhi    CATATCTGAACTTCAAAAGGAGCACAAATTAGTTGCCATGGTTGGTGATGGCATTAATGATGCTGCAGCCCTAGCTTTAG   6367
Template       CATATCTGAACTTCAAAAGGAGCACAAATTAGTTGCCATGGTTGGTGATGGCATTAATGATGCTGCAGCCCTAGCTTTAG   6397
Consensus      catatctgaacttcaaaaggagcacaaattagttgccatggttggtgatggcattaatgatgctgcagccctagctttag

Suyinmai 2     CTGACGTTGGAATTGCAATGGGTGGAGGTGTTGGTGCAGCTAGTGACGTATCTTCAGTTGTTCTCATGGGTAATAGGTTA   6445
Weisuobuzhi    CTGACGTTGGAATTGCAATGGGTGGAGGTGTTGGTGCAGCTAGTGACGTATCTTCAGTTGTTCTCATGGGTAATAGGTTA   6447
Template       CTGACGTTGGAATTGCAATGGGTGGAGGTGTTGGTGCAGCTAGTGACGTATCTTCAGTTGTTCTCATGGGTAATAGGTTA   6477
Consensus      ctgacgttggaattgcaatgggtggaggtgttggtgcagctagtgacgtatcttcagttgttctcatgggtaataggtta

Suyinmai 2     TCTCAGGTAATTCATTCACGACAGCTTGGAGTTACTCGCTGATACTGTTGCCAAATTCATTTAACTTCACTCCCCTCTTT   6525
Weisuobuzhi    TCTCAGGTAATTCATTCACGACAGCTTGGAGTTACTCGCTGATACTGTTGCCAAATTCATTTAACTTCACTCCCCTCTTT   6527
Template       TCTCAGGTAATTCATTCACGACAGCTTGGAGTTACTCGCTGATACTGTTGCCAAATTCATTTAACTTCACTCCCCTCTTT   6557
Consensus      tctcaggtaattcattcacgacagcttggagttactcgctgatactgttgccaaattcatttaacttcactcccctcttt

Suyinmai 2     GTGCTACTATCATCTTTCTTTTTCCTAATAAACTGCTGTGATGGGTCTTTGATGCAGCTTGTTGATGCTTTAGAGTTAAG   6605
Weisuobuzhi    GTGCTACTATCATCTTTCTTTTTCCTAATAAACTGCTGTGATGGGTCTTTGATGCAGCTTGTTGATGCTTTAGAGTTAAG   6607
Template       GTGCTACTATCATCTTTCTTTTTCCTAATAAACTGCTGTGATGGGTCTTTGATGCAGCTTGTTGATGCTTTAGAGTTAAG   6637
Consensus      gtgctactatcatctttctttttcctaataaactgctgtgatgggtctttgatgcagcttgttgatgctttagagttaag

Suyinmai 2     TAAAGAAACCATGAGAACAGTGAAGCAAAATCTTTGGTGGGCTTTTCTGTATAACATTGTACGGTTCCCTTTCTCGTCTC   6685
Weisuobuzhi    TAAAGAAACCATGAGAACAGTGAAGCAAAATCTTTGGTGGGCTTTTCTGTATAACATTGTACGGTTCCCTTTCTCGTCTC   6687
Template       TAAAGAAACCATGAGAACAGTGAAGCAAAATCTTTGGTGGGCTTTTCTGTATAACATTGTACGGTTCCCTTTCTCGTCTC   6717
Consensus      taaagaaaccatgagaacagtgaagcaaaatctttggtgggcttttctgtataacattgtacggttccctttctcgtctc

Suyinmai 2     TTTATGCCTTGCTCATGTGTTTAATAAGTTATCAACTGCCAGGATAATGTTGGACCTCAATTTTAAAGAAGTTACACTTA   6765
Weisuobuzhi    TTTATGCCTTGCTCATGTGTTTAATAAGTTATCAACTGCCAGGATAATGTTGGACCTCAATTTTAAAGAAGTTACACTTA   6767
Template       TTTATGCCTTGCTCATGTGTTTAATAAGTTATCAACTGCCAGGATAATGTTGGACCTCAATTTTAAAGAAGTTACACTTA   6797
Consensus      tttatgccttgctcatgtgtttaataagttatcaactgccaggataatgttggacctcaattttaaagaagttacactta

Suyinmai 2     GATTAAAAATATATGGCTGCTGGCCTCGTTATGATGTGATACTTTGCAGGTTGGGCTACCCGTTGCTGCTGGAGCATTGC   6845
Weisuobuzhi    GATTAAAAATATATGGCTGCTGGCCTCGTTATGATGTGATACTTTGCAGGTTGGGCTACCCGTTGCTGCTGGAGCATTGC   6847
Template       GATTAAAAATATATGGCTGCTGGCCTCGTTATGATGTGATACTTTGCAGGTTGGGCTACCCGTTGCTGCTGGAGCATTGC   6877
Consensus      gattaaaaatatatggctgctggcctcgttatgatgtgatactttgcaggttgggctacccgttgctgctggagcattgc

Suyinmai 2     TGCCAGTGACGGGTACGATGCTGACCCCGTCGATAGCTGGAGCACTCATGGGTTTTAGTTCAGTCAGCGTGATGGCCAAT   6925
Weisuobuzhi    TGCCAGTGACGGGTACGATGCTGACCCCGTCGATAGCTGGAGCACTCATGGGTTTTAGTTCAGTCAGCGTGATGGCCAAT   6927
Template       TGCCAGTGACGGGTACGATGCTGACCCCGTCGATAGCTGGAGCACTCATGGGTTTTAGTTCAGTCAGCGTGATGGCCAAT   6957
Consensus      tgccagtgacgggtacgatgctgaccccgtcgatagctggagcactcatgggttttagttcagtcagcgtgatggccaat

Suyinmai 2     TCCTTGCTTTTGAGGGCGAGGATGAGTTCAAAGCATCATGTTCAGAGCAGACAAAAGCCTCACAACACTATTTCTGATGT   7005
Weisuobuzhi    TCCTTGCTTTTGAGGGCGAGGATGAGTTCAAAGCATCATGTTCAGAGCAGACAAAAGCCTCACAACACTATTTCTGATGT   7007
Template       TCCTTGCTTTTGAGGGCGAGGATGAGTTCAAAGCATCATGTTCAGAGCAGACAAAAGCCTCACAACACTATTTCTGATGT   7037
Consensus      tccttgcttttgagggcgaggatgagttcaaagcatcatgttcagagcagacaaaagcctcacaacactatttctgatgt

Suyinmai 2     GTCAGACGGGCTGGAGTGAGCAA...................................     7028
Weisuobuzhi    GTCAGACGGGGCTGGAGAGAGCAAAGGTT.............................     7036
Template       GTCAGACGGGGCTGGTGAGGTAGAGCAAAGTTATCCATCAAAATGGAGGAGTACCTGA     7095
Consensus      gtcagacggg    g
```

Figure S6 Alignment-based analysis of the full length sequences of *HvPAA1*, including the exons and introns, in Suyinmai 2 and Weisuobuzhi.

Table S1 List of PCR primers used for *HvPAA1* cloning and functional analysis

Gene	Forward primer（5' → 3'）
HvPAA1-RT-PCR-F	ATGTGCTCTTGGTCTTGCCA
HvPAA1-RT-PCR-R	TCCCTCGCTGTGAGAAGCTA
HvPAA1-CDS-F	ATGTCTTGTGGGGGATGTGCA
HvPAA1-CDS-R	TCAGGTACTCCTCCATTTTGA
35SGFP- HvPAA1-F	CTCAGGTACCATGTCTTGTGGGGGATGTGCA
35SGFP- HvPAA1-R	CTAGTCTAGAGGTACTCCTCCATTTTGATGG
HvPAA1-γ-F	GTACGCTAGCTGCAGTGCTGGTTGGTACTT
HvPAA1-γ-R	GTACGCTAGCCTCCGGCAGCCAAACTAAGA
PDS-γ-F	GTACGCTAGCCGACGAGGTTTTTATTGC
PDS-γ-R	GTACGCTAGCAGTTATTTGAGTCCCGTC
PDS- RT-PCR -F	AGTCTTTGGGTGGTGAGGTC
PDS- RT-PCR -R	CTTGAAGATATCGACTGGTG
Actin-F	TGGCTGACGGTGAGGACA
Actin-R	CGAGGGCGACCAACTATG
HvPAA-full-F	ATGCTCGCCATTTTCCCTTG
HvPAA-full-R	CAAAATGGAGGAGTACCTGA

Table S2 The sequences of SNP markers used in the genetic linkage map

Marker_ID	Chr.	cM	Barley_Contig	Sequence
TP61179	1H	0.00	bowman_contig_189321	TGCAGTCTGCACAC[A/G]TATCATTTGTCCCAGTCGG CATTTTTCTTTATTTAATGGAAGTATCTAA
TP19801	1H	0.00	morex_contig_49842	TGCAGC[A/G]TGGCCCTAGGACCTGGACAGAAGGAA TTTATGAAACCGTTGTGCTTTCACGGCATCA
TP47182	1H	0.00	bowman_contig_1987022	TGCAGGCGCGCCGCGGCCAAG[A/G]CGGCGCCGAG CCCGAGCGTGTGCACGCAGCTGTAGGCGGCCT
TP47659	1H	0.00	barke_contig_307038	TGCAGG[C/T]GGCAAAAGCGCGGAAGAGGCGATGC CAGCATCTGAGGACGATCTTGTGCTGGGCATC
TP50412	1H	0.00	-	TGCAGGGCAGGCCACCT[A/T]GGCGGACTAGGCTCC AATCCGAGATCGGAAGAGCACACGTCTGAAC
TP53592	1H	0.00	-	TGCAGGTACCTGTTCCTCATAATCTACGGTGC[G/A]A TCACGCCGTCGTTCTCGACCACATTCTTCA
TP61410	1H	0.00	morex_contig_59652	TGCAGTGAAGC[A/G]ATTGAAGAAGCCTCCTCCTCG CCTAGGAGGAGGAGGCAGAGGATGATGCTCA
TP62315	1H	0.00	barke_contig_53331	TGCAGTGCGCCATGTTGGACTCCAGCAGCAGCCCG AGAAGGTCGTCGCTCGTGGCCTC[A/G]CCGAG
TP61180	1H	0.00	barke_contig_203110	TGCAGTCTGCACACATATCATTTGTGGGAGTCGGCA TTTTTCTTTATTTAAT[G/T]GAAGTCCCAGA
TP22233	1H	1.30	morex_contig_40797	TGCAGCCC[C/G]AGCACACCACACCTCACATGGTTT GCGTCCGCTCACATCAGATTGATCAATTGCA
TP10212	1H	1.30	morex_contig_399750	TGCAGATGAGCTCTTGGCAGGAAAAGACCAGCCCA TGCAACTGGACGGGCATCATGTGCA[C/T]GGC
TP46664	1H	1.30	barke_contig_2779450	TGCAGGCGATAAGCAAAGCGGCAA[C/T]GCTGGGG AGGCAAGGAGTTGGCTTGAGCAGCGTCCCAGT
TP1818	1H	2.60	barke_contig_502131	TGCAGAAGAAGGAGGCGACGATGCGCGATGAGCTC A[C/G]CAAGAGGGCCAAGGAGCTGCACGACAA
TP28289	1H	2.60	-	TGCAGCGCGACG[A/G]CAGGAGCTTTACGGCCATCT GAAGCCCCGAGATCGGAAGAGCACACGTCTG
TP29168	1H	2.60	barke_contig_1567676	TGCAGCGCTCCAATTCCACGCTTGGTTTTCTTCGAA CACGTCGTCGCGCGAAATCC[A/G]TAGATGA
TP38479	1H	2.60	-	TGCAGCTTTTCTGCTTTGTTCTC[A/G]TCTTTAGTTAA ACAGCTCATTAGCTCAACTAGCTACCTGA
TP57775	1H	2.60	barke_contig_283709	TGCAGGTTTGTCACTTTCAGAGACCCA[C/G]AGCAA TCAGACATTTATTCCCTGCCCTTGTTTCTGT
TP63416	1H	2.60	barke_contig_265240	TGCAGTGTCGGGT[C/G]GCATGAATGGTTCAAGGGT ATATGAGCGGTCCGAGATCGGAAGAGCACAC
TP10338	1H	3.90	barke_contig_400447	TGCAGATGCACCCCATTCCTCCTCCTGTACACATCC ACCTTGGA[C/T]TCCGCAGCGACAACCACAT
TP30056	1H	3.90	morex_contig_267887	TGCAGCGGCCCGCG[G/T]TGATCCATCGGCGAACTG GTTGTCGTCGGTCCGAGATCGGAAGAGCACA
TP4623	1H	5.17	morex_contig_55791	TGCAGACCTTGTCGAACTCCTTGACGGCGAGAG[C/T

Marker_ID	Chr.	cM	Barley_Contig	Sequence
				]GCAGTCCTCCTTCTCGAGCAGCGCGCACCC
TP26717	1H	5.17	morex_contig_29228	TGCAGCGCAAAACCCGAAACGGCAAGATCGAGAAACGTCAGTACAGCCGCGCTGCAC[G/T]CATGGG
TP12164	1H	6.50	bowman_contig_870099	TGCAGCAACACGCAGTGGTTTTGACCGTTGTCAACAAGCTGCAT[A/G]CCGCCAGCCCGCTTTCCGT
TP34315	1H	6.51	bowman_contig_65671	TGCAGCTCGAAGGTAGACACGCGGCCGCAGTGCCAGATCTGGCAGAAGAAGAG[A/C]GCGCCCTTGG
TP15481	1H	9.11	morex_contig_45711	TGCAGCAGACCGACAATCGAGAAATCAAGCGAGGAAG[A/G]AAGAAAGAAAGATCGGCGGGGGATGA
TP36838	1H	10.34	barke_contig_481914	TGCAGCTGGGCTTCAATTTCTTTGAGCTTGGCT[A/G]CCAGACGCCGCCAGGTCTTGATATGGTTGA
TP32762	1H	10.34	bowman_contig_15037	TGCAGCTACGACAACTGCGGCAAGACCTTCGTCGA[C/T]GTGGCGGCCTTGAGGAAGCACGCCCACG
TP50496	1H	10.34	morex_contig_8312	TGCAGGGCATGCCAAGTTTCT[C/G]CTAGCTTCCCTTTGCTTTGCTTGCAGCCGAGATCGGAAGAGC
TP3585	1H	10.34	bowman_contig_862606	TGCAGACAATTTTTACCGCTATCAC[C/T]TCCTGTCACTCCGAGATCGGAAGAGCACACGTCTGAAC
TP34797	1H	12.94	morex_contig_56567	TGCAGCTCGGT[C/T]GGCGAGAGCGCGAACATGTCCTCGTGGCAAGTGTGTATCGCCCGAGATCGGA
TP64276	1H	12.94	morex_contig_354257	TGCAGTTCCCCTGCA[C/T]CTAGCGTCGTCGATGGCGTAGAAAGCTCTAGCCGCATGCCCCTCCTAT
TP1116	1H	12.94	bowman_contig_143159	TGCAGAACATCATCGGTGAGGG[A/C]GGCAAGGAGATAGCTGAGGACGATCTGGTCCTGCCGAGATC
TP1933	1H	15.55	barke_contig_278762	TGCAGAAGAGGCTTTTGTGGGAAAAGGAATTTATCTCAAGAGGCTCGCTTACCA[C/T]GCCAAAGGG
TP2966	1H	18.16	barke_contig_299617	TGCAGAATCACCTTCTTGGCCTCCTCCACCGATTTCCCCAC[C/T]AACTCTGGCCACTCTGTCTTTT
TP50214	1H	18.16	morex_contig_244029	TGCAGGGCAACCTGCTCTCCCTTCGGACGTTTGTGTGGGTTCAAATCTATTGTGGTGGAGC[C/G]AG
TP36459	1H	19.46	morex_contig_1562664	TGCAG[C/T]TGGAAGTAGTTGACCACAGAAGGAGGGCGACTGGATCTGGGAGGACGACGGCGCGAAC
TP38885	1H	19.46	bowman_contig_359512	TGCAGGAACAACGTCCTGAAACAATGGGATTGTT[G/T]CAGGAAAACGGTCACGGCTCGTTCACGGA
TP5886	1H	20.76	bowman_contig_69020	TGCAGAGAATGTTGGGA[A/G]AATCAGCTTCTGTATTTTTAAACAGTTAGTCACTTTGATCTTTTAA
TP24110	1H	20.76	morex_contig_18091	TGCAGCCGCTGGACCGACGTTAGTTACGACG[C/T]GAGGCCGCTGGACCGACGTCATGTTCGACGCG
TP59468	1H	20.76	morex_contig_48651	TGCAGTCAAAT[A/G]AATTAGTAACATCATTCAGAATATCAAGGGTTCTTCACAAGATAACTAGACT
TP44354	1H	20.76	barke_contig_62011	TGCAGGCAGGCGGTGGTATGAGAAAGATTGTGCAGCAGGCAACATTGTTT[A/G]CAGCATCAAAGGG
TP34976	1H	20.76	morex_contig_136648	TGCAGCTCTAGAACTGCTAACCCT[C/G]CAAGAATAACAAGCATGTAAGCCTGTTTGGATCAGCTTT

Marker_ID	Chr.	cM	Barley_Contig	Sequence
TP35187	1H	20.76	morex_contig_66295	TGCAGCTCTTCAATAAAA[A/G]GAGGATTTTCTCTGT GAGATGCTCCCAGGAGAGCTTCATTGTTCA
TP49749	1H	20.76	barke_contig_74245	TGCAGGGAGA[A/G]GAGAGCTACATCGATGAGGAG ACGAATCTGGTGTACGTCTCGGACGCTGGCTT
TP12394	1H	23.36	morex_contig_73045	TGCAGCAACGCACGGGGTACAATTTTCTGAAAAAC AGCACTACG[A/G]TGTGAGACCTTTTCACGTT
TP34219	1H	23.36	-	TGCAGCTCCTGCCATGTGATAACCAGCGCAAGGA[C/ T]TAACCGATGAACAAGGATTTCTACATCAC
TP44205	1H	23.36	bowman_contig_221886	TGCAGGCAGCCATGGACGGTCACAACAGCCGC[G/C] CGCAGCGCCTGATTCTGGCGCTCGAGGAGTA
TP38655	1H	23.36	bowman_contig_221886	TGCAGGAAA[C/G]ATCGTGTATACGTAGTATGTATG AACCCGCGCACCAGATATTATTACCAATAGT
TP42791	1H	25.96	morex_contig_1577249	TGCAGGATCACAAGGAC[A/G]GGGTTTCAGAGGCCT GTTCTCCGAGATCGGAAGAGCACACGTCTGA
TP27062	1H	25.96	bowman_contig_936678	TGCAGCGC[A/G]GTGGCGCGGGTCACCCGTTCGACG GATTGCCTGAACCAAGTCGTCCCCACCGCCG
TP33282	1H	25.96	bowman_contig_179967	TGCAGCTATTTGATCAGTGGC[G/T]GCCCAAGCTTCC GCAAAGATTGAGTGTGGTGAACCAGACACG
TP63474	1H	25.96	morex_contig_1803195	TGCAGTGTGAGGTCCAGTTTTGAATTCCATGCCCGT GGTGCTTGTCGGAGCCCGTAC[A/T]GTGCCT
TP18065	1H	27.30	morex_contig_1565287	TGCAGC[A/G]GGGAGATCTCCAACTACATGGTGTAC CTTATGTTCGTGAACCCGAGATCGGAAGAGC
TP30032	1H	27.30	-	TGCAGCGGCCATGGACGTAG[C/G]CGCGGGCCTGGC AACACTGCATGCACCAGCTCGTCCCCCCGAG
TP55331	1H	33.90	-	TGCAGGTCTGGCCACA[A/G]AAGTTCCCGAAGCATC CAGTTCTGAAAAAAAAAAAAAAAAAAAAAAA
TP9596	1H	33.90	barke_contig_1862685	TGCAGATCGCAAGTGACACTGCTG[A/G]CCACAACG CCGAGGTCATCCACAACCTAGCCAAGGACGT
TP479	1H	33.90	-	TGCAGAAAGAAGAAATAACCTGCTTAT[A/G]AAAAT ACTTGTGCTATTACGGGCTCAACTTCCCGAG
TP29157	1H	33.90	morex_contig_2551522	TGCAGCGCTCAACCTTAACCTACTGCCCACCGAGGA ATTAATAACGATATTAAACGCAAAAGG[A/G]
TP21188	1H	33.90	barke_contig_281740	TGCAGCCAGAGCGTGCGGTAGA[C/T]GAGGTCCGAC TTGAAGTACTCGGGGCAGACGAAGCCGACGC
TP5319	1H	35.21	bowman_contig_69116	TGCAGACGTAACTGAATCTAAC[A/C]AGAGGAGGCC GAGCATGGGGAGAAGAAGAAGGGGACGACGA
TP9441	1H	35.21	bowman_contig_1984215	TGCAGATCCTTGTAGTGTACTTGTTCCCAGTGTATT ACTTCTGAACCCAGAGTT[G/T]CCGAGATCG
TP9643	1H	35.21	bowman_contig_981756	TGCAGATCGCGAATGACACTGCAATATGCGCCAGA GGTCGGGGT[A/T]ATCCCCTTTTCGAAAGAAA
TP52522	1H	35.21	bowman_contig_1984215	TGCAGGGGTTTCCCTCTATATATATGCTCGAACTC[A /G]CTCTCCACTTCTTCCCGCTTCCTTTATC
TP59356	1H	35.21	bowman_contig_981756	TGCAGTATGTTGTTTGCATCCCCTGGGACC[A/G]AA

Marker_ID	Chr.	cM	Barley_Contig	Sequence
				ATGTTGTGAAGTTTTGTGGACAGTCTTCCGA
TP61978	1H	35.21	barke_contig_269875	TGCAGTGCAGCATCAACGAGGAATTGAAGCAGATT GCACCTGCATTATTTACCC[C/G]AAATTGCTT
TP23784	1H	37.88	bowman_contig_75783	TGCAGCCGCCTGGTTAGATCCGACCAGCTGCCTCTC CCTGCGCTGCTTGCA[C/T]CTGTCCCAATCG
GST6	1H	38.4		GTTTTCTTGTTGGTGTCGCGAATAA[G/A]GCAATATA AGCTTGCGCGCTGG
TP37473	1H	39.12	bowman_contig_866157	TGCAGCTTCATCCAGGCCAGCTTCGCCCA[C/T]GCGC GGAACGCGTCCAGCCGAGATCGGAAGAGCA
TP31457	1H	40.41	barke_contig_139843	TGCAGCGTATACAGTCAAGCG[C/T]GGCAGGTCAGC CACGCCGACGGAGCCACTTCGACGCAGCGCG
TP49632	1H	40.41	barke_contig_2784595	TGCAGGGACGA[C/T]CCCACAGCAAACCGAAGCACG AACCTGTCACCAACCACCGTGTGTGCTAGAT
TP11932	1H	40.41	morex_contig_1592615	TGCAGCAAAGCTGTGCC[A/T]CGTGACCGTGACCAT GGTGATGCTGCCTTAGCTGTGTCCAACATTT
TP20924	1H	40.41	morex_contig_9427	TGCAG[C/G]CACGCGCCAACCCAGCCAGACCCCGTC GCGCTGGCGGCTGCTGCCGAGATCGGAAGAG
TP27970	1H	40.41	bowman_contig_68763	TGCAGCGCCTCCATCACTCCTGGCCAGCGTGTGTCC TCCTC[A/G]GGCAGAGACTCCTCTAGGGCAC
TP37070	1H	40.41	bowman_contig_68763	TGCAGCTGTCAACGACGGGGGACTGCGTCTACCCTC TGCCAT[C/T]GTCATCGGCCATGCCCAAGGT
TP42121	1H	40.41	bowman_contig_14866	TGCAGGAGGC[A/G]TCGTGCGGCGCGGTGCGCGCCA TCTTCCGCCGCCTGCTCGCCTGCACCACCAC
TP54100	1H	40.41	morex_contig_1592615	TGCAGGTCAACATGAGCCTTCTCCTTGCCTTGGAGC TGCTGCTCTTCCTGCT[C/T]GCCTTGCCATG
TP21508	1H	43.01	bowman_contig_74732	TGCAGCCAGGTGTAAGACGAGAGATGGATCATAGT CGTGGGAGGGCCGAACCTA[C/T]GATGAAGGT
TP29579	1H	43.01	barke_contig_473228	TGCAGCGGACAAGGAGACGCATCAGACGGTTCCGC GTAATCCGCTCGATTTGGA[A/G]AGACCGCGC
TP9867	1H	45.61	morex_contig_62731	TGCAGATCGTGGTGATGAACATGCTCATGGGGGCCT CCACCGTCAGCAGCATCCTCTCCAC[C/T]TA
TP19950	1H	45.61	bowman_contig_125412	TGCAGCATGTGCCGCGGCGTGTCGCGTCCC[G/T]CTC ACTTTTCTCTCTCTCCCTCGCGAAGCGCGG
TP23190	1H	45.61	bowman_contig_125412	TGCAGCCGACCTAGCTCCTCGCCCGTGCCGTGCTCT CGATCAGCGTATAGCTACGGTACTTGT[A/T]
TP35045	1H	45.61	bowman_contig_66662	TGCAGCTCTC[C/G]GTCATCGGCTTCGTGCTCCACTT CATCTTCACCCAGAGCAGCCCGCTCTGGAT
TP36585	1H	45.61	morex_contig_62731	TGCAGCTGGATGGCCTCCAGCGGCGACGCGCCGCC CATGATGAGCCCCGTCAT[G/T]GCCCCCGAGA
TP41556	1H	45.61	bowman_contig_221115	TGCAGGAGCAGCAAGACAGAAGGATGCCCTCTTGT GTTTTTGT[G/T]TCTGTCAAGTTATTGCACCA
TP46652	1H	45.61	barke_contig_278060	TGCAGGCGAGTAGC[A/G]GAAGTTGTGTCTTCGAAG GCCGCCGCCCAAGTCCGAGATCGGAAGAGCA

Marker_ID	Chr.	cM	Barley_Contig	Sequence
TP60075	1H	45.61	morex_contig_2125372	TGCAGT[C/T]CATTCATGCGCACTGCCCACTCGGTGG TCGGCCACCTGGTATGGTAAACACGCAGAG
TP1994	1H	48.21	morex_contig_150418	TGCAGAAGATGCGGTGCCATCTTCAAGTAATTTCTT GATTTCTTTTA[A/G]TTATGTATTGCTACCT
TP3297	1H	48.21	morex_contig_43334	TGCAGAATTAGGTAC[C/T]TGCAAAGGGACTAGAGA TTGGACTGTTGAGTGGAATTCGGGATGGAGA
TP25814	1H	48.21	barke_contig_275901	TGCAGCGACCATTCCTGCCTGGTGCTGAAGCCTGAA AGCAAAGGCACA[A/G]ATCTTTTGAGCAACC
TP4664	1H	58.76	barke_contig_2764424	TGCAGA[C/G]GACAACAGCAAGATTGGTCAGAGAC ATTGCTCTCCTCTCCTTGAAAACGGAACCCGC
TP11036	1H	58.76	morex_contig_109883	TGCAGATGTGGCCCC[A/G]TTGAAGCCTTCTGAAGG AACTGGGTCTCAAATTGATGCCCACTGTCCG
TP36538	1H	58.76	barke_contig_372999	TGCAGCTGGAGGTGAGCAGAGCA[C/T]CCTTGAAGC AGACATCGTTCTTGTCTCTGCTGGGAGAACC
TP6483	1H	60.06	morex_contig_162003	TGCAGAGCAGCCTTTGGCCGCTGCCCAGCGCGAAG GT[C/G]TTCGGAGCCGCGCAGCCGCTGGTGAG
TP24641	1H	60.06	morex_contig_364809	TGCAGCCTCCAATGCCGTTGTCGCGC[G/T]CTGCACG ACATCCTCACCACCGTGGACCGAGATCGGA
TP43383	1H	60.06	barke_contig_372234	TGCAGGATGGTTGAGCAAGATATATGCG[G/T]CATC AACTGGCAAGCTAGGGGTGGTGGCGACATTG
TP24646	1H	60.06	-	TGCAGCCTCCACAGCCGCTGCCGCGCGTTGCACAAC ATCCTC[A/G]CCACCGTGGACCGAGATCGGA
TP38588	1H	61.35	bowman_contig_221126	TGCAGGAAAAGAGTCTGTTGAGCTTATTTTTTGTGG GAGCATCCGTCTCAATGC[A/C]GCAGACGGT
TP2554	1H	61.35	bowman_contig_1992394	TGCAGAAGGCGCCGAACCCGTGGGCTTTCTCGAGC AG[C/G]GACACCACGCAGGCCACACGGAGCAC
TP6304	1H	61.35	bowman_contig_860772	TGCAGAGATTCTACGACCCTGACGCAGGCCACATA CTTCTGGACGGGGTGGACATCCAGAA[A/G]TT
TP8811	1H	61.35	morex_contig_54738	TGCAGATATATCTCATGCATGCATGCACATCACCCCT GCCTTG[C/T]CCGAGATCGGAAGAGCACACG
TP11014	1H	61.35	morex_contig_63726	TGCAGATGTGACACTCCCATGCAGCATGCATGTAGT AGCTGGAGACGTGTTGC[A/G]TGCACGATCT
TP26803	1H	61.35	barke_contig_1855624	TGCAGCGCAATATTTCGTTCCGCAAA[A/T]CCTCATT TTATCGAACTCTAAAACGCGTTTGCAGGTC
TP41354	1H	61.35	morex_contig_1561831	TGCAGGAGAGGGTGACACGGTGGTACATGCAGTCC CTGGCCTCGTGTGCGTCAAAGGG[A/G]GTCGG
TP42558	1H	61.35	bowman_contig_845043	TGCAGGAGTGCCGCGCGCG[C/T]GTCTAATTTTTGG ACTTCCGCTTGAGCCAGCATTCACCGAGATC
TP45843	1H	61.35	barke_contig_259752	TGCAGGCCGCATCCACAGTTTCCGTGTGTTGTGGTC CGTCTCCGCCGATGCGCCATCACC[A/G]CCA
TP64197	1H	61.35	barke_contig_128881	TGCAGTTCATTGCGCGTTAAAGGTAGACAACTATCC TGTAGCACGTGGAGTTG[A/G]TCCAGTTGGC
TP65860	1H	61.35	morex_contig_51489	TGCAGTTTGCATGAAATCAATCGGCTCGGAACTT[C/

Marker_ID	Chr.	cM	Barley_Contig	Sequence
				T]ACCAGAGGTCCTGGAGTGGAGGGTAATAA
TP171	1H	62.65	-	TGCAGAAAAGCGATAGGACTGAACTGTACCAGAAAGA[C/T]GGGGCCGCATTATTCCGAGATCGGAA
TP396	1H	62.65	bowman_contig_866236	TGCAGAAACGCGTTCGCAGCCCATGAAGCACGC[C/T]AGGGACACGCCTATGTGCGAAAGGGAAGCA
TP657	1H	62.65	bowman_contig_175506	TGCAGAAAGTCGCACAAAACAT[A/G]GCGAAAATATCCTAGATTTCTCCAACGAGCTGTGCGCTTGA
TP2066	1H	62.65	morex_contig_127935	TGCAGAAGCAGAAGACAGCAAAAAATA[A/G]ATTAAGAATCGCAACTCATCATACATATCTAACTGG
TP3776	1H	62.65	bowman_contig_64794	TGCAGACAGCTGCTAATTCCATAATACGGTCGACGCGAAATTGAAGCTAGAGC[A/G]GCGGGATCTA
TP4369	1H	62.65	barke_contig_470676	TGCAGACCGCCGATCTCCTACGAGAATC[C/T]TGCCTCGAAGAATCGGCCATGGTGGACGTGTGGAC
TP5082	1H	62.65	morex_contig_42420	TGCAG[A/G]CGCTTACAAGAAAGCAGGCAATACATGCGAGTTCAAAAACACTCTATCAAGAATAATA
TP5688	1H	62.65	bowman_contig_873074	TGCAGACTTCACATGGAGA[C/T]CTAGGGCCCCATTGCAATACCATTTCTTTGTCTGTCTAGCAATG
TP6281	1H	62.65	-	TGCAGAGATGGTACGTAGTAGCATGTGTTTGTTTTTGTGTGTGTGACCGTTGATGTTGATGG[A/G]A
TP12512	1H	62.65	barke_contig_60431	TGCAGCAACTCCGACAACCCTC[A/T]CACCCATCAAGTTTCAGAGTCACTCCTGTTCATGCACGGCT
TP15641	1H	62.65	-	TGCAGC[A/G]GAGCTTCACCGTCCGCTCCGAGTGCTGCAATGAAACGATCGCCGAGATCGGAAGAGC
TP16101	1H	62.65	-	TGCAGCAGCACTACACGTACACGATGACCACCGCCGAGCCTCCC[A/G]TTCCGCCGTCGCCAACCAG
TP18994	1H	62.65	barke_contig_53378	TGCAGCATATCTCAAACCTCCCC[G/T]AAATTAAGTGGATTACCCCTACAATAACACGATATCTTGG
TP22889	1H	62.65	barke_contig_66015	TGCAGCCCTAGATTTGGAACGACGTCTGATAGGCTCTTCTTCCCTCTCTCTGT[C/T]TCTGTTTATG
TP26582	1H	62.65	-	TGCAG[C/T]GATGAAGAACAGCATCGCGCCGACAACAGAAAGTGCGACAGCAAGGATCAGCCTGATG
TP27107	1H	62.65	-	TGCAGCGCATCCCACTCCCTCAAGAAGCCGCCGCAGCATGCCCG[C/T]GTGTCTCCACCGAATCTCC
TP28826	1H	62.65	morex_contig_222866	TGCAGCGCGGCTCTTTGGCGGCGCTGATGGT[C/G]TTCTAGCCAGTGTGGCGAGGATGGCCGAGATC
TP29586	1H	62.65	barke_contig_2623376	TGCAGCGG[A/C]CAGCGGGGTGCGGGCGACGTCTCTGTACGAGCGCGCTGAAGACTCACTCTCCGAG
TP33980	1H	62.65	morex_contig_40685	TGCAGCTCCGAGTTGCC[C/T]TCGGCCTCTAGGCAAAAGAGCCTACCGACCAGCCTCTTTTTGGCTT
TP34769	1H	62.65	bowman_contig_937520	TGCAGCTCGGGGCTGCGTCATTCGGACTCTCGGCAAACCTGCATGGATTC[A/C]AGAGTGACAAATT
TP34909	1H	62.65	morex_contig_66382	TGCAGCTCGTGCGCGTTGACCGCA[C/T]GACTCGCCAATGGCCTAGCCCTATTCTCGTGCCTACGTA

Marker_ID	Chr.	cM	Barley_Contig	Sequence
TP41370	1H	62.65	bowman_contig_222294	TGCAGGAGAGTTCTTCTTCTTGGTATCCATATAGTC TACTG[A/C]TGAAGCTCGTTTGTCGCCAGGT
TP41496	1H	62.65	morex_contig_1566127	TGCAGGAGCA[A/G]GCACGGATTGTGGAGGCTGTAT AGCAGCTCCTGCTCGCAATGTTGTCTCCAGC
TP46106	1H	62.65	morex_contig_41864	TGCAGGCCGTCGACCGCTCCCTCCAGTCCTC[A/C]CA CCAGAGTACGAACCCTCTCTAGCCTTCCTC
TP46662	1H	62.65	-	TGCAGG[C/G]GAGTTGCAGTCGGCCGCACCGCCGCC GACGACGCCGCCGCAGTGCAAGAGGTTGCAC
TP49107	1H	62.65	barke_contig_57281	TGCAGGCTGGTGAGATGCGTAAGGTGAACCACGTC TACTTC[C/T]TTCTCTTTACTCTCGAGTGATT
TP51206	1H	62.65	-	TGCAGGG[C/T]GGCCGACCGAAGAGGCGTTCATGAG GTGGTGCTCGTGCTCGGCGAATCAAGGAGTT
TP53054	1H	62.65	bowman_contig_175506	TGCAGGGTGGGAAGAGGAAAT[A/G]GGAGTCTGGG ATATTTATCTCCGAGATCGGAAGAGCACACGT
TP56630	1H	62.65	morex_contig_41639	TGCAGGTGGTGGGCGCCCCCGCCCGCCGCGGCGCC ACGG[A/T]CCGAGATCGGAAGAGCACACGTCT
TP57575	1H	62.65	-	TGCAGGTTGGTGATGAAGGACTGGCCGAAACCCCA GTTGGGCGGCACGACGTT[A/G]TGGAAGACGA
TP58568	1H	62.65	bowman_contig_64463	TGCAGTACTA[C/T]TGCGTGTTCGCGGCGGTGGGCG TGGCGGTGATCGTGCTCTCGCTCACGTTCCT
TP59425	1H	62.65	-	TGCAGTATTTTTTTCT[A/G]TTGGTATGAGATACAAT GGTTTGCATAGCCCACGTGCACTTGCTAGC
TP62354	1H	62.65	-	TGCAGTGCGGATGCAATTTGAGCTTCCCTCCACTCT CTTTGACGCAAGTTTGCAAGCGTCG[A/T]GT
TP63459	1H	62.65	-	TGCAGTGTGACGCCAGAGCGCTTAGGCGTC[A/G]CA CACGCACCTATGCATTATGCAGCCCGCGGCC
TP64199	1H	62.65	-	TGCAGTTCATTTGAG[A/C]CCTATATGTGTGCTTATC AACAAGTCATTGTACGTAGAAATCCCATTT
TP65577	1H	62.65	morex_contig_42584	TGCAGTTTACCTTTCGCAGAA[C/T]ACTGCTTGCTGG AGCAGTAGGCTCAACCCGAGATCGGAAGAG
TP65627	1H	62.65	morex_contig_46307	TGCAGTTTATTT[C/T]GAGAAGTACATTTATGTTTCC AATGATCTTGTTGCACAAGGAAATCACATC
TP66015	1H	62.65	barke_contig_1854775	TGCAGTTTTCACT[A/G]TGGAGGTCGAGAACCTCAA GGCCGAGATCGGAAGAGCACACGTCTGAACT
TP3549	1H	71.85	-	TGCAGACAAGGTTGAAGCGT[A/C]ACGAATGGCTTA ATCTTCTTTAGTTCTTTTCACTTCCAACTGT
TP3913	1H	71.85	barke_contig_1870849	TGCAGACATGGAGAGCTGGGCGGCAGC[A/G]GAGA TGAGCCAGACGGGCAGGGGCAGTGGCAACGCG
TP16728	1H	71.85	bowman_contig_64749	TGCAGCAGCGACGAGAGCTGCCGCATTCTCGCTA[A/ G]CCCAAAGAAACGGCCCGAAAGAATCGGGC
TP37609	1H	71.85	morex_contig_1564278	TGCAGCTTCGAGGCGTACCAGGACAC[A/G]CTCTAC ACGCACTCCCTCCGCCAGTTCTACCGCGGCT
TP45846	1H	71.85	morex_contig_1564278	TGCAGGCCGC[A/G]TCGGGGTCGTGGTCCGACCGTG

Marker_ID	Chr.	cM	Barley_Contig	Sequence
				GAGGCGGCCTCGCCGAGCCGATCTCCAACGG
TP30760	1H	73.15	bowman_contig_217	TGCAGCGGGAGGTCGAGGAAAGGTGAAGCGAGTTT GGTAAAAAAAAGAGGCGGG[A/G]CCGAGATGA
TP54384	1H	73.15	morex_contig_2553349	TGCAGGTCCAACCTTTTGGAAATATCACGTAGTCAG ACATGTGAGAAAGATTGACTCT[C/T]AAGTC
TP61820	1H	73.15	morex_contig_48566	TGCAGTGATTTCTCTTTTCATCAGAAGAATAAAAGA ACCAATTTAGC[A/G]CAGTGTTTGTCTTTTC
TP35949	1H	74.44	-	TGCAGCTGCGAGATTGGATCGGC[A/T]AGCACTGCG GACACCCGAAAAAAAAAAAAAAAAAAAAAA
TP35950	1H	74.44	-	TGCAGCTGCGAGATTGGATCGGC[A/T]AGCACTGCG GACACCCGAGATCGGAAGAGCACACGTCTGA
TP38672	1H	74.44	bowman_contig_1025830	TGCAGGAAACCCTTTTAAAATATTTCTGAACCTATC ATTATTTGTGGAAAAAATGGTTTCAC[C/T]G
TP45857	1H	74.44	-	TGCAGGCCGCCACAGCCGCCGCGAG[A/G]CCCGTTC ACGCCGAGATCGGAAGAGCACACGTCTGAAC
TP46129	1H	74.44	bowman_contig_125597	TGCAGGCCGTCGGCGCACGTCTGCTGGTTGGTCAGG ATCGCCGACAGCAGCGTCTGCAC[A/G]TCCT
TP22507	1H	77.04	barke_contig_316890	TGCAGCCCCTGAAGAAGGATGC[A/G]TCGCAAGTTA AGCGAAGCAAGGTCGAAATAATCAAGGAGAA
TP43742	1H	77.04	barke_contig_2782439	TGCAGGCAAGAAGAAAG[A/G]TGCAAATGTGTAATC CCTCCGAGATCGGAAGAGCACACGTCTGAAC
TP38555	1H	82.49	bowman_contig_870465	TGCAGGAAAACAACATAGTGGAGCGTGTCAAGCAC AATGTA[A/G]AAGAGTACAAGTACATAACTGA
TP26093	1H	83.50	bowman_contig_872002	TGCAGCGACTTCGTTCGTTACATAAAT[C/T]TGATTA CCGCCGACCTGTGGGCAACTCGAGTTCTAG
TP6496	1H	86.06	bowman_contig_877463	TGCAGAGCAGCTCAGGAACGACGGTCCTGCC[C/G]C CGCGACGGCTTCTGCGACCGACTGTCGCAGT
TP33920	1H	86.06	barke_contig_126614	TGCAGCTCCCGCCGTGGCACCGCCTGCAAGGAGAG GAACCTGTCGA[C/T]GGCGTACATTGTGAGGT
ATPaseB	1H	106.2		AATATTTGGCATATGAATGTGGGAA[G/A]CACGTTC TTGTCATCTTGACAGATA
TP42438	1H	122.98	bowman_contig_859985	TGCAGGAGTACAA[A/G]GCACACGCGGACAGCTTCA TCTGCTCCATGGTGCCCGAGATCGGAAGAGC
TP57570	1H	122.98	morex_contig_1579985	TGCAGGTTGGT[C/T]GTTCCTGGGGTTGATCACACAC CATAATGTCAAGGAAGCGCTGGCCACAAAT
TP3857	1H	125.48	bowman_contig_891947	TGCAGACATATTCACAGTTGC[C/T]GATCCTCTCGAT GGGAGCACCGAGATCGGAAGAGCACACGTC
TP24627	1H	125.48	bowman_contig_22565	TGCAGCCTCATCGAGATCGACGGCGTGGT[G/T]CAC GAGTTCCAAGCCGTTCCGAGATCGGAAGAGC
TP1598	1H	130.69	morex_contig_140117	TGCAGAACTAGCCAACTGCTTGAAAGGTGTGGATG T[C/T]GTTGTCATCCCTGCCGAGATCGGAAGA
TP32456	1H	130.69	morex_contig_136849	TGCAGCGTTTAATAGGAAACGCCTATCACGCCCTTC TTCAGATCGACCGTG[G/T]TCTCTTTTTAGT

Marker_ID	Chr.	cM	Barley_Contig	Sequence
TP8644	1H	130.69	morex_contig_51045	TGCAGATACTGGAAGTGGCGGAACAG[C/G]TCGTCG TCTCCCTCCTGCGAGTTGTCCATCACCGAGA
TP5903	1H	148.28	morex_contig_1580847	TGCAGAGACACAAGACAAACTATAGGAATCAGCAG GCAACACGAAGCAAGCAAACAGCAA[C/G]CGA
TP22155	1H	148.28	morex_contig_46243	TGCAGCCCATGGATCGGTGGAATCATGGATC[C/T]C TCCTCCAACTGGAACAATCAAGATCAATGTT
TP29113	1H	148.28	morex_contig_1593140	TGCAGCGCTACCACGGTGCCGCTCTTGCACTACCTT GTTGGTCGATATC[A/G]TCTTTGTCCTGCCG
TP42450	1H	148.28	morex_contig_6024	TGCAGGAGTA[C/T]GGCCATGTGGCCGACGCCAACG TCTTCTACTGGTTCCAGAACCGCAAGTCCCG
TP11704	1H	148.28	bowman_contig_66072	TGCAGATTTTCTTGAAGCAAGTAAACCTGTTTTTT A[A/G]TTGACAACATGGGAGCATGTAAGAGC
TP18600	1H	148.28	morex_contig_1559731	TGCAGCAGTCCCCTCCTTCATGGGGCCAAAGAGCTC CGCCATAAA[C/G]ACGGCGGAACGGGACGCG
TP24995	1H	148.28	morex_contig_1572401	TGCAGCCTGAGGCATGCACCACCATGTTC[C/T]AGC GAGAAAAACACATCTCAGGCCAATAAACCTC
TP32335	1H	148.28	morex_contig_266577	TGCAGCGTTAACAGAAAAGCAGCAGCTAGTG[C/G]T AGTGCAGTACAAGAGCCGTATACATTCCACT
TP65127	1H	148.28	bowman_contig_67492	TGCAGTTGCTAGATTGTTGGTTGTGATGGATTGGGG GCTGT[C/T]CCTTGTCCGAGATCGGAAGAGC
TP38101	1H	150.87	-	TGCAGCTTGGAGCACCTTCCGATGGCCTTGAGTCCT T[C/G]GTTGGCAACGTCACAGCATGATTCAA
TP49735	1H	150.87	bowman_contig_143462	TGCAGGGACTTTCGCGTCGTACATGGTCGTGAG[C/T]GCGTTCACCCTCGTGTTCGTCATACTTTGG
TP62234	1H	157.74	barke_contig_118351	TGCAGTGCCTCGCCACGCTGGGTGTCTGCTCTTCCC TGCCGACCGACAGGTGCCATTTAAAAA[A/T]
TP13146	1H	158.73	morex_contig_66005	TGCAGCAATCTACTAC[C/T]AAAGTGCTTTTTTTGTC TTGGCCACGCAGGATGTCGGTGCCGAGATC
TP15948	1H	158.73	morex_contig_52827	TGCAGCAGCAACGTAATTAGATCAGAATGC[A/G]TT CATTCAGTCAAAATTGAATGCAAGAGGGAAG
TP28730	1H	158.73	barke_contig_8179	TGCAGCGCGGCATCGTGGCCACCAACCAGATCGCG CACAACGTGAC[G/T]TCCACGCTGCACATCAT
TP31745	1H	158.73	bowman_contig_898196	TGCAGCGTCGCCGTGGCTCCC[G/T]CGCCAAAGCTG GACCCTCCTGGCATGTGTGTAGCTCCAACAA
TP37580	1H	158.73	morex_contig_1570108	TGCAGCTTCCTCCCCTGCCAGGAGAACCGAGCCCAG CTCGATGCGCTC[A/G]GGATGGAGAAGGTCG
TP49410	1H	158.73	-	TGCAGGGAACCAGTAAACAAAATTGCTACAACCAT CAATGCAAAATGCTA[C/T]GACCGAGATCGGA
TP57997	1H	158.73	-	TGCAGTAAGACACCTACAGAAAAGAAAGGGTAAAG ACATGCTCACAT[G/T]AAAACCATATACGTAT
TP2157	1H	160.03	barke_contig_54302	TGCAGAAGCCAGACTC[A/T]ACTAAAGCTTTTGACT CATTCCTTGAGAAACGTAAAGCAGCTCCATC
TP2396	1H	160.03	barke_contig_1794516	TGCAGAAGCTGGTTACAGGAGCTGCACAC[A/G]ACG

Marker_ID	Chr.	cM	Barley_Contig	Sequence
				AACGGCGCGCCGCCCTTTGCCGAAAAAAAA
TP8588	1H	160.03	barke_contig_2804026	TGCAGATACGCGTCATGGATTGGGTTTGTTGGCATC ATATAATAGTACTAG[C/T]CGTCCTTTTTC
TP9267	1H	160.03	bowman_contig_13082	TGCAGATCCATGGTTCACTAAACACTAATCAAGTGA ATTCAAGAGCC[C/T]TTACAACCAAAGGTGG
TP19691	1H	160.03	morex_contig_44745	TGCAGCATGCCTTGGTTAGA[C/T]GACGATGCCGCT GAAGTCTCCTTCCCGAGATCGGAAGAGCACA
TP20072	1H	160.03	barke_contig_271782	TGCAGCATTGAGCAATGGAATTAGTATGTTTGATGA TTC[G/T]GTTTTGTGAAATGCATCTGAGATT
TP29744	1H	160.03	-	TGCAGCGGATGA[A/C]GCGAGGGGCAACCGTGGGG ACGGCGCCGAGATCGGAAGAGCACACGTCTGA
TP34720	1H	160.03	morex_contig_65576	TGCAGCTCGGCGTCGTCACGGCG[C/T]GTCCCCTGA CCGAGACCGTCTCTTCAGCACTCGGCTCGGG
TP35906	1H	160.03	barke_contig_373008	TGCAGCTGC[C/G]GTTTCATCTATCGATCGAATCAAT CCAGGAGACGAGGCAACATTGGCCCAAAAC
TP36658	1H	160.03	bowman_contig_883131	TGCAGCTGGCCGTCGAGGAGGC[C/G]GCCAAGCTGC TGGAAGGGGAGAAGCTGGAGATCGTGGACTT
TP36982	1H	160.03	morex_contig_156695	TGCAGCTGGTTAACCAGAATAGTAGTTAGCTGGT[A/ C]AGCAATATACTACTCCATCAGGCAATTAT
TP38830	1H	160.03	morex_contig_48809	TGCAGGAAATCGTCGTCGCTGTA[C/G]CCGTCCTTG AGCCTCTCGTTGAGCACCGTCACGAACCCCG
TP44274	1H	160.03	bowman_contig_116691	TGCAGGCAGCTGATGGCGCTCGTGTTCGACTTGGTG GCGCTCAAAGCTGCTCG[A/G]TTCGTACGTA
TP44449	1H	160.03	barke_contig_2779723	TGCAGGCAGTGTCGCTTTCAG[A/C]TCTGCAACCTCC CACACCGAGATCGGAAGAGCACACGTCTGA
TP48126	1H	160.03	bowman_contig_9264	TGCAGGCGT[C/G]ACCGTCACCTCCTCGATCAGCGC CTCGATCTCCGAGATCGGAAGAGCACACGTC
TP48338	1H	160.03	morex_contig_156695	TGCAGGCGTTGGGTACAACTTAGCCATTGGCGACG GCAA[A/G]TAAAGTTGTGATAATCATGCAAGT
TP48503	1H	160.03	bowman_contig_883131	TGCAGGCTCACTGC[G/T]TTCACCACAAAAAGAATC ACACCGTATATATATACCTGGGAGGGTTCTG
TP57240	1H	160.03	bowman_contig_67501	TGCAGGTTCTGGTTGTACCGCTCCTGGACG[C/T]CAT GGCGTCCGCCGAGTGCTGCTGCGCCTCCGC
TP58083	1H	160.03	barke_contig_545066	TGCAGTAATCTTA[C/T]AATTAACCATGCATGCATGC ATGAGCAGCTCGAGCCAATCGAGAACAACT
TP58084	1H	160.03	morex_contig_39104	TGCAGTAATCTTATACTTAACCATG[C/T]ATGCATGC ATGAGCAGCTGGAGCCAATCGAGAACAACT
TP59665	1H	160.03	barke_contig_271782	TGCAGTCACTCAGATGCTACTTTGGAAAATGAAACT TCCAAGGAAGATGAAAA[A/G]GCTATATTAA
TP60256	1H	160.03	barke_contig_1791971	TGCAGTCCGCCGTTGATGGAGTACTCTAGTGACACT TTGTTA[A/T]TGTCGTCCGAGATCGGAAGAG
TP60856	1H	160.03	barke_contig_587847	TGCAGTCGGGAACAATTC[A/G]AGGGGGTTTTCAAA AATTTCCAATTATGTTTGACCGAGATCGGAA

Marker_ID	Chr.	cM	Barley_Contig	Sequence
TP60864	1H	160.03	bowman_contig_870990	TGCAGTCGGGCACCCCAAATCCGC[C/T]TCAAACGT CCGAGCTGACGGCCCGAGATCGGAAGAGCAC
TP61331	1H	160.03	morex_contig_135979	TGCAGTCTTTGTTGCCATATGAGTCTGTACTCTTGAT GAAAT[C/G]CTCAACCCACGATGATAATAA
TP62706	1H	160.03	bowman_contig_149713	TGCAGTGGAGCGTGTCCAGGCGGTGACGATGACGG GTGCTCGATGGTTGTTTGCGAAGGACA[C/G]G
TP63448	1H	160.03	barke_contig_541871	TGCAGTGTGAAATAGTGTAAAAAATCTACAGTCAA CAAAGC[C/T]TTTTTATGCCATGACCCGAGAT
TP65822	1H	160.03	morex_contig_43798	TGCAGTTTG[A/G]CTAGCTAGCACTGTGCTACCGTTG TGATGTTGTGTAACATGGTACCATCTCCTC
TP13	1H	161.32	morex_contig_1569224	TGCAGAAAAAA[C/T]AAAAAGCGGCTTCCGCGATGC TGTCAACTGTCTGATCGAGCGGCCACTAAGC
TP6664	1H	161.32	morex_contig_2521503	TGCAGAGCCCCCTCATAGAATTGATCGAAGAAAGC CATA[A/G]TGTCTGTCGTTTCTGACGAGCTGC
TP11916	1H	161.32	barke_contig_73724	TGCAGCAA[A/G]GATGGCATGTCTTCGCAATGTGAC AGCAGAAATCCCAAGAAGAAAACCATGAGCT
TP12848	1H	161.32	barke_contig_518252	TGCAGCAAGCTTCCT[C/T]TTGCTAACCATCATCCTA GCCAGCACCACCAGCGCCACCTCCTCCTCC
TP25141	1H	161.32	morex_contig_8647	TGCAGCCTGCTCCTCCTCAC[A/G]TAACACATCGAA CAGCTCCTGCTTCTTCTGCTCTGCCTTAGAC
TP33143	1H	161.32	barke_contig_118724	TGCAGCTAGTCGTGATGTAGTA[C/T]GGAAAGAAGA TGGGAGGACCCTGCTGCTCCACGAATCTGAG
TP41799	1H	161.32	bowman_contig_21265	TGCAGGAGCGTCTTCGCCCTCGACTCCCCGCCGCTC TGGGG[C/G]CTCCAGTCCGTCTGCGGCCGCC
TP53450	1H	161.32	barke_contig_272256	TGCAGGTAAGCTCTTTCACCTCTTAGTTTTGGCG[G/T]TGTTAGTTTCTCTTTTTATCTGCTGAGGA
TP61403	1H	161.32	barke_contig_60483	TGCAGTGAAGAGGAGCGAGCCGCCACACCTGCTTC CGATT[A/C]TTCTTCATCTGGGCCTTATCCGA
ABCt2	1H	0		AGAGACTTGCATTTCTATCCACCAT[T/G]GGCTTTTA CGCTTCCTTCTTGGCTC
TP2808	2H	0.00	-	TGCAGAAGTGTCCGAC[A/G]TATAACTTTTCCAGTG AATCCGAGATCGGAAGAGCACACGTCTGAAC
TP5259	2H	0.00	barke_contig_378261	TGCAGA[C/T]GGGTGCAGCAAGAAGTCCTGCTATGT CATCTGTCAAGAGCTCACACAGTGTGGACGA
TP10453	2H	0.00	morex_contig_48487	TGCAGATGCCTTCCTTTAGTCACGAGGAC[A/G]GTG AATCAACCAATTGCTTGCCATCAACATCAAC
TP11430	2H	0.00	morex_contig_2550001	TGCAGATTGATGTCTTTGTTAATGCAAAGTTGCATG CATACAAGGATGGTGTTTTGGCTCCCG[A/C]
TP15655	2H	0.00	bowman_contig_143370	TGCAGCAGAGGAT[C/G]TACGCGGGTGGCGGTGTCC CTGATGTTGAGCACACCGAGATCGGAAGAGC
TP33273	2H	0.00	morex_contig_48049	TGCAGCTATTGCTTCTGGTTAATTGGTCCAAGTGGA T[C/T]TCAAGTGAATCTTACCAGTTCGTGTC
TP36037	2H	0.00	bowman_contig_859994	TGCAGCTGCGGCAGGGTGTACGGCTACGCCGTCAG

Marker_ID	Chr.	cM	Barley_Contig	Sequence
				C[A/G]TCATGCCAACGCCGAGATCGGAAGAGC
TP38067	2H	0.00	morex_contig_1563469	TGCAGCTT[G/T]CTGAGGACGGCGAGGGCGCTTGCGGCGGAAAGATCCATGGACGCGGGGGGAGGCC
TP56039	2H	0.00	-	TGCAGGTGCTACACCAAAGGATGACCGCCGAATAC[G/T]CGAGTATGCCGAGATCGGAAGAGCACAC
TP59954	2H	0.00	morex_contig_267414	TGCAGTCCAATACTAAAAAGTAATCTCAGACATCTGAG[A/G]CCGAGAGGAGTGATGCTGAAAGTCT
TP62326	2H	0.00	bowman_contig_15026	TGCAGTGCGCGAC[A/G]CCGCTCCCCCTGCTGCGGCACGTGGCGGCCGCCATGGCCGCCGAGATCGG
TP64787	2H	0.00	morex_contig_41048	TGCAGTTGAA[A/G]TAGGCTCCCCTTGGCCTAGATTCGGATCTCACGTACGGCGCCATGAACGCGTA
chitinase2a	2H	18.3		AGGGCAACAAGCCGTCGAGCCACAA[C/A]GTCGCCCTACGCCGCTGGACGCCGA
TP55154	2H	34.45	-	TGCAGGTCGTC[C/T]GACGCGTTTTTGGATGTCAGAACATCCCGAGATCGGAAGAGCACACGTCTG
TP53831	2H	34.45	barke_contig_269593	TGCAGGTAGCTTATTGCAGATTTCAGTGTTCGGAATAAAAGATGACTGACAGGATCG[C/G]TATAAT
TP30995	2H	34.45	-	TGCAGCGGGTACATGTGGTGCCCATTGAAA[A/G]TGTGTCGGACCGAGATCGGAAGAGCACACGTCT
TP3397	2H	34.45	bowman_contig_63848	TGCAGAATTTGCTATTGGTCTCCTCCAAGGGCCCAAGAGGTAG[A/G]TATCTTATATACTAGTAGCA
TP57702	2H	34.45	morex_contig_67574	TGCAGGTTTCATGCAACGCACGCAATGCAAGCTGTTCGATCCAAAGCCAACTAGCTAATCACC[A/G]
TP49597	2H	34.45	bowman_contig_1985886	TGCAGGGACCCCAAGCACTGGGA[C/T]GCCGCGGAGGAGTTCAGGCCGAGATCGGAAGAGCACACGT
TP39044	2H	34.45	bowman_contig_63848	TGCAGGAACCGAGGGGCGAG[A/G]CCGAAAGAAGTCTCGGCGGGGCAGAAAAGGAAATCTCCCGAGA
TP27814	2H	34.45	bowman_contig_10439	TGCAGCGCCG[C/T]GGGACACAGGGGGGGAGGGGGAGTGGTCGTCCTCAGCAGGAACAGCGGCGGAG
TP10433	2H	34.45	bowman_contig_125833	TGCAGATGCCGAGCTCGCGGAG[A/G]TGGGGGAAGGAGGCGCCACGCAGCGCGGCCGTGCCCGAGAT
TP9619	2H	34.45	bowman_contig_1985886	TGCAGATCGCCCACGCGTTCACGAACACGGTGG[C/T]GCCCTTGGGCACGTCGTACCCGAGGACCTT
TP8308	2H	34.45	morex_contig_44971	TGCAGAGTTTGAAATGCGATAAACCCACTGGTAACCACCTG[A/G]GCCTCAAGTTCTGGGAAGAGTC
TP5998	2H	34.45	morex_contig_55908	TGCAGAGACGTCCAT[A/G]GCCGTGCCGTCGTCTCCAAAGCCTGCCGAGATCGGAAGAGCACACGTC
TP65042	2H	45.00	morex_contig_44492	TGCAGTTGCCCGCTTAACCCTGTTACCCAAGGCCTCACAGTACC[A/G]ACTTTCTTCGCCCGAGATC
TP63460	2H	45.00	bowman_contig_149324	TGCAGTGTGACGCCGAGAGCTTAGGCGCCACACATGCA[C/T]TTATGCTCGGCGCTCAGGCACTCTT
TP63268	2H	45.00	morex_contig_44492	TGCAGTGGTT[C/T]GCCGACAAGGGCCTCACCGCCTACAGCATCTCCTGCGGCACCTCCCTGCTGTA

Marker_ID	Chr.	cM	Barley_Contig	Sequence
TP34998	2H	45.00	-	TGCAGCTCTATC[A/G]ATCCTCCTCCCATGATCTTCT TGCTTACAGCGATGCTGATTGGGCGAGCTG
TP33068	2H	45.00	bowman_contig_69836	TGCAGCTAGCTGAACCCGTCGACGTTCG[A/T]GCAG ATATTTTCGCCTTCTGAATCTCCATTGCTGG
TP32460	2H	45.00	morex_contig_276521	TGCAGCGTTTCCACTTTTGGGGACCTTTGAGAGT[A/ T]TCCTAGAAGTTTTTTCTGGCTTTGGGCCT
TP27748	2H	45.00	-	TGCAGCGCCGCCGCGCTGTACGGCGTCGGCATCGG[C/T]GCTCCGAGATCGGAAGAGCACACGTCTG
TP25666	2H	45.00	-	TGCAGCGA[A/T]TAGCAGTTTCCCCTCAAAAATGGG TCACCTCGTCACCACTTCGACTCCCCGAGAT
TP2043	2H	45.00	morex_contig_8694	TGCAGAAGCACCAGATCAGCGAGTCAGCACGCTCG AACCCCATGGCACCGCCCCCCAATCG[C/T]CC
TP63428	2H	46.30	-	TGCAGTGTCT[A/C]CATACACCACATGTCCACATATG GCTGGATGCAATCGTTTTCTGCTAGGAAAC
TP59916	2H	46.30	-	TGCAGTCATTTTCGGGCAGCATGGAAGAGAGC[T/A] CAAATTCTATATCCCGTCCATGCATTTCCAT
TP57945	2H	46.30	barke_contig_380722	TGCAGTAACCAGTTTGTCACTACTT[C/T]AATAGATA TAATAGAGTATACTCATCATCGTGTAAGCA
TP51793	2H	46.30	morex_contig_37899	TGCAGGGGATCACCGACTCGTTGTAGAACGC[A/G]G AGAAGGACACGCAACAATGCGGCGGCTTGGC
TP47172	2H	46.30	bowman_contig_859491	TGCAGGCGCGCATGGAGCAGAGTGCTCGGCTTCTG ACACCAACTCTGTCAAGGCCCAAGCT[C/T]AC
TP43801	2H	46.30	morex_contig_48444	TGCAGGCAATCCTCGGCATGACTGTGCTGCTGCCTC ATTGGATAGTGCATCCCATATGCCATC[A/C]
TP35662	2H	46.30	-	TGCAGCTGCACCTGCACGGCGGCACCAGCG[A/G]CG GCGGCGGCATTGTTGGTTGTCGCGCCGAGAG
TP35636	2H	46.30	-	TGCAGCTGCACATGCACGGCGGCACCAG[C/G]GGCT GCGGCATTGTTGGTTGTCGCGCCGAGAGCGA
TP33330	2H	46.30	-	TGCAGCTCAACTTTGTGTTGCTATCCTACGGAAGGC CAACATCCCTCA[A/G]CAAAATGATCACATT
TP26789	2H	46.30	morex_contig_54751	TGCAGCGC[A/G]AGGCAGAGGGCCGTTGCTGTGCGT GATATGAAGAGGCCGCAGCGACCCAACTGGC
TP24501	2H	46.30	-	TGCAGCCTA[C/T]TCCTCCGATGCAGTGGCCGTCAAT ACAGAATCAGAGATACGCAAGGGATCATAG
TP9952	2H	46.30	bowman_contig_878275	TGCAGATCTCGAACCCTGATTCAATCGACTCCTAAC ACATAAGCAACCAGCTAGA[C/T]CACCAGCT
TP7094	2H	46.30	bowman_contig_871991	TGCAGAGCTCCACTTGCGGGAGCCTCTTATTTAACA CTCTTTGCGTCAATTAAGAGGTCCTTT[A/T]
TP3906	2H	46.30	barke_contig_396116	TGCAGACATGCCATGCATATGGCTGACAATGGCGA AAA[C/T]ACAACCCATGACGAGCAGGCGTCTC
TP2297	2H	46.30	morex_contig_135631	TGCAGAAGCTACAGCGTTCTCAGCAAGGCG[A/G]AC TCGACGGTGCCGAGATCGGAAGAGCACACGT
TP13049	2H	46.30	-	TGCAGCAATAAAGG[G/T]CAAAAAATCCCCGTCATC

Marker_ID	Chr.	cM	Barley_Contig	Sequence
				ACTGAGCTGTGGGGCACACGCGCGCTCTCCC
TP64156	2H	58.21	-	TGCAGTTCATCCACATCGCGAAGG[C/T]ATTTTTTCT ACTTACAAAAATGGCGTCCGAAAAAAAAAA
TP64157	2H	58.21	-	TGCAGTTCATCCACATCGCGAAGG[C/T]ATTTTTTCT ACTTACAAAAATGGCGTCCGAGATCGGAAG
TP61284	2H	58.21	bowman_contig_849574	TGCAGTCTTCCCCTCCCCGTTTCACTTTT[A/C]GTGA GCGTTTTCTTATCCACACGCACTCGCGGCC
TP49639	2H	58.21	bowman_contig_1982097	TGCAGGGACGAGATGCGCAGGGTGCTCGGCGT[G/C] GCGCTGCTCTGCGCCTCCAGCCTCCCGATCA
TP43562	2H	58.21	bowman_contig_884450	TGCAGGATTGCTGCCGCGCAGA[C/T]GGGCTCAGCT CCAAGAGCCCCAAGATCAGCATGCCCAGTAG
TP33902	2H	58.21	morex_contig_51248	TGCAGCTCCCGAAGGGGGAGCAGCTTATTGGGCG CCTCTTCTCAAGCACCGCTGGATCCA[A/C]AT
TP31050	2H	58.21	bowman_contig_13395	TGCAGCGGGTGGCGTACCT[C/G]AACGTCGGCATCT ACCCGTTCACCTCCATCTTCCTGCTCACCTA
TP16309	2H	58.21	morex_contig_37304	TGCAGCAGCAGGAC[G/T]ATGACGACCACCGACGCC ACGAACGAGGTCGAGTTGCAGTAGAAGAAGG
TP10773	2H	58.21	morex_contig_39661	TGCAGATGGCGGTGAAAATGCATGTGTCAGCAAAT ATAGGACTGATCCATCATTTTGGAAATG[A/G]
TP9111	2H	58.21	morex_contig_164370	TGCAGATC[A/C]TAGGAGAGGTGGATTATCGAACAG GTCTGAAGTAATTAACCAACATCTACTAGGT
TP4465	2H	58.21	barke_contig_268711	TGCAGACC[G/T]TGGTCACCACTCGCCACCACAAAT TTTCTATCCCTGGATGACCGAGATCGGAAGA
TP975	2H	58.21	morex_contig_37304	TGCAGAACAACGTGTTGCTGCTCTATGCCATGAACA CGGCGATCGTGCTGGACCTGCTCGG[C/T]CT
TP52666	2H	59.51	morex_contig_66374	TGCAGGGTCATCTGGTTCATGGCAGAGTAGAGGGC C[A/G]CCGAGATCGGAAGAGCACACGTCTGAA
TP65352	2H	60.81	-	TGCAGTTGGTCC[A/G]AACCATGCAATGCGTGCGCT GGCATGCGGCACAGCCTTGAGTACTTGGACA
TP63424	2H	60.81	-	TGCAGTGTCGTGCT[A/G]GGTCCAGGCGGGCCAGCA TAGCCATCCGCGAATACGTCCATGGAGGATC
TP62294	2H	60.81	morex_contig_1575898	TGCAGTGCGAGCCAAACCCTCT[C/G]GACTACCTGT CCTTCCCGATAGTCAGCACGGCGATCGAATA
TP15954	2H	60.81	barke_contig_54782	TGCAGCAGCAACTGCTTCGGCAAGATGAGC[A/G]AC AGTGAAATCATATTTATGGATCAAATGCAAG
TP9739	2H	60.81	morex_contig_1561391	TGCAGATCGGCGA[C/G]CCGTTCTCCTCCTCATCACC ACGACCACCACCGAGATCGGAAGAGCACAC
TP42469	2H	60.81	morex_contig_140099	TGCAGGAGTAGCAGCCAACTCCTTCTCTGAATCTGC CTGTAGAC[A/G]TGCAAGCAACGTACTTTTA
TP41047	2H	60.81	bowman_contig_67102	TGCAGGACT[G/T]CTATAAGCCCATGCACCTTCCCCT GCTCTTCGATGGCTCGCACGTGCCCTGCAC
TP53127	2H	110.81	morex_contig_160066	TGCAGGGTGTGGGCGGGGAGCTGCCACCCGTGACG CCGCGCCATGGCGGCGC[C/T]GCCGCGGCGGG

Marker_ID	Chr.	cM	Barley_Contig	Sequence
TP61068	2H	110.81	bowman_contig_1985453	TGCAGTCTATTTCCTTAACTTCTGAGTCACTTTCACTGAAAGCCGAATTATA[A/T]CTATCTTGGGA
TP152	2H	121.78	morex_contig_1560381	TGCAGAAAAGACGAGAACACATGTGAGGCGTACTGCGTACAGT[A/T]CAGCACAGTACAGCGGTTCA
TP1019	2H	121.78	bowman_contig_851495	TGCAGAACACATGACCAATCAAGAAT[A/C]TATACATGCTACAAGCCCCTTCACAAATAATCCTTTT
TP3868	2H	121.78	barke_contig_2783595	TGCAGACATCATTTTCCATGTAAATTATGGAGAC[A/G]GTAGGTCCTGCACCGTCTACCAGGTTGTG
TP5553	2H	121.78	morex_contig_41384	TGCAGACTCGAGTATCGGGGCGGCAGGGTTTCGATCG[A/G]GAGGAATCCGAGATCGGAAGAGCACA
TP17477	2H	121.78	bowman_contig_1987151	TGCAGCAGCTTTG[C/T]CAGGCCAGGTTTATTTCTGTCATGGTTCAGGGTGTCTAACTGTATTTAA
TP27066	2H	121.78	barke_contig_4583	TGCAGCG[C/T]ATAAAAATCCCTTCGATCGATTCGTCAGCGGCAGCAACAGTGATGAGCTTGGTAGT
TP41250	2H	121.78	morex_contig_50474	TGCAGGAGACGCCCGTTATTCACCTCAGACGGAGCAGCGGGA[C/G]GGGTTGGTTGACTCGAGACCG
TP43565	2H	121.78	bowman_contig_10214	TGCAGGATTGGACATGGAAGTACACTTTTACTC[A/G]TGATGTTAGGCATGTTTTACACACATGAAA
TP50342	2H	121.78	morex_contig_2551748	TGCAGGGCACGTAAGAGGATTATTAGAGGAACGGTTAAC[C/G]AAGCAAGAAATGCATGGCGTCGTT
TP50873	2H	121.78	morex_contig_2550755	TGCAGGGCGAAGAAGATGGTGCAGCAGCAG[A/G]ATAGTAGCTGCGGCGATCGTCCTATGTTCCACG
TP59519	2H	121.78	barke_contig_271057	TGCAGTC[A/C]AGCAGTACAGAGCTGGCCGCAAGCCCGCCGAGATCGGAAGAGCACACGTCTGAACT
TP62489	2H	123.08	morex_contig_38845	TGCAGTGCTCGCTC[A/C]CGTGCTAATCTAATCAAACCTAGTGCAATCCCCTAAAAGTGCAGGTTTG
TP63205	2H	123.08	-	TGCAGTGGTCTTTGGTGGCGGCGGCATGGGTCTCTCCTCAAGCTTCATC[A/G]GGATGTGCACGGTG
TP10600	2H	124.38	barke_contig_274041	TGCAGATGCTGGACTACAACGTGCCTGGAGGTGAGCAGCAGCTGGCCGATCA[C/T]CCCTCCTCGTT
TP17300	2H	124.38	-	TGCAGCAGCTGCACCTGGAGGAACGGCTGCTGCG[C/T]CGCACCAGCGATAACTGGTGCATCGTCAA
TP29704	2H	124.38	barke_contig_1802081	TGCAGCGGATAAGCATTATTCATGGTTGCCTTCCAAGTATAG[A/C]ATAGGCACATAGTAGAGCGGA
TP33312	2H	124.38	morex_contig_45337	TGCAGCTCAACCCTGATGAAAAAAGCGATCAACAAGTCGGCCGTCCTCGTGATCCT[C/T]CTAAATC
TP35599	2H	124.38	bowman_contig_887399	TGCAGCTGCAAGCGATAC[C/T]CTCACGTCGGGCCAGTCGCTCGCTGTCGGCGGCAGCAAGCTCGTC
TP62596	2H	124.38	-	TGCAGTGCTTGGCGTAGATGAGGAAGAGGAAGAG[A/C]CCCAGGAGGACGCCGAGATCGGAAGAGCA
TP29714	2H	124.38	barke_contig_1802081	TGCAGCGGATCACGACTGCACGAATTATTGGA[T/C]GGTTCAAATACAAGGTGAGAATGGCAAAGGC
TP750	2H	126.98	morex_contig_1569720	TGCAGAAATCGCTGTCACTTTTGGTTTCAGCAGTCA

Marker_ID	Chr.	cM	Barley_Contig	Sequence
				GGA[C/T]TATAATTTTCTTATCGACGATCCC
TP3826	2H	126.98	bowman_contig_116691	TGCAGACAGTTTCAAAAACACAT[A/G]AATTAATT AAGCCCCCTCCAGCTCGTACGTGCTCACCGA
TP3830	2H	126.98	bowman_contig_129881	TGCAGACAGTTTTGAATCGCGTGACAGATTT[A/T]G CAGGAAGCCGAGATCGGAAGAGCACACGTCT
TP7301	2H	126.98	morex_contig_55057	TGCAGAGGAAGCTGCTACTTTAATTGCTT[C/G]GTTA CTGAAATTTGTCAAATTTGCAGGTCGGTAT
TP15860	2H	126.98	barke_contig_1780560	TGCAGCAGATGTACGCATA[C/G]CCGCACTTAATTC GAGGCCTTACGATCTTGCATAGTAATTTGGC
TP18369	2H	126.98	bowman_contig_1993208	TGCAGCAGGTGC[C/G]GTCGGATGAGTAGGCGCCGC TGCCGACCTCACCTCCGAGATCGGAAGAGCA
TP26382	2H	126.98	bowman_contig_852828	TGCAGCGAGGGGACAGGGTG[C/T]GCAGCAGGGCA ATGCGGCAGCGGCAGGAGTGGGTGGGGGACTG
TP48323	2H	126.98	bowman_contig_125967	TGCAGGCGTTGAGGTCGGCAGC[A/G]GCTCCTACTC CGACGGCACCTGCACCCCAGAATCAGTACGA
TP50074	2H	126.98	barke_contig_269115	TGCAGGGATCGCGATCCCCCAACACAATTACGTGA AGCAATGC[A/G]GAACCATTTTGAAGCGGCGT
TP52973	2H	126.98	bowman_contig_90361	TGCAGGGTGCGGGGAG[A/G]GAGTCAGCGAGATGA CGCATCGGCGCGAGCCGCTGGAGGTGGAGGAG
TP58622	2H	126.98	bowman_contig_1982422	TGCAGTACTGGTGGCAACCTTGTGTAACTC[C/G]GTT CATAGTAGTAGTAAAGTTCTTGAGGCATAC
TP59146	2H	126.98	morex_contig_47410	TGCAGTATATGCAACCCGTTATCTCTACGGTTTGTG ATAAGTAGTGTTGAAGCTGCTG[C/T]GCGAC
TP59814	2H	126.98	barke_contig_132880	TGCAGTCAGTGTGGCTCAGTGCACGGGTCATGAAA AAA[A/C]ACACGAAAAACAGTCATGGGACGCG
TP65864	2H	126.98	bowman_contig_2020879	TGCAGTTTGCCATGTAACTTGCTCGGCGATCGTCAA CTTTATC[A/G]CTTAAATTCATAAGGTCGCA
TP5683	2H	126.98	bowman_contig_12872	TGCAGACTT[A/G]GGAATCTTCAACGTAATCCGTAA AAACTTCCCGCATCCGTTCGTGGCCAGAAAA
TP48811	2H	159.46	bowman_contig_17582	TGCAGGCTGAGAGCCGCCGCCCACGTGCGCAGAGG[C/T]GGCGTTATTCGAAAGGGCCTCGGCGCCC
TP44985	2H	159.46	morex_contig_87345	TGCAGGCC[A/T]GTCTACATCAACACGCCAGGGGCA GCGGACGACAGTACGAGGTGCCCAATGAACG
TP19035	2H	159.46	barke_contig_282216	TGCAGCATCAAATAGGGATTGTCCTCTCCCGCTTGC TGGCG[C/T]ATGCTGCGCCCTCCCGTGAGCC
TP23713	2H	159.46	morex_contig_416253	TGCAGCCGCCGCGCACGTCCACGACGCCGCAGAGC ACGGTGTGCCAC[A/G]GGGGCTGACGCGGCGG
TP26248	2H	159.46	bowman_contig_847367	TGCAGCGAGCGCAAAAATATTTGGCAACGCACTGT ATCATT[C/T]GACTGTAGCCATGTGTGCCGTG
TP27592	2H	159.46	morex_contig_61005	TGCAGCG[C/G]CCGCTGAACCGATGGAATTCATGGC GTGGTACTGCGCAGGTGGCAGGAGGGCAGCG
TP33177	2H	159.46	morex_contig_120368	TGCAGCTATAGATCTGGGTGCAC[A/C]AGCGCCGTA TCACCCTTCATACTGGGCTCTATAGATCTGG

Marker_ID	Chr.	cM	Barley_Contig	Sequence
TP35161	2H	159.46	-	TGCAGCTCTGTAAAATCTCAGGATCTGC[A/G]AAGCACCTCTTTCCCCGCTCTGTAATTTTTGACTG
TP38600	2H	159.46	bowman_contig_64529	TGCAGGAA[A/T]AGGGCGAGTAACCGCGATGTCCACCTGTCCTTGTACTGTGCCGAAGAGAATTTTA
TP43755	2H	159.46	-	TGCAGGCAAGATTTCTCGCTTTTCCCCATTGGCCCATAGAGCTCCGC[A/G]TCGAAGGCCGAGATCG
TP51006	2H	159.46	morex_contig_2549358	TGCAGGGCGATGGGCGACCAGCTCGAGGGCGACGAGGG[A/G]CAGCACATGAAGTACCGAGATCGGA
TP65249	2H	159.46	morex_contig_135472	TGCAGTTGGCAAAGGCAGAGGGCTACAAGGGGTAGGCCTCGGCGTCG[A/C]CGCCGAAGTTGCGTAG
TP24166	2H	159.46	barke_contig_411457	TGCAGCCGCTTGG[A/G]TCCCATGCAGGCCACCGCTGAGGCGCCGCCACCGTACATAGCCGAGATCG
TP32600	2H	159.46	bowman_contig_142572	TGCAGCTAATCTAACCTCTAATGTTTTCCCATCAA[C/T]CAGGCGCCCATTCATGCGTTCAATAGCT
TP57712	2H	159.46	bowman_contig_852869	TGCAGGTTTCGATGCTCAGGGATTCAAGAG[C/T]ACCTTCCGAGATCGGAAGAGCACACGTCTGAAC
TP13294	2H	162.06	-	TGCAG[C/G]AATTTCAGCGGACAACTCTACAGTACCACGAGTGCCGATTTGCCGAGATCGGAAGAGC
TP13954	2H	162.06	-	TGCAGCACCCATCCATCCAT[C/T]ACACGCTGCTGCTGCTGCTGCTGAGGCGCTCGACCACCAT
TP16553	2H	162.06	bowman_contig_200644	TGCAGCAGCCAGGCTGCTTACACGTGCATGCACACCCTTGATG[G/T]GCTCAGTTACAGCAGGCGGA
TP34103	2H	162.06	barke_contig_274248	TGCAGCTCCTCACGGGGCGCCGTGTTGCA[C/T]CAAGGGCGAGGCAAGACGAAGAGGCACAACATGC
TP38708	2H	162.06	bowman_contig_314376	TGCAGGAAACTGGAAA[C/G]GGTCATCCTCGCGTCGCTGTGACTCCCCGAGATCGGAAGAGCACACG
TP1265	2H	163.37	-	TGCAGAACCGAAGCAGGGGAAGGAGCTCTGGGAACTC[A/G]TCGTTGACGTGGTCGGGGCCGTCGGT
TP13189	2H	163.37	-	TGCAGCAATGCTTATTGCCTTCAGCCGCCGAGGGGAAGGAGGTTT[A/G]GGGGAAGCTTCCGTCCGA
TP17256	2H	163.37	-	TGCAGCAGCTCTTCCAGCAGCACAAGCTCCAGGCCCAGGCCGACGACTCCACTTCAGCAGGCA[C/T]
TP52842	2H	163.37	barke_contig_58240	TGCAGGGTGAAGGCGGTGACCCTCGGGCAGCAGCAACCACCTCTTGGGGTGGAAGCGCG[A/G]GTCG
TP62550	2H	163.37	morex_contig_1568924	TGCAGTGCTGGTGCCTCAACTTCCGTGGCCGCGTGACGGTGGCCTC[A/G]GTCAAGAACTTCCAGCT
TP526	2H	165.98	barke_contig_1784968	TGCAGAAAGAGGCCTAATGAGGACTTGGGCAGACAGCAACCAGCATGAGC[C/T]TTGAAGTCTGATA
TP20807	2H	165.98	morex_contig_54095	TGCAGCCACCGTCCTACATAGATACAATGTTGCG[G/T]TGGTACAAGGATGTTTCGTACCCCGATGA
TP25066	2H	165.98	barke_contig_369923	TGCAGCCTGCATTTTACTAGTTTTAGCCCAATTCGATATAGTTTAGTTAAATATGCCTGAA[A/G]TA
TP46754	2H	165.98	morex_contig_6358	TGCAGGCGATGTGCCGTGCGCAGGGTCTGGCGCTG

Marker_ID	Chr.	cM	Barley_Contig	Sequence
				GACTCGACGCCTTTCGA[G/T]GAGTGCATCAA
TP58708	2H	165.98	barke_contig_447730	TGCAGTAGAGGAAAATTTAAAGGAGAATCATGTTC AAGGAGGTGCAT[C/G]GATGCATCAATGCCAC
TP539	2H	165.98	bowman_contig_10941	TGCAGAAAGCAAAATTTATAA[C/G]GGCTTCATGAA ATAGATTCAACATGAATGAATCTTATACTGT
TP12249	2H	165.98	barke_contig_1794812	TGCAGCAACATTGTGTCCTATGCGCTTCAC[A/G]CCC TCCACGCCTGGGATCCTCCGAGATCGGAAG
TP18471	2H	165.98	morex_contig_1561486	TGCAGCAGTAAGGAATTCTTGGAG[G/T]CGTGGATG AAGATGTATAGGGCAGACCTATATATGAAAA
TP23512	2H	165.98	-	TGCAGCCGCAGGTAATTAGCGCAACAACACTGGAT ATATACT[A/C]CTGCTGGACTGGGCTCACTGA
TP26659	2H	165.98	bowman_contig_331123	TGCAGCGATGTCTTGTGCTCAGACAGTGGCTT[G/C] AGCTGATGACCATCTCGGTCGCAATCAGCAG
TP30509	2H	165.98	bowman_contig_859696	TGCAGCGGCTCACCGTGGACTACGCGCAGAGGGGC GTCGTCCTGCCCAAGCCT[C/T]CCCCCGAGAT
TP31075	2H	165.98	morex_contig_169130	TGCAGCGGGTTGGGCCCGCTGACGTGGCAGTGCAG GTGCAGCGTCATCTCCCCTCC[G/T]CCCGAGA
TP32939	2H	165.98	morex_contig_2549473	TGCAGCTAGACGGAGATGATGACGAGACGGCGTGT TA[C/T]CTCCAGGATAACTCGCGAATTTTCTC
TP35111	2H	165.98	bowman_contig_157425	TGCAGCTCTGCGTCGGCCGCAGCTGCCTC[A/G]TCTT CCAGCTCCTCCACGCCGACTACGTCCCGCC
TP38458	2H	165.98	morex_contig_42752	TGCAGCTTTTCCCCCAGGCACCCTAG[A/T]AATGGC AGCTTCTCCCGAGATCGGAAGAGCACACGTC
TP43846	2H	165.98	morex_contig_1578068	TGCAGGCACACCAT[C/G]TGCGACACGATTAGCCAC GGATCCCACTCCATCGCCACATCCAACGTCT
TP53693	2H	165.98	bowman_contig_872278	TGCAGGTACTCGGACAAGATGTTGCTGTAGGTCTTA CCACCCTTGACCATTTGATCGGTCAA[C/T]A
TP63747	2H	165.98	morex_contig_511440	TGCAGTTAATCTTGGTCTTGAATTCCTGACATGGTT ATGATTGTTAATCCAG[A/C]TACTGTATATG
TP54387	2H	165.98	bowman_contig_851002	TGCAGGTCCAAGTAAAATAGACCGTCCCTTTCTTCT CCCCAAC[C/T]GCCAATGCAAAACCAGCCGA
TP53339	2H	167.28	bowman_contig_125273	TGCAGGGTTTTCGTTCCATGGTGAGCGCTACGACTC CGAATGCTCGCTATGG[G/T]TTTCCGTGGTG
TP10446	2H	168.58	bowman_contig_9518	TGCAGATGCCGTCTCTTTCGATCGACGTCTTAA[C/T] TTGCCTTCTCTTTCGTGTGGAGCCATGCCT
TP18733	2H	168.58	morex_contig_49284	TGCAGCAGTGGTGAAAATATCATAATGGACTGACC TTGAAACAAGCAATCT[A/T]TTACAAACTAAG
TP18983	2H	168.58	bowman_contig_125417	TGCAGCATATATGCATGTTTAACTTACTTTTATCTTC GAAACCACCGCA[C/T]TGTCCGAGATCGGA
TP27278	2H	168.58	morex_contig_159638	TGCAGCGCCACCACAGGCCCTCCCTAGCGCC[A/G]C CGCCGATGACACAGGCCCTCGCTAGCACCTC
TP32360	2H	168.58	morex_contig_42598	TGCAGCGTT[C/T]AGTGACGTGAGATGATTGGTGTA GCCATCCGCCGCCACCGTGTTCGCCGAGATC

Marker_ID	Chr.	cM	Barley_Contig	Sequence
TP36074	2H	168.58	-	TGCAGCTGCG[G/T]GACGCCGTGGCGTGCCGCCCCG TGTCGCGCCTCTTCCGAGATCGGAAGAGCAC
TP39022	2H	168.58	bowman_contig_14546	TGCAGGAACCCCACCATGAGGGCCGTCCACGACAC GACGTTCCTCTC[A/G]GGCATTCCGTCGAACA
TP52551	2H	168.58	morex_contig_2546833	TGCAGGGTACATGGATCCTGAGTATGTGGTGACCCA GGAGCTGACGGAGAAGAG[C/T]GACATCTAC
TP57099	2H	168.58	morex_contig_54190	TGCAGGTTCCAGCAAAAAAGTCTCAAGGCCACTGG CGTCGCCGCC[C/T]AGCACCATCCGTTGGATG
TP62956	2H	168.58	-	TGCAGTGGCTCCTGAGGCGGAGGTTCGCGAGAGCG GCGTCACGGTGTTGACGC[A/G]AAGACGGGGC
TP64083	2H	168.58	bowman_contig_10364	TGCAGTTCACTGAAACCACAATGAAGAAAATTAA[G /T]TCCTTCCTTTTTTGGGGGGGTCTCCTGAT
TP64777	2H	168.58	barke_contig_1785443	TGCAGTTCTTTGTCGATTTTTCTGCAAATGATTTACC TATTGTGGCCGC[G/T]ATAGATTTTCTAGG
TP5487	2H	168.58	bowman_contig_10364	TGCAGACTATGCTGTGATAGTT[C/G]AAGGTTTGTTT TTGCTATATATGTCTCCAGCCCAAATCAGA
TP32245	2H	168.58	morex_contig_135396	TGCAGCGTGGTGTTGGG[C/T]CCCATAGTCTGCTCCG TGCCTCTGCCCTTGAGGTACACGGACGCGT
TP36221	2H	168.58	bowman_contig_142085	TGCAGCTGCTCGTGCCACCTCGGGGACTTGTTCTTG AGAACCAGCGGGCTCTCCTTGTCC[C/T]CCG
TP36702	2H	168.58	bowman_contig_849131	TGCAGCTGGCGCGAGGCGGACCAGGAGCGGGACAC GCTCCAGGACAGGGACCT[A/G]AAGTGGGAGC
TP40667	2H	168.58	morex_contig_7844	TGCAGGACGAGGTGGGCCTGGTGGAATTCATCGCG GTGG[C/T]GGTGGGGGATACCATGGTTACAGA
TP58917	2H	168.58	barke_contig_58240	TGCAGTAGGAGGATCCCGTCGACCGAGTCGAGGA[C /T]GCGATAGTCGTCAGGAAGTCGAGGGCAAA
TP60489	2H	168.58	-	TGCAGTCGAGCCACTCCTCCGAGCTCTCCTTTCTCC CATTTTTAG[A/G]TTCTCCTCTAATATATAC
TP63633	2H	168.58	morex_contig_9839	TGCAGTGTTGTCGAACTAAAATGACACGTTGATATA CCTGCGGTAACAC[A/G]GTGGCTGGCCGCAG
TP61593	2H	168.58	bowman_contig_1986375	TGCAGTGAGAAAACAAAGTGTAAATACCC[C/T]GCA AAGAAGGTATAAATGTAGAGAAAGGTGTGAT
ZIP7	3H	0		AATGCCTGATTGCGCAGGGGCTCC[T/G]AGACACCC CAAGCGACAGCCCAATG
ZIP5	3H	1.7		CCCGGAGGCCTTCGAGCGGCTCGGC[T/C]CGCCGTG CCTCGTCGACGGGCCGTG
ZIP5	3H	1.7		TCGGCGCATCCCAGAGCGCCAGCAC[G/-]ATCAGAC CACTGGTGGTCGCGCTAA
TP32808	3H	80.00	-	TGCAGCTACGTGTATATATGTGCTGCTGGCAC[T/C]G TCGTAAAAAAGGAAAATGACCGATCGTCAC
TP19612	3H	87.98	morex_contig_2550528	TGCAGCATGATGTCATTGGACCTTGCCAG[C/T]ATTT CACTGAATGGCTTGCCTCATCTTGGTGATT
TP27006	3H	87.98	-	TGCAGCGC[A/G]GCGGCGTCCACGGAGTCGGCCGCG

Marker_ID	Chr.	cM	Barley_Contig	Sequence
				CCGCACGTCAGGATGCTGTGGAGAACCCGCG
TP6083	3H	89.17	bowman_contig_14726	TGCAGAGAGCCAGTCCAGCCAGCAGCAGCACGCG[C/T]GCAGCTCAGCAGAAGAGGTACCCAACGGC
TP41927	3H	91.88	morex_contig_334012	TGCAGGAGCTGGGGAAAGGAGCTGATTGAGAAG[A/G]AGATAAGAAGAATTTAGCGGTGCAAGGATC
TP63630	3H	93.86	bowman_contig_1987587	TGCAGTGTTGTATGGTGCTGAGTGTTGGCCAACTAAAAGGCGACATGTGCAG[C/T]AGTTAGGTGTA
TP13640	3H	106.39	morex_contig_54717	TGCAGCACATCAAGACGTCCATGCGTTGAGCAAAAGTGAATAGCACCGTTGAAATC[A/T]CCATCGT
TP32582	3H	106.39	barke_contig_2783003	TGCAGCTAAGGATGAAGCAAATCCTATGATCATATAAGTATATGC[C/G]AACCAAAGGGGAGCTGGA
TP29239	3H	106.39	morex_contig_67684	TGCAG[C/T]GCTCTGTGCACATTTCGCGGAATGCACATGGAGGGGTATGGGGGTATGGCTACGGATG
TP11716	3H	106.39	barke_contig_370701	TGCAGATTTTGTTCTTGCATCGTTCA[C/T]TACAGCAGTATTGTCAAGCTGTGCTCATAAACCGAGA
TP60843	3H	107.69	morex_contig_1579788	TGCAGTCGGCGTGGTCTTTGCCGCACGCTGGCCGCGGCGGC[A/G]GCATCACGCTGGCGGCCCTGAT
TP57749	3H	107.69	bowman_contig_864906	TGCAGGTTTGCAACCCAAATATCCAACCAAAACCAGTTGTCA[C/G]CCTACAGCAAATCCTTCAATT
TP56636	3H	107.69	barke_contig_71785	TGCAGGTGGTGT[A/G]CGTTGTTGTTGAAACTTTGAAAGCCTTTGTTGATCGATCGTGTGCAGATTC
TP16830	3H	107.69	morex_contig_45263	TGCAGCAGCGCCGTCGTGGGGAGCACGAAGGAGACGTTGTTCACGGCCGCCGC[A/G]AACCGAGATC
TP941	3H	107.69	barke_contig_588249	TGCAGAACAAAATCGCTCAAG[C/G]CAGCATGAGGCACAGCATCCGCCGTGCTGAGCCACGGCTCGA
TP11812	3H	107.69	morex_contig_1579788	TGCAGCAAACAAAGCAGCAGAGCATCAAATCCAACTGCCAC[C/T]GTTATATTGCAGGTAGTAGCAA
TP60565	3H	107.69	morex_contig_45263	TGCAGTCGCA[C/T]TACACGGGGATGTCCAACGGGGTGTACTCGTCCAACTTCCCCGCCGTGCCCAA
TP8095	3H	142.15	bowman_contig_847525	TGCAGAGTGACATTTGTGCATCAAAGTTCT[A/T]TTATTATATCTAGGGCCGAGATCGGAAGAGCAC
TP19440	3H	142.15	bowman_contig_142689	TGCAGCATCTCGCG[C/T]GGTCGCCTTCGGTCACCGCCGTCAGGCACAACGGCTGCCGAGATCGGAA
TP19670	3H	142.15	bowman_contig_62411	TGCAGCATGCCCGCCATGGCCTTGTCG[C/T]GCTCCAAAGCACACGGGGATAGCCTCTCGTCCGAAC
TP34519	3H	142.15	morex_contig_244073	TGCAGCTCGCCGACGTGCTCC[A/G]CGGCCGAGGCCTCGCCATCACCATCCTCCACACCACCTTCAA
TP48884	3H	142.15	bowman_contig_916079	TGCAGGCTGCATCCTCTTTCTCGGGGTTCTGGCTACTGGTTTCGTTCTTCTAGTGATC[C/T]CTACG
TP51383	3H	142.15	barke_contig_2790737	TGCAGGGCGTGGACAGTTCACATGACTC[A/G]GGGGAGGCCCTGCAAGCCGCGAAGGCATTGGCACG
TP52826	3H	142.15	morex_contig_7979	TGCAGGGTG[A/G]AAGCAAACCTATAATAGTATTTGACACGGCAGCTGAGTCGTTCCGAGATCGGAA

Marker_ID	Chr.	cM	Barley_Contig	Sequence
TP58927	3H	142.15	bowman_contig_221597	TGCAGTAGGATCCACCACAGGTGGTGTGACATTCAT [A/G]GCTCGCCCCCTCGGAGCCGAGATCGGA
TP2965	3H	142.15	barke_contig_2781116	TGCAGAATCACCGACTGAAAACAATTAAAGCTTGG CCAGAATCTTGAAAGAGCTTT[C/G]ACCAACC
TP5940	3H	142.15	morex_contig_6555	TGCAGAGACCAT[C/T]AATGCTTATACTTGGGTTCTT CATCCCTTGTTATCCCTGGATGTGTAGACC
TP9985	3H	142.15	bowman_contig_846731	TGCAGATCTGCGGCGA[C/G]CATGCGGACGGCAGCA GGAGCGACACCTCTCCGTCGCAGCAGCCGTC
TP10688	3H	142.15	bowman_contig_2002163	TGCAGATGGAGCATGACGGCGACTAA[C/T]TCGTGG TGAATCCGAGATCGGAAGAGCACACGTCTGA
TP10695	3H	142.15	bowman_contig_17214	TGCAGATGGAGGAGAACCTG[G/T]TGGTGTTCGACG AGGAGAAGCAGACGATGGCCTTCACCGAGAT
TP15709	3H	142.15	bowman_contig_1184523	TGCAGCAG[A/G]TACCATGCTCGATTCGCCAGTGGA TGCACAACGGACCGCCGAGATCGGAAGAGCA
TP16507	3H	142.15	barke_contig_2789793	TGCAGCAGCCACATGTCTTCCTTGCCAAGGTGG[A/G]AGCTTGCCCGCGATCTTATCTTCAAGATGC
TP16674	3H	142.15	bowman_contig_26047	TGCAGCAGCCTAAGAAGACGTA[A/G]CAGCCCCAGG TCCACGCCGTGCACATCCCGTGCCGCCTTGC
TP18406	3H	142.15	morex_contig_159562	TGCAGC[A/G]GGTGGCGTCGCGTCGGCCCCCGCCGT GAAGCCACGCGCCCCCTTCTCAAAGCTCCCA
TP21391	3H	142.15	bowman_contig_195149	TGCAGCCAGCTCGGAACATCAACCTCCTTCCAATCC ATCTTTTTTGGCTTT[C/T]CCTTGATGTTAT
TP24010	3H	142.15	bowman_contig_86295	TGCAGCCGCTCCTCGGCAAC[C/T]CTTGCGTCGATGA GCCCGCGGGTGGGACTGGGGTGAAAAAGGA
TP33272	3H	142.15	morex_contig_68252	TGCAGCTATTGCTCTGACCCTCGGTCGGAGTTACCG AGCGC[A/G]TGCCACGATCAACGCTTTCGTC
TP33625	3H	142.15	barke_contig_443213	TGCAGCTCATGTGGATATAGATAGCTAGGCCGTTAA GCAGCAGC[A/C]CCACACTCAACTGCCCTCA
TP37712	3H	142.15	barke_contig_1808605	TGCAGCTTCTATCTTCTCTGCTG[C/T]TGCTGCCTGCT CCAAGACTGCTGCGGCCCTTTCTTCGGAC
TP40500	3H	142.15	bowman_contig_83164	TGCAGGACCTCTCTGGCATTTTCTTCTCTAGACAAT GATTTTCTATGCACATCGCGTCCG[C/T]CAA
TP42935	3H	142.15	morex_contig_39773	TGCAGGATCGCCA[A/G]TGGACACTGCACGCGATGA GTTAGCTAGGTTGATTAGCAAGGTTGAGATT
TP49127	3H	142.15	bowman_contig_263279	TGCAGGCTGTCAAAATTCGTTGCCAATTTCAAGGGC ACAGGCAACAGGTCGCG[C/T]GCCACAGATG
TP54347	3H	142.15	barke_contig_269985	TGCAGGTCATGCCTTTTTGAGGGATACAGCCACCCA CGGTCGTGGGCTTCTGGCAAAGTC[A/G]TCT
TP55134	3H	142.15	barke_contig_2789793	TGCAGGTCGTACGGTTCTTGTCAAG[C/G]CTTTCCTA ACGGACATCGCAATATATCATCTCACTCCT
TP56729	3H	142.15	morex_contig_47201	TGCAGGTGTCGTCGAAGAAGACGGCGGAGGATCTG TGGGCGAGCCTGAAGAC[A/T]CGGTTCATCGG
TP58076	3H	142.15	bowman_contig_151644	TGCAGTAATCAGGATACGACTTCACCACGTTTTA[C/

Marker_ID	Chr.	cM	Barley_Contig	Sequence
				T]AGACAACAATTTCGACAAGATAGCCATTT
TP8158	3H	142.15	barke_contig_269985	TGCAGAGTGCTGAGCAGAACAGGATGAAGCCCCTG ATGGAAGCCGCGATTGTTGAT[C/G]TATGCAG
TP13952	3H	142.15	bowman_contig_291958	TGCAGCACCCATATACTAACGGTGTTCTTGTCATGG TCATTGCAATAGATGCC[A/G]AGCGTGTCAT
TP51407	3H	142.15	morex_contig_52042	TGCAGGG[C/T]GTGTATTGATGGACGAGCGTAGGAT GGTGGCGTTGTCTGTCGTCGTGATGGCGTCA
TP57888	3H	142.15	barke_contig_517867	TGCAGTAAAGACAGGCA[C/T]GTAATTGTTGGATAG GCCAGCCACGTAAACCTAAGAAAAACTCAAA
TP59526	3H	142.15	bowman_contig_62187	TGCAGTCAAGCTTAGAAGATTCAGTGCAGTTCAG[A/ G]AGGATATCTCGCAATACACTTCTGTAAAC
TP6952	3H	143.45	morex_contig_138554	TGCAGAGCGGCCTTCTGCACGTAGAGCGCGACGCG AAGCTTTTCCTGTGGAG[A/G]CGCCACGAAAA
TP39512	3H	143.45	bowman_contig_221016	TGCAGGAAGCTGTCTGGGATGCTGACATTGTGGTGA ATGG[C/G]CTGCCGTCCACTGAAACAAGGGA
TP52309	3H	143.45	-	TGCAGGGGTCATTGAT[A/G]GTGCGATGTAAATTGT AATGCTAATCGGCGGTTGTCAGCGTTGCGAT
TP53073	3H	143.45	morex_contig_1558152	TGCAGGGTGGTGCGCAGATGCTGGTGTCCCAGATC GCCGTGGGAACTCTTATTGC[G/T]GTGAGGTT
TP54122	3H	143.45	bowman_contig_69819	TGCAGGT[C/G]AAGCTCAACGGCGTCGCAACCATCA TCGGCAAGATCGGGCTCGCCTTCGCGGTGCT
TP55561	3H	143.45	bowman_contig_850485	TGCAGGTGAGGAAAAACGGCAGGTGGTACCCG[T/C] TGAACCCGCTGCCAGGCGCCCTCGTCATCAA
TP5727	3H	147.34	morex_contig_347651	TGCAGACTTGTTAAGTTCAGTAGACATCACACGACC ACCTACCCTATTACAGAAGA[C/G]GCAGTAC
TP20423	3H	147.34	bowman_contig_292580	TGCAGCCAAGGCAGGGGCGGCGGCTGGGGAGGGG AGCTAGAGCTAGAGACGACCGATGGCCGT[C/G]
TP57414	3H	147.34	bowman_contig_292580	TGCAGGTTGCCGTCCTATCTTCGTTTGCCAGCGAGT GAGCTCGT[C/T]GGTCTGGTCTCCGAGATCG
TP58331	3H	147.34	morex_contig_44373	TGCAGTACCATGAGGAGCCAACTCTTTGAAACGTG ACAAA[A/C]ATAGACCACCGAGATCGGAAGAG
TP9788	3H	151.25	bowman_contig_203765	TGCAGATCGGGGCGGAGGAGGACAGGTTAGAAA[G/ T]ATACTAGCAGTAGCAGTGATAGGAGAGTGA
TP17747	3H	151.25	barke_contig_2785736	TGCAGCAGGCAGCAGAGTCCTCCTCCTTCTCCTCTG CTGCTTCTTGATCATCTTGGCGA[G/T]TCCC
TP29242	3H	151.25	bowman_contig_72607	TGCAGCGCTCTTCTTTGTCCTCCTCGCCCTGGCCACA ATGCTGCCGCAGACCGC[A/G]TCGTCCGAG
TP52851	3H	151.25	barke_contig_513111	TGCAGGGTGACAAGAAGGCCCCACGAGCAACGGTG CTGCT[C/G]CTCCTGGCCGTTCCGAGATCGGA
TP54021	3H	151.25	barke_contig_303223	TGCAGGTATCTGTCTGATATATCACCAGAAATGTCT GGTTGACTCCCA[C/G]GAATGAAGCACGGGC
TP40669	3H	152.55	bowman_contig_69031	TGCAGGA[C/T]GAGTCTGTATTGATATGGTTTTCAGG GAAAGAGGAGAAGCATCTAAGGTTGAGCCA

Marker_ID	Chr.	cM	Barley_Contig	Sequence
TP43983	3H	152.55	bowman_contig_872180	TGCAGGCACGAGTTAACGTGCATTTTGTTAACTTGC ACCTG[C/T]CGCGTTCTTCAATAGTCCGAGA
TP55761	3H	152.55	morex_contig_1568777	TGCAGGTGCAGCAGCAGCAGCAGAAGAAGCCCACC CATC[A/G]CACGGCCTAATGCGACTCGCTTCT
TP65212	3H	152.55	morex_contig_2547323	TGCAGTTGGAAGCCCCGAACGATGGACCAAAGGCG AAGGAATTCG[C/T]GGCAAACGCGCAAGTTGT
TP2055	3H	157.76	morex_contig_2554032	TGCAGAAGCACGGGAATTGCGCGCACGCATCAGCA A[C/G]CCTGGACCGAGATCGGAAGAGCACACG
TP3927	3H	157.76	morex_contig_40749	TGCAG[A/C]CATGGGGTTCTGCTGCGGGCCTTCGGA TGTCCAGGTGCTCCCAAGAACACCTCCTCAT
TP4241	3H	157.76	barke_contig_269491	TGCAGACC[C/G]TACGTCCACCTATGCTATGCGTGTT CCGATCAAACATGCAAGCACGTTGTGTTAG
TP11454	3H	157.76	bowman_contig_1983379	TGCAGATTGCCATTGAGCAATTCGAGCATGAGGCA GC[A/G]GAGGCGGCTCGGCTCGCCAAGCTCAA
TP13732	3H	157.76	-	TGCAGCACCAACACCGACGCGGC[A/G]GAGCCACAC CGTAGCACCGAGATCGGAAGAGCACACGTCT
TP23280	3H	157.76	bowman_contig_852007	TGCAGCCGAGGCACCAGAGG[G/T]CTGGCACGATTG GCCTATTGTCATCTATACCGAGATCGGAAGA
TP23694	3H	157.76	barke_contig_1854278	TGCAGCCGCCGCCGATGCATG[C/T]TGCACATGCAA TGATGTCCGTCGATGCGCCGCTGCTCCCCAC
TP24083	3H	157.76	bowman_contig_293952	TGCAGCCGCTGCCGCTG[C/T]ATGCTGCACATGCAA TGATGTCCGCCGATGCGCCGCTGTTCCCCAC
TP25393	3H	157.76	morex_contig_46889	TGCAGCCTTCTCATGCTCG[A/G]TTTCCTGCCGAGTT TTTCGCAACTCGGCCGAGATCGGAAGAGCA
TP28253	3H	157.76	bowman_contig_2004203	TGCAGCGCGAAGGCGTGCGTCTGGTGTGTTGGGAC AGCTCT[A/C]TCTCCCCTGACTAGACGGAGGT
TP31518	3H	157.76	barke_contig_269491	TGCAGCGTCACCACCGCGTGACGTTGCTCCAT[T/C]G CAACACTAAATGCCTCGTCGAAGTTCCATC
TP61630	3H	157.76	morex_contig_368179	TGCAGTGAGCATGAAGGCCTAATTTCTCATACACGG ATACAC[C/G]TCAGAGATAATTGCACCAGTA
TP63986	3H	159.06	barke_contig_294599	TGCAGTTATTCACTGCCATCTTTTATTTCTTCAAATG AATCGTGTGCATATCATTA[A/T]TGGGGGA
TP24268	3H	159.06	-	TGCAGCCGTCGCTCTCCACGGCGAGCTTGCATGCGA ACTGCGGCGC[A/G]CCTGGCGCCTTGCCGTC
TP32348	3H	159.06	barke_contig_1783305	TGCAGCGTTATAT[C/T]GGCCAAGGGGAGCCTAGCA ACGGAAAAGCTGCTCCAAATCCGATGACGAA
TP52612	3H	159.06	morex_contig_43211	TGCAGGGTCAACGGGCG[C/T]GGCTACCACACCACT AGGGCTAGCTACTTGGAACAGAAAGTAAGCA
TP58647	3H	159.06	morex_contig_369094	TGCAGTACTTGATCTGATCTGTTC[A/C]TCTTCCATC CAACAGGGTATCCTGGATGAGAAGGCCGAG
TP59767	3H	159.06	-	TGCAGTCAGCTGTGTAGCACAAAA[G/T]TCGCATCG TCCCCTATCTTCGGTTCTTCTGGTTTCCTCT
TP59820	3H	159.06	morex_contig_302872	TGCAGTCAGTTGT[C/G]CAGCACAAAATTCGCATCG

Marker_ID	Chr.	cM	Barley_Contig	Sequence
				TCCCCTACCTTCGGTTCTTCTGGTTTCCTCT
TP3814	3H	160.36	morex_contig_46051	TGCAGACAGTGGAAAGAG[A/C]AGACAGGGCCAAA TTAAAGATCGTGATCTTCCAATAAAACGTAAC
TP3980	3H	160.36	bowman_contig_981669	TGCAGACCAAGACGATGCGCGCCTGCTCGACCGTC AGTCACC[A/T]CGACACACGACAGTCGACAAC
TP9663	3H	160.36	morex_contig_139553	TGCAGATCGCGGTCTTCCGTGTATA[A/T]CTCACCAA TGCCGTAGTATATGCAGTTCCCGTCGACGG
TP9964	3H	160.36	morex_contig_46315	TGCAGATCTCGGGCACGTTACTAACCATGCTGCTGC T[C/G]TCCAAGTACCGAGATCGGAAGAGCAC
TP19717	3H	160.36	bowman_contig_201409	TGCAGCATGCGGGAGATACGAAGGTGGTCG[G/T]CT CGTTAGCGTGATTGATGGACCATCGCGGTTG
TP32769	3H	160.36	morex_contig_66533	TGCAGCTACGAGGGGCAAACAAGCTCAA[C/G]CTCG AAACGCACCCCGACGCCGCCCCGAGATCGGA
TP34193	3H	160.36	-	TGCAGCTCCTCTGCCTCGACCGAACCCCGACTGCCT CCCGTC[A/G]CGTCAGCCGCGGGTGCCGCCC
TP38267	3H	160.36	barke_contig_374155	TGCAGCTTGTCTAGTCCCTTTAAAAACAAGGA[G/A] ACGTTTTACGCCGAGATCGGAAGAGCACACG
TP45302	3H	160.36	barke_contig_540780	TGCAGGC[C/T]CCGTAGACGAGGCCCAGTACCCCCA CGACGAGCCAGAAGATATCAGGAGAGCCGAG
TP53308	3H	160.36	bowman_contig_145704	TGCAGGGTTTCATTTACCTTCACG[A/G]GCCTTCTCG CTGGATTTCATTGAGAGATGAGGAGGATGA
TP55942	3H	160.36	barke_contig_375360	TGCAGGTGCCTTGGGGTCCATGACAGGGG[C/G]CCA ACAGGTGGCTGGCCCACATGTCACTGGCCCA
TP61954	3H	160.36	-	TGCAGTGCAGAGCAG[G/T]TTTGTTGCATTATATTTT CCTGTACCACTTTTCCTGTACATGCCACAT
TP11854	3H	162.96	bowman_contig_129429	TGCAGCAAACCTCACCCAA[A/C]CCATCGTGCCCTC CTCTTCCTCTGTCTGCGCGAACCCTAGCCAC
TP2656	3H	162.96	bowman_contig_1997223	TGCAGAAGGTCATGCAGTAGGCGCAGAAGGCGAGG CACTTGTTGGGGCAGGTCTTGGGGCA[G/T]GA
TP5583	3H	162.96	-	TGCAGACTCTGGACGTCAGGGGAACATCCATTGTTA CCATGCCAGAAAGCATCATCAATCTAC[A/G]
TP5623	3H	162.96	bowman_contig_63211	TGCAGACTGCGACGTCATCGGTGCTAGTGATGGCG GC[C/G]AGGATACAACTGTTTGAAGTTGGCGG
TP5731	3H	162.96	barke_contig_276490	TGCAGA[C/T]TTTACATCATCGGTCGCCTGAAACGG AAGGTGTCACCTCAAATAAAAAGCATGTTAT
TP12906	3H	162.96	bowman_contig_898075	TGCAGCAAGGCGCATGAGGCAACTGATAGATCCCG TGGATCGTCAA[A/G]GCTAGATGTGTTCGGTG
TP20360	3H	162.96	bowman_contig_223262	TGCAGCC[A/G]ACTCAGAGCAGATAGAGTTGAGGAT GGCCAGCTGCTCCGAGATCGGAAGAGCACAC
TP30778	3H	162.96	bowman_contig_871968	TGCAGCGGGATGACAAATTCCACGGGCAAGTGGGT GGA[A/G]ACCACAAGCTTAGGCGACCATGCCT
TP32515	3H	162.96	morex_contig_1570908	TGCAGCTAAACAAACAGTTACCACGGTTTATCAACC ATGACAACTGTCCC[G/T]CAGGGGCACGCGG

Marker_ID	Chr.	cM	Barley_Contig	Sequence
TP37215	3H	162.96	morex_contig_41573	TGCAGCTGTGGTGGC[C/G]ACTTCACCTGGGAAGATCCCACCACCTGAGGTGGTGGGTCCCTTCCCT
TP48815	3H	162.96	bowman_contig_63211	TGCAGGCTGAGCCACCAGGAGGCT[A/G]GGTGGTCGACAAGGAAATATTTTCAGGACCGAGATCGGA
TP49201	3H	162.96	bowman_contig_871968	TGCAGGCTTCATCGACTAGTTCAAACACCGTGAAAGAGGG[G/T]CTGCGGTCAAAATCGCTGGTCAC
TP54019	3H	162.96	bowman_contig_884775	TGCAGGTATCTCCTCTCCCTCCTCTCTTTCTACTCCCTCCGT[A/C]CCGAAGTTAATGTCGGACGC
TP58758	3H	162.96	morex_contig_134322	TGCAGTAGCAACAAAAGTTCAAGACATTAAGTAACCAAAGTAGAGCTACA[A/G]ATTATGACTGTAA
TP59794	3H	162.96	bowman_contig_200541	TGCAGTCAGTC[A/C]ACGACTCCACGTACGAAAACTCAAGCAATTAATTAGTTTACACATGGAGGTG
TP61741	3H	162.96	morex_contig_172232	TGCAGTGATCTGACCCGAGGCGGCCGCGACGGCGA[C/T]GCGGAGAGGAGTGCCGCCCCGCAGAGTG
TP62029	3H	162.96	morex_contig_66933	TGCAGTGCAGTGCAGTAGTACTAGGAGGTGGTGCAA[A/C]GACATTGCAGAGGAACAGCTAAATGGT
TP57029	3H	162.96	bowman_contig_944991	TGCAGGTTCAG[A/G]AAAAGTGGAGTAGTTGGTTTCGACAGAGAGCAGATTTATCTACGCGTTGGCG
TP51873	3H	164.26	morex_contig_134629	TGCAGGGGCAGCAAGTTAAGGGTCAAGGAACAGGGCAGCATA[A/G]TTAAAAAGAAAAGATAACCAA
TP15152	3H	164.26	morex_contig_307995	TGCAGCACTGCTCTTAGCCCTCACGTCCGCCAT[G/T]AACATCACTGCCCAGAATGACGACGCTGAC
TP26385	3H	164.26	-	TGCAGCGAGGGGCACGTCAGTGATGATTTGTTGGGGTCGC[C/T]TATGCTGCATTGCAACATGACGG
TP28422	3H	164.26	morex_contig_440286	TGCAGCGCGATGGCGGGAGCTTTACGGGTGTGGAGAGCCTGGTCGGGCCT[A/G]TGGGCCCACGAGC
TP39636	3H	164.26	-	TGCAGGAAGTCAAGGGAAAAGGTTTCCTGCTGGTTTCTAC[G/T]GACATAGCGAGCAGAGGGTTTGA
TP55422	3H	164.26	-	TGCAGGTGAACTAGCTAGTAACCCTAGTCAAGTCAAAAAAACTCCCTCT[A/G]TCCTGAATTACTTG
TP10237	3H	165.56	bowman_contig_1989888	TGCAGATGAGTAATTTG[C/G]TTATCCTGAATCTGTGATGTTAGATGGAGCCCTGGACATGTACTCT
TP11522	3H	165.56	morex_contig_2555775	TGCAGATTGGTCACTG[A/G]CCTGGTTCACACTTAATTGGCTACTAAAGTACTAGCTGCCATGGCAG
TP12420	3H	165.56	bowman_contig_200926	TGCAGCAACGCGCGGGGCATC[A/G]ACTAGTTCAAGGAGTGAAGGCTCCAGTTCCAGTGCATACGAG
TP13362	3H	165.56	bowman_contig_16057	TGCAGCACAACTTTTCTGCATTGTCCCTTCTAAGATCGTACAATTAAGCTC[C/G]CTACTACTGCTA
TP22181	3H	165.56	-	TGCAGCCCCAAAGAGGGAATCTTCA[C/T]AGTGCATGTTCACGTCATCTCGATCAGCTAGGTTTCTA
TP26210	3H	165.56	-	TGCAGCGAGCATACAGTGCATTTGTCGT[C/G]TGTTTTGGTCTCCACCGTCTAATCTCCGAGATCGG
TP41495	3H	165.56	morex_contig_132528	TGCAGGAGCAAGATGGCGCATTTCATGGTGCCTAG

Marker_ID	Chr.	cM	Barley_Contig	Sequence
				GAA[A/G]GTGGTGTTCGTCGATGAGCTGCCCA
TP42420	3H	165.56	bowman_contig_894189	TGCAGGA[G/T]GTTAAAGTCCAGCTTTGCAAGCTCC AAAAGAACTGGATTATGGCCTTCTTCTTGTT
TP42744	3H	165.56	bowman_contig_62467	TGCAGGATATAGG[A/G]TCGATGCCGCTAGCTTTGC ATCAGCATTGGAATATCATGATGCTGCATTC
TP58733	3H	165.56	bowman_contig_71010	TGCAGTAGAT[A/C]CTTTGCGTGTTGTGCCTTTTTTG AAGCAAATTCCTTTTTCCTCATGGCATTCA
TP4256	3H	166.86	bowman_contig_63375	TGCAGACCCTGCTAACAAAACGGATGAACTCATCTC [C/T]GACAGATCTGTGGAATAAAGGACATAT
TP16637	3H	166.86	bowman_contig_200279	TGCAGCAGCCGCCCTCGCTGCGGCAACGGTCTCTTG GCCGCCAGCTGCTCGAGCACCG[C/T]CGAGG
TP35586	3H	166.86	morex_contig_135201	TGCAGCTGCAACCAGAGAACCATCATGAGACATTC CACCA[C/T]CTACATAAATCTTTGTGTGGCCG
TP48019	3H	166.86	bowman_contig_200279	TGCAGGCGGTGATGGAGGCGGGGCCTCT[C/G]CTGG AGAGCCTGCTGGTGGCAGGGCCTGTGCCGCA
TP59048	3H	166.86	-	TGCAGTAGTGAGGAGGC[C/T]TTTTTTTAGAGAAG GAGGTCAAACCCCCGAGATCGGAAGAGCACA
TP63677	3H	166.86	barke_contig_274949	TGCAGTTAAACAAACATGGAAGAGGGG[C/T]CGTTC GGAGGTGAGGGCGCGTGCTGACTGTCACGCG
TP65122	3H	166.86	morex_contig_1559531	TGCAGTTG[C/G]TACTGTAGTAAGTAAGCTATAGTT ATGCATATCTTTAGATGGTGCTCTACAAGCT
TP66051	3H	166.86	morex_contig_96865	TGCAGTTTTCTTGGTGGTGGGCGAGACCGAGAAGA GTGTTGATGCTGCTGCTGCTGCTGCTG[C/G]T
TP3588	3H	166.86	barke_contig_479624	TGCAGACACAACCAAATTT[C/T]TGAAAAAAAATTG CTGCCAAAAGAAACCAAGCCCGTGATGGATC
TP35466	3H	166.86	bowman_contig_1983915	TGCAGCTGA[G/T]GTGAAGTATGAAGAAAAGCTTGC TTCATCTCTCTTTTTTTCATCATGTGAGATG
TP39318	3H	166.86	bowman_contig_67331	TGCAGGAAGAGAAGATGATCCTGCCCTCACAATCT CCAAGGATCATACCAGCTCCAGCGCC[A/G]CC
TP61356	3H	166.86	-	TGCAGTGAAATGGATCGATCATGAGAGTAAGATTA ATTTTACCGATGACCGAA[C/T]TGCCCGTGTG
TP64084	3H	166.86	bowman_contig_891926	TGCAGTTCAGAAAAAGGTG[A/G]AGCGGACGAGCA GTTGGTTTCGACGGAGCAGATTCATCTGCGCG
TP19755	4H	51.00	bowman_contig_10798	TGCAGCATGGAAGGCCTGAGCTCCTCACACCGTAA AAATGTTTGCTTGACTG[C/T]TGCATCATGAT
TP36955	4H	51.00	-	TGCAGCTGGTGGAGCAC[A/G]GGATGATCGTGCACA AGTCGTGGAGGCTGCCAGCGGAGAATGAGGC
TP27632	4H	51.00	-	TGCAGCGCCGAAGGTGGTATCCACCTCTCCC[C/T]C GCCTCCCCCCTTCGGCGGGAGCGGCGAGGTC
TP65670	4H	51.00	bowman_contig_190290	TGCAGTTTCAGTAGTCAGGTTTGGGTACAGTTTAGG [A/G]CATGGGCAAGAGCTGATTTCCTGATTC
TP63833	4H	51.00	barke_contig_272404	TGCAGTTACTA[C/T]GAAGCTTAGGCAGGAATGGAC ACTGTTCTTCAAGTTCAGTAGAGTTTTAGCT

Marker_ID	Chr.	cM	Barley_Contig	Sequence
TP61460	4H	51.00	bowman_contig_846860	TGCAGTGAATCCAAACACACAAATTTATGCAACAG AGATCTATCGTTC[C/T]GAGTGCTGTAGAAGA
TP54367	4H	51.00	bowman_contig_223778	TGCAGGTCATGTATGGCGGTAATGCAATCCTT[A/C] AGCTCGGATGCCCTGTACCCGAGATCGGAAG
TP52493	4H	51.00	-	TGCAGGGGTTCGAAC[A/C]CGCAACCTGCGGTGTGG AGGCACGGTGCGGCTGTCATTGCGCTAGGAA
TP47368	4H	51.00	barke_contig_269639	TGCAGGCGCTCGTTTGCAAAATTGCATTGCAACGGA TGCTTGTGACGCCAAATAG[A/G]TTTTGCCA
TP45314	4H	51.00	-	TGCAGGCCCGACACCAC[A/G]CCATGTGGCACTACG CGGTGCCTTCGGACTCCACTCAGTCTCATCC
TP41164	4H	51.00	morex_contig_188347	TGCAGGAGAAGATGGCCAAAGCCAAGCGGGACGTG GACGCGGAAGCTGCCAGGATGAC[A/G]GCACA
TP37971	4H	51.00	morex_contig_136802	TGCAGCTTGCACGGCCGCCGTCGCGTTGCAGTCAAG CCCAAACAAGCTGC[G/T]GTGATTTGACGTC
TP36917	4H	51.00	bowman_contig_235704	TGCAGCTGGTCGCGTCAGCGGGAAGC[C/T]GACGGG ATGGATGGAACCGAGATCGGAAGAGCACACG
TP34952	4H	51.00	barke_contig_1570100	TGCAGCT[C/T]GTTGCGGTGCAGATGTTGCTCTGTCC CTGGTCCGAGATCGGAAGAGCACACGTCTG
TP33382	4H	51.00	bowman_contig_125160	TGCAGCTCACAAAGGCTGCTGGTACCGTCTCAAATG TCCGTCTGTTTCGTGGCTGAACAC[A/C]TCT
TP29979	4H	51.00	bowman_contig_196689	TGCAGCGGCATGCCACGAGGAGGACGTGGCCAAGA CAACAACGGC[A/G]ATGGAGGCTCCAGAGGTG
TP24692	4H	51.00	barke_contig_1839671	TGCAGCCTCCCGATCGCACCGCCTGCCGCCCTGGC[C/T]GCCACCGCCTCCAGGGGTGCTAGCCGAC
TP22291	4H	51.00	morex_contig_345976	TGCAGCCCCCCTGGTGGCAGTGCTCCTGGCGCTAGG AGCAGGGGCCGATGGGGCTGGTTTG[C/T]TG
TP21580	4H	51.00	-	TGCAGCCAGTGGTAGTACCGCTGGAGTGCCAGC[A/ G]GTAGTACCGCCGAGATCGGAAGAGCACACG
TP19038	4H	51.00	morex_contig_1559081	TGCAGCATCAACATTGCGAACGGAGTCATG[A/G]CG ATTCGAGACCCCTCGCGGTGTATGATGGCGC
TP18416	4H	51.00	morex_contig_8401	TGCAGCAGGTGGTGGGAAATAATCGTAATCACTGG CACCCTTTTGGCACACGTCGG[C/T]TGTTGGT
TP17174	4H	51.00	-	TGCAGCAGCTCACCCAGGTCAACTTGTTTTATCCCA AGGATCGAGA[C/T]CGAACTCCCGTCTCAGG
TP14801	4H	51.00	-	TGCAGCACGGCGTTC[C/G]CCGCAAGATGGTGCGGC TGTAGGCTAAAGTGTGCCAAGAAGCTGGAGA
TP14615	4H	51.00	-	TGCAGCACGCCTCATGAGATCGCGAG[A/C]AGACAC TCCTAGGCTTCGTTGGGCCCGAGATCGGAAG
TP14247	4H	51.00	bowman_contig_860704	TGCAGCACCTCCTTGCAGCAAGTCGCCGATCGTC[A/ G]CAGCACCGTCTCGCAACCCATCGTCGGTG
TP14005	4H	51.00	morex_contig_91117	TGCAGCACCCGCCGCCGTCGCCC[C/T]CGTGCTGCG ACGCCCTCGTCCCCGACACGCCTCCCGAGAT
TP12755	4H	51.00	morex_contig_1571157	TGCAGCAAGCATTTGAAACCTATCTACCT[C/G]CCTT

Marker_ID	Chr.	cM	Barley_Contig	Sequence
				AGATAATATGAATATTCTCCTATTTAAGCT
TP7867	4H	51.00	bowman_contig_290955	TGCAGAGGTGCGTATAGGAGAATCAGTCTGTAGTA TGGAAG[A/C]GGACATGCGTCGCCGAGATCGG
TP3206	4H	51.00	-	TGCAGAATGGAGGGGAGTGGCTCGGTA[C/G]ACACG GAGAACTCGCGGCCATGGTTGTGGCCACACC
TP1938	4H	51.00	morex_contig_40547	TGCAGAAGAGGTCAAATGCATCAGACTTGCTCAAT GGCTCGAGCTCAAGGTGACG[A/G]GTGGGAGA
TP22263	4H	51.00	morex_contig_1576868	TGCAGCCCCATGGTATGGAGGGACTCGCAGCGCGA GATCACCCTT[C/T]GGTGTTCGTCTTTCCCGA
TP51979	4H	53.60	bowman_contig_144774	TGCAGGGGCGCGG[A/T]GGTCGGCCATGGGTCGTCG TGAGGCTGGAGCGGGGGCGGCAGGATGATCG
TP30013	4H	53.60	-	TGCAGCGGCCAGACGAGCGCGGCGGTTGCCGCAGC TGTGGCGGTTGCCAGGGCAGCCA[C/T]GACGG
TP11052	4H	53.60	bowman_contig_148663	TGCAGATGTGTTGTTGCTAGCCACCCCAGCAATTGG TGCTAGCTATT[C/T]GTCGGTTTCGGCTGAT
TP59687	4H	54.86	barke_contig_370058	TGCAGTCACTGGTGCACGAACAAGAGCCACGAGAG AAGGTGGTGACGCA[C/G]CGCAAACGCCTCCG
TP38222	4H	54.86	bowman_contig_128628	TGCAGCTTGTACAGTACCATAGTA[G/T]CATTACAGT ACACTTGGTGGTACAAACATAATAGCAGCT
TP28571	4H	54.86	morex_contig_1558498	TGCAGCGCGCGCAGCCACCAAACGGGGTCTCGGA[C /G]GTGCTGCCTGCCATCTCGTCTGCCACCCC
TP23343	4H	54.86	morex_contig_41456	TGCAGCCGATGGCGAGGATGATCATTTCCGAGAAT CCCAGTGAGTGAGTTGT[C/G]ATGACATGTGT
TP21080	4H	54.94	-	TGCAGCCACTGCTCCAGGCAGGCC[C/G]AATGGAAG ATGTGCTTGCAGCGCAGCTGGATCAGCCTGT
TP46986	4H	82.98	barke_contig_272322	TGCAGGCGCC[A/G]CTTTACGAAAATGCATACGAAT GAAGGCCTGGAACACTAAATAGGAAACACTG
TP62017	4H	83.00	bowman_contig_844574	TGCAGTGCAGTGAATCAGTCACATGCATGCCATAGC CTT[A/G]TCATCTTTTCCGTGTAGATGATCC
TP26370	4H	129.60	-	TGCAGCGAGGCTGCGTCGTCCACGCCACCTTGAGAT CCCTCCGTAAGTGAACCTCCATCTCCA[A/T]
TP59956	4H	129.60	morex_contig_87276	TGCAGTCCAATGAAGCGCTAACTGCTAAGCATACCT GAAGCAAT[C/T]GCCTTAGCGGCAACCTTGA
TP8649	4H	129.60	morex_contig_87276	TGCAGATACTGTAGTGGGATC[C/G]TTGGATTCTCTT TGTTAGGATCCTAATTGTGAGCACAGTGAG
TP53065	4H	129.60	-	TGCAGGGTGGGGTCGGGCCTGTAACGGGGC[A/G]TC GGCATGGCCGAGATCGGAAGAGCACACGTCT
TP39658	4H	129.60	morex_contig_367754	TGCAGGAAGTCGCCGACGTTCACCACCAGCGACGC CTCCCCC[A/G]CCGCCGAGATCGGAAGAGCAC
TP859	4H	129.60	barke_contig_276869	TGCAGAAATTGGATGTACCTATCTTTTTAGAAAAG GAGGA[C/T]GACCCCCGAGATCGGAAGAGCA
TP287	4H	129.60	-	TGCAGAAACAATAGTCAGATTGGAACCCCCCTCTCT TGGCGGTAGGCGCGCCAGGTGA[C/T]ACGTG

Marker_ID	Chr.	cM	Barley_Contig	Sequence
TP59213	4H	138.70	barke_contig_269320	TGCAGT[A/T]TCGGACAGGATGTTCCTTTCGATAGTC TCTGTGGATACCGAGATCGGAAGAGCACAC
TP61055	4H	138.70	bowman_contig_11063	TGCAGTCTACTTACGTGTCGAGTACTCGAGTACAGA GGGAGGAGCTTGCACTGCTTCC[A/G]CCCAT
TLP3	5H	0		GCTTCTACCACTACGGCATCACTAC[G/A]GACAAGG GGTTCAACCTGCCGCTGG
TP54118	5H	1.00	bowman_contig_1998759	TGCAGGTCAAGATGTTTTCTTACAACTAGTACAAA TGC[C/T]CGTGCGTTGCACCGAGATCGGAAG
TP63590	5H	1.00	bowman_contig_219311	TGCAGTGTTATTGTAAGTCAGCCCTAAGCTGAGAAG GTAT[C/G]TTTATAAGGAGACTAGATCGACG
TP13528	5H	2.07	bowman_contig_10320	TGCAGCACAGAATTTGTAGTGTTTGTAT[A/G]GAAA ACAACTACAAATGTTTCAAGGCCATTATGTT
TP3726	5H	3.37	morex_contig_141545	TGCAGACAGACAGATACTAACACATGATGAACTTC AAGTGAGCAGCATGTATC[A/G]CGATGAATGG
TP12039	5H	3.37	bowman_contig_221981	TGCAGCAAATCGCGACGGCAGTT[A/G]CAGCTAGAT GGGACACTGGTGGTAGCAATTTTCAATGCCG
TP24182	5H	3.37	morex_contig_141545	TGCAGCCGTAAGCGGCCACATGTG[A/G]CCTTGCCT AGTTCTTGTTCTGCTTCTAGTTCTTGTTCTA
TP59917	5H	3.37	barke_contig_269588	TGCAGTCCAA[A/C]ACCAAAATGGACGAAAGTTGTG CCTACTACAAGATTCGTTTTTGGATATTCC
TP62237	5H	3.37	barke_contig_2786554	TGCAGTGCCTCGGCGATGTTGTGGTAGAAGGATGC ATCGT[C/G]AGCAAGGGCTCCGTCAGAGGGAG
TP38608	5H	4.67	morex_contig_73732	TGCAGGAAAATATAGAGAAGCAAGGACACTAGTGT CTC[A/C]GCGTGGATCGCTGACTCTATCCCAA
TP54510	5H	4.67	bowman_contig_1984153	TGCAGGTCCGACAGAAGGTGTGGATAATGCT[A/C]A GCAACAATTCGAGAAAACTCCGCTTCTTTGA
TP1392	5H	4.67	bowman_contig_144877	TGCAGAACGACACCTGGAACGGCACCTCCCCTCAG CA[C/G]GGCACGTACGCGGCGCCGAGATCGGA
TP41194	5H	4.67	bowman_contig_67402	TGCAGGAGAAGTCCATGGGGACGTTATACCCATCG ATGACCGAGATGT[C/T]GTAGTAGTCCTGCGA
TP48109	5H	4.67	morex_contig_1582097	TGCAGGCGTAGACGGGAT[A/T]TTTAGACGAGACAC ACGAGGCCGAGATCGGAAGAGCACACGTCTG
TP497	5H	12.52	barke_contig_371009	TGCAGAAAGAC[A/C]GAGTAGCTAGGCCTTACGCAG CGCCGAGATCGGAAGAGCACACGTCTGAACT
TP33438	5H	12.52	bowman_contig_13818	TGCAGCT[C/G]ACGATACGCCAGTTGAATCCAGCAA GCGCGACGTGCTCCGATGACAGCGGCGAGGG
TP43934	5H	12.52	bowman_contig_865713	TGCAGGCACCCGTTTGCGCTCTTGGCGCCAGCAGCG CTCCA[C/T]GCCGAGATCGGAAGAGCACACG
TP38150	5H	13.82	morex_contig_60728	TGCAGCTTGGCGCTGGCGCT[C/T]TGTTGGTCGCTGA AGCGGCCGAGATCGGAAGAGCACACGTCTG
TP32708	5H	15.13	morex_contig_46634	TGCAGCTACCATGT[C/G]AGTAGGAAGATAACAGGA TCTTATCCGAGATCGGAAGAGCACACGTCTG
TP63447	5H	16.42	barke_contig_282158	TGCAGTGTGAAACTTCCACAAAACTGTCAATTTCGC

Marker_ID	Chr.	cM	Barley_Contig	Sequence
				ACCGCGCCC[A/G]CCGCATTCATTCCGAGAT
TP21886	5H	16.42	morex_contig_67868	TGCAGCCATTGCTCCTACGCCTCTACTGGTGGAGTT TATACTTTTCACCGTCTAAAACGGGTG[A/T]
TP7774	5H	16.42	morex_contig_1581968	TGCAGAGGGGGCGGGTCGATGTATGTATATCTGGCT TTGA[C/T]GAGGAACCGAGATCGGAAGAGCA
TP12346	5H	16.42	morex_contig_72763	TGCAGCAACCTATTAAGCGTCATGGTTGTTTGATG CCACACCTGAGTACT[A/G]TGTGTTTGGTTT
TP22738	5H	16.42	barke_contig_282309	TGCAGCC[C/T]GCCGCTTCTTTAATTGCAGTATCATG CACTGACCGAGATCGGAAGAGCACACGTCT
TP37374	5H	16.42	morex_contig_65198	TGCAGCTTATGTAATGGTTTTACTTTGGTTGGAACA T[C/G]AGGTCTCTGCTTCTAGTTCATCCCAG
TP64344	5H	16.42	-	TGCAGTTCCTCTTTAATTC[A/C]AGACCTGACCTGGT AAGGAGTACTATTAAACCGAGATCGGAAGA
TP11725	5H	19.03	morex_contig_56495	TGCAGATTTTTTCTTTACTCACAATGATTTCAA[C/T] TTGTTTGTCGTGAATAGGAGAGAACTTGAT
TP31687	5H	19.03	bowman_contig_861463	TGCAGCGTCCTCGGCGTAGGCTACGGCGGAGGCGG GGCGCTC[C/G]AGGCGGCAGGTGAAGCAGCGG
TP3371	5H	20.31	morex_contig_83442	TGCAGAATTGTCGTTGTACAATCTTGAGCGCTAGCA ATGTTTCCTTC[G/T]TGTCTTTTCCTTGTAG
TP3372	5H	20.31	morex_contig_83442	TGCAGAATTGTCGTTGTACAATCTTGAGCGCTAGCA ATGTTTTCCTCGTGT[C/T]TTTTCCTTGTAG
TP10347	5H	25.54	bowman_contig_62910	TGCAGATGCACTCGATCCACGACC[C/T]GACGAATG CGGCGACGACCGACGATTAGTCACTACGGCC
TP41431	5H	25.54	bowman_contig_924366	TGCAGGAGATGATTGACGGAGGAGGAAGAGATCAA TA[C/T]GGAGATGGGCCTTGCTGCTCCACGTC
TP59966	5H	25.54	morex_contig_1592368	TGCAGTCCACAGTAGCCGCTGCCCATGTGC[A/G]AG GACAGAAGTCTACAGTAGCCAGCACACCCAT
TP16940	5H	29.44	-	TGCAGCAGCGGAGACAACACATG[C/T]CTTTACAGG GTACCCTACATGGACCGAGATCGGAAGAGCA
TP38718	5H	29.44	barke_contig_4003	TGCAGGAAAGAAAAGGCTTTTTATGCTCTGTTA[C/T] CGATACTGTTTGCTCCTGCAAATCTAAACA
TP39357	5H	29.44	morex_contig_80211	TGCAGGA[A/G]GATATATACGTATGGAGCTTTTGCT GACCCAATGGATTTGGAGGACATAGGTGTAT
TP41281	5H	29.44	barke_contig_1799546	TGCAGGAGAGACAGATTCAATCATTTCGAGTCCTGC GGTCACCTTTTGAAAAGGG[A/G]GAAAACAC
TP44740	5H	29.44	morex_contig_37437	TGCAGGCCAAGCTATTATGTACACCCAGATTTTATT TTCTGCACCGCGAATGAGGATGCA[A/G]CAC
TP49057	5H	29.44	morex_contig_1561753	TGCAGGCTGGCG[C/G]ACCTGCATGCATGCCGTCCG TCCATGTTTTGCACGAACCCCGATGTCGACG
TP65258	5H	29.44	morex_contig_48893	TGCAGTTGGCCA[G/T]AGCCGTCAACTCTGTATTGG GGTTGCCAGCTGGAAGGTTGTACCGCGCGCT
TP22385	5H	30.74	barke_contig_1799546	TGCAGCCCCGCACCTTCTTCCCGC[C/T]ATTCGGCCA CGCCACCCCTTCCACCGCGGACCTCAAGG

Marker_ID	Chr.	cM	Barley_Contig	Sequence
TP8224	5H	32.04	morex_contig_1559060	TGCAGAGTGTGTCACAACGCAGCCCTCACGATCAA CGCAGTCCTCAGGTC[G/T]AGGCTCTTGGACC
TP23771	5H	32.04	bowman_contig_900313	TGCAGCCGCCTCGAAGGAAAAGACTGGTGCGAGAC GACACGGCATAGTTGCCCCCA[A/G]AGAGCTC
TP30417	5H	32.04	morex_contig_125350	TGCAG[C/G]GGCGGGCCCATGGTGCACACGATGTTT TCTATGCACCAACAGAGTTATGGCGGATGCT
TP31354	5H	32.04	bowman_contig_900313	TGCAG[C/T]GGTTTCAACGCGTCGGCGGGCGTCGGA TGCAGCGTGCCCGTGGACGTCACGTCCAGGC
TP31606	5H	32.04	morex_contig_1592149	TGCAGCGTC[A/G]TTGAGGTTGTTGCGGTAGCAACA AGATGAACGAGCACCGAGATCGGAAGAGCAC
TP34330	5H	32.04	bowman_contig_128296	TGCAGCTCGACAGTGCTCCATTGCAACCCCAGGACG CTGCAATGA[A/G]TCCCGACGGCGCTTCAAT
TP37289	5H	32.04	barke_contig_275310	TGCAGCTGTTTTGTAACTTGTGTGAATC[A/G]AATTC TGTATTACTCTGTACTATGAATCTACGATA
TP51233	5H	32.04	barke_contig_1884202	TGCAGGGCGGCGCGATGACTGGCGGTGGCAC[C/T]C TGGGTGGGCCTGTCGTGGGCTTTCCGACGTC
TP55675	5H	32.04	bowman_contig_255014	TGCAGGTGATGTGGGCTATGGGCGGCCGCATG[G/T] CGCCGAGATCGGAAGAGCACACGTCTGAACT
TP57315	5H	32.04	barke_contig_2782335	TGCAGGTTGAG[A/G]GGCTTGGTGGCGAAGAGAAGC CGTCGGCGTCGGGATCCCTTCCTCGTCCATC
TP63562	5H	32.04	morex_contig_52357	TGCAGTGTGTGTGCCGTCGTTTCCGCCACC[A/C]CCA GCACAAGCCACACATTCGTCTGCCGAGATC
TP54285	5H	34.72	barke_contig_1824120	TGCAGGTCAGGTTTCGATCCCACACGTTTCCA[T/C]T ATTATTTCTACACAGAGTCCAGGCCCAACC
TP4814	5H	35.95	-	TGCAG[A/G]CGCACTACAAGCGGAGGCACTGTGCCA AGCCATTCCTCTGCCGCAAGTGCGGCAAGGC
TP5901	5H	35.95	barke_contig_2781259	TGCAGAGA[C/T]AAGGACCTCACACGGCAAAAGCCA CTCACAACCGTGTAGCCCCGACCCGATACCT
TP8541	5H	35.95	-	TGCAGATACAGGTAAAATTATTTTATTCAGTGATGC CAAAAGAACGTC[C/G]AAAGAGTATGCCCGA
TP11256	5H	35.95	barke_contig_315490	TGCAGATTCCACTTGGCG[A/C]TTTCCTTGTGAATTC ACTAAGGGTGTCTTTTGCAGTTGGTCAAGC
TP12019	5H	35.95	barke_contig_2495902	TGCAGCAAATACCTAGCGCCACCGCTAGAAGTTTCG TC[A/G]CCGCGAGGAACAGCTTCACTACCTC
TP13055	5H	35.95	barke_contig_265532	TGCAGCAATAATGGAAGCCCGACGATGGGGGC[G/A]GGCGGAGGCACGAGTCCGACAATGTTCTCTT
TP13091	5H	35.95	barke_contig_1860061	TGCAGCAATATTTAAAGTGAGGTCACGCCCAGAGA TCCTCCCTCGACTCC[C/G]TGCAAGGAAGCAA
TP13232	5H	35.95	-	TGCAGCAATGTCTTT[C/G]GAAGAGACAGAAGCTCC AACAAATATCTCTACGTTGGTAGCCGAGATC
TP17485	5H	35.95	bowman_contig_220994	TGCAGCAGGAAATACTTCT[A/T]CACTTGACTTTTCT TGAACCGAGATCGGAAGAGCACACGTCTGA
TP20697	5H	35.95	bowman_contig_882388	TGCAGCCACCACG[C/T]TCCGCAACCTTTCACCGAG

Marker_ID	Chr.	cM	Barley_Contig	Sequence
				CACCAGCGGGAGGAACTTCGGTGGAGCTACG
TP24994	5H	35.95	bowman_contig_143375	TGCAGCCTGAGGAGGACGCC[A/G]AGCAGGTCCTCA TCTCTCTCAGGCGCCGAGATCGGAAGAGCAC
TP25506	5H	35.95	-	TGCAGCCTTTTTAATCGCACTGGAA[G/T]AGCGCAA GATCATGTGAAATGAAACGTACCAGCCGAGA
TP26591	5H	35.95	morex_contig_1598031	TGCAGCGATG[A/G]CGTATTATAAGCTACTGTTTGGT AAAAAAGGCTGCTGCAAATTAGAATTGGAA
TP32400	5H	35.95	morex_contig_52950	TGCAGCGTTG[A/C]CCTTCCCTGCCACGGCGTCGGC GCCTATCGTCGCCCGAGATCGGAAGAGCACA
TP32436	5H	35.95	bowman_contig_861370	TGCAGCGTTGGGGCTGGGTGCATGGTAGTAGCATA GCTAGGCTCGGTTCCTTTCGCGTCGGG[A/G]T
TP35895	5H	35.95	barke_contig_8115	TGCAGCTGCCGTCGCTAGCAGCACAT[C/G]AATGGC AAAGGCATCCATCGTACATGATGGTTGATGT
TP37689	5H	35.95	bowman_contig_10137	TGCAGCTTCGTCTT[C/G]GGCGTCGCGGAGCTGGGC GTCCGCAGCCAGGAGTGCCGCGACCACACCA
TP41021	5H	35.95	bowman_contig_1991579	TGCAGG[A/G]CTCGAGGCTTTGGATCGGAACGACGG TGCCCGCTGGTGCACCCTGGACGTGTGCGGG
TP43149	5H	35.95	-	TGCAGGATGAGGTGGAGCTTCAAGATGGGGC[A/T]A TAGCATGATGTTACGGAGCTTGGTGAGATCC
TP48096	5H	35.95	-	TGCAGGCGTACAGCCAAGTCCACCATGAGCTCCCG CGGGAC[A/G]CCTTCTGCGCCACTGTTATATA
TP59839	5H	35.95	-	TGCAGTCATATAACTTGCCTATGCATGCAAAACG[A/ T]TCACTTCTTCCGAGATCGGAAGAGCACAC
TP65348	5H	35.95	-	TGCAGTTGGTACGCGCCATGGT[C/T]GCGTCGACGG ACACGCACGGCTGCGTCGAGCACGGCACGGT
TP20247	5H	37.24	barke_contig_270304	TGCAGCCAAATAAAATGAAACCACGAATTCAAAGG ATGTGTGCCTCAGA[A/G]TATCACGCAGTGGA
TP37715	5H	37.24	barke_contig_129454	TGCAGCTTCTC[A/G]AGATGAAGCTCGGCTAGCAAG GCACCCTGTGGGATCGCCTGGAGAACAGGGT
TP54027	5H	37.24	barke_contig_2785050	TGCAGGTATGAGCTAGGTAGGCCCGCTGGGGTCTTC ACTCTTTTGAC[C/T]TGGGAGCATCAATCAT
TP55268	5H	37.24	morex_contig_41158	TGCAGGTCTCCACCGTATCAAACTCC[C/T]TTCTGCC ACCGCTATCTTCCGCACCGAGATCGGAAGA
TP11893	5H	37.24	morex_contig_2547773	TGCAGCAAAGAACCTTGTCCTCGCGGGTGTCAAGTC TGTAACCTTGCA[C/T]GATGATGGTAATGTG
TP24336	5H	38.53	barke_contig_1968067	TGCAGCCGTGGAGTTAACCGTGC[A/G]TGAGACCAC AAAGGGATGCAATGGGTTACCGAGATCGGAA
TP13185	5H	40.19	morex_contig_216174	TGCAGCAATGCTAGCTCCTCCTTGGCCTCC[C/G]CCT CGCCTTGGCGGGCGACGTCGAGGGCGGGAT
TP12521	5H	41.16	-	TGCAGCAACTCGCCTTGTGGGCAGGCCGTGCCCT[C/ T]GGGTTTTGCAATTCCTTGTATTGATCTAG
TP3303	5H	41.16	bowman_contig_887289	TGCAGAATTATTTTTTCCATAACAGAAGACACGCGG GAGACTTGAGAACCAAGGCACG[G/T]GACCT

Marker_ID	Chr.	cM	Barley_Contig	Sequence
TP471	5H	41.16	-	TGCAGAAAGAAATAGCAGCCTGAAC[A/G]CTCCAAAAGTCCAACCATGTGACCTCCGAGATCGGAAG
TP926	5H	41.16	morex_contig_60397	TGCAGAAATTTTGTCCAAAGAT[G/T]CTGCAACAGTTATGAACCTGGTAGCAGGGTGGTGCTGCAAC
TP2413	5H	41.16	barke_contig_63654	TGCAGAAG[C/T]TTCTGCTGCTGCTGACAATGATAAAAGAACAGTAACAGTTGATGAACCAGTGTTG
TP3335	5H	41.16	morex_contig_61229	TGCAGAATTCTGATGCTGATAGAATGATAAGACATTTATAAGTTACTAAT[G/T]TGGAGAACAACCT
TP3396	5H	41.16	barke_contig_411017	TGCAGAATTTGCTATACATATTGATTAATACTCACAGTTGTAC[C/G]ATGGAACAAGCTCGTATAAA
TP3842	5H	41.16	barke_contig_267982	TGCAG[A/G]CATACTGATGTCAACAAATGGGAAGTATGTATATAACAATATGTCAACTGAGATTAGC
TP4088	5H	41.16	-	TGCAGAC[C/T]ATAACAATGACTTAAGCTAAATCGCTCTTCCATCAATGGCAATTTGCAGGAGTTAC
TP5468	5H	41.16	morex_contig_66584	TGCAGACTAGCAATGGTAAGGCGTGCTCGGCTCATCTGATGCC[A/G]CAAATCTGACGGCTCGCGAC
TP6316	5H	41.16	morex_contig_38131	TGCAGAGATTGTTCTTGGATGCGCCGTTTTGC[G/A]ACCGAGATCGGAAGAGCACACGTCTGAACTC
TP6862	5H	41.16	morex_contig_38131	TGCAGAGCGCCAC[A/G]AGCGCCGTGTTGACCGTGACGGCGTCGAACGGGACGCCGCGCTTGCACAT
TP7498	5H	41.16	morex_contig_51506	TGCAGAG[G/T]CAGATCAGCGTGCTGCACATGTCCCAGGCTGCTGCCGCCGCCAGAGCATCCGTCGG
TP8196	5H	41.16	bowman_contig_2010139	TGCAGAGTGGTGTCCTCTTCCCAGCCAAAGTCAGCTGAGTC[A/G]AAGCAGCACTTGCGAATCACAA
TP8385	5H	41.16	bowman_contig_879642	TGCAGATAAAACATTACCCACCTGTACTGTTTTAGATACATAAATCTACACCTTGCAACTGT[A/C]G
TP9562	5H	41.16	-	TGCAGATCGATACTGCAAATTCAAACATACTAGTT[C/G]GCGCTAGAAACATATCACCGAGATCGGA
TP10983	5H	41.16	morex_contig_38410	TGCAGATGTCGATTTGCAGTTTCTGATC[A/G]TCTATAACTATGAGTGTGCAGATACAATCAGTACT
TP12162	5H	41.16	bowman_contig_895903	TGCAGCAACACGAT[C/T]CATCGAACCATATGTGTATATCCAATTAGAACAAAACTTTAGTTTATCC
TP12713	5H	41.16	barke_contig_606900	TGCAGCAAGCAAAATAACTTCAA[C/G]TAGACTTGTAAATAAAGCTATGCTCCCCGAGATCGGAAGA
TP13208	5H	41.16	-	TGCAGCAATGGCGCCGTGCCAGTGGTCGTGGAGC[G/T]TACCGAGATCGGAAGAGCACACGTCTGAA
TP13639	5H	41.16	-	TGCAGCACATCAAAATCATCACGCAGAGACAACACCTCCTGGAGATCCGCGTCATCA[A/G]TCGCCT
TP14124	5H	41.16	morex_contig_369170	TGCAGCACCGCCCACACCTCATTTAACAGATGG[A/G]AGGCTGACGATACCCGTGCCGAGATCGGAA
TP14847	5H	41.16	bowman_contig_88897	TGCAGCACGGTCAACTATATATCAACAAGACTTACACCGC[C/T]ATCACAGCCGAAGAGTTAAGAGC
TP15318	5H	41.16	-	TGCAG[C/T]AGAACGAGGTCGTTGAAAGGCGCAATC

Marker_ID	Chr.	cM	Barley_Contig	Sequence
				GAACCGTTGTTGACATGGCAAGGAGTTTACT
TP15610	5H	41.16	-	TGCAGCAGAGCACTGTAGCCATGTCCTGCCC[A/G]AGGCTCCCGAGATCGGAAGAGCACACGTCTGA
TP18295	5H	41.16	bowman_contig_1317130	TGCAGCAGGTCCCCCTCCGTCACGTGGAGGGGAGGAAGTCGC[C/G]CTGGATCCGTCCCGCCGAGAT
TP19507	5H	41.16	barke_contig_57234	TGCAGCATCTTCACAAGCGATACCAGTT[C/T]TGCTGGGGATACCGAGATCGGAAGAGCACACGTCT
TP20451	5H	41.16	morex_contig_1797732	TGCAGCCAAGTTGAGGACA[C/T]GCGTGCGGAATATCTCGATGTTTTCATGATGAGAACGCCGACCG
TP21189	5H	41.16	bowman_contig_843599	TGCAGCCAGAGCTCCACATACA[A/G]CCTCATATGGCGTTTGGACGCGGACGAGGGCTCACCGAGAT
TP21573	5H	41.16	-	TGCAGCCAGTGGCAGCCCCGTCGACCGTGGTGGGGGAGGCTGGAGTGGACGGGGA[C/T]GCCGAGAT
TP22146	5H	41.16	barke_contig_314565	TGCAGCCCATGCACACTAC[A/G]CATGCATAAGAAGCAACATCGCGAGGCTAACCTGCCGAGATCGG
TP22195	5H	41.16	bowman_contig_131167	TGCAGCCCCA[A/T]GGGTGGCCCTGGACCCCTGGGGAGGTCGCAGCTGACCACAGCTTCGGCCATGG
TP22362	5H	41.16	-	TGCAGCCCCGACGCGGGGCAACTCCTACGCGTAGGGCGACGATGACGGGCCGA[C/T]GAAACTCCGA
TP22571	5H	41.16	morex_contig_60397	TGCAGCCCGAACTGCGGTAACATGAGAGCATGGTTAACAGCAC[A/G]TCCGAGATCGGAAGAGCACA
TP24291	5H	41.16	-	TGCAGCCGTCTCCTCCCCTCCTCCT[A/G]GTCTCGTCCTCTCCCTTCTGGATCAAAAGGGAGCTCTG
TP24655	5H	41.16	-	TGCAGCCTCCACCCT[A/G]ACAAAGGGGCAGACGATCAAGCTCGAGGCGCGGAAGAAGAAGGGGAAG
TP24715	5H	41.16	bowman_contig_129823	TGCAGCCTC[C/T]GCCATGACAAAGGGGCAGACTAGCAAGCTAGAGGCGGGGAAGAAGAAGGGGAAC
TP25403	5H	41.16	morex_contig_1569564	TGCAGCCTTCTGATGTGAAGTTATTTCTCTTTT[C/G]TGCTTTGACATAGTACTCTTGGTTATACTT
TP25613	5H	41.16	bowman_contig_67252	TGCAGCGAAGAGAGATCGAATGACGCCGAAAGTTCTTCCTCGAGCGACATTGACG[A/G]GGTGGCAG
TP29107	5H	41.16	bowman_contig_91228	TGCAGCGCTAC[A/G]ACTTTGGCGTTCGCTTCCGAGCTTGGATGGCCATCCTGCTATCTTCAGCAAG
TP29770	5H	41.16	bowman_contig_201679	TGCAGCGGATTCCCCACTGCCGTCTCCAATAGAAGCAGCCACTGACCATAAC[A/C]TATTGCTAGTA
TP30150	5H	41.16	bowman_contig_79643	TGCAGCGG[C/T]GACGAGCTCCTCAATGCCGTGGAGCCAATCGTTGTGGCGGTCAGGGGCGGGCCGA
TP31701	5H	41.16	bowman_contig_11695	TGCAGCGTCGAAGACGTGCGACGAAATGATAGGGAAGCG[C/T]TTGGCATTGTAGGTGTTGGTCCAG
TP33057	5H	41.16	barke_contig_199004	TGCAGCTAGCTAGGGCACACATGCACGCACG[C/T]ACGTACGTACACGTGGTGGAAGGAACTGTGGA
TP33779	5H	41.16	barke_contig_1854775	TGCAGCTCCATAGT[C/G]GGCTGCCTCTCTCTGCGCACCCGAGATCGGAAGAGCACACGTCTGAACT

Marker_ID	Chr.	cM	Barley_Contig	Sequence
TP34297	5H	41.16	-	TGCAGCTCGAACATGGAGTACAGGGCATCCTCGACCTCTCGCGCAGCCAT[A/G]GACGGCCTCGTCC
TP35051	5H	41.16	morex_contig_127915	TGCAGCTCTCCTCGTAATCGCAGGCCCACGTGCAGAGGTAGTAGAC[A/G]TAGTTGGTAGCAGGAGA
TP35464	5H	41.16	bowman_contig_863279	TGCAGCTGAGGTCATCGTCCGCCCGTCCGCCAGCACGCGTCTCTT[C/T]CAGTGCAGTCCAGTTCGC
TP35851	5H	41.16	-	TGCAGCTGCCGCACAAGCCAAGCCGCTAC[C/T]GCCGTGCACCTCATCGCCGAGATCGGAAGAGCAC
TP36023	5H	41.16	morex_contig_2659267	TGCAGCTGCGGAGG[G/T]CGGCACGAGCGCCCGTGTTGGCGGAGGGGTGTGGGCCGCGGTCGCCGC
TP36182	5H	41.16	-	TGCAGCTGCTCCTCGGGCTAGAGGCAGGAGCCTTCGGGGGCTCG[A/G]GGCTACGAGCATGTCGACG
TP37226	5H	41.16	morex_contig_1564422	TGCAGCTGTGTGG[C/T]TGCACCTGCTGGCCGCAGCGGTATATATGTACACACGATCGACACAAGAA
TP37239	5H	41.16	bowman_contig_2050429	TGCAGCTGTTCAATCTTCTTCCCATGTGTTCGTTCTGCTGACTGTTCGGTCTATTCTAT[A/T]AGGG
TP38987	5H	41.16	bowman_contig_111327	TGCAGGAACATCTGTCTGGGAT[C/T]CCACTGCGGTCCCTGCCCCTGCGGCCGCCCAAATGAGCTCG
TP40327	5H	41.16	barke_contig_64855	TGCAGGACC[A/G]ATGATGGACATTGTGCTAGAGGAGGACAGTCACCAGACAGGATTTCTGGCCTCC
TP41557	5H	41.16	bowman_contig_1984088	TGCAGGAGCAG[C/G]AGCACAAGCTGGCGGCAGCATTGGTGGACGTGGAGCAATGGCGCCGAGATCG
TP42649	5H	41.16	-	TGCAGGAGTTGGTCCCGCCAGGCCTGCAAAAT[G/T]TAGATGTTAGTTCAAAATGTACTGCCAACAT
TP43711	5H	41.16	bowman_contig_127247	TGCAGGCAACC[A/G]TGCGCGCAAACACTGAGACAAACGAGGCCTATTCTTGTTGGCCAATTCGGCA
TP43953	5H	41.16	barke_contig_366989	TGCAGGCACCGTTTTCCATAACCGACGCTGAAGCGCCGAATAGGAGGTCCCCTTTTTTTA[C/T]AGG
TP45444	5H	41.16	morex_contig_159411	TGCAGGCCCTCTGCAAAGCAATGCGCGAGGCGCTGAGTCCCATCGCCATC[A/G]GGGCGAGCATGCA
TP46989	5H	41.16	bowman_contig_899379	TGCAGGCGCCAGAGGCA[A/T]GCGGAGCTCCTAGTCAGCGCTACAGGCACTGGTTAACGATCCGAGA
TP48667	5H	41.16	bowman_contig_112624	TGCAGGCTCGGAGCCCGAGCACCATGGCGAGGCGGCAGCATCCCCTGGGG[C/T]GGAGGCTGTTGAT
TP49145	5H	41.16	bowman_contig_176481	TGCAGGCTGTCTTTTTTTCACAAACAAATCTCCAGTACATGCACGTCCA[A/G]CTCGCAACTTCCCG
TP49239	5H	41.16	morex_contig_136756	TGCAGGCTTGAATCCGTACGGTT[A/T]AAAAAGGTAATAAAAAAAAGTCCTACCTTGCCCAACTAAA
TP49483	5H	41.16	-	TGCAGGGAAGGAACGT[C/T]CGCTACTCAATTCTGAACGATTAACTTTCCATAACAGTTATAGTACT
TP49502	5H	41.16	bowman_contig_867044	TGCAGGGAAGTGTGGCCATGCATGCCCGTGCACTGTTTTGGTCCTAGCCCAA[C/G]AATGTTCCTAC
TP49647	5H	41.16	morex_contig_135879	TGCAGGGACGATAAGGCGACCCCTTACTTGGTCCCT

Marker_ID	Chr.	cM	Barley_Contig	Sequence
				TTTAGGT[C/G]GACTCGTCTTCGTGGGACGC
TP50271	5H	41.16	morex_contig_42293	TGCAGGGCA[C/T]AACCTCTTATTTCACCTATCGAGG TTGACCACCAGAGTCTCTCCGAGATCGGAA
TP53229	5H	41.16	bowman_contig_854679	TGCAGGGTTCTGTGAATAGGAAAGTGTCTATGACAC ATATGAATCATG[C/G]GAGTAGTCGCTCCCA
TP53401	5H	41.16	morex_contig_6554	TGCAGGTAACCAATACTTGCAATTCCTCCCCATGTT[C/T]GAGAAATCAGCCGCACCAAATGTGCGC
TP54638	5H	41.16	barke_contig_364158	TGCAGGTCCTTCAAGGATCTCACCCGAGCTATG[A/T]TCCGAGATCGGAAGAGCACACGTCTGAACT
TP57913	5H	41.16	morex_contig_1610622	TGCAGTAAATCGAGTATGTCAATTCCTGCAT[C/T]CG CCAACCACTGTCCACTTCTCAGCTGCAAAA
TP58055	5H	41.16	barke_contig_1854775	TGCAGTAATAAAATCGCACAGGAACGA[A/G]GCCA ACCCGAGATCGGAAGAGCACACGTCTGAACTC
TP58963	5H	41.16	bowman_contig_2074103	TGCAGTAGGGCCGAACATGTCAGCGCGTACTGCTG[A/C]TGCGGTAGACTCTGATCATTCGTCTTGT
TP59142	5H	41.16	bowman_contig_99214	TGCAGTATATATGCAGTGCAAGA[C/G]CTGTTAGAT AAAGCGAGATCAAAGGATCACAAGTTATTTA
TP59387	5H	41.16	morex_contig_59753	TGCAGTATTGCAACCGTATGAA[C/T]ACCAGACATC TTTACAGAAGTAGATTAACTCTACGCAAGAT
TP59811	5H	41.16	barke_contig_458195	TGCAGTCAGTGGGGACAAGATGCTC[C/T]CCATCCC CAGCACCCAAGAGCGGGGTGAGCATCGAAAA
TP59926	5H	41.16	morex_contig_60995	TGCAGTCCAACACTCCA[A/G]ACAACCCAAACTAGG CAGTGCAGCTCCGAGATCGGAAGAGCACACG
TP60136	5H	41.16	morex_contig_61229	TGCAGTCCCCTGGTCGAAGTGTTGGAAACG[A/G]AA GTAAGAACAAGGCAGTAGGATTTGGAACAAA
TP61245	5H	41.16	barke_contig_421793	TGCAGTCTGGTGACACCACTGCACTACACAC[C/G]G TAGGTACAGTAGCCATGCATCTGTGAATTGA
TP61830	5H	41.16	bowman_contig_853407	TGCAGTGCAAAGAACAACCATCACGGGAACGACCA ACAATGACAGATGTT[C/G]TGGCTGAGCTCAA
TP63097	5H	41.16	barke_contig_53074	TGCAGTGGGGCATC[A/C]AGCCTATCTTTCATCTTCT TGTCAACTATAGCTAACACATCTTTGTAGC
TP65374	5H	41.16	-	TGCAGTTGGTGGAGCACGGAATGATTGCGCATAAG TCGTGGAGGTT[G/T]CCGAGATCGGAAGAGCA
TP65412	5H	41.16	bowman_contig_152375	TGCAGTTGTAGCTAACTGACTAGGATTACCAGATCG [C/G]AGATCTTCTCATGTGCACGTCGGCGAC
TP65588	5H	41.16	bowman_contig_179016	TGCAGTTT[A/G]CTTTTGCAATAATTTTCCAGAGGCT GGAAAGACACTAGGCCGAGATCGGAAGAGC
TP65734	5H	41.16	-	TGCAGTTTCGCTGTTGTTTGTTTGTTTCGTATCCCGT CGCT[C/G]GTCTCTCGCCTTCTTCTCCTAC
TP66054	5H	41.16	morex_contig_1576126	TGCAGTTTTG[A/T]ATTCTTGCAGAAACAAAAATCA ATTGAAATTGTAGAAAATTACATGTACGGGA
TP66165	5H	41.16	-	TGCAGTTTTTTTTACAAC[A/T]GACGCTTTGTTGCAT CTTTTTTTATTATTTTTGCAACAATCATTT

Marker_ID	Chr.	cM	Barley_Contig	Sequence
TP33838	5H	42.46	bowman_contig_270988	TGCAGCTCCCCAT[A/C]TCCCATGGCCCGCAGTGCA CCTCACCCCGAGATCGGAAGAGCACACGTCT
TP3584	5H	42.46	-	TGCAGACAATTTGAACAATAATACGCG[A/T]ATTCC TTTTTGTGTTATCCCAAATCCTATGATTTGT
TP5761	5H	42.46	-	TGCAGAGAAAC[C/T]TGCCGAGCGAGCCAAGGCAA AGCATCCAGAACCATGTCCTGGTCCAAGGCGG
TP7729	5H	42.46	morex_contig_1558640	TGCAGAGGGCGCCGCCTGTCCTCCGATGCCCATGAC GCCGCAAGCATTT[C/G]CTCCATCGCGCCAT
TP7958	5H	42.46	morex_contig_49466	TGCAGAGTACCTCTTGTC[C/G]AGCTCTGCAATTTTT ACTTGCTGCTCCTCCAACTCCGAGATCGGA
TP10885	5H	42.46	-	TGCAGATGGTCTTTTATCTCTTTCAAAGTTTATTAAA CTTTGTACTAATT[A/T]ATCCCGAGATCGG
TP12380	5H	42.46	barke_contig_361544	TGCAGCAACGAGACCAGCCCCGATGTCCTCCCTGAT GTC[A/G]CCGAGATCGGAAGAGCACACGTCT
TP13427	5H	42.46	-	TGCAGCACACACACCGTGCATCC[A/G]CTCCATTCC GTGCCGAGATCGGAAGAGCACACGTCTGAAC
TP14225	5H	42.46	-	TGCAGCACCTCCATCACCGCCATCACCTCTCCCATT TT[C/T]CCCTTCTCGCAGTCGCCCGCCGCCA
TP15770	5H	42.46	-	TGCAGCAGATCCTGCTAAGAATGCCCTCTACACGGA GTCGACGGATGGCCACGACACCGACAC[A/G]
TP19018	5H	42.46	-	TGCAGCAT[A/C]TTCACCACAGCCCTCATTGGTGGG CGTTTGCTCGGGGCGGCGTTGACGCACAGCA
TP19746	5H	42.46	-	TGCAGCATGCTGGCCGCCAGAGGCATCACGACCGC AGCCACAACCACCTCGCCGCACGAC[C/T]AAG
TP19989	5H	42.46	morex_contig_47079	TGCAGCATGTTGACG[A/G]TGAAGATCTAGCAGATG TGTGTGCCGTGGGGCCGAGATCGGAAGAGCA
TP21798	5H	42.46	barke_contig_138539	TGCAGCCATGGCCAACGGTGGAGC[C/G]GCGTTACA ACACCCGATGTTAGGACGTCGGTGCTGAGCT
TP24659	5H	42.46	-	TGCAGCCTCCACTATGACGAAGGGGCAGACGAGCA AGCTCGAGGCG[G/T]AGAAGAAGAAGAAGACG
TP24841	5H	42.46	-	TGCAGCCTCGTAGGTAGGGCGCTTTCGCCTTGACCT AGCCCTCTATCCTCATACGTT[C/T]TCCTAA
TP25800	5H	42.46	bowman_contig_903304	TGCAGCGACCA[C/T]CAGGGATTCCAACTATGACCG CATTCTGAAAAATACATATGCATAAGCGGTG
TP26624	5H	42.46	barke_contig_381272	TGCAGCGATGCTGGCTCAGATCCGCAAGATGAGGC ATGCACT[C/T]GTGAGTCTCTTTTGTAACTCC
TP27134	5H	42.46	morex_contig_274333	TGCAGCGCATCGCCTGGGTCGGCGACGACCACGAG CGCTTCCTCTCCAA[A/G]TTCAAGGACCGCGT
TP29274	5H	42.46	barke_contig_501348	TGCAGCGCTGCATAGGAGCTCCCTCAATCCGCCGCA AAGTGCGGTAGATAG[C/G]AGATCCAAACAA
TP30803	5H	42.46	morex_contig_128321	TGCAGCGGGCAGTTGTCGCACTTCATGAACGGCAAT G[C/G]TGAGCCGAGATCGGAAGAGCACACGT
TP32593	5H	42.46	bowman_contig_925657	TGCAGCTAAGTGCATCTTCACCCGCGTCCCCAACAG

Marker_ID	Chr.	cM	Barley_Contig	Sequence
				GCTTCCAA[C/T]GCCCTTTTTACCGTTGACG
TP32709	5H	42.46	bowman_contig_1984151	TGCAG[C/G]TACCATTAACGCCCAAAGTGACTTTCT GCGGGTGGCTGCGCTTCACACGCTACGCGTC
TP35245	5H	42.46	-	TGCAGCTCTTGTTGGAAAAAGAAAAGGCATACT[G/T]TTTGTATGTGGCCGAGATCGGAAGAGCACA
TP36612	5H	42.46	morex_contig_85755	TGCAGCTGGCATGAGTCAAAGGCAGGGACACGACA [G/T]CTCGGGCGAGCGCATGCAAAACGTGCGC
TP36706	5H	42.46	bowman_contig_64133	TGCAGCTGGCGCGCGCTCCCGCG[A/G]GACGAGGAT GTCGTGGTCGATTTCGATCCCTGGTGGTTCG
TP38808	5H	42.46	bowman_contig_142662	TGCAGGAAATAGCATAA[C/T]TGATGAGACAAACGT ATATAACAGATCATCCATTAAAGCGGGTCAT
TP39328	5H	42.46	-	TGCAGGAAGAGCATGGCCAGCGGCGGTTCGTCC[C/ G]CGCACAACTGCGAGGCCTCCGAGATCGGAA
TP39402	5H	42.46	morex_contig_274423	TGCAGGAAGCATCGTCGCGGTGTGTTCAGAGAGAC GATGTAC[C/G]TATATCGCCGTGTGCGAGGCT
TP39536	5H	42.46	-	TGCAGGAAGGAGCTCGTC[C/T]GTAACCCGAAGCTG ACGAGGAAGTGGCTGTCGAAGACAACGCGCT
TP42117	5H	42.46	-	TGCAGGAGGCAGGCGGCGCGGTTGCTGTGGAAGAC GGCGCGGTCGGGATGGCC[A/T]CGCGGGGCAA
TP48976	5H	42.46	barke_contig_394590	TGCAGGCTGCTCGTCGAGCTCAGCGGCGGGGAAGG GTACTGCATTCGCAACACGTAGGA[C/T]GAGA
TP49350	5H	42.46	-	TGCAGGGAAAACGGAAAGGCCAAGGGGCGCGTCCA CTTA[C/T]CCAAGTCAGCAGCGGCGGGGCCGA
TP53525	5H	42.46	bowman_contig_877666	TGCAGGT[A/G]CAGGGGTGGGGAGAGAAGACGTGG CTTTGGCGAACCCAAGTGTGAGCGTCACCACG
TP53911	5H	42.46	bowman_contig_867723	TGCAGGTAGGTTCCCTGAATACATTTGTCTGTAGAT GTGGTG[C/T]CCAATTTGGTTAGCTATCTCA
TP55299	5H	42.46	-	TGCAGGTCTCTGCCTTCGCA[G/T]ATGTGGAAGATGT AGCGGAGTCGGAGGGCTCCGAGCCGCCCGA
TP57834	5H	42.46	-	TGCAGGTTTTGTACTGCTACAACATTATCTTA[T/C]T GCAATATACTACTTGCTCCTATGCGTTTTT
TP57869	5H	42.46	barke_contig_63596	TGCAGTAAAATGCACTAGCCGTCATTGAACTTGTCC CGATAGCTTATTTTGATCATCGTATTT[A/T]
TP58107	5H	42.46	morex_contig_67290	TGCAGTAATT[A/G]GGTGACGCGCCGCCACCATCCG CCGCCGTCCTCCACGCCTCTCTTCCCCGCCG
TP59631	5H	42.46	-	TGCAGTCACCTCGCTAATCCTGGGTTGGATGCGACA C[C/G]CTCCCAGCTGGGTCCAGTTTGTTTCT
TP65152	5H	42.46	barke_contig_1783299	TGCAGTTGCTCTGGTTGACTGACACCGCTTTTAGTC ACCAGCTTGCTTCCACTACATAGCCTC[C/T]
TP65189	5H	42.46	barke_contig_1823530	TGCAGTTGCTTAATTAGAGCATCTCCAACAGCCACG CTAAACTAGCGCCGCG[A/C]CGCAAATCCGA
TP33684	5H	42.46	morex_contig_131234	TGCAGCTCCACCA[C/T]CCCTTCATCCTCTATCTCAA CCGAGATCGGAAGAGCACACGTCTGAACTC

Marker_ID	Chr.	cM	Barley_Contig	Sequence
TP220	5H	43.76	morex_contig_208770	TGCAGAAAATATTTGCCATTTATTTGGTCTCTTTTGT TTTTGCAATATGG[C/T]TGTTTTTGCGAAA
TP3891	5H	43.76	-	TGCAGACATGACGGATGTTTG[A/G]GACTGAAAGGC TTGGTTGTTGTTGTTGTTGTTGTGCAGACAT
TP6238	5H	43.76	-	TGCAGAGATCGATTCGT[A/T]TTCGAGGCGTTCGTAC GTACGAACGTGCGTACGAGTAATACATGCG
TP6819	5H	43.76	bowman_contig_16293	TGCAGAGCGACGTGTACGCGTTCGGCGTCGTCCTCC TGGAGCTGCT[C/G]ACCGAGATCGGAAGAGC
TP7886	5H	43.76	barke_contig_1785439	TGCAGAGGTGTCGTCCCTCAAGGAGTCGCTCAAGG CTACAAC[A/G]TTGGAGGAGAAATCGTCCGAG
TP8496	5H	43.76	bowman_contig_1983012	TGCAGATAATGGGCTCAG[C/T]AATCTGCACAGGAA TCATGCAGAATAGTTAGTTAACTCAACCCTT
TP9960	5H	43.76	-	TGCAGATCTCGCGTGCTGCGGCACGG[A/G]AATGGG TTCAGGGGGTCCTGGCGACTCTCCTGGTGGT
TP17695	5H	43.76	-	TGCAGCAGGATCTCGTTCCACGTGTCGAT[A/G]GGG CCAGGGAGGCACCAGTGCACGCAGTCGTTCG
TP19426	5H	43.76	bowman_contig_11204	TGCAGCATCTCCTCATTTGCTTCAAAATTTTGAAGA GTTTGCAGACTAGTGGAATGC[C/T]ATTTGC
TP20271	5H	43.76	barke_contig_2785616	TGCAGCCAACAACACAGGCAATAGAGGCGGTGACT TGTA[A/G]CCCACCGAGATCGGAAGAGCACAC
TP20478	5H	43.76	-	TGCAGCCAATCGCCGTATTCTTGGCGTATTTTTCAG[C/T]GAGGCGTATTTTTCCGTAGTTGTCTCT
TP20745	5H	43.76	-	TGCAGCCACCCGAAGGCCGTGCCGCGGTGGTCGTC GTCGGTGGAGTCGGCCACCAGTGACTG[G/T]A
TP21813	5H	43.76	bowman_contig_861561	TGCAGC[C/T]ATGGCGGTTGCACAGAGTGCACCCTC TGCTGCTTAGGCCACGCCGAGATCGGAAGAG
TP22087	5H	43.76	-	TGCAGCCCAGG[A/G]CGTTCAGAACGCCTGACTGTC GCGAAGTGATGACTACTCTCTGGTTTAGTGC
TP29695	5H	43.76	morex_contig_1568669	TGCAGCGGAGGTCCAATGACCCGAGGAGGAAGAA[A/G]GCGGCAAGCGGGCGCTACGGTGCCCTCCA
TP31560	5H	43.76	bowman_contig_16293	TGCAGCGTCAGCTTCCCCGTCTGCACGCGAGATGGA GTCCTCGTTAGTGCAACCAGTACCAG[G/T]A
TP36985	5H	43.76	bowman_contig_146457	TGCAGCTGGTTACAAACTTTATATGTCTTGGGCTGG TTCACTGGAAACTC[C/G]TGGAAATCTGATA
TP40891	5H	43.76	morex_contig_39358	TGCAGGACGGTGGCGGA[A/G]TGTGCGGCGAAGAA GAAGCTGGTGGAGCCGAGATCGGAAGAGCACA
TP43784	5H	43.76	bowman_contig_69889	TGCAGGCAAGGCATGCACGCGCCCATCCATGGCGG GACGTACG[A/T]ACGTGTTCGGTTAGCTCACG
TP44287	5H	43.76	barke_contig_586761	TGCAGGCAGG[A/G]AGCTAATCGAGCAAGTAATGA ATCATCACACAGGGGAGAAAAAGACGGATAGA
TP49266	5H	43.76	barke_contig_1810049	TGCAGGCTTGCGA[C/T]CATCCCATGAAGCACCAGT GATGCTCCGAGATCGGAAGAGCACACGTCTG
TP53415	5H	43.76	-	TGCAGGT[A/G]ACGAGGCTTGCGGATATGCGCGCGG

Marker_ID	Chr.	cM	Barley_Contig	Sequence
				ACGCTCACCCCGAGATCGGAAGAGCACACGT
TP58241	5H	43.76	bowman_contig_1990868	TGCAGTACAGGAAGGAGTATATGGCGG[C/T]GCAGA CCGTGAACGGAATCGCGATGGCCGTGTACAG
TP62023	5H	43.76	-	TGCAGTGCAGTGCACAGCACACCAAGCAAAATGAA GTGAGGATTCT[C/T]ATTGTGCTGGAGCAAAT
TP64213	5H	43.76	morex_contig_56912	TGCAGTTCCACCACAAGTCGGGCGACCT[G/T]TACG TCGCCGACGCCTACCTGGGGCTGATGAGGGT
TP7694	5H	45.05	morex_contig_190738	TGCAGAGGGAGATGGGCTGGCCCAGTTGATTTCGC CCCTGTGCGTTTGCCCAA[C/T]TATTTGACGC
TP14496	5H	45.05	-	TGCAGCACGAGCGG[C/T]CATGTACATGCATGCGAC AGTGCTAGGACTAGGAGCAAGTCGGAATTGA
TP38269	5H	45.05	morex_contig_47842	TGCAGCTTGTCTGGGTTCTTCCCGCTCATCACAAGC TCTCTATCTGCCTACACCAAGAT[A/T]ATAA
TP27649	5H	48.97	bowman_contig_1986783	TGCAGCGCCGACCGCACCTGATCTGT[C/T]AGATGA GCGGCCGAGATCGGAAGAGCACACGTCTGAA
TP57339	5H	48.97	morex_contig_140159	TGCAGGTTGAGTTGCTAGTGAAGCCTGGGCAGGAT CAGCTGGCTGGGCACTTGGTG[C/T]TGGTGCA
TP22561	5H	50.04	barke_contig_277976	TGCAGCCCGAAAGAAGTTCAGTCAGCATACAATGC GAGTTAGAAGAAGGCTTGAA[G/T]CCTTTTCT
TP25346	5H	54.11	bowman_contig_9828	TGCAGCCTTCATTCCAATATCGAACACGTCCCCCAA TGTCTCCTGCAACG[C/G]ACCACTCTGAGCA
TP14521	5H	54.11	morex_contig_47526	TGCAGCACGATCCCCTCCCACCATCCGCCGTGCATC CGCGC[A/G]TCGACGACGACACCGACGGCCA
TP53164	5H	55.47	barke_contig_364076	TGCAGGGTTAAATCTTTCGGAAAGTC[A/G]TGCCCG CGGTTATGTGGAGAACTTGGAAACTTTGTAT
TP63944	5H	55.47	-	TGCAGTTATACACGACCAAGGGAGGGGGTTTGATT[A/G]TGTTTTTGTTTTCAGATCGAGTGGTGTG
TP64610	5H	55.47	morex_contig_39146	TGCAGTTCGTGAAGG[A/G]AAAATCTGAGCCCTGGC ACGACTTTCACACTGTCTCTGGGCCGCGAGA
TP65403	5H	55.47	bowman_contig_66286	TGCAGTTGTACTCCCTCCCGCGGCAACGGCAG[T/C] GCAGGCGCGCGCCCGAGATCGGAAGAGCACA
TP61970	5H	59.47	-	TGCAGTGCAGATTGGTCGCAAAATTTCAGAAAACTC ATGCACATGCACATGC[A/T]ACCCATGCACG
TP63388	5H	59.47	-	TGCAGTGTCCGCGATT[A/T]AAACCTTGATGAAAAC TGTCACCGAGATCGGAAGAGCACACGTCTGA
TP22150	5H	61.98	bowman_contig_14155	TGCAG[C/T]CCATGCCCCAACCGATACAGAAGACAC GCGAGGGAATACGTACGGATTGATCGCTCGA
TP62302	5H	61.98	bowman_contig_1982336	TGCAGTGCGATAT[A/T]CTTTGCCAAATCCAATCCTC TAAACTTGTACAAAATTCATGAAATCCCAA
TP137	5H	63.27	morex_contig_137527	TGCAGAAAACTCCAGCAGCGGTAGGAACGGTACGC [A/G]GCACTGCAAGCTGAATTTCGTTTTTGAT
TP697	5H	63.27	morex_contig_1574269	TGCAGAAAT[A/T]CGACGCGGTGGTGGCGGACATGA CCATCACGGCTAAGCGATCAAACCACGTGGA

Marker_ID	Chr.	cM	Barley_Contig	Sequence
TP3803	5H	63.27	bowman_contig_69638	TGCAGACAGTATAAATAATCTCTCCGATAGCGTCAC GCAGCACCATACATCCCCCAG[C/G]AGCTCC
TP11237	5H	63.27	-	TGCAGATTCATAGCGAAATCGAGGGGAAGATTGAG C[A/G]GACTGCCATCCACCGAGATCGGAAGAG
TP18294	5H	63.27	bowman_contig_370012	TGCAGCAGGTCCCCCTCCGTCACCGTGGAGGGGAG GAAGTCGCCCTGGATCCAT[C/T]CGCCGAGAT
TP26529	5H	63.27	-	TGCAG[C/T]GATCACACATGATGACCGATAGAGGCT CGCCGATGAGCAGTTGTGCGAGGGTTGAGTC
TP29062	5H	63.27	barke_contig_1788313	TGCAGCGCGTGGTCCAACGACGGGGCCTTCTGCTAC GGCTG[C/T]CAGTCGTGCAAGGCCGAGATCG
TP36946	5H	63.27	barke_contig_373500	TGCAGCTGGTGCTATCTAGGCAAAACTACGATTCTT AACCTTGTT[A/T]TTCTGCTGAAATTAACAT
TP47311	5H	63.27	-	TGCAGGCGCTAGTTTCCCTAGCGGG[C/T]GCCCACG CACCATCGCCCCGTGGGCCGAGATCGGAAGA
TP55128	5H	63.27	morex_contig_132683	TGCAGGTCGGTGTCAGTCAG[C/G]CAAGGAGATCTC CAGGGACGAAGAACGGAAGAAGCCATCGCTG
TP59005	5H	63.27	-	TGCAGTAGTACCTGTACATGTACACGTCTTTGTCGG G[A/G]CTGAGCTCCCTCCGAGATCGGAAGAG
TP60017	5H	63.27	morex_contig_1574269	TGCAGTCCAGCTACAC[C/G]GCCAGCCTCACCTCCTT GCTCACGGTGCCGCGGATCGGGCCGAGATC
TP13622	5H	64.57	barke_contig_59565	TGCAGCACAGTTTTTTGAACAT[A/G]GCCACTATGA AACTAGTCTTGGTGTCACGAAAACTCTACAG
TP17578	5H	64.57	bowman_contig_65258	TGCAGCAGGACGAGCAGGAGGCAGAGGAGGATCG GGAAGACGGTGATGTGGGAGGGC[A/G]ACGGGG
TP19385	5H	64.57	bowman_contig_846564	TGCAGCATCGTG[A/G]ACGTTAGTTAGCCCCGAAGA TAAGCCACACGCAGCAATGTCGCAGTCCGAA
TP33192	5H	64.57	bowman_contig_856820	TGCAGCTATATGTGTGCTGTTGGGGCTCTTGAA[A/G] CTTCTGTCTTGTCATTTTCACCTACGTGGT
TP39805	5H	64.57	morex_contig_42789	TGCAGGAATCATCACAAAAATTAACGAACCGAGAC GCTGAATA[C/G]CTGGCCTGAACTGGACTGCG
TP49859	5H	64.57	morex_contig_1559383	TGCAGGGAGCTAATTTCATGAGAGACACATTGATGT [G/T]GTTGGTTCTAGAAATTACTATTCTTCA
TP61923	5H	64.57	-	TGCAGTGCACGTCGTGCCACGGCTC[C/T]CTCGGTCC GCCGTACCGCAGCGAGGCGTGCCTGAAGTT
TP63873	5H	64.57	bowman_contig_86300	TGCAGTTAGAACTTAGAACCACTATGACTCCTGCTA T[C/G]TAATGATTGATTAGAACTTGCAGAAG
TP21428	5H	69.69	-	TGCAGCCAGG[C/T]AAGCTGCGCCATGCATGCACGT AGAGCGACGGGCAGCGGCTGGCCCAGGCCGA
cytochrome P450b	5H	72.5		GTGCTGGCCAACCTGCTGTATCACT[T/A]TGACTGGG CGATTCCGTGGGGAA
TP1327	5H	75.18	morex_contig_46046	TGCAGAACCTCTTCTTGTCTATCTCCTTCCTTGTTGC TCTATACTCCATAAGCTAGGCG[C/T]GTTA
TP63617	5H	75.18	morex_contig_159734	TGCAGTGTTGCATAGGAGCTCCCGCTAGCGCTGGTT

Marker_ID	Chr.	cM	Barley_Contig	Sequence
				AGGAGTTCT[C/T]GATGTTAGTGAGGGAAAA
TP2564	5H	77.63	barke_contig_1854775	TGCAGAAGGCGGCGCGCCGACGCCATTG[A/G]TTTC TGCCGAGATCGGAAGAGCACACGTCTGAACT
TP10726	5H	77.63	bowman_contig_105040	TGCAGATGGCATCCAGCATGTTACCTGCGCAAAACC ACTAGGGTTTGATTAC[A/G]GACATTGCTCT
TP21381	5H	77.63	-	TGCAGCCAGCTCATCATCTATCTCCATGACATCCAC CCACT[C/G]GGAGGATGCGAGCCCGAAAAAA
TP43107	5H	77.63	morex_contig_158756	TGCAGGATG[A/G]AGTCAGGCATGGATAAACATAGC CGCAACCAGCTCTTCCCTTCGTCCGAGATCG
TP52198	5H	77.63	bowman_contig_844272	TGCAGGGGGCT[C/T]GCCATCATCAAGGACTTGGCA ACCACCGCGGACCAGCGGCGGGAGCTCTGCG
TP61877	5H	77.63	bowman_contig_125479	TGCAGTGCACAAATGGAGATCGATCCGCAGAAAAC AACGGAGCGG[C/T]GATGGCGTGATTGGCGAA
TP21896	5H	84.09	morex_contig_53162	TGCAGCCATTGGTCGTGCATTGCTTCCCCAGCCTGC CTCCTGAACCCCA[C/T]GGCTCGATCGCTAG
TP13479	5H	88.09	bowman_contig_71185	TGCAGCACACGAGACGCCACGGACGGAACGGATCA CCGAGCGAGGATGGAGCGGTCGATG[C/G]AGA
TP15815	5H	88.09	bowman_contig_10226	TGCAGCAGATGATGC[C/T]GCTTCAGGTGTCCCTGTC ATGGAAGAGCAGGTGTGTTACTCCCCTCCC
TP18359	5H	88.09	morex_contig_6830	TGCAGCAGGTGCACTGTGCGCG[C/T]ACGTACGTAC GTACGTGGCCAGCTGCATGCGGCGTACTTGG
TP21841	5H	88.09	morex_contig_36884	TGCAGCCATGTTTTCCGTTCGTCCGA[C/T]CCATTGC AGAACAGCATGTCAGCATTCCCCGAGATCG
TP32805	5H	88.09	morex_contig_41925	TGCAGCTACGTGACGGAGGGCGGCGACGTCGAGGT CGACAACGTCGACGAG[C/T]CCCTCATCCTCG
TP34406	5H	88.09	morex_contig_160351	TGCAGCTCGAGCCTCCTCTCCCCTACACTCGCCGT[C /G]AGCTTGCCGCGAGGGAGGCCATGAAGTT
TP37425	5H	88.09	bowman_contig_861425	TGCAGCTTCACTTTCCAAAAGCACATATTCACGCGC TCCT[C/T]CCCCTATCTCATCTGCAATCGAG
TP41320	5H	88.09	barke_contig_272400	TGCAGGAGAGGAGATTTGTAGCGACCCAAGCGTGT GACCTGCCACAAGCG[A/G]CGACAGCGCCATG
TP44835	5H	88.09	morex_contig_41925	TGCAGGC[C/T]ACCTCCGAGGACATGGCGGCGGACG GCCTCGTTGCCCGAGATCGGAAGAGCACACG
TP54120	5H	88.09	-	TGCAGGTCAAGCAGCTAGCTACCCACATCGGCAGG ACAAGTGGTCTGAACTGTCGAG[A/T]TGCGCG
TP60422	5H	88.09	barke_contig_396930	TGCAGTCCTTTCTTTAACTAG[C/T]ACTACTGTCCTA AAGGAATGGACTACGGAGTTCTGCATTCTT
TP61807	5H	88.09	-	TGCAGTGATTCCGCTGTAAACATGTCCTAAGTGGTT[A/T]ATATTCATGGTGCAAAATGAACACATT
TP62069	5H	88.09	-	TGCAGTGCATGCAGGATAGGG[A/G]TCGGAGATCCG CACGAGCCGAGATCGGAAGAGCACACGTCTG
stress1	5H	90.9		GCTCTGGTCACGGCGTCGGCGC[-/A]GCTGTCGCCGA CGTTCTACGACACG

Marker_ID	Chr.	cM	Barley_Contig	Sequence
TP31423	5H	92.08	bowman_contig_1983607	TGCAGCGTACTGGACGACTTTTTTGTTTTTTGAGG GAAGCGTACTGGA[C/G]TACTGGTGCTGCCG
TP27265	5H	94.60	-	TGCAGCGCCACACGCAGGTTGCAGGCAGGAGGTCG TGG[A/T]ACGTCCTGACATGTGTTTTTTATCT
TP60961	5H	94.60	bowman_contig_11370	TGCAGTCGTAGCTCGGCGGCGCGTGGAAGCACGGC TCCATGGACGTCGCCTG[C/G]ACGCAGGGCGG
TP767	5H	98.51	morex_contig_162013	TGCAGAAATGAACCCTCATGTCAGCCGTCAGCGAT ACTACCAA[A/T]TGCCAATACAGAGCATTTTT
TP57496	5H	98.51	morex_contig_162013	TGCAGGTTGGCAA[C/T]CCGTACCTCGATGACTACA TGAACACAAAGGGGACGTTCGAGTTCCTCTG
TP57637	5H	101.11	bowman_contig_67781	TGCAGGTTGTGGAAGCCGCTGAGAGCTTCTTGCAAG TGCACGACGGCGC[C/T]GCTGTAGTTGACGA
TP64512	5H	101.11	bowman_contig_67781	TGCAGTTCGGACGGCGCCTAGCATCACGGAGGCGG C[C/T]GTGGGGAGGATCGCGCAGGGGACCAAG
TP8909	5H	101.11	morex_contig_43849	TGCAGATCAA[A/G]CCATACAATGGAGAGGTAGAAC GCACCACTTCTCGTGAAGAGGACGAAGGAGA
TP3241	5H	102.41	barke_contig_559486	TGCAGAATGGTGGTATGGG[C/T]CGAGACTGCTTAA ATAGAGGGGAGATGAGGTGGTCGCGTGCATG
TP5372	5H	102.41	morex_contig_46457	TGCAGACGTGCGTGAGCGTACTCGT[C/T]AGTTTTTC TTCCTTTTTTCGGAAGATTGTATATCTTGC
TP38379	5H	102.41	bowman_contig_67912	TGCAGCTTTCTTTCCTGGGGAATGCTGCCGCCACTG TGGTAAAAGCAGTAAATTTCCTTCA[C/T]TT
TP57386	5H	102.41	bowman_contig_861930	TGCAGGTTGCATGGGTGCGCGAACGA[C/T]GGCGTG GACGACTAGCGAGGGCGGCAAACGACGACGA
TP59288	5H	102.41	bowman_contig_861930	TGCAGTATGCAGCTCTTAGCGAGCTCGACTGCCTTG CTACAATCACCATGCATATATGCTA[C/T]AA
TP61985	5H	102.41	morex_contig_111665	TGCAGTGCAGCCGTAGTAGAAA[A/G]CCTATTCATG ACGCATATGTCCAATGAACCTTTAGTCATAT
TP65251	5H	103.05	morex_contig_223455	TGCAGTTGGCAAGCTGTCC[A/T]CAAATCAACTGGC CAAGCCTGCGTGAGCATGTGAATACAAAACT
TP53055	5H	103.71	morex_contig_37227	TGCAGGGTGGGCCGAGGACCCGAAGTGAGATGCAC TGGAGCCCCAAGCTGTAGGTGTGAACCA[A/G]
TP57856	5H	103.71	-	TGCAGT[A/T]AAAATGTGCTGCGAAACTGAAGCAGC ACTTATCCGCGACTACGCGACGCCCGCGGAG
TP6068	5H	105.01	morex_contig_66092	TGCAGAGAGATTTGGTAGCTCTCTCACTGTGGCTTC GAC[C/T]GAAAGTCCCAAACCATGGCGATGC
TP11663	5H	105.01	bowman_contig_260417	TGCAGATTTGGTCACTTGAGAACGAAACGGAGCAA AGGTGGAGCCAGAAGTTCATAATTCAG[C/T]A
TP15528	5H	105.01	bowman_contig_845997	TGCAGCAGA[C/T]GGAGGCTCGAGGCAGAGGTTCGA GACCACTGAATTCATCATCCAATCCGAGATC
TP18999	5H	105.01	-	TGCAGCATATGAAGATCAGGATTATTATGGTCCTCG TAA[A/G]TTCTGGATCAGAGATATAGATGGG
TP28695	5H	105.01	bowman_contig_320547	TGCAGCGCGGAGACGAAGGCGGAGGAGGGCGGCG

Marker_ID	Chr.	cM	Barley_Contig	Sequence
				ACGG[C/T]GAGCCGAGGTAGGGCGAGGCGGGGC
TP31389	5H	105.01	morex_contig_52892	TGCAGCGTACAACAAGCTAGCTAGCAAAGAT[A/G]GCCATGGCTAGGAGCTCCAGCCTAGTGCTCGT
TP32607	5H	105.01	barke_contig_274849	TGCAGCTAATGCATAAACTACGGTGA[A/G]AATAAACCCTATACAATCTCTTGTGAAAATAAGTCTA
TP48663	5H	105.01	morex_contig_55755	TGCAGGCT[C/T]GCGGCGCCCCGCCGCCCCACGCCGAGCTCCTGAACGACGACCAGGCCAGGGTCGA
TP54493	5H	105.01	bowman_contig_71764	TGCAGGTCCCGAGGCCACCTAGCCATTGCAGGACACGCGGTGGCAAG[C/T]GGAGGGCGCGGCCACG
TP3247	5H	107.61	barke_contig_68684	TGCAGAATGTAA[A/G]CCATTGGATGAAAGAAGAATCCATGCTGCCCTTTCTGAATAAAGTGTAACA
TP33037	5H	107.61	morex_contig_357672	TGCAGCT[A/T]GCGCTCAAGTACAACGAGAAGAGGATGATCTGCCGCAAGTACGTGCATGATCAACC
TP8192	5H	110.21	bowman_contig_62579	TGCAGAGTGGTCACAGTTCAGTGTTCAATAATGACCACAGAATGTGAATACACAATA[A/G]TTTGAA
TP40039	5H	110.21	-	TGCAGGACA[A/G]CGGCAGCGCGAGGACTCGAAGCTGTCGGCGCAATCATTTAAGACGAAGACTTTG
TP26496	5H	110.21	barke_contig_371622	TGCAGCGATAAACATCACTCATTCACTCCCCTTCGCCTTGAGACACGCA[C/T]CATGTTTTTAGCCT
TP27060	5H	110.21	bowman_contig_69363	TGCAGCGCAGTGGAGTGCAACGCAAGTTCGCTCTCCTTGTCTCGACCCGTTGACACTGACAG[A/C]A
TP53979	5H	110.21	bowman_contig_853190	TGCAGGTATAGTAGGAACTGGAAATCTCCGTTGTTCTTCAGAAACATCATATAATGGAGAA[A/G]AC
TP25339	5H	110.21	morex_contig_37259	TGCAGCCTTCAACTTGCCAACTGTCTCAGATTCTATGAGTGTATGAACTAGAGATCTCCC[A/C]TCA
TP56728	5H	113.47	barke_contig_378550	TGCAGGTGTCGGTTAATATCTAGTATTTCGTTAGCTGACGGCTGTAGCGTCCTTAAATG[A/G]CCGA
TP174	5H	113.47	barke_contig_371809	TGCAGAAAAGCTCCCACTTATCTAACAGGAAAACATC[A/G]TCATGTTAGAATAAGGTGAAATCAAA
TP7055	5H	113.47	barke_contig_2806663	TGCAGAGCTAAGCTGGAGAGAACGCATCCACACAAACAAT[A/G]TGCAAAACTTGTTTTTAATCCA
TP22271	5H	116.71	-	TGCAGCCCCCAACCTCTCTCTTTCCTGTGTTCCTTTTTTTTACCCACTTCTTGC[C/T]CTTGCACCC
TP410	5H	116.71	barke_contig_56335	TGCAGAAACGTTGAATACCGTGAAGACCATGAAAGT[C/T]GACCGAATCCAGACGGATCCACTAGAA
TP63324	5H	116.71	morex_contig_138656	TGCAGTGTAGAGTTGTTGGCTCGCAATCTCGCATGGATTATATAATCTGAGCTGATTTTTA[C/T]CA
TP4105	5H	119.31	morex_contig_44590	TGCAGACCATCCG[A/C]CACATGCTCCAGATTCACGTCCTCGTCGACTACTACGGCGGCAAGAAGCT
TP59765	5H	119.31	morex_contig_49500	TGCAGTC[A/G]GCTGGAGCTGCTCTCCTTGAGCCAGTGTGGCTCTAGCTCCGACTCCGTGCTACGCA
TP66147	5H	120.64	bowman_contig_1149810	TGCAGTTTTTGTGTCAAAATTTCAAAAACA[A/G]CAACTTTCAATCTTGTTTTGGCCACAACTTTTT

Marker_ID	Chr.	cM	Barley_Contig	Sequence
TP23797	5H	120.64	morex_contig_1562535	TGCAGCCGCGACAGAG[C/G]ACCAACGACTCCCCATCGGCGCTCCATTGCAGTGCATGTGACGCTGC
TP61552	5H	123.21	barke_contig_373428	TGCAGTGACGGATCGAGCAAAGAACTGTGGCTCTAGTCCTT[A/G]AACCGCACAGCAGTACAAGAAC
TP21685	5H	123.21	bowman_contig_201213	TGCAGCCATCCA[C/T]GACGCTTGCCTAGAGAGGGTCAGTCCGTCGTCATGACTGTCTCCACCGCCG
TP44796	5H	123.21	bowman_contig_858672	TGCAGGCCACAGCGAGCGAGCGTACTGAAAATTGT[C/G]AGCCTTCCATACCGAGATCGGAAGAGCA
TP30502	5H	123.21	barke_contig_1854775	TGCAGCGGCTATGCCAT[C/T]TCGAGGTGTTTGAATGCCCGAGATCGGAAGAGCACACGTCTGAACT
TP481	5H	124.51	barke_contig_55586	TGCAGAAAGAAGCA[A/C]ACAAAAGCATCTGATTGCGGCAGGCACACAAAGAATTACGGAGAAGCAA
TP1203	5H	124.51	morex_contig_158491	TGCAGAACCAGCTACAGTATGGTACAGAGGTAATAATAAGCAAACCGAGGAGAA[C/G]CACCGAGAT
TP26666	5H	124.51	-	TGCAGCGATGTTTCTTTGGGGATGCTTTCGAGATGTTATGGGCGGATG[C/T]TGGACCCACGATAAT
TP31853	5H	124.51	morex_contig_1573938	TGCAGCGTCTCGG[A/C]CGACTTGTCGCTGCTGCGCGCGCCGAGATCGGAAGAGCACACGTCTGAAC
TP54576	5H	124.51	morex_contig_5956	TGCAGGTCCTCACGGGGCTGCTCGACCTAAACATCGTCGAGCAGGTGCAGGA[A/G]GCGATGAAGAT
TP55427	5H	124.51	barke_contig_275876	TGCAGGTGAAGAAAGACAACGCGTACCCTGAAATTGGGAGCATC[G/T]TCTGGTCAACAGGAAGGAT
TP57995	5H	124.51	barke_contig_54270	TGCAGT[A/G]AGAATTACCCCCACGATTAGGAGTCAGATAGTCCGAGATCGGAAGAGCACACGTCTG
TP1552	5H	125.81	barke_contig_1885936	TGCAGAACGTCGCCCGTCCATTAGGCCACACGTGT[A/C]ATCCATCCGAGATCGGAAGAGCACACGT
TP7983	5H	125.81	bowman_contig_872797	TGCAGAGTATCATCATGGACGGTGTCGTGTGT[T/C]GTCGAGGAAGAACTACGTCATGGCTGTTCAT
TP9226	5H	125.81	bowman_contig_860067	TGCAGATCCAGAAATTGAAG[A/G]AGTTTGTGGAAATTACAAAAACCTGCCACCGCTAAGTGAAATA
TP9378	5H	125.81	bowman_contig_66315	TGCAGATCCTCAAAATTCCAGCATGCCTTCAAGATGGTTGATGAGATGAGAAA[A/G]AGTGGAATTG
TP21869	5H	125.81	barke_contig_2791097	TGCAGCCATTCTGGCCTCCTGGAGCAAGGGAGAGCCTTCTTTGAGT[C/T]GATGGTGTCTGTCCATG
TP37653	5H	125.81	bowman_contig_855219	TGCAGCTTCGGCAACACCGACCA[A/G]CATTGCATGCCGCCGAGATCGGAAGAGCACACGTCTGAAC
TP46144	5H	125.81	-	TGCAGGCCGTCTTCAACAAGATCCTGAACGTGGCTGTGGCGGGCGCGCTG[C/T]CCAAGGACATGAT
TP57270	5H	125.81	bowman_contig_1981664	TGCAGGTTCTTTTATGC[C/T]TCGAGTGCTTGCATCTACCCTGAATTTAAGCAGCTGGAAACTGTAG
TP64421	5H	125.81	morex_contig_46296	TGCAGTTCGAGAGACATG[A/C]ACATGCTCATGCCAGTTGCTGCATATTACACCATACAGCAGCAAC
TP66111	5H	125.81	bowman_contig_864459	TGCAGTTTTTAGGTGAACCCGTGAAGGGGTCAGTGA

Marker_ID	Chr.	cM	Barley_Contig	Sequence
				GAAGAGGTTAAGCTCAAATCTTG[A/G]CCTG
TP38439	5H	125.81	-	TGCAGCTTTGTGAAAGGTCCACACACAAGCGCATC GTCACGCTTCATCGTAAAAT[A/C]TCTCTTAC
TP41829	5H	125.81	barke_contig_347920	TGCAGGAGCTATTGCAGGCATTTCCTGCGTTAGCTA TGACGACAAGTTC[C/T]GTAAATGCCACAGC
TP51646	5H	125.81	bowman_contig_127664	TGCAGGGGAAAAGTTTTTCGTACACCTGC[C/G]TAA AATGTTACGACGAACCAAAAATATGAAAAGC
TP55608	5H	125.81	morex_contig_7556	TGCAGGTGATCACT[C/G]CTACTACCACGGCTACTCC ATTTCTTTCTCCTCCGAGATCGGAAGAGCA
TP30920	5H	125.81	morex_contig_47850	TGCAGCGGGG[A/C]GAACTACTACTACTCGCCCTCG ACGTCGCCGAGATCGGAAGAGCACACGTCTG
TP10449	5H	125.81	barke_contig_54858	TGCAGATGCCTGCCCGTCTGGCAAAAGTTGGTGCCT TTTATATCCAACAGCGACAGAAT[C/G]GTTA
TP30857	5H	125.81	morex_contig_54269	TGCAGCGGGCGCGTGCCGACGGGTCGCTCGGGGCT CGGGGTGTTTCTGCCGCCGCC[G/T]CCGCGCA
TP38765	5H	125.81	barke_contig_370074	TGCAGGAAAGGCGACATATGCATGTAGGAG[C/T]AC GTACGTAGCAAATTAGGCGGCAGATCTGAAC
TP49142	5H	125.81	morex_contig_56378	TGCAGG[C/T]TGTCTCAGTCGAAGAGCAGGCGCCGT CGCAGGGACTTGGGTATCCTTTTTGTCAGGT
TP48739	5H	125.81	morex_contig_110432	TGCAGGCTCTGGCGCGACGCCGTCGACGAGC[G/T]C ACGACGGAGATGCACAGCCGCGCCAAGGCCC
TP32701	5H	125.81	barke_contig_389322	TGCAGCTACCAGCCAGGAACCAAGGCAGATTCCAG ACCAAAC[A/T]CACGTGCAACTCACAGCCAAC
TP19288	5H	126.67	bowman_contig_63697	TGCAGCATCGAGTCGGGTGAAGCATGCATTGCTGG ACTACGCCTTACCACAAGGAGCTCCGTC[A/G]
TP59559	5H	126.67	barke_contig_271560	TGCAGTCACAAAAGCATTCGCCATAAAC[A/G]AATT ATGTCAGTACTGTACAATATACCATTAAGAT
TP6718	5H	175.03	bowman_contig_9961	TGCAGAGCCGTGTGCTCCTTTAGGCCCTTGTGGACG GAC[C/G]TCTTCTTCTTCTTCAGCCAAAGAA
TP51583	5H	175.03	barke_contig_467928	TGCAGGGCTTCCT[G/T]GTCTTCAACGCCGTCGGCGG TGGAACCGAGATCGGAAGAGCACACGTCTG
TP10873	5H	178.79	morex_contig_51415	TGCAGATGGTCCACACCTCCTGAATTTTTAGGTTGC GAGCGCACA[C/T]GTCAGAGGGTCAATGTTA
TP34159	5H	178.79	morex_contig_160060	TGCAGCTCCTCCTTGTCCTCCTACTCTAGCGGCAGC CCGCAGAGCCCAAGGGGACGG[C/T]GTCGGA
TP39117	5H	178.79	morex_contig_52159	TGCAGGAACGGAAACAGAAAGGGGAAAACACTGA ACAAAATTATCTGAAATCTCAACAC[C/T]TGCA
TP3264	5H	180.09	-	TGCAGAATGTCTCAGGTTACATGTCGCCGT[A/G]TC GTCGTCTGCTCAAATGGTTTGATCTGCTGGG
TP6176	5H	180.09	barke_contig_320982	TGCAGAGAGTGGCCGCCCCATGGTAGAGAGATGTT AAGGCCATCTCCA[A/C]CGCACCATCCCATCC
TP7478	5H	180.09	bowman_contig_1982499	TGCAGAGGCAACTACTCGTA[C/T]AGGAAGGGTAAT AAGGCCAGGGATTCGGATGCCGCCAAGGAGG

Marker_ID	Chr.	cM	Barley_Contig	Sequence
TP19179	5H	180.09	morex_contig_231457	TGCAGCATCCAGTT[A/G]TGGTACTGCGAGAGGCCG TTGAGCCGCCGCAGGCATCCCGTCCGAGATC
TP23369	5H	180.09	bowman_contig_66744	TGCAGCCGCAAACCATTGG[G/T]ACAACGAGAGAGG CAGCAAGAGAAAGGGGACCTCCAGATCTGCC
TP44456	5H	180.09	barke_contig_1781958	TGCAGGCAGTTCATGTACTCGTCGCCGACCTCCGAC GACCCGAAGCTCCCCGTGTTGGAGCT[C/G]G
TP48597	5H	180.09	bowman_contig_66744	TGCAGGCTCCCGCAGCCGCTGCTCCGTTCATCC[A/C] CCAGCAGCACCAGTACGTCACTCAGACGGG
TP49325	5H	180.09	barke_contig_2783582	TGCAGGCTTTGCTCGGACAAATAACGACAAAACAG TTAGTGCAG[A/G]CAATCCTCCAAGAACATCA
TP53206	5H	180.09	barke_contig_1794363	TGCAGGGTTCA[A/C]AGCGGAGCCCCTGAGAGCGTG TTGCGGCGGGGGTGGGCGGTACAACTACAAC
TP56902	5H	180.09	-	TGCAGGTTAATTAGCAGGAGAC[A/G]AGCATTTATA GGCTAAACACATGATCACTAGGCTAGGCGGT
TP65419	5H	180.09	barke_contig_58717	TGCAGTTGTATATAAT[A/C]TATATAGGTCCAGATA AGCACCTTGTGGACCATGGATGATTACAGGT
TP22082	5H	180.09	barke_contig_428513	TGCAGC[C/T]CAGCTGCAAGGTCACATGGAGGAGAC GGCGAGGTGGCGTTGTGGTCCAGCAGCCAGG
TP22103	5H	181.39	morex_contig_139963	TGCAGCCCAGTCCACGAGCAGACCAGA[C/T]CTTCG TTCTCGATTCTTCCGCTCGAGAGGAAAAAAA
TP22358	5H	181.39	-	TGCAGCCCCGACCTACATGCGGCAGCTTCAGCAGCT TCAGGG[A/C]CTGGTGTAGCTGGATAGCAGA
TP39803	5H	181.39	bowman_contig_218365	TGCAGGAATCAGG[C/T]GATCGACGGCGAGCTCACC TGGCCGAGGTCCACCGCCGAGATCGGAAGAG
TP798	5H	181.39	morex_contig_47725	TGCAGAAATGGCGCAAGAGAGTTGGAAAGAGGCAG AGGATACGGGTGTCCACGCACC[C/T]GAGGCC
TP3343	5H	181.39	morex_contig_2550379	TGCAGAATTGAAAATC[G/T]TATATTCAGACAAACG AATTAGTTTGCAACTTCGATACGCTTACGGT
TP21169	5H	181.39	barke_contig_53394	TGCAGCCAGACTAATCTAACCTCCTACACAT[A/C]T GGACACGTCCGAGATCGGAAGAGCACACGTC
TP37989	5H	181.39	-	TGCAGCTTGCCAGCAGGGTTGGTCGGTTCTGGCTGG GGAGT[C/T]GGCGCGGCGGTCTCGAGGATGG
TP41742	5H	181.39	morex_contig_70254	TGCAGGAGCGCGCAAACATGCTGGTGCCGAGGAAG GCGATGCGC[A/G]GGACGCCGCGCTCCGCTGC
TP47606	5H	181.39	bowman_contig_67440	TGCAGGCGGAGAGCTGCGCGGTGG[A/G]CATCCTCG AATGGCTCGCGTCCCGAGATCGGAAGAGCAC
TP55695	5H	181.39	bowman_contig_860717	TGCAGGTGCAAATCTACTTAAGTTACTACTTCAT[G/ T]CCTCAGAAGGAACTGGCAATGATTTTTTT
TP58602	5H	181.39	morex_contig_46503	TGCAGTACTCTTTTTTTGATAGGATAGTATTTAAAA TTTGCTTCTATTGAGGCAAAGC[A/T]AGTAC
TP62039	5H	181.39	morex_contig_41924	TGCAGTGCAGTTCTTGACTCTTGTAACTCGTAAAAT ACAGTCGAACACTTACC[A/G]CCGTTGCACT
TP57657	5H	181.39	morex_contig_277371	TGCAGGTTGTTGAGTTAGATTTTCTGTATCCAAGTG

Marker_ID	Chr.	cM	Barley_Contig	Sequence
				AAGGAATCCATCAGCGATATGG[A/C]GCCGA
TP13481	5H	183.99	morex_contig_86226	TGCAGCACACGATCGATGAAGCAATAGTGCA[A/G]TACAGACACGAGTCTAGCTAGCTCACTTGCAT
TP39509	5H	183.99	bowman_contig_65023	TGCAGGAAGCTGTATGATTACGTGAGGTATGATCTAAAAGAGATCGCCTTCCCGTC[C/T]TCTCTGC
TP59332	5H	183.99	barke_contig_274072	TGCAGTATGGTAAAAAAGTTTCAAAT[A/G]TATTTATTTGGGGCAACCCTAGGAGCAGGCTACAACA
TP2242	5H	183.99	morex_contig_45652	TGCAGAAGCGATGTTATTGACGACA[A/G]CATTGGTGAAGACCGAGATCGGAAGAGCACACGTCTGA
TP3689	5H	183.99	barke_contig_1787638	TGCAG[A/G]CACTCTCGGACATGTCAAGCTAGCAAACAAAAATATTAGCCATTAACAGCTCTGCCGA
TP5236	5H	183.99	morex_contig_123972	TGCAGACGGGCCTGACGTGACCTCACCTC[A/G]CCACTAAACTATGATAATTCCCATAGTTTCGTTA
TP5448	5H	183.99	barke_contig_277063	TGCAGACTACAAAATGGAAAGGAGAACGG[C/T]TCCCCTTCACCACGATGCGCCCATTGTCGAGTAT
TP8990	5H	183.99	barke_contig_53425	TGCAGATCACAGACGCCGTGAGAAGACATTACT[A/G]GTAGCATAAGCGAAGCACGATGACCGATCT
TP27336	5H	183.99	morex_contig_123972	TGCAGCGCCACGTA[C/G]GCCTTCTCCAGCCTCGAGTTGTTGGTCGCCGAGATCGGAAGAGCACACG
TP31416	5H	183.99	bowman_contig_2014329	TGCAGCGTACGTCGTCGT[C/G]TTCCTCCAGTCCTCCAGTAGCATTGGGCCATTGGCTCTCCTGAAT
TP35059	5H	183.99	barke_contig_272783	TGCAGCTCTCGCAACTGCTTA[C/G]CCGCTCGCGGGACCGAGACGCCATCAACTCCATCATCTCTCG
TP37982	5H	183.99	barke_contig_544572	TGCAGCTTGCATTTTCCAAGAAAAATATGCAGCTTCGCACGCAACGCAACTCGT[C/T]ATCTCGTGT
TP39184	5H	183.99	morex_contig_54112	TGCAGGAACTCTCCTGCATGGCGTCGAAGAGCTCCGCCTTGAAGACG[A/G]ATGACCGAGCAGCAAG
TP52840	5H	183.99	barke_contig_271043	TGCAGGGTGAAGCTGATACATCAAGATGTTGCAGGCCACGCTTATCC[C/T]AATCAGGCTGTAGCGT
TP60335	5H	183.99	bowman_contig_853471	TGCAGTCCTCCGCCCACCACCACCCAGCAGCTCGTG[C/G]TCCGCCGCCGCCGAGATCGGAAGAGCA
TP62947	5H	183.99	morex_contig_42326	TGCAGTGGCTAGTTACCTTTGTCTGGCA[A/G]AGCACAATCGCGAGCTCTAATCCTAGTTGACGCTC
TP64061	5H	183.99	barke_contig_270250	TGCAGTTCACATTTGCCCTGTTTCTTTTTAATAAGCCGTT[G/T]CCATGGCCAGAACATGCACCGTT
TP64348	5H	183.99	morex_contig_8885	TGCAGTTCCTGATCCCAATCCGAGCTGCCCGATGCCCGTTCCCAGTTCG[A/G]TATCCCAATCCGAG
TP11543	5H	185.28	-	TGCAGATTGTGATACATCTATTTGAATGCTTAAGATCAAGATGAAACGAGTTCTTGAGTTT[C/G]CA
TP1550	5H	185.28	bowman_contig_1985505	TGCAGAACGTCCTGGTGCTGGGCGTCGGCC[C/T]TCGGGTAGAACCACAGGATGTTGTTCTGCACCA
TP3154	5H	185.28	-	TGCAGAATGATGCAGATTCATTGTTGCACT[G/T]TAGGGCTCAAGTTTCATGTTTCTTCTTTCTAAA

Marker_ID	Chr.	cM	Barley_Contig	Sequence
TP12018	5H	185.28	bowman_contig_112649	TGCAGCAAATACAGAGTATGCTTCCTTAT[C/T]GCGAAAATGCCTTTTGGAGCTCGGCCTCCTTATC
TP20996	5H	185.28	barke_contig_269667	TGCAGCCACTAGCAAAAGTTGGACCATGTCAGTGTCCTTATGTCCCTCAAGATCC[A/G]TGTCTCTA
TP32921	5H	185.28	-	TGCAGCTAGAAATATTGGTCACGGCACGG[G/T]GCGTCGCTGGGGAGCCTCACGGACATACTCAGA
TP46278	5H	185.28	barke_contig_57390	TGCAGGCCTCGACCCAGTTACATAT[A/G]TTGATGTTGATCAGCAAGAGAACACTGGAAGTTTGGGA
TP62437	5H	186.58	barke_contig_268803	TGCAGTGCTAAATGGGAAAGCGGGGCAGCAGGACCCACCCTGGAAATC[A/T]CGGGGGAGAAGGATT
TP11822	5H	187.88	barke_contig_2791648	TGCAGCAAACACACC[A/G]AGTTGCAAACATCATGATAAGACACCTAGTATCATGACATGGTCAGGA
TP4844	5H	187.88	morex_contig_53026	TGCAGACGCAGGTATAAACTGGAGTT[C/G]ATCCAGGAACATACATCAATAGTGATCTCGCGATCTA
TP40414	5H	187.88	morex_contig_2548295	TGCAGGACCCGAGAAGAAATCAAAATGAAGGCCGCC[A/C]CCGAGATCGGAAGAGCACACGTCTGAA
TP43885	5H	187.88	bowman_contig_859458	TGCAGGCACATCGTGGCCACTTGTCTCCACAGGAATGTCCTC[A/G]TAGGCATCAAAATTGATACCT
TP44185	5H	187.88	bowman_contig_66544	TGCAGGCAGCAGAACCATGTTGCATGTTCATGGGTGTGGGAGGCATC[A/G]GGTACATGCTAGGCGT
TP45290	5H	187.88	bowman_contig_229848	TGCAGGCCCCCTGCTTCTGGGGAGATCGAGATCTGCCACT[C/T]GCTGCTGTCCGAGATCGGAAGAG
TP49768	5H	187.88	barke_contig_606190	TGCAGGGAGACGAACCTCAACGCCC[A/T]CGGGCAATATCCAGATCGAGCCCAAGCCCACCTACCTG
TP50488	5H	187.88	morex_contig_244290	TGCAGGGCATGAGTCCTTGAGGCCAACGGTGTGTCACACTCTATCACATG[A/G]TCGTGGAACTGGC
TP65214	5H	187.88	morex_contig_58034	TGCAGTTGGAAGTCCAAATATTAGTTAGAGAGTTAAATGTAAAA[A/G]GCAAGTTCTTATTTCGTGC
TP30029	5H	187.88	barke_contig_1790225	TGCAGCGGCCATGCCTAGGTGAAGCTCACACGTGTTCATGCACTGTGTTGTGGA[G/T]GCATGGTCG
TP4477	5H	187.88	morex_contig_89703	TGCAGACCTAATCGGTGCGTCTACGGATCGATGGG[C/T]TTTGTGCGGGAGCAAGCCCAGTTAACCA
TP37917	5H	187.88	bowman_contig_1984825	TGCAGCTTGAGCACCTTGAACCCGTCGTGCAC[G/A]GCGATGAAGTGGCGTTCGGGGCTGTACATGA
TP42000	5H	187.88	barke_contig_2787691	TGCAGGAGGAATGGACGCAGCGG[A/G]TGAGTAGAACCTGTTCGTGGGCGTGGGAAGCATCGGGGAC
TP61711	5H	187.88	morex_contig_442138	TGCAGTGATCAATCCGCTGCCGTATTGCGGCTATTGC[C/T]TTTGCATGCGTGAGCTGAGGCAGGTA
TP62143	5H	187.88	morex_contig_1566620	TGCAGTGCCCAC[A/G]CCGCGGCGAGCCACAGCGCCGCCAGAGGAGAGCGCCGAGATCGGAAGAGCA
TP41746	5H	190.56	morex_contig_242440	TGCAGGAGCGCGCGGCGGGC[C/T]TCGTCGGTGGGCAGCTTGCGGGCCGACTCCTCGAGGTCAGCGA
TP6753	5H	191.72	morex_contig_85302	TGCAGAGCCTCTACAACCTCGGAGCGCGCAAGGTG

Marker_ID	Chr.	cM	Barley_Contig	Sequence
				TTGTTCCTTGGCACGGG[A/G]CCGAGATCGGA
TP32903	5H	194.39	barke_contig_512818	TGCAGCTACTGTTGGTCGTAG[C/T]AGCGTCAATCTACCGCAAGCCCAACCACATACAGGGATAACA
TP39096	5H	194.39	barke_contig_13080	TGCAGGAACGCCATCCTCCCGAGACCCCTGCGAAACAC[A/G]GCGCGGATTTTAGATTGGATGATTG
TP54281	5H	194.39	barke_contig_1799706	TGCAGGTCAGGTAGCTTGACTTGAGTTCTGATCTGAACCGTTAAGGGTCGTTGTCACTCGCCT[C/G]
TP61839	5H	194.39	barke_contig_271923	TGCAGTGCAACACGTGGTGCT[A/G]CCAACGGACCTCGCCGAGATCGGAAGAGCACACGTCTGAACT
TP36470	5H	195.69	morex_contig_1559810	TGCAGCTGGACCAGACGGCGACAAGCGGGCCAAGAAAACGAGCCTGG[A/G]AGGAGGAGGGTTCGGT
TP5610	5H	195.69	barke_contig_402615	TGCAGACTGATCTGAAGTCCCAGCAATGTCGCT[C/G]CCAGCTCTAGTATGCATCCGCGCTGCACGC
TP10176	5H	195.69	bowman_contig_62424	TGCAGATGACGGGCGATGTGTCATCCGTTCATGTGTAGCCTC[A/G]TGGCTCCGTAACGGCGCCATC
TP21943	5H	195.69	morex_contig_316065	TGCAGCCCAACACTCATACTA[C/G]GCAGCGTAGCTCCGATTCCGACGGTGAACTACGATGGAAAAC
TP37968	5H	195.69	bowman_contig_223211	TGCAGCTTGCACGCGTCCATTGGGCGACTCGTACGGGCGG[A/G]CCCGAAAAAAAAAAAAAAAAAA
TP51503	5H	195.69	bowman_contig_127042	TGCAGGGCTGACGGAGATTGCAGC[C/T]AGGGGCGGCATTGCCAGGACTTCGGAGCCGCTTGCAGCA
TP12617	5H	196.99	barke_contig_64286	TGCAGCAACTTGTTCAGGTATCTCTTCGGCCG[T/C]GACTCCCGAGATCGGAAGAGCACACGTCTGA
TP18291	5H	196.99	-	TGCAGCAGGTCCAGCAGCTTGGCGTC[G/T]TGCTGCCTCGTCCACGCGTCCACGTGCAGCCTCGAGA
TP27267	5H	196.99	-	TGCAGCGCC[A/G]CACGGCGGGACGAGGCGGCGGCGTGGGCGAGCTGACCGAGATCGGAAGAGCACA
TP34977	5H	196.99	bowman_contig_1276882	TGCAGCTCTAGAAGTTGTGGCATGATCCGAGCGGGATCCCCTTGCC[A/G]CCCTGGCAGCCGACGGA
TP37969	5H	196.99	bowman_contig_223211	TGCAGCTTGCACGCGTCCATTGGGCGACTCGTACGGGCGG[A/G]CCCGAGATCGGAAGAGCACACGT
TP66033	5H	196.99	barke_contig_66480	TGCAGTTTT[C/T]GATGAAGGGGCGGAGGATGCCTATATCCAGAATACAAGTGCAGAGTAGTTTGTG
TP60726	5H	198.29	morex_contig_142158	TGCAGTCGCTGGTGCCG[A/C]AGCTGCCGACGTGCTGCCGAGATCGGAAGAGCACACGTCTGAACTC
TP64809	5H	198.29	morex_contig_13130	TGCAGTTGA[A/G]TGAAGAGAGCACACCACATCAGATCAGAGCAGATAGATACACACAAACACATCA
TP10921	5H	200.89	bowman_contig_201794	TGCAG[A/G]TGGTGTCTACATCTGGAGCAACGAAGTTTCCTTCTAACATTCCCCGTTCCACGCATGT
TP11091	5H	200.89	bowman_contig_11993	TGCAGATGTTTCTTCATCAAACTCAATGCAAGTGCACTGCTG[C/T]ACAAAAATCCAGTGATGGTCT
TP34588	5H	200.89	morex_contig_2551295	TGCAGCTCGCGGAAGTCCCGCTCCAAGCCCTC[G/C]ACCCTCCTCTCGCCGCGCCGCTGCGGCGCCG

Marker_ID	Chr.	cM	Barley_Contig	Sequence
TP30169	5H	200.89	morex_contig_1567717	TGCAGCGGCGAGACCAGGCCCGAGTGCTTCAGGTACAGGGTGCTGGGCCTTCCGCGGGGG[A/C]GGC
TP59893	5H	200.89	morex_contig_137174	TGCAGTCATGGGGTGCTCCCTCGTGGTGTACCTC[G/T]CGGGGTGGAGGACGTACCGAGATCGGAAG
TP4070	5H	200.89	morex_contig_1563308	TGCAGACCAGCCCGAAATATCTGTTTGCGTAC[G/C]TGGATGCGATGGCTCAGTATCAGATCTGAGA
TP50175	5H	200.89	bowman_contig_128539	TGCAGGGATTTTATGTGCTCGAATTTTTTGCGGGTTCAG[A/G]ATTTCTGTGATGTGGAGAATCTG
TP55790	5H	200.89	morex_contig_2547596	TGCAGGTGCAGGACGAGC[C/T]CAACTGAACTCTCGGCCGTCTTCCAACGGCTTCAGTCCGACGTAC
TP55260	5H	202.19	barke_contig_395496	TGCAGGTCTCACCATAGGTTGTTGACCTCTACGTCCCACCCAAC[C/G]CTGCCGCTGGAGCCAAAAC
TP13050	5H	202.19	morex_contig_319918	TGCAGCAATAACATGTAACTCA[A/T]CGGCGGCAGGGCGGCGCCGACGGAGGGCCTCGGGTCGGCCG
TP30172	5H	202.19	bowman_contig_846417	TGCAGCGGCGAGAGAGAAAGATTCG[A/G]GGCGAGGGGGAGGGGACAAGAACTAAACGAGAGCAAGA
TP45761	5H	202.19	barke_contig_1789262	TGCAGGCCGAGTCCA[A/G]GAAGTCGACGAGGGCGTGGGCCGTAGTCACGGTGCGCATCAGCGTACG
TP48193	5H	202.19	-	TGCAGGCGTCG[G/T]TTAAAATGCATTTTGCTTAAGTAATAGGCCGAGATCGGAAGAGCACACGTCT
TP53507	5H	202.19	morex_contig_91427	TGCAGGTACACGCCGATGGCCGTGAACTTGATGAAGT[G/T]GCCGCCGATCTCCATCCCGCGCACGC
TP62277	5H	202.19	barke_contig_62361	TGCAGTGCGAAGGTCGCTGTTTTGAAGGTGACGAGAG[A/G]GGCTTTTGTTTACGGAATAATTCATT
TP19027	5H	203.49	morex_contig_60807	TGCAGCATATTGCATTGACTGAACAACTTCACCCGTGCATGCAAGCTGTACT[A/G]CCCTATTTGAT
TP27254	5H	203.49	bowman_contig_62237	TGCAGCGCCAAGCTCCGTCCTTCCGTGCAG[A/G]CCTGCAACATCGTCGTCAGGGCTCTGGGCAGGA
TP14830	5H	203.49	-	TGCAGCACGGGGATTGGCT[C/G]GCTGTTAGTCTGTCTGTCGGACCATCCATCCCTCCATGGCGCCG
TP34723	5H	203.49	bowman_contig_1988567	TGCAGCTCGGCTCAGCTCCTACCTCTG[C/G]GACGAAACGTAGGTTTAGGTGGTGCCTGCCTACCTG
TP60737	5H	203.49	morex_contig_134518	TGCAGTCGCTTCC[A/G]CCGCACGGGGAGAAAGACGGCGCGTGTTCGCTGCCGATGGGGAAGGTGCG
TP55232	5H	204.79	barke_contig_503370	TGCAGGTCTAACTTATCAAAGAACTCATCCAAGTCATCATCTTT[C/T]CCTTGGGATCCACGATTTG
TP10789	5H	204.79	barke_contig_272710	TGCAGATGGCTTCTCATGGCGTATTATCGCCTCCA[C/T]CGCCAGCACCCAGGGTTAATTGCTTGCT
TP19964	5H	204.79	barke_contig_276429	TGCAGCATGTGGATTCGAGCGGCCTGAC[C/T]CACCTGATCCCACAAAATAATCCCATAGGTTCTTT
TP1197	5H	204.79	bowman_contig_14487	TGCAGAACCAGAGCTGGAGCGGCCTCGCCTTCGTCATCCTCCCTTTCTGGCTAAACACT[A/G]CCGC
TP2211	5H	204.79	barke_contig_148484	TGCAGA[A/G]GCCGACAAGTTTGGCGTTTCGACGCC

Marker_ID	Chr.	cM	Barley_Contig	Sequence
				GTTCCTTGGCTCGGTGCGTGCGGCCTTGAAC
TP2234	5H	204.79	barke_contig_272710	TGCAGAAGCGAACGATCGAACAAATGGTCTCTCCTCCGCCGATGAGC[A/G]CTCTCGCCCGTTAAAT
TP30119	5H	204.79	morex_contig_145048	TGCAGCGGCCTGCACATTCCAGCGTGGAGTGCCAC[A/G]ATGCCGAGATCGGAAGAGCACACGTCTG
TP42582	5H	204.79	bowman_contig_1983327	TGCAGGAGTGGAAAAACTACGGTTGC[C/G]AGAACCATATATCATCGGTATGGACGGGAGTTCCAG
TP45615	5H	204.79	bowman_contig_240	TGCAGGCCGAAGATTCAGAACAGCAGAATCAAAACGCACAAGTAA[C/G]TGGGTGTGTGGGGGTGGA
TP57330	5H	204.79	-	TGCAGGTTGAGGAGTTGGCGGCGGCGGAGAGGGCGGCGG[C/T]GGGCGGGGTGAGGATGATGCTGAT
TP57942	5H	204.79	bowman_contig_65586	TGCAGTAACCACTAGTTTGTCACC[A/G]TACTTGAAAATTTTTGTTTGTTTTATTTCTATTTATTCT
TP60516	5H	204.79	barke_contig_479626	TGCAGTCGATCGACCTCCACACCTCTTTCAGACAAAACATCAAGCGCATG[A/G]GCTGCATTAGTCA
TP62529	5H	206.09	morex_contig_67914	TGCAGTGCTGCTCAT[C/T]GCCTACGTTCCCAAGGCGTACAGACGCTGGTCGAGGGACGCAGGCAGC
TP35230	5H	207.39	barke_contig_358023	TGCAGCTCTTGCCACAGCA[C/T]AGCTAACATTCTGTCCATCACTTGCCTGCGAGACCACCCTCTTA
TP53633	5H	207.39	-	TGCAGGTACGCTTCCAAACTAAAGCACCCGATCT[C/T]CTCTCCGAGATCGGAAGAGCACACGTCTG
TP17247	5H	209.99	morex_contig_156786	TGCAGCAGCTCGTCGCCCGCGAGACCAACCCGCTCCAAGGCGC[A/G]GTGGTGTCGGTGACCACCAT
TP22583	5H	209.99	morex_contig_156786	TGCAGCCCGAATTCCGAACACTTCTTCTACCTCTCAACAACAGAAACAGAGGCGAA[A/T]GCAGAGC
TP31831	5H	209.99	bowman_contig_11564	TGCAGCGTCTACAAAAGAATGCTTTGAAGTACCGCCAAGAGCAATCGGGTT[A/G]ATCTCTGAGAGA
TP39189	5H	209.99	barke_contig_582834	TGCAGGAACTCTTGACTTGGTAATCTCCAAACATTCCACATCTGAGACCAA[A/G]CCCCTCTTGCAT
TP45424	5H	209.99	bowman_contig_864780	TGCAGGCCCTCAGAAATCCCTTTAATTATTTTGTAGCACGTTCGCCACTCA[C/T]GATCTGCATCTA
TP44555	5H	211.23	barke_contig_518669	TGCAGGCATGGCGTGGAGCAACCACGAAACCCCTGGCA[C/T]GACGGCCCAGAGTTCATAACGCAGC
TP3650	5H	211.23	-	TGCAGACACCTA[A/C]CTCCGCCTCTGTTGTGCCCGTGCAAGCCGCCGTCCGCCTCGCCGAGATCGG
TP8766	5H	211.23	barke_contig_408575	TGCAGATAGGTGTGGCACTGTTGATTAGTGTTTGTGGATTTTTTGGAGCGCA[G/T]GTAGAGGAAGT
TP25323	5H	211.23	bowman_contig_200307	TGCAGCCTTACTGCATGCCTTAGCTCAACCTCTTTCATCTTATTAGGTCTCTGTCCTCCT[A/C]TAG
TP33692	5H	211.85	-	TGCAGCTCCACCCCTTGCACATGTACCTCGGGGACCCTCAACGTGAGCTCG[C/T]CACCACCAAGGC
TP5429	5H	212.47	morex_contig_2546879	TGCAGACGTTGTCGTTCCCCTTCACCTACA[C/T]GTCGCCTCCTCGGGGCCTTTCACCTTGACCATG

Marker_ID	Chr.	cM	Barley_Contig	Sequence
TP4670	5H	212.47	bowman_contig_66345	TGCAGACGACCAATACAAAGGTTGCATAATAATAT TTTTAGA[G/T]GATATGAATCATGAATAATAC
TP11745	5H	212.47	barke_contig_272767	TGCAGC[A/G]AAAACCGTACCCAAAAATCGATTTTT TAAAACAGAATTTTGCATTTCGATAAATTTA
TP30240	5H	212.47	bowman_contig_865289	TGCAGCGGCGCCAAGGACGGTGGCGCTGTAGCGG[C /T]GGCTCCGTGGCCTGCCGAGATCGGAAGAG
TP49334	5H	212.47	bowman_contig_222682	TGCAGGCTTTT[A/T]AATTGTGTGAATTGTAGGACGC CACTTGTGTAGCGGCGACCAGTATGCGTGT
TP59128	5H	212.47	bowman_contig_126736	TGCAGTATACTCGTTTATTATGAATGGATGGAGGGT TGTA[G/T]ATCTAACCTTGCTTGCTGGCCTT
TP60289	5H	212.47	bowman_contig_1982916	TGCAGT[C/G]CGTGCAGTGGAGTGCTTTCGTGGCTTT GCTCTGCTTCGTTAGTCTTCTGGGCTGGAG
TP63592	5H	212.47	barke_contig_272767	TGCAGTGTTCAAGTACAACGTAATGGCGCACGACA TGGTGACAATGAGCTCAG[C/T]GGGGTACAAA
TP5406	5H	219.43	morex_contig_43922	TGCAGACGTTCATGTTGACTCACACTCCTGAGACCT AGCCGAAATCTTTTTTTTT[A/T]AAAGGAGG
TP35246	5H	220.20	bowman_contig_848115	TGCAGCTCTTTAAACCATAAACCAGTACATAAACCA GTACTCCTGGCAGT[A/C]AAAAATCTCTGCA
TP63322	5H	225.82	morex_contig_1563668	TGCAGTGTACTTTATCCAGACGACT[A/G]TAAAAGG GGATTGTTGGTCTAGTTTGATGAATTTACTA
TP48164	5H	226.86	bowman_contig_69746	TGCAGGCGTCGATTAAAGTGCATTTTTCTTAATCGG TGCCCGCAGC[A/G]CCGAGATCGGAAGAGCA
TP1757	5H	226.86	bowman_contig_15108	TGCAGAAGAAATAAGCCTTCCCTTTCCCCTCGCCGA GGTCCATGC[A/C]CAGACCATGGTCGCGTGT
TP14416	5H	229.49	morex_contig_42299	TGCAGCACGAAGATGGTCTTCGTCGGCTACGA[T/A] GCGGGCTCAAAGGCGTATAGGTTGTATGATC
TP27410	5H	229.49	barke_contig_1805875	TGCAGCGCCAGCGTGAACGGGGGTGTGGCGCTGAC GACTTAGGCGCCACATAGT[A/G]TAGTGTGGC
TP53372	5H	229.49	morex_contig_1647134	TGCAGGTAAACACGCCTC[A/G]TGCGGGGAGGGCCT CGCGTAGAACTTCGCGATGTTATCCCCGAGA
TP57064	5H	229.49	bowman_contig_64048	TGCAGGTTCATCCACTACCCTCCTTCTCAAGGCAAC C[G/T]TCATCGCCGCCATGCTCGCAGTCCTA
TP58662	5H	229.49	barke_contig_423851	TGCAGT[A/G]GAAATCAGTAAGGCATACAAATCACC ATTTCACATTTCACATCTGTAACGCGGTGGA
TP168	5H	232.09	morex_contig_2553377	TGCAGAAAAGCC[C/T]GACTTCGAATTAACAAAGCC ATCAACCGAGATCGGAAGAGCACACGTCTGA
TP9435	5H	232.09	barke_contig_505944	TGCAG[A/G]TCCTTGCAGCTCATTCCTACGGCCTGAG CCCACTCCGAGATCGGAAGAGCACACGTCT
TP20446	5H	232.09	barke_contig_360216	TGCAGCCAAGTGAAGTTTGTTGATGGACCACCTTGT CTGTGTGAGT[C/G]TGGCTAGATCCGAGATC
TP22613	5H	232.09	barke_contig_554943	TGCAGCCCGAGATGTAGTTCCCCCGCAA[A/G]TGAA AGGTAAATAGAAAAGATGGTACGTGCCAAAA
TP48606	5H	232.09	-	TGCAGGCTCCGCCTCCTCCTCCTCCT[C/G]TTCCTCC

Marker_ID	Chr.	cM	Barley_Contig	Sequence
				TCCGAGATCGGAAGAGCACACGTCTGAACT
TP16034	5H	232.09	bowman_contig_12310	TGCAGCAGCACCGCCAG[A/C]CGAGCAAGACAAAC CCAAAGAGGAACCACCACCGCCGCCGAGATCG
TP52094	5H	233.32	bowman_contig_849388	TGCAGGGGCTTCGTCGACCGATGACGCATCGGAAT CCATTGTTGAAGTATGGCTCTTCTTCCC[A/G]
TP4268	5H	236.00	morex_contig_38449	TGCAGACCCTGGTAGACCACAGCAC[C/G]AACCCCA TGCCCATCCCGAACATGAGCCCGCCGCCCCG
TP5836	5H	236.00	bowman_contig_13213	TGCAGAGAAGCCA[G/T]ATCTATTGGGCCAATCCGA AATGAACCTTGCAGGCCCACCCACCCAGGCC
TP9934	5H	236.00	morex_contig_44270	TGCAGATCTCATCAACAGAATATCTTTCCAGGTAAA TCCTTTTTACAGTTTCAC[C/T]ATGGTTTTC
TP32941	5H	236.00	morex_contig_158733	TGCAGCTAGAG[A/G]AACTACAACCACGGGAATCAC CCACAACATGCTCCTGCGTTTGTTGTTGAGA
TP38332	5H	236.00	barke_contig_425270	TGCAGCTTTCACTCCAGCATCTCTCC[C/T]GTCTGCC TCTGTTTTTAAACCAAACAGAGCAAACATG
TP57848	5H	236.00	-	TGCAGTAAAAACGGATCGTACGTACATATATACGT ACATTAGATTTGGT[C/T]TACCGTGGAGTAAT
TP65691	5H	236.00	morex_contig_1577539	TGCAGTTTCCATGACCAGTGGC[A/G]ACAGGTTCTC GTCCTCGGAGATATTGGCCGAGATCGGAAGA
TP50222	5H	238.59	bowman_contig_88215	TGCAGGGCAAC[G/T]TTGACGTCCTCGCTCCGCAGC AGCATCTCAGACACCTCCGCCGAGATCGGAA
TP58969	5H	238.59	morex_contig_140269	TGCAGTAGGGGAGCACGTTGCCGTCCACCACGTCGT CCTCCTGCTGCTGCACCTG[A/C]CCTTGCTG
TP63837	5H	238.59	barke_contig_282096	TGCAGTTACTATGGTGCATGTTCCTC[A/G]TCGGGAT GGCTAGGCTAATACGAGCCATCTCGTGGAC
TP66058	5H	238.59	barke_contig_305794	TGCAGTTTTGAGATAACAATTAAAAAATCTGATATG AAAAAG[C/T]CACATCCGACGTAACTAACAG
TP37385	5H	238.59	bowman_contig_117472	TGCAGCTTC[A/G]ACACCATGACCAGGGAAGACAAA CCCCCCAAGGTATACACCGACACCGACTCAG
HMA2	6H	26.3		GTGAGGGGATTTAACGGTGAAATTGG[T/C]GGAGAG GGCGTATATGTTGGGAACA
TP1401	6H	35.00	bowman_contig_222947	TGCAGAACGACGGCGT[G/T]GTGAACCCCGAGGCCA TCATGATGCAGGCCATCGCCGAGATCGGAAG
TP10615	6H	35.00	morex_contig_37357	TGCAGATGCTGGTGATGATATCC[A/T]CCCAGCACC TGGTGGTGCAGCTGGTAGTGGTCCCCGAGAT
TP15309	6H	35.00	morex_contig_37357	TGCAGCAGAACCCCGTCATCTCCAAGAACCTGCCAT GGATCATCATCAATTCATCATCATC[A/C]AG
TP19558	6H	35.00	bowman_contig_62186	TGCAGCATGACACCCT[C/T]GTAATGACTCGGGACG CCATCAACAGAATCGTTGAGCGGATGCACAC
TP43077	6H	35.00	morex_contig_37912	TGCAGG[A/G]TCTTCCACAACCACTGCAAGGACAAC GGCATCGCCCTGGAGGACAAGAACTTCACCC
TP52483	6H	35.00	morex_contig_37357	TGCAGGGGTGTTGGATCATGAGCAAAGCATAGGGC CA[G/T]TTTGGGGGTGCACTTCAGAAACGCAG

Marker_ID	Chr.	cM	Barley_Contig	Sequence
TP56183	6H	35.00	morex_contig_37357	TGCAGGTGCTGTGCTCCCACAAGTC[G/T]GGGCGCT TCGTCCGCATCATGGACGAGATCGCCGCCCT
TP61626	6H	35.00	bowman_contig_846256	TGCAGTGAGCAGGCCG[A/T]CGTCCCTCTTTGCTTGG CCGAGGTAGTTGATTGCCGAATGCACTAGC
TP63346	6H	35.00	morex_contig_114452	TGCAGTGTATTATCTCCTAATAATCTTCAGACAATG CAACGAAGGAGGATGTGT[C/T]TGTTCTTTC
TP26274	6H	85.00	barke_contig_58737	TGCAGCGAGCGGTTACTGACGCCTGCTCATGTTTGC AACG[A/G]CAGCAACCTCTCAGGGTTCAATC
TP24379	6H	85.00	bowman_contig_845032	TGCAGC[C/G]GTGTGTAGGTTTAAACGGAAGCAAGT GCGTACGTACGTACAGCGCCGAGATCGGAAG
TP21231	6H	85.00	barke_contig_58737	TGCAGCCAG[C/T]AAATGGAGCAATAAGATTTCGCA AGCAACGGAGAGCTGCTTACAGAGCTGCAAC
TP13410	6H	85.00	bowman_contig_151876	TGCAGCACAATCTAATTCTTTTATAATTATTCCTTCA TTGGTG[A/T]GTATAGACCGAGATCGGAAG
TP49189	6H	86.24	morex_contig_1564053	TGCAGGCTTAGGTGTGTGTGTGCATGTGAGTTTACT GTGT[C/T]GGACAGGATTGTTTAACTCATAT
TP12107	6H	86.24	morex_contig_2547976	TGCAG[C/T]AACAAATAGCGCCCTGTTACTCATCCTC CTTCCGTAGAAAATCGCGAATTATACAAAA
TP24352	6H	92.39	morex_contig_162970	TGCAGCCGTGGGGATAACCGTGCGTGAGACCG[T/C] GAAGAGATGCGATGTGTTACAAGCCGAGATC
TP50437	6H	117.60	-	TGCAGGGCATACGAAGATC[A/G]GTAAGTAAGTTCA GTTCACCGAGATCGGAAGAGCACACGTCTGA
TP34958	6H	117.60	morex_contig_50809	TGCAGCTCGTTTTGTTTATGG[A/G]GTGATACAGCGA GCCAGCGACTGAATATTCAGCGGCGCGACC
TP64958	6H	117.60	bowman_contig_63088	TGCAGTTGCACGTCCGTGTACACGCGCACAACTAGT TACATGTTGCTTGGGTTTGTAC[C/G]AGATC
TP64683	6H	117.60	morex_contig_2549403	TGCAGTTCTGACTGATCCTTTGTTGTTGTCCTT[A/C]T TGTTGCGCAGACGAAGGGAACGGGCAGCT
TP32398	6H	117.60	morex_contig_45229	TGCAGCGTTGAACGCCCGCCAAAAAATATATCGGC GTGTTTGGTG[C/G]CCTGCATGCAGCCGAGAT
TP30146	6H	117.60	bowman_contig_13308	TGCAGCGGCG[A/C]CCGCTCACAGACGCATCATTAT CCACCTCCAGCCACGCCGAGATCGGAAGAGC
TP28433	6H	117.60	bowman_contig_860499	TGCAGCGCGCAAGCTGTGGAAAGCACGGCGCAATT AGCAAGGCCACATGTCGTC[A/G]GTCAGTACT
TP26484	6H	117.60	bowman_contig_861033	TGCAGCGAGTTATGGGCCAAACGCACACAAGGTAG CGAGGCGAAGGATTTT[A/G]ATGAGCTGGCCC
TP9913	6H	117.60	barke_contig_7267	TGCAG[A/G]TCTAGATGGATCGGGTCGAGCTTGCAG ATAACAGTTTATAAAGCAGCAAGAGCTCACT
TP9184	6H	117.60	barke_contig_1786619	TGCAGATCATTCTGCACCCGAACCAGGGACGTGTTT TGAC[C/T]GTCCGCGAGAACGCTAGGCTGCA
TP6707	6H	117.60	bowman_contig_22879	TGCAGAGCCGCGTGAAGAGCTCCTTCTTGGACTTGG AGCAGTCGC[A/G]GCGGTCATCGTCGTCGTC
TP3983	6H	117.60	-	TGCAGACCAAGATACAC[A/G]TACGTAGTCTACAAT

Marker_ID	Chr.	cM	Barley_Contig	Sequence
				CATTACCAAATGATTACGTACCTCTCACTGG
TP34790	6H	118.90	-	TGCAGCTCGGTCATGGCCGCAGGGGA[C/T]AGAATG TCGGGCCGAGATCGGAAGAGCACACGTCTGA
TP10055	6H	118.90	-	TGCAG[A/G]TGAAACGAAGGTGGGAATAGCGAATT AAGTTGGTGGGTTAGACGGGTCGGATCAAAGC
TP24224	6H	118.91	morex_contig_157599	TGCAGCCGTC[A/T]ACCAGCAGCACCGTGTCGTTGTT GCTCCGCCGCATCGTCGGACGCTGCTGCTG
TP59185	6H	118.91	morex_contig_136042	TGCAGTATCCA[A/C]AGAATCGGCATCGGTTACTTT GAGCAAGGGTATATCATTAACTGGTAAACTT
TP34767	6H	121.50	bowman_contig_954043	TGCAGCTCGGGGAGATAAGAGAGTGAGAGTGAGTG CACTAGTGGAAAACGGG[C/T]CTTTGGCCTGG
TP3084	6H	121.50	barke_contig_321152	TGCAGAATCTCTACAAA[A/G]CTCGGACAGTCTAGT CAAGCACAGGATCGAACCGAGATCGGAAGAG
TP59832	6H	121.50	bowman_contig_64727	TGCAGTCATAGAAAATGTGGGGCAAAGAAGGGAAA T[A/G]TGGAGCAGCTTCTGTTGTGACCTGGAG
TP49009	6H	121.50	-	TGCAGG[C/T]TGGACATGCACATCATTGTGGTCGGC AAGTGGCCGATCGATGGTGAGGTTGGTATGG
TP41558	6H	121.50	morex_contig_38713	TGCAGGAGCAGCAGCAGCAGC[A/G]GCGGCGGCGG CTGGTTGATTTGATCTGATTAGTTGAGAATCA
TP39275	6H	121.50	morex_contig_41348	TGCAGGAAGAAGCTGCGCGCCGCCGTGAGGGGCCT CAGGCAGGCCGC[G/T]GGCGTGCAGGCGGTGC
TP20968	6H	121.50	-	TGCAGCCA[C/G]GTACGTCGCTCTCGTCCGTCCGTCC ATTTGTTCCACTACTTGTGCTATATATATC
TP59279	6H	121.50	-	TGCAGTATGATGAACTTCATT[A/T]AGACACAATTC AAATGAAGCGAAATTTTCCGAATGTAAACAA
TP58067	6H	121.50	morex_contig_1582761	TGCAGTAATATAGTCAAAAGAATTCCAGTGCT[G/T] GCTTCCTTCCGAGATCGGAAGAGCACACGTC
TP48037	6H	121.50	bowman_contig_1985801	TGCAGGCGGTGCTCATGGACCAGAACCACGGCGGC GGC[A/G]CCTCCGCCGAGGAGCTCAGCATGTA
TP40151	6H	121.50	barke_contig_63367	TGCAGGACACTTTTC[A/G]TAGTTCATTTACACATGG CACCTTCAAAATAATCCAAGCAACTAGGAA
TP6138	6H	121.50	bowman_contig_845528	TGCAGAGAGGCGTCAAGTTGATGAACGTGTCCAGA AGATCATTGAATT[A/G]AAAAATAAGGTATTT
TP38093	6H	121.50	morex_contig_1567204	TGCAGCTTGGACCGCAGGCAGCCTGTTCGACGAAA TGT[C/G]CAGGAGGCTGAAGGAGGAGGGAAGC
TP21840	6H	121.50	bowman_contig_62231	TGCAGCCATGTGTTGCTCCCATG[A/G]AAGTGATTG GTTGAAAGTTGAAATTAACATCTATAACAGA
TP7744	6H	121.50	bowman_contig_845528	TGCAGAGGGCTGTGATTGAA[C/T]TGAATTAAACGG AAACAAAATGATGTTAGTACAGTTGTTAGTA
TP62784	6H	121.50	morex_contig_46762	TGCAGTGGCAAGGAAGAAGAAATGTAGTTTCAGTT T[A/C]TGGTTCCCATGCTGTAAAAGCAACACT
TP10679	6H	122.80	bowman_contig_849014	TGCAGATGGAGAGAGCAGGGATGAGATGAATAAGC CATCCACACACAAAGATCAAAGGA[A/C]GAGA

Marker_ID	Chr.	cM	Barley_Contig	Sequence
TP2233	6H	122.80	bowman_contig_251246	TGCAGAAGCCTTTGCTGCCATGACGACCCG[A/C]CA CCCTGTCCGAGATCGGAAGAGCACACGTCTG
TP421	6H	122.80	morex_contig_59606	TGCAGAAACTCAATAGAAGACGTTGACCAAAA[G/A] AAAGGAAAATGAATCGCTTGGACAAAGACAC
TP64895	6H	124.10	barke_contig_512992	TGCAGTTGATAATCTCAAGTTGGCTGAGTTGAGGGT GTTCCACTTGTAGAGAAGACGAGAT[C/G]TC
TP55808	6H	124.10	barke_contig_199432	TGCAGGTGCAGGTCAAAACTAACTAAAATCCGTCC GT[C/G]GAGAAGGAAATCGAGAATGCATGCAT
TP43100	6H	124.10	-	TGCAGGATGAAGCAAAGGCTCAATATGGCGCGTCC TC[A/G]TCTGGATCCCGTGAGAACCTTTATCC
TP32229	6H	124.10	morex_contig_136822	TGCAG[C/T]GTGGTCAAAAGCAACAGGGATTAGGGA TAACACTCATGCTACAGCCTTCAGTAACAGG
TP19126	6H	124.10	morex_contig_40556	TGCAGCATCAGGTTGCACGA[A/G]CAACAGAGGCGC AGGAGCCACCTGCACCTGCTCCGAGATCGGA
TP8867	6H	124.10	bowman_contig_855242	TGCAGATATTATGGCGAGTCCGACCTCCTCGGCTTC GACGACGACGGCGG[C/T]GACAAACACGGCG
TP4533	6H	124.10	-	TGCAGACCTGAACGTC[A/G]ATGTCAGGTCAGACAT GGAGGTGCTCCGAGATCGGAAGAGCACACGT
TP35032	6H	125.40	bowman_contig_63959	TGCAGCTCTCCCTCCTCACCCCATACATCCAGGTAA CTCCCC[A/G]CCTCGCCGAGATCGGAAGAGC
TP25247	6H	125.40	barke_contig_303061	TGCAGCCTGTAGCCGTCGAGCTCGTCGATCACATCG TCG[A/G]CATCGTAGAGCAGCTCCTTGACGG
TP6267	6H	125.40	bowman_contig_85559	TGCAGAGATGGACGGCGTGGAGAGCCAGATACCCG ATCCCGATGG[A/G]CATCAAGAGATGAATAAT
TP41963	6H	125.40	barke_contig_64472	TGCAGGAGCTTGTCATGGAGGAC[A/G]TGTGTAATT TCCGAGATCGGAAGAGCACACGTCTGAACTC
TP11284	6H	125.40	barke_contig_465957	TGCAGATTCGATAATGT[A/G]GAGTGCTATCTAAAA TCGGTGTTGTCGTCAATCCATCCACATAACG
TP64253	6H	126.70	barke_contig_67682	TGCAGTTCCATATAGCCCAAAGAAGTGCACAAACT CCTATA[C/T]GGATTTGTCTTGCAATCGTCAT
TP57774	6H	126.70	bowman_contig_1986938	TGCAGGTTTGTATCAAATAAGTGGTTTGGAGCTTTG GTGTCCAGTGGAATTTT[G/T]CTCGGGCGAC
TP53464	6H	126.70	morex_contig_55855	TGCAGGTA[A/G]TCGAACAGCTCCCTCTTCTTGCCGT CGCGCTCCGAGATCGGAAGAGCACACGTCT
TP52135	6H	126.70	morex_contig_55855	TGCAGGGGGAGGCCAACTCGTCGCTGGAGCT[C/G]C TGCACAAGTGCGCCGAGGTGGACAGGAAGGA
TP42401	6H	126.70	bowman_contig_87490	TGCAGGAGGTGGCCAGTGTCGCCCTCGTTGT[C/G]A AATCCCATAGCCGAGATCGGAAGAGCACACG
TP41372	6H	126.70	morex_contig_269568	TGCAGGAGATAAAAACCAAGCCTGTGGGAGGAAG[A/G]CCGAGATCGGAAGAGCACACGTCTGAACT
TP41371	6H	126.70	bowman_contig_78535	TGCAGGAGATAAAAACCAAGCCTGAGGATGGAAGG CCGAC[A/G]CGAGGAAGGCTGGTGGTTTGTGG
TP40166	6H	126.70	barke_contig_56433	TGCAGGACAG[C/G]AAGGCAAAGCAAAGTCAGGGG

Marker_ID	Chr.	cM	Barley_Contig	Sequence
				CACGAATGAATCCAACTGAACCGAACCTGAAA
TP29738	6H	126.70	barke_contig_55198	TGCAGCGGATCTCACTCCACTCATCGGTGACTGTGAGGTCAAAGCACGGAGCCCAC[A/G]CTGACGT
TP24200	6H	126.70	barke_contig_134907	TGCAGCCGTACGCGCGGCAGCGGGTATCTCCGCGGCCGCCGCGTCGCCGTCGACCTCTCGT[C/T]GT
TP20339	6H	126.70	bowman_contig_13674	TGCAGCCAACGCCTCCCTCTCCTACTCCGCTCCAGCGCCA[C/G]TGCCGCCGAGATCGGAAGAGCAC
TP13756	6H	126.70	morex_contig_46523	TGCAGCACCACAAACACCAAGTCAGCGACATCATATACATGCTTCTC[G/T]CTCAGCAACTGAAACT
TP44317	6H	126.70	morex_contig_57887	TGCAGGCAGGCAGGAACAGGGCAACACCAAAAGTTTCAGTCAGGTACAAAATTGAACATGG[C/T]CA
TP60043	7H	0.00	bowman_contig_142790	TGCAGTCCATCCATGGCATTGGACGGGCACGCGCCCGCCAGATCCTTTCAGAG[C/T]TCAATTTAGA
TP2350	7H	0.00	barke_contig_275930	TGCAGAAGCTGAAAGATTGGAGTAGCACCGTGGTGTACGTCGCTGGCACGCTCTGACCAAA[A/G]CC
TP3030	7H	0.00	barke_contig_52828	TGCAGAATCGAAGGGCTGAGTATGTCTCAG[A/C]AATTCTAGAGAAGCTTGTGTCGTGGGAAATGGT
TP5230	7H	0.00	-	TGCAGACGGGCAGGACCATGACCTAC[A/G]ACCTCCAACTCCAAGACGGTGCCGAGATCGGAAGAGC
TP5814	7H	0.00	bowman_contig_222246	TGCAGAGAACTACAAGTGACATGGG[C/T]TGGATTGTGGATGCCACCAGTAATTCGTCCAGTTTTTT
TP6296	7H	0.00	bowman_contig_9428	TGCAGAGATTATT[A/T]TTTTGTACTCACTCTGCTTTGCTCTGTTTCTGTTTTTCCCCTTCATATCT
TP6333	7H	0.00	morex_contig_40276	TGCAGAGCAAAGTACCTCAATTGTTGAAGATGTTAACCATTGCTTGGGC[A/G]AGTGTCTGGTCAGA
TP7271	7H	0.00	bowman_contig_164394	TGCAGAGGAACGAAGACCGATTGATGAGCATTGCTGCTGGTGTCAGC[C/G]GCATGAGATAAGGGGG
TP12874	7H	0.00	morex_contig_300315	TGCAGCAAGGAGTCGATTGGCCTAAT[A/G]CCACCAACATAATAGGATTCTTACTTTGTTCTTGCAG
TP13570	7H	0.00	morex_contig_41777	TGCAGCACAGCGGCGAGGTGAGCGCT[C/G]GTCCCGCCGATGAGCCAGAACTGCTCATTCCTCCACC
TP14096	7H	0.00	morex_contig_43514	TGCAGCACCGCACGCACGA[C/T]AGCTTAAATGCATCACCTGTGGCGTCCAATGACGCTCCATTGCA
TP17633	7H	0.00	-	TGCAGCAGGAGCGATAGTAGCAAGTACTTAACCGAGCCACCTTGAATAATTAAG[C/T]GTGGCCGCC
TP18745	7H	0.00	bowman_contig_61715	TGCAGCAGTGTCG[A/G]CGGACGGACGAGGCGAGGAAGCAATCCGAGATCGGAAGAGCACACGTCTG
TP18839	7H	0.00	bowman_contig_11475	TGCAGCAGTTGGAACCTGGCGGCGTCGACATGGAGCTTGAGCGCGCTCATGT[A/C]CCAGCCCCCCG
TP23026	7H	0.00	bowman_contig_883846	TGCAG[C/T]CCTGTGACGTTGTCCCTGTAGAAGTAGTGCACCGTGGTGAGCTCCAACGTAGCCCCAT
TP23247	7H	0.00	bowman_contig_883846	TGCAGCCGAGCAAC[A/G]ACGCGCTGGCCATCATGGTGCTGGTGCCCCCCACGCCCACGGTGAGCGT

Marker_ID	Chr.	cM	Barley_Contig	Sequence
TP25755	7H	0.00	bowman_contig_1992273	TGCAG[C/T]GACAGCGACACGGTGCTGAACGACCTC GACTCCCGCGCCTCCGAGATCGGAAGAGCAC
TP29038	7H	0.00	morex_contig_39067	TGCAGCGCGTGCTGGACGAGCGGCGGGCCAGGGGA GGGAAGAC[A/G]GCGGCGGGTGCGGCGGCGCC
TP29150	7H	0.00	barke_contig_300067	TGCAGCGCTATCTCTTGCTCTTCCGTGAAGCTTTCCG TCCCCCA[A/G]TGAGACCAGACTTCAAGTG
TP31597	7H	0.00	bowman_contig_222575	TGCAGCGTCATCGTTGT[C/T]GTCGGCGGCGCCGCCC CAGCTCCAGCTGACGAAGCTCTTGGCGCCG
TP33279	7H	0.00	bowman_contig_864293	TGCAGCTATTGTTGCAAAAGGCGGGGATGCCTTCAC TTCTCCATCGACTCCTCATGCAG[A/G]ATAT
TP38518	7H	0.00	morex_contig_37137	TGCAGCTTTTTCTCCGA[C/T]CTCTTCAGCAGGTCTG TTATCTCATGCGTGAGAACCTCAATTGCCC
TP43007	7H	0.00	barke_contig_266341	TGCAGGATCTCGCTGTTGATGGTGAGCTTGAAGCCG ACCGCGATGG[A/C]GTTGAGGTTGACCATCA
TP44975	7H	0.00	barke_contig_2823051	TGCAGGCCAGGTTTGTGGCCGACGTGGCTCCCCTGG C[C/G]CTGGGCGTTGTGCAGCAGGCCTTGCG
TP48114	7H	0.00	barke_contig_1788463	TGCAGGCGTAGTTTCAGCTGGCCAAGTAT[A/T]GAC CAGGCACCAAGGGAAAATTACGCCGCTTATG
TP49234	7H	0.00	morex_contig_46236	TGCAGGCTTCTTTCTTTTGACCGCCATTGTGAAGAG TGGACCTTTCA[C/T]GGATCCTCTCCTGTAT
TP49737	7H	0.00	morex_contig_46236	TGCAGGGACTTTTTGGACGTCCTGGTTTCTCTTGA[C/ G]GATGCAAACGGACAACCGTTGCTCTCCG
TP49887	7H	0.00	morex_contig_12243	TGCAGGGAGGAGATGAGGCTAGTTGATCCAT[C/G]G AGTAAAGCCGAGATCGGAAGAGCACACGTCT
TP52500	7H	0.00	-	TGCAGGGGTTGAAGCAGCGTTACGAAG[A/G]CCACC ATGGACTGAAAATTCACGACGACGCTCTTTC
TP52509	7H	0.00	bowman_contig_103664	TGCAGGGGTTGGAATCACCCTGTTGAAATCCGCAA GCCGAACACC[A/G]TCCTTGGTGTGCTTTTCC
TP58365	7H	0.00	morex_contig_1577866	TGCAGTACCGCGGACGACACGATA[G/T]ATCAGATC TGATAAACACATACCGCATAATTTAAACTAA
TP58479	7H	0.00	morex_contig_57798	TGCAGTACGCGGACGACA[C/T]GCTTATCATCTTCCT TGTTGATCCGAGATCGGAAGAGCACACGTC
TP58906	7H	0.00	bowman_contig_9428	TGCAGTAGGAAGGAACCAACCAAAAAATCAAGATC AATTCAGCAACAGCTGCGTGCC[G/T]TCCTCC
TP60049	7H	0.00	-	TGCAGTCCATCCTTTCGCCGC[A/G]CCGCGCCGTTGG GTTGGGTTGGCATTTCCCAGATGGTCTCAG
TP60121	7H	0.00	morex_contig_80182	TGCAGTCCCCACCGCCATCGTCAGGGTACTACGCGT CCTCGGAGGGTTC[A/G]GACGGGTACCTGAC
TP61675	7H	0.00	bowman_contig_25598	TGCAGTGAGGTTGCTCGTGCGACCTCACTCTCCTCC[A/G]TCGGCGCTTCATCTCCTTTGCCTACCG
TP63567	7H	0.00	morex_contig_1563311	TGCAGTGTGTTCATTT[A/G]ACGGAATGAGTTGATTG CTGATTCGAGTGTCGACTCATTTCAGGGAA
TP64258	7H	0.00	morex_contig_140449	TGCAGTTCCATGCAGCCCACAAAAGTGCACAAGTT[

Marker_ID	Chr.	cM	Barley_Contig	Sequence
				C/T]CAACCCGAGATCGGAAGAGCACACGTCT
TP1449	7H	1.30	morex_contig_718103	TGCAGAACGCCGCGTCGCCCCTGTACATGTAGGCGTGGCGGTCCGAG[A/G]CGATGCGGCGCTTGCA
TP2072	7H	1.30	morex_contig_2528380	TGCAGAAGCAGAGAAG[A/G]AAGCGGTCCATGGTGTGGCAACGGTCGGTGGAGCTTACGGAGCGGGG
TP4810	7H	1.30	bowman_contig_78045	TGCAGA[C/T]GCACGGCCAACGGTGAGGGGCGAGCAGTAGTACACGGCTGGCACGTCGGTCTTGTTG
TP16778	7H	1.30	barke_contig_1792629	TGCAGC[A/T]GCGCAGGTGGAGCATGGAAGCACATGTCGTCGATGCCGAGATCGGAAGAGCACACGT
TP33073	7H	1.30	bowman_contig_65032	TGCAGCTAGCTGGCTTAGATCGTTTCCAACTTAACTATTGACAAC[C/T]GAAGCACATGACCAGCTT
TP44300	7H	1.30	bowman_contig_877413	TGCAGGCAGGAGCAGATGGACATGGACGCCGC[G/A]CTGGCCGCCGTCGCGCGCCGAGATCGGAAGA
TP47291	7H	1.30	-	TGCAGGCGCGTGACTGCTAGGCATCCAACGAGGAGC[C/G]GAGCGGCAGCGTGGTGGCAGTGTGCTT
TP60520	7H	1.30	barke_contig_2780678	TGCAGTCGATCGGCAGGCTATGTGGAGCGCGACGTCTTCCTTCTGGGAAGATG[A/G]TACGTTATAA
TP61955	7H	1.30	bowman_contig_877413	TGCAGTG[C/T]AGAGCGCTGGAGTAGTGATCGGTCGGCCATGGAGTCGAGGCACGGGAAGGCGGCAT
TP58638	7H	1.30	-	TGCAGTACTTCGAAGCGGCCA[C/T]GGGAACGGAGTTCAAGGTGCCGAGATCGGAAGAGCACACGTC
TP35443	7H	1.30	morex_contig_136340	TGCAGCTGAGGAAAAGAGAGGTCCCGCCGCTGCC[A/G]ATGCCGACCGAGATCGGAAGAGCACACGT
TP37026	7H	1.30	morex_contig_1572075	TGCAGCTGTACAGCCCGAGCCACGGGACGGCGGCGTCGCTGAAGACGGA[C/G]GTCCACCAGGCCAT
TP47587	7H	1.30	morex_contig_49664	TGCAGGCGGACGAGGTAGAGCGAGGGGGCCATCTTGCTCTTGAGCAT[C/G]GGCGTGAGCAGGTATC
TP62459	7H	1.30	morex_contig_1574023	TGCAGTGCTCACCGTTTGATGACAAGCC[A/C]GCGGCTGTTGGTCCGCTCCACGAACCTCTCCGAGA
TP3181	7H	3.90	bowman_contig_144193	TGCAGAATGCCAGGCAGCTCTGTAACCAAGGTGGT[A/G]GGTGACGGAAGGTGAATAGATGATCTTC
TP4582	7H	3.90	morex_contig_122840	TGCAGACCTGTAAAGTGATGCAA[C/G]AAGGTTGAAAAACTCAGCAATTTTCTATCTTATTGAACTT
TP13518	7H	3.90	bowman_contig_850798	TGCAGC[A/T]CACTGGCTTCGCACCCCACGAGCGGGCAACCGAGATCGGAAGAGCACACGTCTGAAC
TP688	7H	3.90	bowman_contig_10020	TGCAGAAATAATTCTGCAACAAGGTTTGTATTTCAATGATGAATGCGTGCTT[C/G]GCTGCTTGACA
TP2512	7H	3.90	bowman_contig_95121	TGCAGAAGGCACGGGA[A/G]CTGGCAGCGTCTGTGCCAGCAGAGGCGGTTCCCGTGCTCCTTCCAGC
TP4491	7H	3.90	bowman_contig_11044	TGCAGACCTAGGAACAATAGC[A/G]ATAGCCTTATCTGCTGTGCCGAGATCGGAAGAGCACACGTCT
TP12917	7H	3.90	morex_contig_1607885	TGCAGCAAGGCTCCATC[A/C]CCTCTTCTGCTCCGTCTCTGTTCAACCCCTGTCCTCCAACCGCCCC

Marker_ID	Chr.	cM	Barley_Contig	Sequence
TP13517	7H	3.90	bowman_contig_850798	TGCAGC[A/T]CACTGGCTTCGCACCCCACGAGCGGG CAACCGAAAAAAAAAAAAAAAAAAAAAAAA
TP17176	7H	3.90	bowman_contig_276908	TGCAGCAGCTCACCGCCGCATG[C/T]TTCCTCGGCCC CATCGCCTTCTTCCGAGATCGGAAGAGCAC
TP19462	7H	3.90	morex_contig_2548032	TGCAGCATCTCGTTGCTCCACGGGAACCCGTCGGCA TCGG[A/C]GCCGAGATCGGAAGAGCACACGT
TP19831	7H	3.90	bowman_contig_10020	TGCAGCATGGCGTTGCTCCACGGGAAGCCGCCGTTG GCGGAGTAGGCCCCCGA[C/T]GTCTTCTCCG
TP22827	7H	3.90	bowman_contig_10112	TGCAGCCCGTGCACGATCCCCGTGGC[A/G]AACGTC GCGAAGCTGATCTCCTTCTTCTTGCCGCCAG
TP26003	7H	3.90	bowman_contig_62956	TGCAGCGACGGGTGAACATGGACTTG[C/T]TGCGGT ATGGCAGCGGATTGGGAGCACGACAAAGTCT
TP29953	7H	3.90	bowman_contig_276908	TGCAGCGGCATACAGTGGTGCAAGAGATAGAGGGA GCTG[C/T]GCGGTGTAGTGCTGTGCTGTAGGT
TP34396	7H	3.90	-	TGCAGCTCGAGACCATG[A/G]CCGACATGAAGCGCG AGTCGGTGGCGCTGCATGAGATGGTCATCTC
TP37241	7H	3.90	barke_contig_271919	TGCAGCTGTTCAT[C/G]GGTTGGCTTCGATGATGGG GCTCTACGGTGAAATCGGAGTCATCCGTTCA
TP41993	7H	3.90	morex_contig_39172	TGCAGGAGGAAGGCGGCCGCGCGCGCGGGGTTCTC GGGGGTCTACGGGGC[G/T]CGGGGCCACGCCC
TP47115	7H	3.90	bowman_contig_120362	TGCAGGCGCCGTTGTTCGTGGAGACCAA[C/T]ATGA CGTTGGGGGTGGCTGCTTCTCGCAAGCGACG
TP63437	7H	3.90	bowman_contig_9981	TGCAGTGTCTGGGCCATCCATACCCTGTTAATAACA ATTCAAAAATAAA[A/C]TCGTTATATTCCAC
TP63543	7H	3.90	bowman_contig_856044	TGCAGTGTGTAACTGGCTAATTAGCCACCAAACCCA TACAGGCTTGA[A/T]ATTTCCCACATAGGGT
TP978	7H	3.90	barke_contig_366025	TGCAGAACAACTTATTGATCAATGCGTCGACAACA GACCTTCTCTTTGATAT[A/G]AACACTACAAA
TP17196	7H	3.90	bowman_contig_202627	TGCAGCAGCTCCCAGCCGTCGAGGAA[C/T]TACATT TGACAAACTGCCCCAAGTTGTGTGGAGCCTT
pathogenesis	7H	27.1		CCGAGAATGCAGACGCCCAAGCTAG[C/T]CATCTTG CTCGCCCTAGCCATGGCA
pbr1	7H	28.1		GACGCCCAAGCTAGCCATCTTGCTC[C/G]CCCTAGC CATGC3CAGCCGCCATGGT
TP3502	7H	42.61	morex_contig_134884	TGCAGACAA[C/T]GTACAAGGAAGGCTGTTTATCTG AGGCGGCGGAGGCATCCATAGTTTCCTTCAT
TP1240	7H	42.61	morex_contig_274279	TGCAGAACC[C/T]CTTCCTCTACGTCCTCCTCAGCCA GGGGATCTCCAGGCGGCAGATCGTGTTCGT
TP4217	7H	42.61	bowman_contig_1983791	TGCAGAC[C/T]CGCCTCCCCGATGACCTCTCCGTGAC CGTGTTCCGTCGCCAGCCGAGATCGGAAGA
TP5709	7H	42.61	barke_contig_403715	TGCAGACTTGCAGCAGACCGCC[A/G]CACGGTGCCT GAACAACATACCACATGTCCCGAGATCGGAA
TP10034	7H	42.61	barke_contig_119107	TGCAGATCTTGGCCATGGACATCAACCGCGAGAAC

Marker_ID	Chr.	cM	Barley_Contig	Sequence
				TACGAGCTGGGG[C/T]TGCCGTGCATCGAGAA
TP20397	7H	42.61	morex_contig_2600128	TGCAGCCAAGCCCCA[C/T]CTATAAAAAATCTCCGA CGCATCTCCAGCCCGTCCGAGATCGGAAGAG
TP24875	7H	42.61	morex_contig_140545	TGCAGC[C/T]TCTCCACTAGTCCCCAAACACAAAGC AGCAGGTTCAGAAACCACAGCAGCAACCACT
TP29601	7H	42.61	-	TGCAG[C/G]GGACCGCTCCGCAACAGCCTTTGACAT CCCGAACATCTGAGCAACACAAACCAAGTGG
TP33251	7H	42.61	morex_contig_244362	TGCAGCTATGGGCTGTGGCTAGTTGGTTATAATTAG GGGGGGAAATCATTTCGTAATTAACAT[A/T]
TP33901	7H	42.61	morex_contig_137884	TGCAGCTC[C/G]CGAAAATTTGTGTTTGCTGTTGCTG GTCGAAGGCCAGTGCCCCAACCATAAAATA
TP52053	7H	42.61	morex_contig_56149	TGCAGGGGCTCGGCCTCGTGCACCCGCAGGCCGTG CACCGCTTCTACGACGAGCT[C/T]CACGCGTA
TP61195	7H	42.61	morex_contig_49594	TGCAGTCTGCATCCCCTTGACCCAATGTCCGCATGA G[C/T]CTAAGAACTCGGATGTGCAAGAATGG
TP61659	7H	42.61	bowman_contig_16630	TGCAGTGAGGCGACCGATCGAATAACACACAGTGC CATGATTG[A/G]CGAACACAGAGCAGACACCT
TP62025	7H	42.61	bowman_contig_876452	TGCAGTGCAGTGCAGACAGTGCGGCCAATTAACTC GTGGTTATCCATGAAGATCTAG[A/C]GTTATA
TP63291	7H	42.61	barke_contig_1783906	TGCAGTGTAAATAAAAA[C/T]AGTGTATTTTCATCAT GGACAGGGAGAAGATGTATTTGAGGCATTC
TP10787	7H	47.82	morex_contig_2547471	TGCAGATGGCTGCAACCA[C/T]GCCGCCTGCAACTT GGGCCCATTTTTCGTGGTTTTAATCGGCCTT
TP11488	7H	47.82	bowman_contig_64572	TGCAGATTGGAAA[G/T]GTTGCAAGGAAACCTGAAG GAGCAGAGAGGTTACCGCCGAGATCGGAAGA
TP15643	7H	47.82	barke_contig_67211	TGCAGCAGAGCTTCACGTCGTTGTGCTCCTGGAG[A/ G]CGCCGCCGAGATCGGAAGAGCACACGTCT
TP22194	7H	47.82	barke_contig_132498	TGCAGCCCCAAGGGGTGGGGATCA[C/G]AGGGGATT TGATGACCCGCTCCACCCATTTTCCGAGATC
TP27651	7H	47.82	bowman_contig_1988792	TGCAGCGCCGACCGCAGCTTGGCGGCTTCCTCTGCA TCATTGGATGCAGACAGCAGGCC[C/G]AGGT
TP40078	7H	47.82	morex_contig_2547568	TGCAGGACAAGGTGGTGTACGGCACGGCGGACCAT GG[A/G]GGGCCGTGCGCGCCCTTCGCCAAGAG
TP42381	7H	47.82	morex_contig_41188	TGCAGGAGGTGCAGGGAGAGGC[G/T]TCGGAGGCG GCGCATCGCCGAGATCGGAAGAGCACACGTCT
TP62503	7H	47.82	barke_contig_66494	TGCAGTGCTCTGCT[A/G]TGGCATCGCCTGATGGGA ACCAAGGTCCTGGAAGCGAGGTTCACCGAGA
TP53304	7H	47.82	barke_contig_372067	TGCAGGGTTTCAAACCCAACTCTCTCTCTCTCTC[A/ G]TCTGAACATATATATACATATGCAGCACT
TP24611	7H	47.82	barke_contig_67211	TGCAGCCTCAG[C/T]GAAAAGCTGCGCGTAGAGTCT CTCGGTGGCTACGTGGACGACGGCTACCTGA
TP35729	7H	49.12	bowman_contig_238685	TGCAGCTGCATCGCAACTCACAAG[C/G]AACGTTAA CACGGTGTACGTCCGTCGCCGAGATCGGAAG

Marker_ID	Chr.	cM	Barley_Contig	Sequence
TP1540	7H	50.42	barke_contig_756104	TGCAGAACGGTTTCGGTGGGCAAGAAAGAACCAGCGCCATACTACTCGCGTCGCGTG[C/G]CCCCCT
TP6175	7H	50.42	morex_contig_45950	TGCAGAGAGTGCGCGACGACGGCGGCCGAGCGGTTGGC[G/T]TCGATGAGAGGCTGCACGCCGAGAT
TP15395	7H	50.42	barke_contig_2002164	TGCAGCAGAAGTACACATGGCCAACTCCC[C/T]GTCAAGCTGGCCGAGATCGGAAGAGCACACGTCT
TP23372	7H	50.42	morex_contig_2547134	TGCAGCCGCAACATCTCAAACCTCTAGGTGGGACCAAATCCTACGACTGCCCAAGAACC[C/G]AAGA
TP25650	7H	50.42	bowman_contig_75801	TGCAGCGAAGGGATTCGGGCCTTAATTAGACGGGGATTACGTGCGTGGCAGCGTAGTA[C/G]GAATG
TP25759	7H	50.42	bowman_contig_1996090	TGCAGCGACAG[C/T]GATGTCCGTGGTCGTCACCCTCCTCCTTCCGCGGATCTCCCATCCGCCTCGA
TP43614	7H	50.42	bowman_contig_1991407	TGCAGGATTTGAGTTTATTTGATGGGTGCTTCTCCT[C/T]GACCGAGATCGGAAGAGCACACGTCTG
TP58996	7H	51.72	morex_contig_1560116	TGCAGTAGGTTTGGCACAATTA[A/G]CAAAAAACTGAAGTCCATGCAAGGCAACTAGCTATTCAGCT
TP4764	7H	51.72	bowman_contig_270926	TGCAGACGATGATGGCACTCTTGTGCCT[G/T]CCACCTCGCCTTCTTTCTCCTCCGACTCGTACGCG
TP7914	7H	51.72	morex_contig_135845	TGCAGAGGTTGTCCAA[C/T]CGCAGATTGATGGGTGTTACGACAACGAATCGGGGACAGGAGCAGCC
TP8125	7H	51.72	barke_contig_1782197	TGCAGAGTGC[A/G]TCCCTGACGAAGCTGGGCGCCGAGATGGCTGCACGGACAACGTGCTCGCGCCG
TP12659	7H	51.72	morex_contig_52505	TGCAGCAAGACGGTCAGTGCCACCTCCT[C/T]CTCCATCCACCAAAGCTTTCTCCACGGCGACATGC
TP14821	7H	51.72	morex_contig_135128	TGCAGCACGGGCGGGAGCCAGCGAGTTAGTTACG[A/G]CCGCGAGGGTTTGCGGGGCGCGGGGACTG
TP19529	7H	51.72	bowman_contig_900075	TGCAGC[A/T]TCTTGGCTTACCTCTCCGTGCCCAAGCTCGTGGAGCTTGGCGAGGTTCCCATCCCGA
TP20520	7H	51.72	morex_contig_1560212	TGCAGCCAATTTGTCAT[C/T]GTACTTAATAACAAGTATGGGAATTTGAACGCTTACGATGCCTTAG
TP40153	7H	51.72	barke_contig_349487	TGCAGGA[C/G]AGAAGTACGATGACATGTGAATCAATCCAAGAGGGAGCGGTTTCCTCAATCTACAA
TP43675	7H	51.72	barke_contig_370060	TGCAGG[C/T]AAAGAGCTGAAACAGATAGTCTGGCCAGGCGAAACGGCCACCGTGCCGATTGGGTGG
TP50545	7H	51.72	bowman_contig_900075	TGCAGGGCCACATCAACTCGATGCTTCATTTCGC[C/T]GCGGGTCTCCTCGACGCCGAGATCGGAAG
TP56077	7H	51.72	bowman_contig_847047	TGCAGGTGCTCGCGGGCATCCTCAACGTGTGCGCGCTCGTGGG[C/G]TCGCTCACCGCCGAGATCGG
TP60160	7H	51.72	barke_contig_1796573	TGCAGTCCCT[C/G]ATAAGTTTCATCGACGAGATTGACAGGCAGGAATCCACGTACGTTCTCTCTAG
TP64388	7H	51.72	bowman_contig_902616	TGCAGTTCG[A/G]AATCAAGCTAGTCAAACCAGATCGATCGAAGGATCGAGTACGTTAATTCATCAT
TP14993	7H	54.32	morex_contig_136277	TGCAGCA[C/T]GTTGATCCACTGTTAAGCAACGTGC

Marker_ID	Chr.	cM	Barley_Contig	Sequence
				ATACAGGACACATGAGCCGAGATCGGAAGAG
TP15174	7H	54.32	bowman_contig_846025	TGCAGCACTGTAAGCGACATCTTTGTTTC[A/C]TTGT AGTCGCGGCGTCGTCCCATATCCTTCCGTC
TP19081	7H	54.32	morex_contig_2549457	TGCAGCATCACGCCGCAACTGTTGTTGGCTTCGCAC CAACTCTCCATGA[A/T]GATGATGGCATCGC
TP26925	7H	54.32	barke_contig_715696	TGCAGCGCACTATTTC[A/G]CCCTACAAAATCCTGTT TTATTGCACTGTAAACGATTTTTGCGGGTC
TP45209	7H	54.32	-	TGCAGGCCCAGCGCGCGCCGCAGCCCGCGCCCTAC CTCCTCCACGAA[C/T]CCCGAGATCGGAAGAG
TP49492	7H	54.32	bowman_contig_855106	TGCAGGGAAGGCGGCGCCAACATGATTTGCTCGGC TTCGTTTCCTCCCACCAAC[A/G]GGCATATTG
TP59689	7H	54.32	-	TGCAGTCACTGTCACTTCAGCTTCAATACAACGATA ACATGCAATTA[C/T]TTGGCTCCTCTATAGG
TP28646	7H	54.32	-	TGCAGCGCGCTCGAGAAATCAATCAACGCCAAGAT GAGTCAAATCAA[G/T]AAACAGAATAAAATCG
TP5266	7H	58.22	-	TGCAGACGGTAGATCAGAATTTGAGGCAAAGTAAC ACCAAAAAAAGA[C/T]TACTACGTGCTAGCAT
TP32563	7H	58.22	bowman_contig_849072	TGCAGCTAACTCCATGGCCTTTAACTGTGCTGGACG TCCACTCACATGTT[A/G]TGCTAGATGTGCG
TP778	7H	58.22	morex_contig_135454	TGCAGAAATGCACAGTTGCTGA[A/G]AAATTACCAA CAACTAGCTCAGCAATCCGAACGCGTGGTAA
TP23822	7H	58.22	bowman_contig_861345	TGCAGCCGCGATAGACGGACCGATCCATTCTGTTTT TAGAGAAAC[C/T]TCAAAAAGGTGGGTCAAC
TP1620	7H	58.22	-	TGCAGAA[C/T]TCCAGCCGCTGTAAATTCAGCATGA ACATGGCGTTGGATATCCCTCACTAAACCGT
TP4043	7H	58.22	-	TGCAGACCACTAGAATGACGACCATGCCGCGTGGA TCTC[A/G]GGCACTTTCGTCGATGTGGGGTAG
TP7777	7H	58.22	bowman_contig_852829	TGCAGAGGGGGGCCATCCTGGAGCACGCA[C/G]TGG AGAAGGAGGCCAAGGGCGCCGCCGAGATCGG
TP8088	7H	58.22	morex_contig_37191	TGCAGAGTGAAGCAGTGACTGCATGTACTGGAGGT TGGCA[G/T]CCTGGGCTGTCTGCTCTTGCAGC
TP11347	7H	58.22	barke_contig_349158	TGCAGATTCTGCCGATCCGTCAAA[A/G]GAATCGTA TGCCGCGGCAGAGGATTTTTTCCTGCCATCA
TP13726	7H	58.22	-	TGCAGCA[C/T]CAAATAGGATTTGCCCGCAAAGAGC GTCATATAGGTGCTCCCTTCCAAAAGATCCC
TP14040	7H	58.22	morex_contig_54207	TGCAGCACCGAACTGGATGCGTCGTTGGTTGCATGA GGAAGTTGCAGAAATGGTGCCT[C/G]TCATC
TP16541	7H	58.22	barke_contig_15520	TGCAGCAGCCAGCCAGCCAGCCAGACAGCAAAAAA T[C/T]AGTTCAGGACTAGTCGATCAAGAATTG
TP18066	7H	58.22	-	TGCAGCAGGGAGCAA[C/T]GGCAATGGCGTCGCGGT GAAGCAGCAGCAGCAGCAGCAGCTTGGAGGC
TP18924	7H	58.22	-	TGCAGCATACGCGCCTCCGCC[A/G]CCGTGGCTCGC CTCACTGGCCCGCCTCCACAGCAGCGGCCCC

Marker_ID	Chr.	cM	Barley_Contig	Sequence
TP21345	7H	58.22	bowman_contig_1223400	TGCAGCCAG[C/T]GGTACTACCGCCAGGACCACAGC GGTACTACCGCCGAGATCGGAAGAGCACACG
TP37684	7H	58.22	barke_contig_681542	TGCAGCTTCGTCCTCCACTGTGAGGACAA[A/G]TTA CAATGATGATATCGTGCCACCGAGATCGGAA
TP39018	7H	58.22	morex_contig_54207	TGCAGGA[A/G]CCATTCGTCGAGATACCCGATGAGA AAATCCGAGATCGGAAGAGCACACGTCTGAA
TP41965	7H	58.22	barke_contig_328005	TGCAGGAGCTTGTCGTCCACGCCCCAGCAGCCGCGC AGGTC[C/G]AGGAACTTGAGGTCGTGGCACC
TP42029	7H	58.22	bowman_contig_1322559	TGCAGGAGGAGATGGCCAAAACCCTGCGGGGCGTG GAAGCGGAAGCCGCCAGGATGG[C/T]GGCACA
TP44480	7H	58.22	morex_contig_1574953	TGCAGGCATAGATAGGGGCTATGG[C/T]GGATGCCT CATCTGAAGAAGCAGCGCCGTGGCCGCGCAC
TP50294	7H	58.22	-	TGCAGGGCA[C/T]CGAACCTTTAGCAGCAAAATAGC AGGTGTCAGTCATATGCATTCGGAGGCAAGG
TP53014	7H	58.22	-	TGCAGGGTGGATCCGCGTCCA[G/T]CTCCAGCTCCA ATCCACGGTGAGCGGGTCGGCCGAGCCGAGA
TP55742	7H	58.22	barke_contig_68005	TGCAGGTGCAGAAAAAAGCATCATCTTTCTACCCAA AAAAA[A/C]CCCCGAGATCGGAAGAGCACAC
TP61211	7H	58.22	morex_contig_48455	TGCAGTCTGCGGTCTGCACCC[A/G]TCACCGTCAGTC ATCGCACAACGATCGGATCGACAGTGAGCT
TP35956	7H	58.22	barke_contig_2289641	TGCAGCTGCGATGAGAGGAAAGGAATGACCC[A/G] CGAGATTCTGTTGACCACATCGAACGAGCGAC
TP49448	7H	60.07	morex_contig_137938	TGCAGGGAAGAAGCGGTGTATATGGA[A/C]CCAAGC AGCATGGTTCGATGGATGGCAGACGTCGTGC
TP34122	7H	62.72	barke_contig_395800	TGCAGCTCCTCCAGCATCTGCTGGCCTACATGGAAT G[A/G]CTTGAAATGTTCAGCAAACTCTAGGA
TP49703	7H	62.72	-	TGCAGGGACTAATGGAAGCGA[C/T]GTTTTTGTATG CAGACCACGCAAACGGCCAAAGGGAAGGGGA
TP8699	7H	63.29	barke_contig_265380	TGCAGATAGCAAAAAAGTTGC[C/T]ACTCCCAGAGA TAGCCGAGATCGGAAGAGCACACGTCTGAAC
TP15206	7H	63.29	-	TGCAGCACTT[C/T]GAGCGGGAGCACCACGACAAGG TGCGCGGGTGGGTGGGGTTCTCCGTGCGCCT
TP24370	7H	63.29	morex_contig_2045184	TGCAGCCGTGTCAAGTTGCAGGCGTTTGCCCACCTC TATGGCG[C/T]AGCGCAGCGCAGGAGCAACA
TP27242	7H	63.29	barke_contig_484159	TGCAGCGCCAAATCGGATTTGCCTTTCCCCAGTCGA AGGATATCCTTA[C/G]GTCACTGGCCCAGCT
TP51084	7H	63.29	-	TGCAGGGCGCGAGCGGGCGGCCGCAGAGGCACTTG TTGGGCGCGAAGTGC[C/T]TGGGGCCGAACCT
TP53007	7H	63.29	barke_contig_1781877	TGCAGGGTGGACTGGCATGGCACGGACATGCATGT GGCGT[C/G]CGTTATTAAACTGATTGATCCAC
TP59843	7H	63.29	barke_contig_281461	TGCAGTCATATTGG[C/G]TTTCCTCGATCGCACGTCA GCACCTCATCGTTAGTTCAACTCCATGTAA
TP61845	7H	63.29	barke_contig_1785894	TGCAGTGCAACCCACTTGATTG[C/T]CAGCAACTGG

Marker_ID	Chr.	cM	Barley_Contig	Sequence
				TCGCTGGAGCTATCACCAATCACGAGCCACC
TP58516	7H	63.29	-	TGCAGTACGTAGA[C/G]CAGCCGAACAAGGAGAGC AACACGTGAGCGGCCACAATAGGCTGGAGGAG
TP28221	7H	64.59	-	TGCAGCGCGAAATAAGATTTCCCA[G/T]TTGGAGCC AGTACCGAGATCGGAAGAGCACACGTCTGAA
TP55815	7H	64.59	barke_contig_1810563	TGCAGGTGCATACACACCGCGAGTGGGAC[C/G]TGA TAAGCATACGAAGGACAAGAAGTGGACAACC
TP8213	7H	64.59	morex_contig_50040	TGCAGAGTGTGAA[C/T]CTTGCAGAGAGCATTTTAC AATATTACTGGGTCTGAACTAGACGGTACCC
TP42518	7H	64.59	barke_contig_269315	TGCAGGAGTCGGAAAGTTACAGGAGCAGAGACGGA TGGGGTAGGTGATTGGCCGAGTGGCT[C/G]AG
TP22938	7H	67.19	bowman_contig_1305932	TGCAGC[C/T]CTCCTTAGTCTTTTCTCGATTCGGCGG TATTGTTGGATTGAAGTGGTCTGGACCAAC
TP36669	7H	67.19	morex_contig_160365	TGCAG[C/T]TGGCCTTGTTCAGACATAATAACGTAAT GAGCAGGTGTATCTGAGGTTACACAGTTGG
TP42798	7H	67.19	bowman_contig_865093	TGCAGGATCACCTTCTCGTGATCGGACGT[C/T]GAA GACGATGAGGAACTCGAAGGCTGGAGCTGCT
TP44977	7H	67.19	bowman_contig_862842	TGCAGGCCAGTACGATCAAAA[A/T]CCTATTGCCAT GAATTACATTTCACTTATTAGCTGTACGACT
TP63191	7H	67.19	morex_contig_48256	TGCAGTGGTCGACCCATGAGGTATGCTATTCGGTAC CAACACTTCATTCTCAACATAAGGTT[G/T]T
TP39842	7H	68.49	-	TGCAGGAATCTCGTAGACGGGTTGTTTGCT[G/T]CCT AGAATCTCGGCAATGAACCGAGATCGGAAG
TP40897	7H	68.49	morex_contig_2553047	TGCAGGA[C/T]GTAAGAGGACCGTTCAGTTCATGCT GAGTAGCAGTTTTTACCACCTTTTAATTTTA
TP53452	7H	71.09	morex_contig_1567895	TGCAGGTA[A/C]GGAAAATCACATTATCAGTTCGTG TTTTGCAGCTGTGGATGCTTGTGGCCTTTTC
TP8704	7H	71.09	bowman_contig_866777	TGCAGATAGCACTAGTGCACCTGATCCTCTTTTCTT[C/T]CCCCCCTACTGCTGCCGTTCTGTTCGA
TP16092	7H	71.09	bowman_contig_270877	TGCAGCAGCACGTCCACCAT[A/G]TCCCTCGCCGCG AAGCTCTCCCCTTCCATTCGCCGAGATCGGA
TP18054	7H	71.09	morex_contig_42803	TGCAGCAGGGACAGCGTACACACTGCGCGCAC[T/C] ACGCGCAGGACAGAGGAGGGGGCGCTTGCTT
TP34869	7H	71.09	morex_contig_157691	TGCAGCTCGTCGATCACCGCACGGCCGCCCGCGTGG ATGCAGAAGTGCTCGAACGCCATGCG[A/G]A
TP38417	7H	71.09	bowman_contig_1981570	TGCAGCTTTGGATGGTG[A/G]CGCGACGGCGCCACG ATGGTGGCGGTGCACCGACGTGGAAGGATCT
TP55342	7H	71.09	barke_contig_1854775	TGCAGGTCTGTAACGCATGCATGGTCCGAGCA[A/C] CCGAGATCGGAAGAGCACACGTCTGAACTCC
TP30771	7H	72.39	-	TGCAGCGGGATCCCGCGAGGCGGCTA[A/G]GGTACG CAGGCGGCGCCGACGAGATCCGAGATCGGAA
TP3256	7H	73.69	morex_contig_50681	TGCAGAATGTCAATGGTACGGGCGTA[C/G]GGCATA CCTGCACGGTATGAACGAATGTCAATGGTGC

Marker_ID	Chr.	cM	Barley_Contig	Sequence
TP11089	7H	73.69	barke_contig_1781940	TGCAGATGTTTATGGAGAACGATGGAAGTTGTGAGGA[C/T]ATGGGTCCTCGTGCTTTCCCGAGATC
TP12266	7H	73.69	barke_contig_1854775	TGCAGCAACCACA[C/T]GTGTCTTTCGTAAATCATGCACCGAGATCGGAAGAGCACACGTCTGAACT
TP17492	7H	73.69	morex_contig_101827	TGCAGCAGGAACACCTCCTCCTCCATTGCACGTGT[C/T]GACTCGATGACCTTGTGGCTGCTGCGCG
TP18473	7H	73.69	morex_contig_1559127	TGCAGCAGTAATCGGTGTCAATG[C/T]CACTATAACCAACAATGTCTATAACACTAAGCTCCCTAGC
TP23633	7H	73.69	morex_contig_343347	TGCAGCCGCCCCGCACCACCCATGAGCC[C/T]AACTCCCTCCTCAATGCAGCCGACGGGAGTGGAAA
TP32654	7H	73.69	morex_contig_62319	TGCAGCTACACTGCACCACACTCCACCCTCCTCACCAACTACACAACCA[G/T]GCAGTTGCACGCTC
TP38559	7H	73.69	barke_contig_318169	TGCAGGAAAACAGGCACATGTTTGATCTTGTTATCAGTGATGT[A/G]CACATGACAGACATGGATGG
TP39236	7H	73.69	bowman_contig_849108	TGCAGGAACTTCCAAGCCCAGCATCAAGAATCTAATGGTCCCTGCTTCCACTCCACC[G/T]CCACCT
TP43513	7H	73.69	barke_contig_1781940	TGCAGGATTCCATTCTCATCTTCCT[C/T]GCTGTGCCTGGTGCGCCACCAATGCCGATGAGTGTGCT
TP43794	7H	73.69	bowman_contig_855915	TGCAGGCAATCAAC[C/T]CATAGAGCGACAGTGTGAACCAACGCGGTTTCCTCAGTAATCACACGGC
TP46236	7H	73.69	morex_contig_158676	TGCAGGCCTAGCAAGCTAGCTAGCTAGTGGCCTACTACAGAAAACATA[A/G]GTCTACCTAGTAGAG
TP47548	7H	73.69	-	TGCAGGCGCTTGTGCAG[A/G]GTCTCGAAGCCGCGCAAGTCCTCCTTCAAAGCCTCCACGTCCGAGA
TP49402	7H	73.69	bowman_contig_1487581	TGCAGGGAACAGGAACCGTGAGCCGCGGCTGACTCGGACGGAGAATCTATCCGTTCGGA[A/G]CGGG
TP54288	7H	73.69	bowman_contig_862842	TGCAGGTCAGTACGATCAAGAACCTATGAATTGTGTTTTATGAGTATGTG[C/T]TCTGTCTGCCGAG
TP56918	7H	73.69	morex_contig_40019	TGCAGGTTACCATTAGGGACGTAGTTGTAGAGCAAGAGCTT[G/T]ACAGACTTGTTCGAGCAGTAAC
TP60554	7H	73.69	morex_contig_70903	TGCAGTCGCACACCACCGTGCACACCGACTGCACCGACGA[C/G]TCCGAGATCGGAAGAGCACACGT
TP62108	7H	73.69	barke_contig_317578	TGCAGTGCCAC[C/T]AGGTAGCGTGTGTCATACCATATGACGTGCCTGAGGACTGAATCCCACCGAG
TP66056	7H	73.69	bowman_contig_859124	TGCAGTTTTG[A/T]CAATTCTTTTTGGGATTCGTAGTACGGAATCCCTGGATAGGGGTTTGCTTGCT
TP17765	7H	73.69	morex_contig_138415	TGCAGCAGGCCAAACTTGAGTTGCAGTTCTTTTTGA[A/G]GGATTGCTCACTTTTTTTAGTTCTTT
TP65273	7H	73.69	bowman_contig_896268	TGCAGTTGGCCGCCTACGTCACCCTCTGCGAGG[A/G]CTTCCAAGGGATCGAGCCGCGCCTGGACCT
TP4996	7H	74.99	bowman_contig_142178	TGCAGACGCTCCGTTTCG[A/T]TGTTAGGAAAACACTCATCTGGCAGTTTAGCACCAGGTACCAAAT
TP6975	7H	74.99	bowman_contig_127825	TGCAGAGCGGGCAGCTGGAGTGGGAGGCAAGCCAC

Marker_ID	Chr.	cM	Barley_Contig	Sequence
				ATGTCGACGCACTCGGTGTGGAA[A/G]CCGTG
TP10186	7H	74.99	barke_contig_338946	TGCAGATGAGA[A/G]GAGCAGCAGCAGCAGAAGAA GAGTTAAAAAAAAGTCAACACCACGATTACAA
TP15693	7H	74.99	-	TGCAGCAGAGTTACATAGTAGC[A/G]TCCCGTCAGA CGTCGGCAAGATGCGGGGGTTTGCACAACCA
TP15706	7H	74.99	bowman_contig_1179618	TGCAGCAGATAATGGGCTCCGTGCACAGTGCGAAA AGCAATACACA[C/G]AATATGCACTAGTAGAA
TP21751	7H	74.99	morex_contig_43876	TGCAGCCATGAGGGTAAAGAGTTGATATGA[C/T]CA AGTTTGGTGACTCTGCATATCGGGTGAACCC
TP50553	7H	74.99	bowman_contig_127825	TGCAGGGCCACCGTCGGCAAG[C/G]CCGACGCGTCG CAGGCGCTTACATCGACGAGTCTCCCTTCCC
TP62329	7H	74.99	bowman_contig_15910	TGCAGTGCGCGCACACCACGCCAGGC[G/T]CCACAC GCGGGAATGCGTCCCGCATTTCAGCTTTGTT
TP62566	7H	74.99	morex_contig_2547915	TGCAGTGCTTCACCATCAGCAGCACCACCACCA[C/G]CAGCAGCAGCATCACGTCGTGGACGAGCAC
TP63022	7H	74.99	barke_contig_1837716	TGCAGTGGGACACTTAGCGGATAA[C/G]TGTGCGTC TCGTTTCTGCACAAACTTTAACCATTTTGAC
TP65515	7H	74.99	bowman_contig_291120	TGCAGTTGTTGACCAGTCCCATGACCTCTCCGAAGT C[A/G]GCCAGGATGGCACTAGTAGAAAAGAG
TP37759	7H	74.99	-	TGCAGCTTCTCGCTGGCCAGCACCTCCTGGCGCCCG CAAGCTGTCGTTGC[C/G]TGAGCTTGCAGTC
TP7689	7H	74.99	morex_contig_61296	TGCAGAGGGAGATAGGCAGGTCCAGTTTATCG[T/C] GCCCCTGTGCGTTTGCCCAACAATTTGATAC
TP29775	7H	74.99	-	TGCAGCGGATTTGCTATCATTCACCCGATAGATTGA TAGATGCTAGT[C/T]CCGAGATCGGAAGAGC
TP45010	7H	74.99	barke_contig_71884	TGCAGGCCATCAACAAACAAAAACAGGGTAA[C/G]T AAGCAGTATTGGACTCTGATTCTTCTTCTCG
TP45323	7H	74.99	bowman_contig_291243	TGCAGGCCCGA[C/T]GCGGTCTCGAATATGCCTAGG ATGCCCTTCACAGCGAGCACCTCATCCATCT
TP19444	7H	74.99	barke_contig_2793114	TGCAGCATCTCGCGTGGTCACCCTCGATCACCGC[C/ T]GTCAGGCGCAGCGGCTGCCAGTGGAGAAG
TP33415	7H	74.99	bowman_contig_145211	TGCAGCTCACCGAAGAGGACGCAGAGGCTGCCAAC ATCAGTGGTAAGATCA[C/T]CGATGGGATTGG
TP59818	7H	74.99	barke_contig_2781556	TGCAGTCAGTTCTCAAATGTTAT[A/G]TACTGCCTTA AAATATATTTTAGAATCACATGAAGAGAGG
TP6362	7H	76.28	bowman_contig_879126	TGCAGAGCAAGCAGCAGCGAACGAGGCGGGCATCC ATGGACGT[A/G]CGGCGCGTTCTCTCGATACG
TP3304	7H	77.26	morex_contig_56831	TGCAGAATTCAA[C/T]ATGAAGATACATTAGGACCA ATATCTGGAAATGTTGCTTCAGTCTGCTGCA
TP48571	7H	84.19	bowman_contig_1983479	TGCAGGCTCCAAACTTTGCCCGCATGTCGTGTA[C/T] CTTGGTGGCATGTAACAAACTCTATTCTCC
TP2529	7H	90.73	barke_contig_2787307	TGCAGAAGGCCCTTTATATGCCTGCAAAATAATC[G/ T]CCAGTTTATGGTTTTGTGTGAAAAAAGTG

Marker_ID	Chr.	cM	Barley_Contig	Sequence
TP10725	7H	90.73	-	TGCAGAT[G/T]GCAGGTGCCATTGACGTTGAAATCA AACAGTTCGTAAGCAGAGCGGCGACATACGT
TP22582	7H	90.73	bowman_contig_857366	TGCAGCCCGAATAAGGTTTGTTGCCCAAATTATAAA ATTTTAACTCTAAGGTAATGCTTCCGA[G/T]
TP31140	7H	90.73	-	TGCAGCGGTACTACCG[C/T]TGATCCCATGGTAGTG CAAGGACACAACCGAGATCGGAAGAGCACAC
TP60511	7H	90.73	bowman_contig_11574	TGCAGTCGATAGGCCCAGCTCTAGCTTTAGATCAAC TCGGGCCCAACTC[C/T]AGCTTCCGATCGAC
TP26119	7H	92.02	morex_contig_39091	TGCAGCGAG[A/G]ATCAAGAGGAGAGGAAGAGCCT GGCGATGCCTGGTTGCGTGCTGCGTGCGTCTG
TP24997	7H	92.02	bowman_contig_869648	TGCAGCCTGAGGGAGCGGCGTTCGGGAACTACCTC ATACAGTACGCCTTAGC[C/T]CTGGTGCTGAA
TP860	7H	92.02	morex_contig_83111	TGCAGAAATTGGGTAGTAGGC[C/G]GTGTGCTCATA CTTTTGCACACTTGCAAAGAAAACTCGACAA
TP8249	7H	92.02	bowman_contig_63887	TGCAGAGTTATGCCAAGGGCACCGTGGAGGACTCT GCTCGCTGACTGT[A/C]TCTGCTGGCAGCTGA
TP11682	7H	92.02	bowman_contig_946769	TGCAGATTTTAGTTTCAATA[A/C]TTCGCAAACCAGA TCAGTAACATTCAGATGGCTCAGATAGGAA
TP15356	7H	92.02	-	TGCAGCAGAAGCACGCTCTGCTGCTTCGTCGATGTT CAGAGTGCAGACTACTCTCCACTGTTA[A/T]
TP46245	7H	92.02	morex_contig_57291	TGCAGGCCTCAAGTTTTGTTTTTGGCATGTGATAAC TAGGGTTGCTG[C/T]TAGACAAGGAAGATCG
TP55063	7H	92.02	bowman_contig_70129	TGCAGGTCGG[C/T]GGCGGAGACTGGCAGCCATCAC ATCCGAGATCGGAAGAGCACACGTCTGAACT
TP4849	7H	93.29	barke_contig_1806244	TGCAGACGCAGTGCCGCGCGAA[A/C]ACAGCAAAC ATTTTCTTCTAGTTAATCACCAGAAACACGAA
TP32029	7H	93.29	-	TGCAGCGTGCCAGCAGCGGCGGCGACATG[A/G]CGA CGGAGCCGAGATCGGAAGAGCACACGTCTGA
TP38324	7H	93.29	barke_contig_1800968	TGCAGCTTTAGTA[C/T]TATTCATCACCATGTGGTAA GTTCCGCTCACTCAAGTTATTGTTGGCAGG
TP38352	7H	93.29	bowman_contig_75231	TGCAGCTTTCCTGCTAGCCGAGGTACGAGGGCTTGG GGCAGGTCGATCTAGGGTGTGCGGCGG[C/T]
TP19	7H	95.86	morex_contig_302433	TGCAG[A/G]AAAAACGAAAGCGACTTCGACGAACA CGGGACGCTGTCGACTGTCTGATCAACACATG
TP31899	7H	95.86	bowman_contig_62443	TGCAGCGTCTTCATCTGCCCGACCTCTCG[C/T]GGGA TCGCGCCGAGATCGGAAGAGCACACGTCTG
TP37594	7H	95.86	morex_contig_1572438	TGCAGCTTC[C/T]TGGCGAACCTGCACAGGCACCGC CTCACCGCGTGCCCCTTGGGCGCGGCCGCGT
TP41029	7H	95.86	barke_contig_2786562	TGCAGGACTGAATCCTAAACAAAAAATAGTTGG[G/ T]CCACGCTGGAAAAATTGCAAGCAGTGCGCC
TP61791	7H	99.93	bowman_contig_12253	TGCAGTGATGTGATAA[C/T]AACAATATCAGCAGAA TTAGAACAGTTACACAAAAACACTGAAGTCG
TP35770	7H	100.78	bowman_contig_2078905	TGCAGCTGCCAAATTGTACCACCA[C/G]ACCCTAGC

Marker_ID	Chr.	cM	Barley_Contig	Sequence
				TGTGCCTCTAATCCTCTACAACTCCGTCGAT
TP1246	7H	107.64	morex_contig_58412	TGCAGAACCCGACGCTAG[A/G]CTCGTACTGTATGT GGTACACCGTGTGGCGGGCCGAGATCGGAAG
TP16772	7H	107.64	bowman_contig_958852	TGCAGCAGCGCACTGCTCTCCTGGCCACGGCGAGG CCCCGCGGAGGCGGCGGAGCT[C/T]GGACGAT
TP19933	7H	107.64	morex_contig_38701	TGCAGC[A/G]TGTCGGCGGCCACGAGGCGGCCGTTC ATGGCGTAGGGGTCGTTGGTGGAGACCAGGT
TP22896	7H	107.64	bowman_contig_66971	TGCAGCCCTAGCTAGAGGCCCCATC[A/C]CCATCCG TCATAGCTGCTTGCGACAAAGCCGTCGGAAC
TP23249	7H	107.64	-	TGCAGCCGAGCAAGCAAAGCGAGGGGAAATCCTAT TTAGCGTCACAGG[C/T]GCCGAGATCGGAAGA
TP29882	7H	107.64	morex_contig_76302	TGCAGCGGCAGCAACCTCGTCGAGGACGCGCGCAT AAGCATCACTCCTCCGAGCGGACCTC[A/G]GC
TP32444	7H	107.64	bowman_contig_147432	TGCAGCGTTGGTGCTACCCTATTGCAGCTTCACCT[A /G]TTGCCGAGATCGGAAGAGCACACGTCTG
TP36198	7H	107.64	morex_contig_60065	TGCAGCTGCTCGCACCCCCGTGCTCGACCGAGGCTG TCGCTGTTGCTCGCGCCCCCGTGC[A/C]TGG
TP41643	7H	107.64	bowman_contig_10186	TGCAGGAGCCATCGTGTTTGCAAAGAAA[C/G]TGCG GAGAAAGCCGTGCACATTAGATGCCGACAAA
TP48444	7H	107.64	-	TGCAGGCTAGTATTTTCATTTTTTGTATTGTTGGCCT GGTAGGA[A/G]ATAACCGAGATCGGAAGAG
TP56311	7H	107.64	bowman_contig_1982368	TGCAGGTGGCAAGCCAGGTGATAAACTGGCTTCAC GAGGAGACACAGGTCCTGGGGATGAGCG[C/T]
TP62093	7H	107.64	morex_contig_83798	TGCAGTGCATTATAA[C/G]ACGATTACTACAAAACG AGAAGAAACTGAAATATTACGCCTCGAGCGC
TP26585	7H	110.23	-	TGCAGCGATGAAGAGCGGCACGGCGGCGCTCCATT GCAGCGT[C/G]GACGCAACCGAGATCGGAAGA
TP37776	7H	110.23	barke_contig_119580	TGCAGCTTCTG[A/G]TCTTCACTTTCTCGTGAATTTG TATGTGGTGAAGAAGTTGTATACAGCTCGC
TP41023	7H	110.23	-	TGCAGGACTCGGCAGCCAACACGACGGAGATCCTG AT[C/T]CATACACGAATCCGAGATCGGAAGAG
TP47373	7H	110.23	morex_contig_83798	TGCAGGCGCTCTCGAGGGAGCAAGAAGGATAGGGA CAAG[A/C]AGAAGATAGCCGAGATCGGAAGAG
TP28351	7H	118.07	-	TGCAGCGCGAGCTTCCTGTAGTTCCAC[C/G]CCGTGT AGGCCTCCGAGATCGGAAGAGCACACGTCT
TP32265	7H	118.07	morex_contig_158332	TGCAGCGTGTCCGCGTCACGATTCAAAGCGGTGGTG CCCGTCAGTCAGGCTCCACGATGGG[C/T]GG
TP52533	7H	118.07	morex_contig_43850	TGCAGGGTAAATTGAGCAGGAAATTACAGCGGTTA TG[C/G]GAACCTCCGAGATCGGAAGAGCACAC
TP62533	7H	118.07	morex_contig_158332	TGCAGTGCTGCTGCGGAGGCAGGGGAAGGAAGGAA GGT[A/G]GGCAGGGGGAGTCCGAGATCGGAAG
TP24955	7H	120.35	-	TGCAGCCTGAAGCTCCTTCGTCCGCCGCTTTGGGTG ACAGCTC[G/T]TGCAGTGCTTCCGTCCCGAT

Marker_ID	Chr.	cM	Barley_Contig	Sequence
TP14866	7H	128.55	morex_contig_212778	TGCAGCACGGTGGTGAGCCTGGGTTTTCTGTATCTTCTCAACAGGAGCGA[A/G]GAGCACACCACGC
TP53737	7H	128.55	bowman_contig_201101	TGCAGGTAGACACCAACACACACCACCGTTA[A/G]ATGAATGATCCAGCTCACGCGCCGCCGTTGAA
TP55582	7H	128.55	barke_contig_2782945	TGCAGGTGAGGTGATGGAGGGGCACGTTAATATATTCA[G/T]GTGGTTCAGCTGGCCGCGTGATCCA
TP29488	7H	129.85	barke_contig_57841	TGCAGCGCTTTAATCCATATAATAGTTTT[C/T]GGTATTTTGATAGCCTCTCCGAGATCGGAAGAGC
TP39245	7H	129.85	morex_contig_62194	TGCAGG[A/G]ACTTGGCCGTGGGGAAGCTGACGGCGAAGGAGAGGGGGCCGAGATCGGAAGAGCACA
TP56832	7H	131.08	barke_contig_410719	TGCAGGTGTTGCCGCACCGCATGATCGATGTGGACGTACGCTGAGTGAATGAACGC[C/T]GTCATTC
TP63570	7H	133.75	-	TGCAGTGTGTTGTCGGCTACTAACA[A/G]CAGGAGCAGCACGGGTCGGCCACGAGCGGGAGCGGCAG
TP7367	7H	133.75	morex_contig_1560296	TGCAGAGGAGCAAATCAATGGACGGATCGGAGCGGTGGCA[A/T]TGGCACGTACCCCCTGGCCGTCT
TP22215	7H	133.75	morex_contig_132551	TGCAGCCCCACCGTCTGCTCGCGTCGCA[G/T]CCCCTCTGCCTGCTCGCGCTGCCCCGCCACCAGCT
TP22538	7H	133.75	morex_contig_38058	TGCAGCCCCTGTCCCA[A/G]TCGTCCAGCAGCACGTAGAGATGCTGCTGCTGCCGCCGCTCGAACGG
TP30904	7H	133.75	morex_contig_38226	TGCAG[C/T]GGGCTGGCAACTACACCGCCAGCACCCAGCCGCTCGTCGCCGCTCCCCTGACTCCCGA
TP38574	7H	133.75	morex_contig_53679	TGCAGG[A/G]AAACGGGGAGTAGGTGCTGGACGAGCTAGGGTCGAAGAGCGGGTCCGCCTGCGGGTG
TP46524	7H	133.75	barke_contig_274885	TGCAGGCGACACCAGACAAAACGCACGCAAGACCATTGCTACATACAGGGGCTGTTGCCGATT[G/T]
TP46616	7H	133.75	bowman_contig_860394	TGCAGGCGAGCTTGAATGCCT[C/T]CCGACGTGGCTACGTACGTACGTACCTACGTACACGACCAGC
TP46642	7H	133.75	barke_contig_274885	TGCAGGCGAGGTAATGGAATTCCCTAAGCTCATTGCGTCAAGATGTC[A/G]CTGTTTCACAACTAGC
TP48418	7H	133.75	-	TGCAGGCTACGTG[C/T]CTGGCCAGCTACACGCACGGATGGCGTGTGACCGAGATCGGAAGAGCACA
TP49474	7H	133.75	morex_contig_38941	TGCAGGGAAGC[G/T]ATTCTTTGAGGAATTGGATAGAGATGGTGATGGCGAAGTTACCCTAGGTCTC
TP51639	7H	133.75	barke_contig_2152046	TGCAGGGCTTTTGGATCGCCAGAGCAATGCCATCCGAGATTCCCAC[A/G]GGGGAACTGTGTGTACA
TP57226	7H	133.75	barke_contig_57104	TGCAGGTTCTGCTTCAGGTCTCCCAGGCACTCTATCAGGAACTGCGCCCTCAATGCTGCCACA[A/G]
TP63213	7H	133.75	-	TGCAGTGGTGAGCATG[C/G]GCGTTGCCCTGTTATCACGCCGAGATCGGAAGAGCACACGTCTGAAC
TP64696	7H	133.75	bowman_contig_9692	TGCAGTTCTGCCTGACA[C/T]GACACCATCATTTAGAAGTAGATGACGTCTCATCAACTGTATCCTC
TP5326	7H	133.75	morex_contig_54769	TGCAGACGT[A/T]GTACTCTAGTATTGAACTATTGAT

Marker_ID	Chr.	cM	Barley_Contig	Sequence
				CATGCGTCTTGGAGCATCTCCAACTCCAAC
TP7943	7H	133.75	morex_contig_63520	TGCAGAGT[A/G]ATCTTCAAACTTACTTGTGCCACA ACCACAACGGTCGTCGTCGTTGCCTGCCTCT
TP36934	7H	133.75	barke_contig_662270	TGCAGCTGGTGATGAAGAGCGGCAAGTACAC[C/G]C TCGGCTACAAGACCGTCCTCAAGACGCTCCG
TP38302	7H	133.75	barke_contig_662270	TGCAGCTTGTTGTTGATGTTCTCCGTGCTCTTCTTCT GCACA[C/G]AGTCGGAATCGAGGTCCGTCA

SNP is shown in square brackets

Table S3 The primer sequences used to amplify SSR markers used in the genetic linkage map

Marker ID	Chr.	cM	Forward primer	Reverse primer
GMS021	1H	10.8	CTATCACACGACGCAACATG	CCTGAGAAAGAAAGCGCAAC
Bmac0213	1H	20.7	ATGGATGCAAGACCAAAC	CTATGAGAGGTAGAGCAGCC
Bmag0872	1H	31.6	ATGTACCATTACGCATCCA	GAAATGTAGAGATGGCACTTG
Bmac0032	1H	43.7	CCATCAA AGTCCGGCTAG	GTCGGGCCTCATACTGAC
Bmag0770	1H	45.5	AAGCTCTTTCTTGTATTCGTG	GTCCATACTCTTTAACATCCG
Bmag0211	1H	45.5	ATTCATCGATCTTGTATTAGTCC	ACATCATGTCGATCAAAGC
Bmag0347	1H	46.9	CTGGGATTGGATCACTCTAA	AA AACAAGTACTGAAAATAGGAGA
Bmag0718	1H	47	ATCGTGACATCTCAAGAACA	CCTGATACTGCCTAGCATTAG
HVM43	1H	53.4	GGATTTTCTCAAGAACACTT	GCGTGAGTGCATAACATT
EBmac0501	1H	70.9	ACTTAAGTGCCATGCA A AG	AGGGACAAAAATGGCTAAG
EBmag0793	1H	98.2	ATATATCAGCTCGGTCTCTCA	AACATAGTAGAGGCGTAGGTG
Bmag0222	2H	28.5	ATGCTACTCTGGAGTGGAGTA	GACCTTCAACTTTGCCTTATA
GMS002	2H	46.2	CCGACAACATGCTATGAAGC	CTGCAGCAAATACCCATGTG
HVM3	4H	0	ACACCTTCCCAGGACAATCCATTG	AGCACGCAGAGCACCGAAAAAGTC
HVRCABG	4H	8.2	ACACCTTCCCAGGACAATCC	CAGAGCACCGAAAAAGTCTGTA
EBmag0781	4H	9.4	CTATTTTCTAATGCTTGGACC	TGTCTAGTTCATCATCATTGC
Bmac0181	4H	9.4	ATAGATCACCAAGTGAACCAC	GGTTATCACTGAGGCAAATAC
Bmag0375	4H	9.4	CCCTAGCCTTCCTTGAAG	TTACTCAGCAATGGCACTAG
EBmac0906	4H	17.5	CAAATCAATCAAGAGGCC	TTTGAAGTGAGACATTTCCA
HVM07	5H	15.9	ATGTAGCGGAAAAAATACCATCAT	CCTAGCTAGTTCGTGAGCTACCTC
Bmag0337	5H	23.4	ACAAAGAGGGAGTAGTACGC	GACCCATGATATATGAAGATCA
Bmac0096	5H	25.4	GCTATGGCGTACTATGTATGGTTG	TCACGATGAGGTATGATCAAAGA
Bmag323	5H	27.2	TTTGTGACATCTCAAGAACAC	TGACAAACAAATAATCACAGG
Bmag0005	5H	30.9	TCCATGATGATGTGTGCATAGA	CGGATCCCAACA A ACACAC
Bmac0113	5H	32.2	TCAAAAGCCGGTCTAATGCT	GTGCAAAGAAAATGCACAGATAG
Bmag0121	5H	34.7	ATAAGATAGGTCACCGCAATA	AGTAGTTCAATACAGACCTACAGG
Bmag0387	5H	43	CGATGACCATTGTATTGAAG	CTCATGTTGATGTGTGGTTAG
EBmac0557	5H	93.4	ATGCATGTGTAGATGTAGATGTG	AACAAGGATA A ACTAACATGGG
EBmac0615	5H	108.8	AATTGGTTCGAGTCATAGCT	CTAGTGGGTGTATGCAAGTG
EBmatc0054	5H	129.3	TGACCACCATTGTGAGACAG	AGTGGTAGTGGGAGGAGGAG
Bmac0040	6H	0	AGCCCGATCAGATTTACG	TTCTCCCTTTGGTCCTTG
Bmag0500	6H	20.3	GGGAACTTGCTAATGAAGAG	AATGTAAGGGAGTGTCCATAG
Bmag0120	7H	0	ATTTCATCCCAAAGGAGAC	GTCACATAGACAGTTGTCTTCC
EBmac0764	7H	7.8	AGAATCAAGATCGACCAAAC	AAA AACATGAACCGATGAA
Bmac0167	7H	18.3	CATTTCCACTTCAAAATATCC	CCAAAGTTTGAGTGCAGAC
Bmac0187	7H	22.5	GCTCTCTCTCAGAAAAATGAA	GAATTATTCTAGGGCTGTGAA
GBM1464	7H	32.8	ATAGCCGTGCTCTTGCTCAT	CAAGACCACCATTTGCATTG
Bmag0010	7H	38.8	AGTAGTTCACCCTTGGGGCT	AGCACGTGATACATCAAGAACG
Bmag0914	7H	46.1	GGGCAATATACAGTTCAACTC	ATGAACTGGAGGCAGTAAATA
AF022725A	7H	61.1	AGTATGGGGAATTTATTTGG	GCTGCAAAGTATGACAATATG

Primer sequence were either obtained from the following databases
http://www.genetics.org/cgi/content/full/156/4/1997/DC1, http://germinate.scri.ac.uk/ssr/barley_s.html,
http://bioinf.scri.ac.uk/ssr/ssr_table.html
and http://wheat.pw.usda.gov/cgi-bin/graingenes/browse.cgi?class=marker

Table S4 The annotated genes within the QTL marker interval between TP18054 and TP11089

Gene	cM	Description
MLOC_22078.1	68.06	Heat shock protein DnaJ, N-terminal
MLOC_5322.3	68.06	Cellular retinaldehyde-binding/triple function, N-terminal;Cellular retinaldehyde-binding/triple function, C-terminal
MLOC_63870.4	68.06	Zinc finger, CCCH-type;Zinc finger, C3HC4 RING-type
MLOC_37511.2	68.41	Helix-loop-helix DNA-binding domain
MLOC_74450.1	68.41	Serine-threonine/tyrosine-protein kinase
MLOC_43588.1	68.84	Glycosyltransferase AER61, uncharacterised
MLOC_69030.3	68.84	Aminotransferase, class V/Cysteine desulfurase
AK360719	69.11	Multi antimicrobial extrusion protein
AK371088	69.11	Ethylene insensitive 3
MLOC_14372.1	69.11	Peptidase C48, SUMO/Sentrin/Ubl1
MLOC_48422.3	69.11	C2 calcium-dependent membrane targeting;GRAM
MLOC_65913.1	69.11	Serine/threonine-protein kinase, active site;Serine/threonine-protein kinase-like domain;Protein kinase, catalytic domain;Serine/threonine-protein kinase domain;Protein kinase, ATP binding site
AK253105.1	69.26	Mov34/MPN/PAD-1
AK355136	69.26	ATPase-like, ATP-binding domain;Heat shock protein Hsp90;Heat shock protein Hsp90, conserved site;Heat shock protein Hsp90, N-terminal
AK369877	69.26	BTB/POZ
MLOC_3499.1	69.26	Pentatricopeptide repeat
MLOC_6357.1	69.26	Glycosyltransferase AER61, uncharacterised
MLOC_6476.1	69.26	Glycerophosphoryl diester phosphodiesterase
MLOC_65139.1	69.26	Ribosomal protein L31e
AK356978	69.33	Fatty acid desaturase, type 2
AK359310	69.56	Zinc finger, A20-type;Zinc finger, AN1-type
AK373386	69.56	Phosphofructokinase;Phosphofructokinase domain
AK373844	69.56	Pentatricopeptide repeat
AK374613	69.56	Aux/IAA-ARF-dimerisation;Transcriptional factor B3;Auxin response factor;AUX/IAA protein
AK375094	69.56	Short-chain dehydrogenase/reductase SDR;Glucose/ribitol dehydrogenase
MLOC_14286.1	69.56	Alpha/beta hydrolase fold-1;AB-hydrolase-associated lipase region
MLOC_18369.1	69.56	Pathogenic type III effector avirulence factor Avr cleavage site
MLOC_39328.2	69.56	FAS1 domain
MLOC_55370.1	69.56	Haem peroxidase, plant/fungal/bacterial
MLOC_55371.1	69.56	Glycoside hydrolase, family 19, catalytic
MLOC_60995.3	69.56	Ion transport
MLOC_64612.1	69.56	Zinc finger, Dof-type

Gene	cM	Description
MLOC_6490.2	69.56	Pentatricopeptide repeat
MLOC_6492.5	69.56	EXS, C-terminal;SPX, N-terminal
MLOC_6766.2	69.56	Protein kinase, catalytic domain;Serine/threonine-protein kinase, active site;Serine/threonine-protein kinase-like domain
MLOC_67834.1	69.56	Helix-loop-helix DNA-binding domain
MLOC_70730.1	69.56	O-methyltransferase, family 3
MLOC_75288.1	69.56	D-isomer specific 2-hydroxyacid dehydrogenase, NAD-binding;D-isomer specific 2-hydroxyacid dehydrogenase, catalytic domain
MLOC_80269.1	69.56	BURP
MLOC_80270.1	69.56	Nonaspanin (TM9SF)
AK355848	69.82	Heavy metal-associated domain, HMA;ATPase, P-type, K/Mg/Cd/Cu/Zn/Na/Ca/Na/H-transporter;ATPase, P type, cation/copper-transporter;Haloacid dehalogenase-like hydrolase;ATPase, P-type phosphorylation site;ATPase, P-type, ATPase-associated domain
AK248860.1	70.13	Methyl-CpG DNA binding;Zinc finger, CW-type
AK249904.1	70.13	Transcription factor, MADS-box;Transcription factor, K-box
AK353608	70.13	DNA topoisomerase I, C-terminal, eukaryotic-type;DNA topoisomerase I, active site;DNA topoisomerase I, DNA binding, eukaryotic-type;DNA topoisomerase I, C-terminal;DNA topoisomerase I, catalytic core, eukaryotic-type
AK354984	70.13	NAD-dependent epimerase/dehydratase
AK358287	70.13	RNA recognition motif domain
AK361661	70.13	Uridine kinase;Phosphoribulokinase/uridine kinase;Phosphoribosyltransferase
AK362747	70.13	Zinc finger, CW-type;Methyl-CpG DNA binding
AK365423	70.13	Serine/threonine-protein kinase, active site;Legume lectin, beta chain;Serine/threonine-protein kinase-like domain;Protein kinase, catalytic domain;Protein kinase, ATP binding site
AK365615	70.13	Protein of unknown function DUF3546;Zinc finger, C2H2-like;Arsenite-resistance protein 2;Zinc finger, C2H2
AK366556	70.13	Twin-arginine translocation pathway, signal sequence
AK369089	70.13	Bromodomain;SANT domain, DNA binding;Myb, DNA-binding
AK372126	70.13	Rab-GAP/TBC domain
AK372300	70.13	DNA mismatch repair protein;DNA mismatch repair protein Mlh1
AK373688	70.13	Endonuclease/exonuclease/phosphatase;Inositol polyphosphate-related phosphatase;WD40 repeat
AK377123	70.13	Serine/threonine-protein kinase-like domain;Leucine-rich repeat-containing N-terminal, type 2;Protein kinase, catalytic domain
MLOC_13746.2	70.13	Ribosomal protein L32e
MLOC_13859.1	70.13	Mitochondrial transcription termination factor-related
MLOC_13932.1	70.13	No apical meristem (NAM) protein
MLOC_15839.1	70.13	DSBA-like thioredoxin domain
